# Palladium catalysed C–H arylation of pyrenes: access to a new class of exfoliating agents for water-based graphene dispersions†

**DOI:** 10.1039/c9sc05101e

**Published:** 2020-01-28

**Authors:** Xavier Just-Baringo, Yuyoung Shin, Adyasha Panigrahi, Marco Zarattini, Vaiva Nagyte, Ling Zhao, Kostas Kostarelos, Cinzia Casiraghi, Igor Larrosa

**Affiliations:** Department of Chemistry, University of Manchester Oxford Road Manchester M13 9PL UK cinzia.casiraghi@manchester.ac.uk igor.larrosa@manchester.ac.uk; Nanomedicine Lab, Faculty of Biology, Medicine & Health, University of Manchester AV Hill Building, Oxford Road Manchester M13 9PL UK

## Abstract

A new and diverse family of pyrene derivatives was synthesised *via* palladium-catalysed C–H *ortho*-arylation of pyrene-1-carboxylic acid. The strategy affords easy access to a broad scope of 2-substituted and 1,2-disubstituted pyrenes. The C1-substituent can be easily transformed into carboxylic acid, iodide, alkynyl, aryl or alkyl functionalities. This approach gives access to arylated pyrene ammonium salts, which outperformed their non-arylated parent compound during aqueous Liquid Phase Exfoliation (LPE) of graphite and compare favourably to state-of-the-art sodium pyrene-1-sulfonate **PS1**. This allowed the production of concentrated and stable suspensions of graphene flakes in water.

## Abstract 2

Novel pyrene surfactants for aqueous graphene exfoliation were prepared *via* C–H arylation of pyrene-1-carboxylic acid.

## Graphical abstract

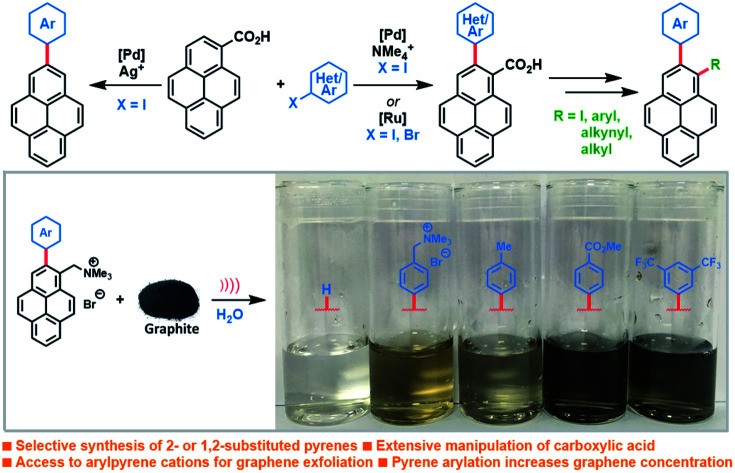

## Introduction

During the last few years graphene has emerged as a revolutionary material attracting much attention due to its unique properties and multiple applications.^1^ The first isolation of graphene by Geim and coworkers in 2004 using the “Scotch-tape method” allowed its identification and spurred many others to further investigate the material and develop new and more efficient methods of production. On this regard, LPE offers a straightforward approach to obtain graphene dispersions directly from graphite.^2^ LPE is based on exposing directly the material to a solvent with a surface tension that favours an increase in the total area of graphite crystallites.^2*a*^ Typically solvents used are NMP and DMF. However, LPE in water is also possible by using an exfoliating agent.^3^ This also allows to non-covalently functionalise the graphene flakes *in situ*, by tuning their surface chemistry depending on the exfoliating agent used.^2*i*^

**Fig. 1:**
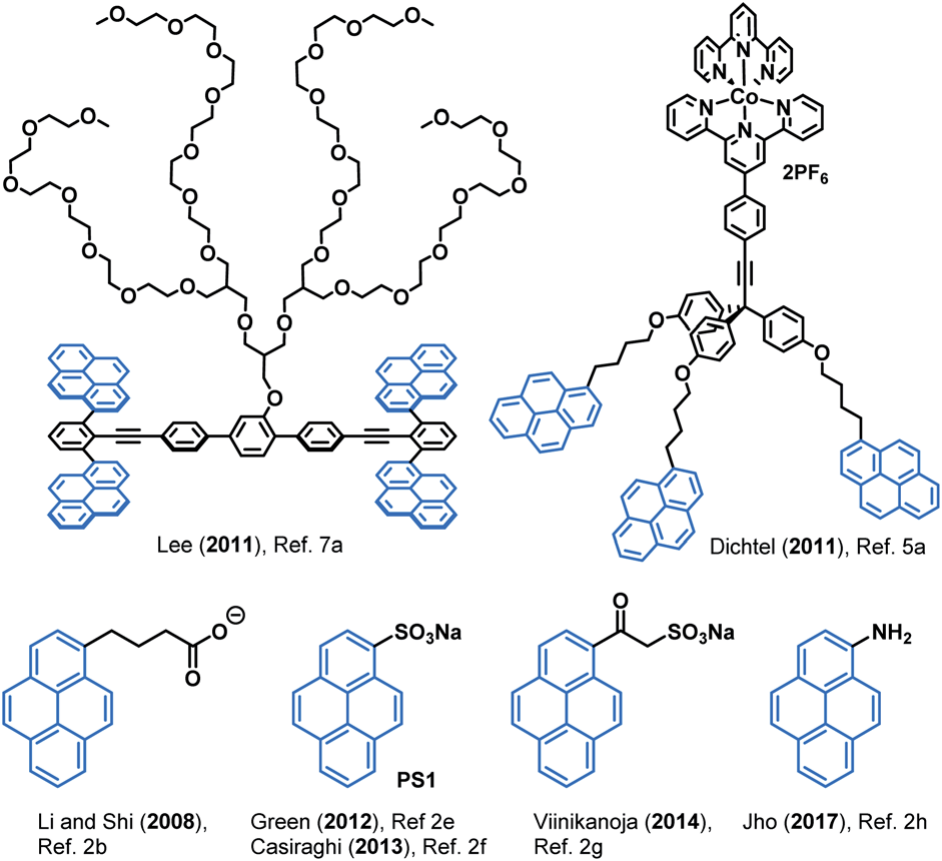
Selected pyrene-based compounds used in graphene non-covalent functionalisation and liquid phase exfoliation.

Non-covalent functionalisation of graphene offers an opportunity to add new motifs and tune the surface chemistry of graphene without disrupting its inherent conjugation and thus, its high conductivity.^3^ In this context, pyrene derivatives have arisen as a privileged scaffold due to its strong affinity for graphene.^4^ For instance, several types of pyrene derivatives (Fig. 1) have been used to develop graphene-based biosensors,^5^ water-based electronic inks,^6^ composite materials,^7^ light-harvesting devices,^8^ among many.

**Scheme 1:**
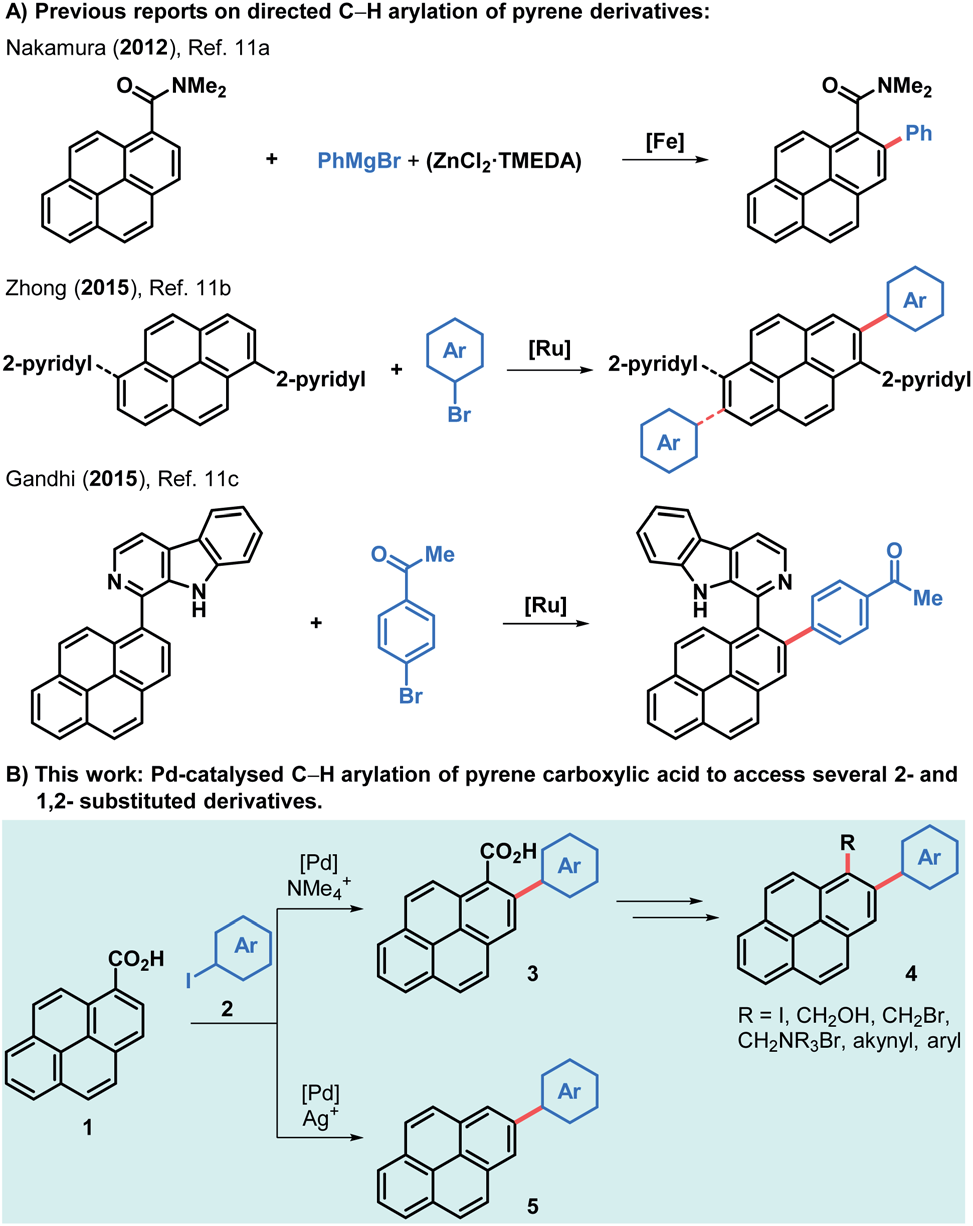
Overview of C–H arylation of pyrene derivatives.

Although reports on pyrene-functionalised graphene flakes are very abundant, examples of cationic pyrene-derived surfactants used to obtain suspensions of positively charged graphene flakes are scarce and require further investigation.^2*e*,*f*^ Most methods of pyrene derivatisation rely on electrophilic aromatic substitutions and its clear preference to yield 1-substituted pyrene derivatives.^9^ Nonetheless, several 2-substituted pyrenes have been produced after Marder and co-workers unveiled a method to generate 2-borylated and 2,7-diborylated pyrene,^10^ which can be extensively derivatised to obtain aryl, alkyl, alkynyl and amino derivatives, among others.^10*b*^ Only a few methods have been reported for the directed C–H arylation of pyrene derivatives (Scheme 1).^11^ Notably, the work of Zhong and co-workers reports a scope of 2-arylated and 2,7-diarylated products when one or two 2-pyridyl groups are present in the 1- or in the 1- and 6-positions, respectively. Moreover, the directed C–H alkynylation, alkenylation and borylation of aromatics have also been tested in pyrene substrates but only a handful of examples have been reported.^12–14^ In order to access a varied family of pyrene derivatives that can find use among the multiple applications outlined above we envisioned that commercially available pyrene-1-carboxylic acid (**1**) would offer a flexible platform to that end. The palladium-catalysed C–H *ortho*-arylation of aryl carboxylic acids offers an oxygen and moisture-compatible way to derivatise these common feedstocks while choosing the fate of the acid moiety, which can be preserved or removed during the arylation reaction by judicious choice of reagents and conditions.^15–17^ Thus, the use of a silver-salt additive can lead to decarboxylated products,^16^ whilst replacing it with a tetramethylammonium salt, the *ortho*-arylated carboxylic acids are obtained.^17*b*,*c*^

## Results and discussion

The feasibility of the proposed approach was first assessed using pyrene-1-carboxylic acid (**1**) and 3,5-iodoxylene (**2a**). After screening of the reaction parameters,^18^ both the 2-arylated pyrenecarboxylic acid **3a** and the corresponding decarboxylated 2-arylpyrene **5a** could be obtained in good yields (Table 1, entries **3a** and **5a**). Arylated carboxylic acids **3** were obtained in very good yields regardless of the substituents in iodoarenes **2**. Both electron-withdrawing and electron-donating functionalities were broadly tolerated; haloarenes were also obtained in good yields (entries **3g–j**) and the use of trifluoromethylated aryl iodides led to excellent yields (entries **3k** and **3m**). Ester-substituted products, which are amenable for further derivatisation, were also obtained in good yields (entries **3o** and **3p**). On the other hand, products of decarboxylative C–H arylation **5** were obtained in moderate to good yields in most cases and only in two particular cases the decarboxylative coupling proved inefficient (entries **5k** and **5n**). Nonetheless, the desired products could still be obtained in good yields by decarboxylation of the corresponding carboxylic acid.^19^ Importantly, this decarboxylative coupling can be a useful one-step alternative to access 2-arylpyrenes. Finally, using a modified protocol of our recent iododecarboxylation of carboxylic acids,^20^ arylated pyrene acids **3** could be further derivatised into the corresponding iodoarenes **4**,^18^ which grant access to a whole new family of unexplored pyrene derivatives (*vide infra*). In this case yields ranged from moderate to good, although as expected, the best yields were obtained among the most electron-rich substrates (**4a–e**). To our delight, some substrates bearing electron withdrawing groups and halogen substituents also gave very satisfactory results (**4h**, **4i**, **4m** and **4o**). In general, products **3–5** were obtained in good yields and reactions could be scaled up with ease (entries **3a**, **3b**, **3e**, **3p**, **4a**, **4b** and **4e**).

**Table 1:** Scope of C–H arylation of pyrene-1-carboxylic acid (**1**) and subsequent iododecarboxylation of 2-arylpyrene-1-carboxylic acids 3^a^

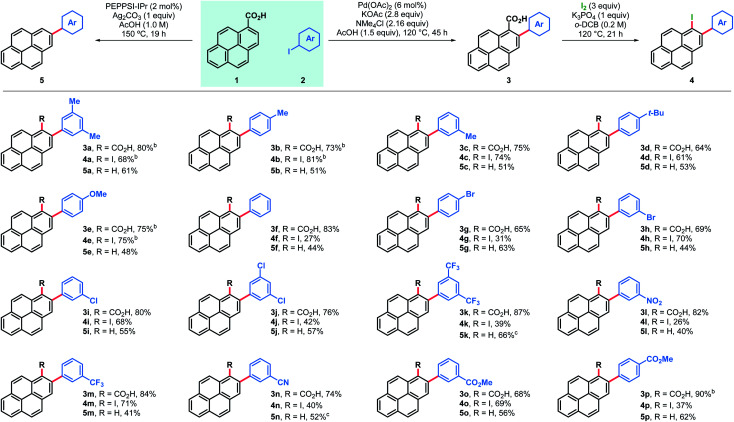

a All yields are isolated.

b Reaction run at 1.00–3.00 mmol scale; see ESI for further details.

c Conditions: **3** (20 mg), Ag_2_CO_3_ (1 equiv.), DMSO (0.07 M), 140 °C, 15 h.

Alternatively, arylation products **3** could be obtained in good yields using aryl bromide electrophiles under ruthenium catalysis (Table 2).^21^ This system also offered an opportunity to introduce both electron-rich and electron-deficient heteroaromatic substituents in the pyrene ring and obtain heteroaryl-substituted pyrene carboxylic acids in moderate to good yields, while the same heteroaryl electrophiles failed to engage in both the Pd catalyzed decarboxylative and non-decarboxylative cross-coupling reactions.

**Table 2:** Use of aryl bromides and heteroarylation of pyrene-1-carboxylic acid

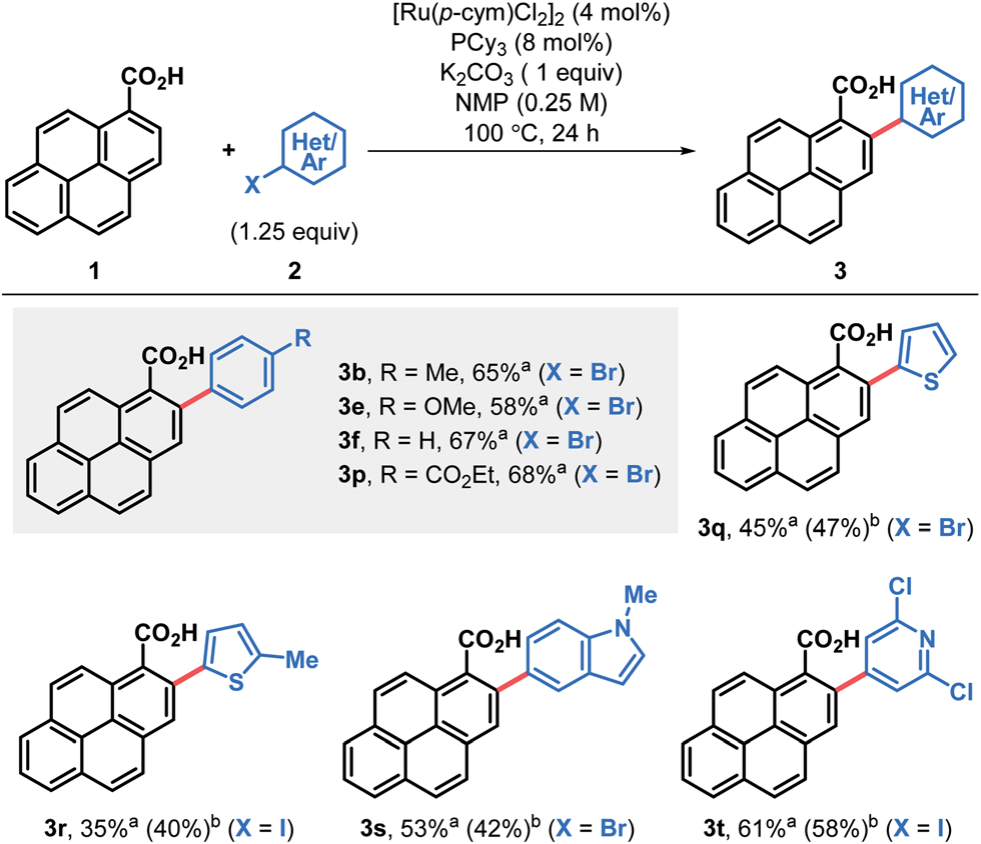

a NMR yield determined using nitromethane as internal standard.

b Isolated yield.

Arylated iodopyrenes **4** offer a platform for further derivatisation using well stablished cross-coupling reactions (Table 3). This was first validated with the preparation of a series of alkynylated derivatives **6** using primary aryl alkynes and trimethylsilylacetylene in Sonogashira couplings,^18^ which gave the desired products in good yields with both trimethylacetylene and a range of arylalkynes. Alkynylated derivatives have been previously used in the design and synthesis of extended polyaromatic hydrocarbons, such as graphene nanoribbons.^22^ Alternatively, 1,2-diarylated pyrenes **7** were obtained in good yields after coupling the same iodopyrenes **4** with arylboronic acids using a Suzuki coupling. Furthermore, these products can be useful in the synthesis of even more substituted pyrene compounds. For instance, C–H borylation was used to obtain bisarylated pyreneboronic ester **8** in good yield and exquisite regioselectivity, offering a route to 1,2,7-trisubstituted pyrenes. Pyrene-2-boronic esters have previously been reported to be versatile intermediates for extensive further manipulation.^10*b*^

**Table 3:** Derivatisation of arylated iodopyrenes^a^

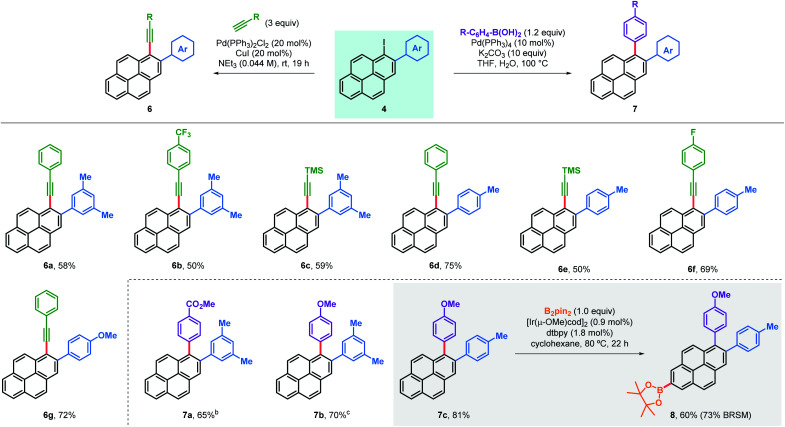

a All yields are isolated.

b K_2_CO_3_ replaced by Na_2_CO_3_ (7.2 equiv.).

c EtOH added as co-solvent.

Next, we embarked in the derivatisation of carboxylic acids **3** to obtain positively charged pyrene derivatives that can be used in the production of sable water-based graphene suspensions (Table 4). To that end, carboxylic acids **3b**, **3p** and **3k** were reduced to benzylic alcohols **9**, brominated and subsequently transformed into the corresponding trimethylammonium bromide salts **10a**, **10b** and **10d**, respectively. Alternatively, **3p** was fully reduced into the corresponding diol after reaction of both the carboxylic acid and ester groups, allowing the formation of the corresponding dicationic salt **10c**.^18^ For comparison, non-arylated pyrene cation **11** was also prepared and used as control.^18^

**Table 4:** Synthesis of arylated pyrene cations^a^

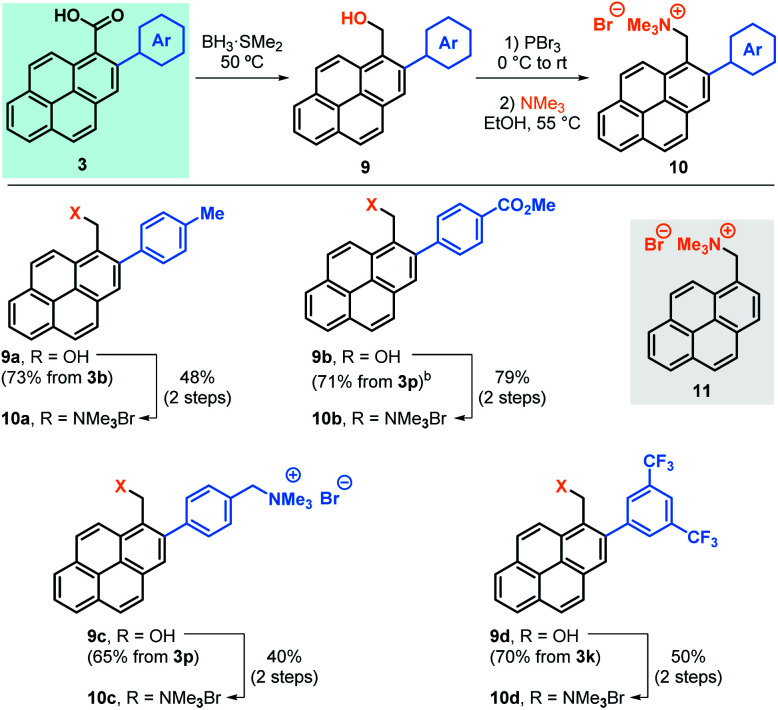

a All yields are isolated.

b In this case, the carboxylic acid was selectively reduced in a two-step procedure: (a) SOCl_2_, 70 °C; (b) NaBH_4_, THF, 50 °C.

It is very interesting to assess the exfoliation efficiency of the set of aryl-substituted pyrenes **10a–c** and compare them with unsubstituted pyrene cation **11**. Pyrene derivatives have been widely used as exfoliating agents; however, the underlying nature of the interaction of the pyrene derivatives with graphene sheets during exfoliation is not completely understood. Thus, it is important to gain more insight on how to design a pyrene-based exfoliating agent that would allow the highest exfoliation efficiency, both in terms of concentration and relative percentage of single-layer graphene in the dispersion.

Pyrene cations **10a–c** and **11** were then tested for their ability to exfoliate graphite in water to produce stable aqueous graphene dispersions. Higher concentrations of graphene were obtained when using arylated pyrene cations **10**, according to UV-Vis spectroscopy analysis, as opposed to non-arylated analogue **11** (Table 5).^18^ Compounds **10a** and **10c** gave very similar concentrations of exfoliated graphene despite the extra ammonium group in **10c**. However, the ester-bearing cation **10b** gave twice as concentrated graphene dispersions, presumably thanks to the higher electron deficiency of the ester-substituted pyrene derivative. This trend was further confirmed by **10d**, another electron-poor analogue, which gave a concentration of aqueous graphene similar to that of **10b**. The concentrations obtained with **10a–d** were of the same order of magnitude than the one obtained with negatively charged sodium 1-pyrenesulfonate (**PS1**, see Fig. 1) under the same exfoliation conditions (110.9 μg mL^−1^).^18^**PS1** is one of the most commonly used negatively charged organic surfactants for aqueous graphene exfoliation and has found application in different graphene-based technologies.^2*f*,*g*,6^

**Table 5:** Aqueous liquid phase exfoliation of graphite with pyrene cations

Compound	Graphene concentration^a^ (μg mL^−1^)	Zeta-potential (mV)
**10a**	88.8	40.9
**10b**	170.3	37.0
**10c**	79.6	46.0
**10d**	143.3	39.0
**11**	18.1	40.5

a Determined by UV-Vis spectroscopy.

**Fig. 2:**
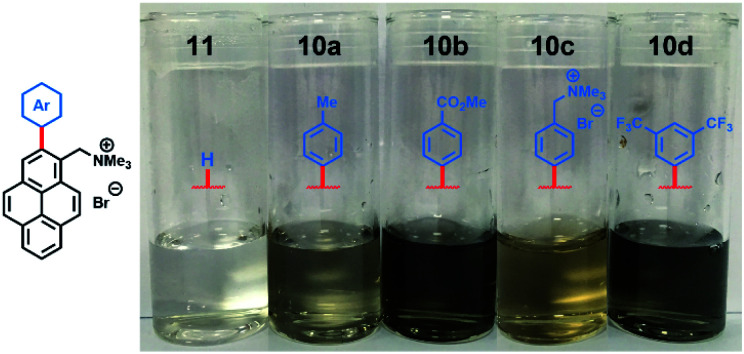
Pictures of diluted (×10) aqueous dispersions of exfoliated graphene with pyrene cations **10a–d** and **11**.

Aqueous suspensions of exfoliated graphene were diluted (×10) for visual inspection. The pictures in Fig. 2 clearly illustrate the stark difference in graphene concentration between suspensions prepared with arylated pyrene cations **10a–d** and non-arylated pyrene cation **11**. As expected, the more concentrated suspensions, prepared with electron-poor compounds **10b** and **10d**, were darker than the rest.

**Fig. 3:**
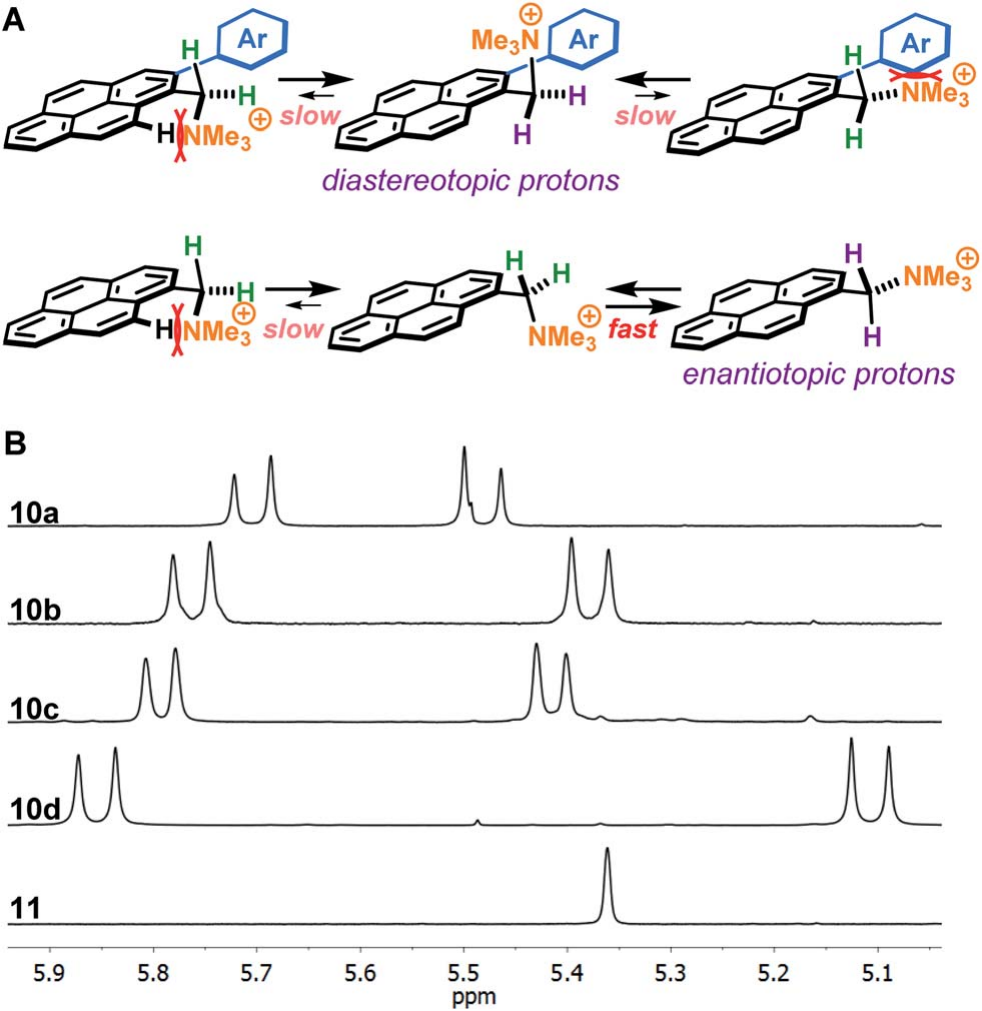
Differences in benzylic protons between pyrene cations in this study. (A) Rotamers along the C sp^2^–C sp^3^ bond of arylated (**10a–d**) and non-arylated (**11**) pyrene cations. (B) ^1^H-NMR signals of benzylic protons in compounds **10a–d** and **11** in methanol-d_4_. The diastereotopic benzylic protons of arylated compounds **10a–d** indicate that the ammonium group is locked pointing away off the pyrene ring plane. The second set of benzylic protons of compound **10c** appear outside the shown range. See ESI† for full spectra.

A plausible explanation for the results obtained with 2-arylated pyrene cations **10** is that steric clashes result in the benzylic ammonium group to be oriented perpendicular to the plane of the pyrene ring, locking the molecule in a favourable conformation (Fig. 3A). Due to the increased sterics, there is restricted rotation around the C sp^2^–C sp^3^ bond leading to the emergence of axial chirality. This hypothesis is supported by ^1^H-NMR analysis, which shows that the benzylic protons in the 2-arylpyrenes **10** are diastereotopic, suggesting that the ammonium group is positioned preferably perpendicular to the pyrene ring plane, thus favouring π-stacking to graphene with one face of the molecule and enhancing interaction with water on the side where the ammonium group points away from the graphene flake (Fig. 3B). This is in sharp contrast with the benzylic protons in non-arylated compound **11**, which appear as a singlet in the corresponding ^1^H-NMR, consistent with free rotation along the C sp^2^–C sp^3^ bond. Moreover, complexation of aromatic molecules bearing bi-aryl moieties with graphene has been reported to increase planarity of those molecules, leading to increased π-stacking interactions.^23^

**Fig. 4:**
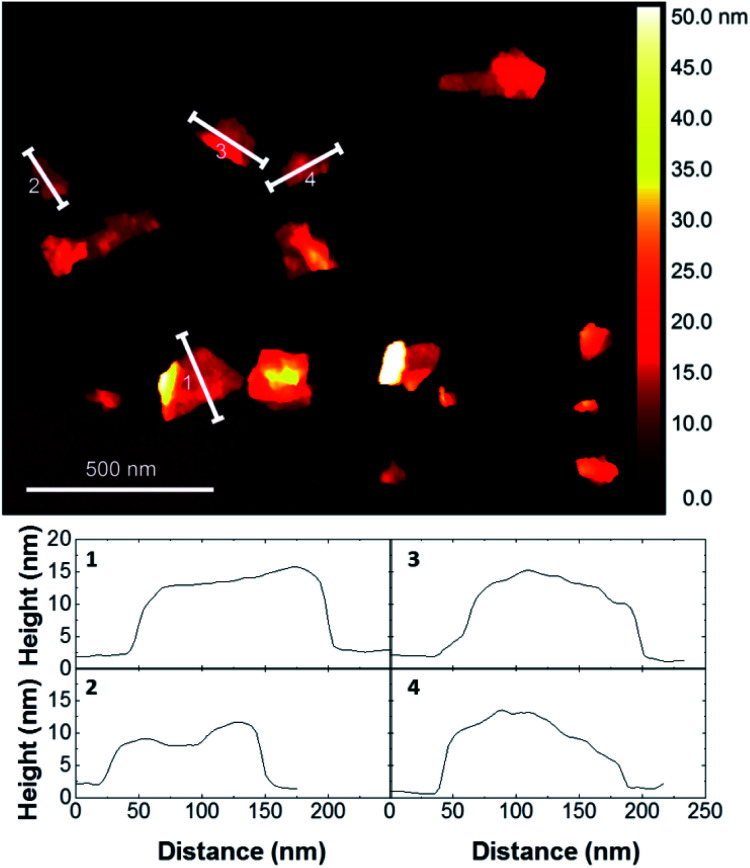
Representative AFM image of graphene flakes prepared with pyrene cation **10b** and cross-section analyses of several flakes. See ESI† for AFM images of graphene flakes exfoliated with all pyrene cations.

The exfoliated graphene nanosheets were characterised by atomic force microscopy (AFM) for analysis of lateral size. Fig. 4 shows that most of the nanosheets have a lateral size of few hundreds of nanometers. Due to the adsorbed pyrene derivatives, the number of layers of nanosheets cannot be reliably estimated from AFM. Thus, we performed Raman analysis using a protocol that allows to qualitatively determining the number of single-layers, few-layers (less than 10 layers) and residual graphite (>10 layers) by analyzing the symmetry of the Raman 2D peak of several flakes.^24^ The Raman results showed that all the graphene dispersions prepared in this study consist of mostly single-layer and few-layer graphene nanosheets, showing effective exfoliation of graphite using pyrene derivatives.^18^ The stability of the exfoliated graphene aqueous dispersion was investigated by zeta-potential measurements, which are typically used as a measure of stability of colloidal suspensions. All graphene dispersions showed a zeta-potential absolute value higher than 30 mV (Table 5), confirming electrostatic stabilization. This was also supported by visual inspection as no noticeable sedimentation was observed over several months of storage at room temperature.

## Conclusions

In conclusion, we have developed a versatile strategy for the synthesis of 2-arylated pyrenes, including 1,2-disubstituted derivatives with several functionalities easily introduced in position 1, such as carboxylic acid, iodide, alkynyl, aryl and alkyl (benzylic alcohols, bromides and ammonium salts). This approach also offers a one-step alternative to the synthesis of 2-aryl pyrenes and opens new possibilities for the design and synthesis of unprecedented functional pyrene-based materials, previously unexplored due to its synthetic inaccessibility. Importantly, the cationic arylated pyrene ammonium salts accessible by this method can be used to obtain stable aqueous graphene suspensions by liquid phase exfoliation of graphite, composed mostly of single- and few-layer graphene flakes, and of concentrations comparable to those achieved with state-of-the-art anionic pyrene exfoliators.

## Conflicts of interest

The authors declare no conflict of interest.

## Supplementary Material

SC-011-C9SC05101E-s001
