# Supplementary material for: Palladium catalysed C–H arylation of pyrenes: access to a new class of exfoliating agents for water-based graphene dispersions
Source: Chem Sci. 2020 Jan 28;11(9):2472–8. doi: 10.1039/c9sc05101e (PMC8157272; doi:10.1039/c9sc05101e)
Supplement: SC-011-C9SC05101E-s001 [file SC-011-C9SC05101E-s001.pdf]

Electronic Supplementary Material (ESI) for Chemical Science.
This journal is © The Royal Society of Chemistry 2020

Palladium Catalysed C–H Arylation of Pyrenes: Access to a New Class of Exfoliating Agents for Water-Based Graphene Dispersions

Xavier Just-Baringo,[a] Yuyoung Shin,[a] Adyasha Panigrahi,[a] Marco Zarattini,[a] Vaiva Nagyte,[a] Ling Zhao,[a] Kostas Kostarelos,[b] Cinzia Casiraghi,*[,a] Igor Larrosa*[,a]

[a]Department of Chemistry, University of Manchester, Oxford Road, Manchester, M13 9PL (UK)

[b]Nanomedicine Lab, Faculty of Biology, Medicine & Health, AV Hill Building, University of Manchester, Oxford Road, Manchester, M13 9PL (UK)

e-mails: cinzia.casiraghi@manchester.ac.uk / igor.larrosa@manchester.ac.uk

Table of Contents

1. General Information S1
2. Optimisation Tables S2
3. Synthesis and Characterisation S5
4. NMR Spectra S40
5. Liquid Phase Exfoliation S123
6. AFM Analysis S124
7. Raman Analysis S125
8. Zeta-Potential Measurements S126
9. UV-Vis Spectroscopy S126

1. General Information

Tetrahydrofuran (THF) and toluene were dried using a PureSolv solvent purification system. All other solvents and reagents used were purchased from commercial suppliers and used without further purification. ^{1}H-NMR spectra were obtained at room temperature on a Bruker 400 MHz or 500 MHz spectrometer. ^{13}C-NMR spectra were obtained at 100 or 125 MHz, respectively. ^{19}F-NMR spectra were obtained at 376 or 471 MHz, respectively. All NMR spectra were processed using *MestReNova* NMR software. Chemical shifts are reported in parts per million (ppm) and coupling constants (*J*) are reported in Hz. Splitting patterns are reported as follows: singlet (s), doublet (d), triplet (t), quadruplet (q), quintuplet (quint), doublet of doublets (dd), doublet of doublets of doublets (ddd), multiplet (m), etc. NMR signals were assigned using the appropriate 2D NMR experiments (*i.e.* HSQC and HMBC) when necessary. TLC analysis was carried out on aluminium sheets coated with silica gel and visualised using potassium permanganate solution and/or UV light. Infra-red spectra were recorded as evaporated films or neat using FT/IR spectrometers. Melting points were measured on solids as obtained after chromatography. Mass spectra were obtained using positive or negative electrospray (ESI), atmospheric pressure chemical ionization (APCI) or atmospheric solids analysis probe (ASAP).

For details on Liquid Fase Exfoliation and the subsequent analyses (AFM, Raman, Zeta-Potential and UV-Vis), see the corresponding sections.

2. Optimisation Tables

Table S1. Non-decarboxylative C–H *ortho*-arylation.[a]

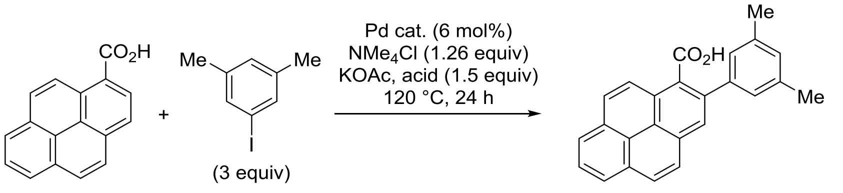

1 **2a** **3a**

entry	Pd cat. (mol%)	acid (equiv)	3a - NMR yield (isolated yield)	1 (NMR RSM)
**1**	$Pd(OAc)_2$	AcOH (1.5)	82	17
**2**	PEPPSI-IPr	AcOH (1.5)	84	16
**3**	$Pd(OAc)_2$	AcOH (1 M)	9	26
**4**	PEPPSI-IPr	AcOH (1 M)	-	31
**5**	$Pd(OAc)_2$	TFA (1.5)	28	29
**6**[b]	$Pd(OAc)_2$	AcOH (1.5)	89	-
**7**[b,c]	$Pd(OAc)_2$	AcOH (1.5)	(80)	N/A

[a]Reactions run at 0.203 mmol scale; NMR yields determined with $MeNO_2$ as internal standard. [b]Extra NMe_4Cl (0.80 mmol) and KOAc (0.80 mmol) added after 24 h and stirred for another 21 h at 120 ˚C. [c]Reaction run at 1.00 mmol scale.

Table S2. Iododecarboxylation.[a]

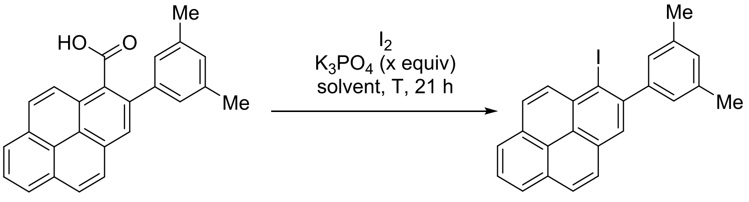

3a **4a**

entry	Solvent	T (˚C)	I_2 (equiv)	K_3PO_4 (equiv)	4a - NMR yield (isolated yield)
**1**	MeCN (0.2 M)	100	4	1	<34
**2**	1,4-dioxane (1.0 M)	150	4	1	-
**3**	*o*-DCB (1.0 M)	150	4	1	~50
**4**	*o*-DCB (1.0 M)	150	3	1	~50
**5**	*o*-DCB (1.0 M)	150	2	1	~50
**6**	*o*-DCB (1.0 M)	120	3	1	49
**7**	*o*-DCB (1.0 M)	100	3	1	43
**8**	*o*-DCB (1.0 M)	120	2	1	47

**9**	*o*-DCB (1.0 M)	100	2	1	46
**10**	*o*-DCB (1.0 M)	120	3	1	56
**11**	*o*-DCB (1.0 M)	120	3	2	55
**12**	*o*-DCB (0.5 M)	120	3	1	67
**13**	*o*-DCB (0.5 M)	120	6	1	54
**14**	*o*-DCB (0.5 M)	120	6	2	51
**15**	*o*-DCB (0.2 M)	120	3	1	64
**16**	*o*-DCB (0.1 M)	120	3	1	52
**17**	*o*-DCB (0.2 M)	120	3	1	69 (65)
**18**	*o*-DCB (0.5 M)	120	3	1	65
**19**	*o*-DCB (0.2 M)	150	3	1	57
**20**	*o*-DCB (0.5 M)	150	3	1	62
**21**	*o*-DCB (0.2 M)	170	3	1	47
**22**	*o*-DCB (0.5 M)	170	3	1	59

[a]Reactions run at 0.1 mmol scale. NMR yields determined with $MeNO_2$ as internal standard.

Table S3. Decarboxylative C–H *ortho*-arylation.[a]

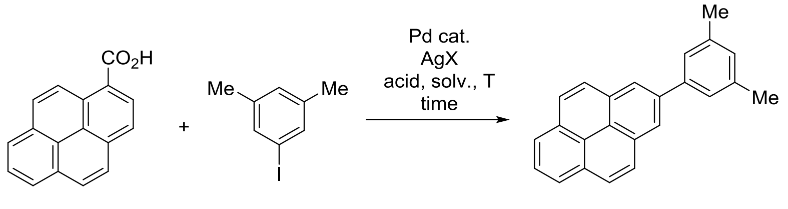

1 **2a** **5a**

entry	2a (equiv)	Pd cat. (mol%)	AgX (equiv)	acid (equiv)	T (°C)	time	5a - NMR yield (isolated yield)
**1**	3	$Pd(OAc)_2$ (2)	Ag_2CO_3 (1)	TFA (3.5)	130	3 days	-
**2**	10	$Pd(OAc)_2$ (2)	Ag_2CO_3 (1)	TFA (3.5)	130	3 days	-
**3**	3	$Pd(OAc)_2$ (2)	Ag_2CO_3 (1)	AcOH (3.5)	130	19 h	30
**4**	3	$Pd(OAc)_2$ (2)	Ag_2CO_3 (1)	AcOH (7.0)	130	19 h	50
**5**	3	$Pd(OAc)_2$ (2)	AgOAc (2)	AcOH (3.5)	130	19 h	58
**6**[b]	3	$Pd(OAc)_2$ (2)	Ag_2CO_3 (1)	AcOH (3.5)	130	19 h	<5
**7**	3	$Pd(OAc)_2$ (2)	AgOAc (2)	AcOH (7.0)	130	19 h	50
**8**	3	$Pd(OAc)_2$ (2)	AgOAc (2)	AcOH (14.0)	130	19 h	44
**9**	3	$Pd(OAc)_2$ (2)	Ag_2CO_3 (1)	AcOH (3.5)	130	3 days	61
**10**	10	$Pd(OAc)_2$ (2)	Ag_2CO_3 (1)	AcOH (3.5)	130	3 days	54
**11**	3	$Pd(OAc)_2$ (2)	AgOAc (2)	AcOH (3.5)	130	3 days	57
**12**	3	PEPPSI-IPr (2)	AgOAc (2)	AcOH (3.5)	130	17 h	36
**13**	3	PEPPSI-IPr (2)	Ag_2CO_3 (1)	AcOH (3.5)	150	19 h	57
**14**	3	PEPPSI-IPr (2)	Ag_2CO_3 (0.5)	AcOH (3.5)	150	19 h	32
**15**	3	PEPPSI-IPr (2)	AgOAc (2)	AcOH (3.5)	150	19 h	63

**16**	3	PEPPSI-IPr (2)	AgOAc (1)	AcOH (3.5)	150	19 h	32
**17**	3	PEPPSI-IPr (2)	Ag_2CO_3 (1)	AcOH (1.0 M)	150	19 h	69 (61)
**18**[c]	3	PEPPSI-IPr (2)	Ag_2CO_3 (1)	AcOH (1.0 M)	150	19 h	50
**19**	3	PEPPSI-IPr (2)	Ag_2CO_3 (0.5)	AcOH (1.0 M)	150	19 h	24
**20**[c]	3	PEPPSI-IPr (2)	Ag_2CO_3 (0.5)	AcOH (1.0 M)	150	19 h	11
**21**[d]	3	PEPPSI-IPr (2)	Ag_2CO_3 (1)	AcOH (1.0 M)	150	19 h	59
**22**	3	$Pd(OAc)_2$ (2)	Ag_2CO_3 (1)	AcOH (1.0 M)	150	19 h	53
**23**	3	$Pd(dppf)Cl_2$ (2)	Ag_2CO_3 (1)	AcOH (1.0 M)	150	19 h	38
**24**	3	PEPPSI-IPent (2)	Ag_2CO_3 (1)	AcOH (1.0 M)	150	19 h	68
**25**	3	PEPPSI-IPr (2)	AgOAc (2)	AcOH (1.0 M)	150	19 h	66
**26**	3	PEPPSI-IPr (2)	AgO_2CCF_3 (2)	AcOH (1.0 M)	150	19 h	32
**27**	3	PEPPSI-IPr (2)	Ag_2O (1)	AcOH (1.0 M)	150	19 h	62
**28**	3	PEPPSI-IPr (2)	$AgBF_4$ (2)	AcOH (1.0 M)	150	19 h	N.P.
**29**	3	PEPPSI-IPr (2)	Ag_2CO_3 (1)	AcOH (1.0 M)	150	3 days	67
**30**	3	PEPPSI-IPr (2)	AgOAc (2)	AcOH (1.0 M)	150	3 days	66
**31**	3	PEPPSI-IPr (2)	Ag_2O (1)	AcOH (1.0 M)	150	3 days	64

[a]Reactions run at 0.203 mmol scale; NMR yields determined with $MeNO_2$ as internal standard. [b]Toluene (0.4 mL) added as solvent. [c]K_2CO_3 (14 mg, 0.102 mmol) added. [d]Extra Ag_2CO_3 (1 equiv) in DMSO (1.0 mL) was added after 19 h and the mixture was stirred at 170 ˚C for 3.5 h.

Table S4. Sonogashira coupling.[a]

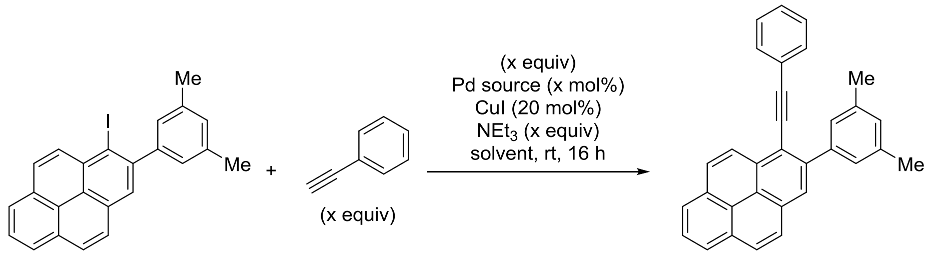

4a **6a**

Entry	Phenylacetylene (equiv)	Pd cat. (mol%)	NEt_3	solvent	6a - NMR yield (isolated yield)	4a (NMR RSM)
**1**	1.1	$PdCl_2(PPh_3)_2$ (20 mol%)	0.044 M	-	36	-
**2**	1.0	$Pd(PPh_3)_4$ (5 mol%)	1.5 equiv	THF (0.1 M)	15	64
**3**	3.0	$Pd(PPh_3)_4$ (5 mol%)	1.5 equiv	THF (0.1 M)	-	81
**4**	3.0	$PdCl_2(PPh_3)_2$ (20 mol%)	0.044 M	-	75 (58)	-
**5**	3.0	$Pd(PPh_3)_4$ (20 mol%)	0.044 M	-	72	-
**6**	3.0	$PdCl_2(PPh_3)_2$ (20 mol%)	1.5 equiv	THF (0.1 M)	15	56

[a]Reactions run at 0.0347 mmol (15 mg) scale; NMR yield determined with $MeNO_2$ as internal standard.

3. Synthesis and characterisation

General Procedure A: Palladium catalysed non-decarboxylative *ortho*-arylation of pyrene-1-carboxylic acid

2-(3,5-Dimethylphenyl)pyrene-1-carboxylic acid (3a)

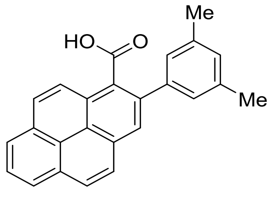

A microwave vial fitted with a Teflon-coated stirring bar was loaded with pyrene-1-carboxylic acid (246 mg, 1.00 mmol), 5-iodo-ortho-xylene (433 μL, 3.00 mmol), $Pd(OAc)_2$ (13.5 mg, 0.06 mmol), KOAc (196 mg, 2.00 mmol), NMe_4Cl (138 mg, 1.26 mmol) and acetic acid (87 μL, 1.50 mmol). The vial was sealed under air and the mixture was stirred at 120 ˚C. After 24 h the mixture was allowed to reach room temperature, the vial was opened and loaded with more KOAc (79 mg, 0.80 mmol) and NMe_4Cl (88 mg, 0.80 mmol) and the vial was resealed and stirred at 120 ˚C for 21 h. After cooling to room temperature, the mixture was partitioned between 2 M aqueous HCl (15 mL) and EtOAc (20 mL) and the organic fraction was filtered through a short plug of Celite. After removing volatiles under vacuum the crude product was purified by silica flash column chromatography (hexane/EtOAc, 100:0 to 50:50) to yield the title product as a pale orange solid (282 mg, 80%), mp (EtOAc) 232-235 ºC. ^{1}H-NMR (400 MHz, DMSO-d_6) δ 2.38 (s, C*H*$_3$, 6 H), 7.11 (s, Ar*H*, 1 H), 7.28 (s, Ar*H*, 2 H), 8.09-8.21 (m, Ar*H*, 2 H), 8.23-8.42 (m, Ar*H*, 6 H), 13.57 (bs, CO$_2$*H*, 1 H) ppm; ^{13}C-NMR (125 MHz, DMSO-d_6) δ 21.1 (C*H*$_3$), 122.5 (Ar*C*), 123.4 (Ar*C*), 124.1 (Ar*C*H), 125.8 (Ar*C*H), 126.0 (Ar*C*H), 126.1 (Ar*C*H), 126.7 (Ar*C*H), 126.8 (Ar*C*), 126.9 (Ar*C*H), 127.2 (Ar*C*H), 128.5 (Ar*C*H), 128.9 (Ar*C*H), 129.0 (Ar*C*H), 129.4 (Ar*C*), 130.1 (Ar*C*), 130.7 (Ar*C*), 131.0 (Ar*C*), 136.6 (Ar*C*), 137.4 (Ar*C*), 140.6 (Ar*C*), 170.6 (Ar*C*O$_2$H) ppm; IR ν_{max} (neat/cm^{-1}): 3035, 2916, 1690, 1597, 1407, 1275; HRMS calcd for $C_{25}H_{19}O_2$ $[M+H]^+$: 351.1380, found 351.1368.

2-(4-Methylphenyl)pyrene-1-carboxylic acid (3b)

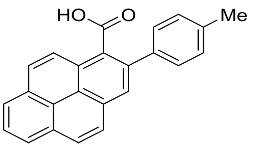

Prepared according to general procedure A using pyrene-1-carboxylic acid (246 mg, 1.00 mmol) and 4-iodotoluene (654 mg, 3.00 mmol). The crude product was purified by silica flash column chromatography (hexane/EtOAc, 100:0 to 50:50) to yield the title product as a brown solid (252 mg, 73%), mp (EtOAc) 254-257 ºC. ^{1}H-NMR (500 MHz, DMSO-d_6) δ 2.41 (s, C*H*$_3$, 3 H), 7.35 (d, *J* = 8.0 Hz, Ar*H*, 2 H), 7.56 (d, *J* = 8.0 Hz, Ar*H*, 2 H), 8.14 (t, *J* = 7.8 Hz, Ar*H*, 1 H), 8.18 (d, *J* = 9.0 Hz, Ar*H*, 1 H), 8.26 (d, *J* = 8.5 Hz, Ar*H*, 1 H), 8.29 (d, *J* = 8.5 Hz, Ar*H*, 1 H), 8.31-8.35 (m, Ar*H*, 2 H), 8.36-8.40 (m, Ar*H*, 2 H), 13.53 (bs, CO$_2$*H*, 1 H) ppm; ^{13}C-NMR (125 MHz, DMSO-d_6) δ 20.8 (*C*H$_3$), 122.4 (Ar*C*), 123.4 (Ar*C*), 124.1 (Ar*C*H), 125.8 (Ar*C*H), 126.0 (Ar*C*H), 126.1 (Ar*C*H), 126.7 (Ar*C*H), 126.9 (Ar*C*), 127.2 (Ar*C*H), 128.5 (Ar*C*H), 128.9 (Ar*C*H), 128.9 (Ar*C*H), 129.1 (Ar*C*H), 129.6 (Ar*C*), 130.1 (Ar*C*), 130.7 (Ar*C*), 131.0 (Ar*C*), 136.3 (Ar*C*), 136.9 (Ar*C*), 137.8 (Ar*C*), 170.6 (*C*O$_2$H) ppm; IR ν_{max} (neat/cm^{-1}): 3037, 1682, 1596, 1512, 1404, 1267; HRMS calcd for $C_{24}H_{15}O_2$ $[M–H]^-$: 335.1078, found 335.1070.

2-(3-Tolyl)pyrene-1-carboxylic acid (3c)

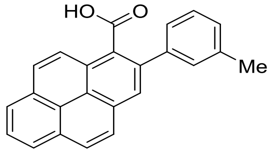

Prepared according to general procedure A using pyrene-1-carboxylic acid (100 mg, 0.406 mmol) and 3-iodotoluene (157 μL, 1.22 mmol). The crude product was purified by silica flash column chromatography (hexane/EtOAc, 100:0 to 60:40) to yield the title product as an orange solid (102 mg, 75%), mp (CH_2Cl_2) 188-191 °C. ^{1}H-NMR (400 MHz, DMSO-d_6) δ 2.43 (s, C*H*$_3$, 3 H), 7.29 (d, *J* = 7.2 Hz, Ar*H*, 1 H), 7.39-7.53 (m, Ar*H*, 3 H), 8.14 (t, *J* = 7.6 Hz, Ar*H*, 1 H), 8.19 (d, *J* = 9.2 Hz, Ar*H*, 1 H), 8.27 (d, *J* = 9.2 Hz, Ar*H*, 1 H), 8.30 (d, *J* = 9.2 Hz, Ar*H*, 1 H), 8.32-8.41 (m, Ar*H*, 4 H), 13.57 (s, CO_2*H*, 1 H) ppm; ^{13}C-NMR (125 MHz, DMSO-d_6) δ 21.2 (*C*H_3), 122.5 (Ar*C*), 123.4 (Ar*C*), 124.1 (Ar*C*H), 125.8 (Ar*C*H), 126.1 (Ar*C*H), 126.1 (Ar*C*H), 126.1 (Ar*C*H), 126.7 (Ar*C*H), 126.9 (Ar*C*), 127.2 (Ar*C*H), 128.3 (Ar*C*H), 128.4 (Ar*C*H), 128.6 (Ar*C*H), 129.0 (Ar*C*H), 129.7 (Ar*C*H), 130.1 (Ar*C*), 130.7 (Ar*C*), 131.0 (Ar*C*), 136.5 (Ar*C*), 137.6 (Ar*C*), 140.7 (Ar*C*), 170.5 (Ar*C*O_2H) ppm, one Ar*C* was not observed; IR ν_{max} (neat/cm^{-1}): 2916, 1682, 1597, 1411, 1279; HRMS calcd for $C_{24}H_{15}O_2$ [M–H]$^-$: 335.1078, found 335.1080.

2-(4-*tert*-Butylphenyl)pyrene-1-carboxylic acid (3d)

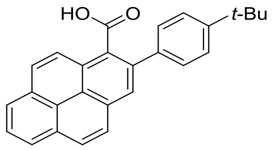

Prepared according to general procedure A using pyrene-1-carboxylic acid (100 mg, 0.406 mmol) and 1-(*tert*-butyl)-1-iodobenzene (216 μL, 1.22 mmol). The crude product was purified by silica flash column chromatography (hexane/EtOAc, 100:0 to 50:50) to yield the title product as a pale orange solid (99 mg, 64%), mp (CH_2Cl_2) 210-213 °C. ^{1}H-NMR (500 MHz, DMSO-d_6) δ 1.37 (s, C(C*H*$_3$)$_3$, 9 H), 7.57 (d, *J* = 8.3 Hz, Ar*H*, 2 H), 7.62 (d, *J* = 8.3 Hz, Ar*H*, 2 H), 8.14 (t, *J* = 7.8 Hz, Ar*H*, 1 H), 8.18 (d, *J* = 9.5 Hz, Ar*H*, 1 H), 8.25 (d, *J* = 9.0 Hz, Ar*H*, 1 H), 8.29 (d, *J* = 9.0 Hz, Ar*H*, 1 H), 8.31-8.36 (m, Ar*H*, 2 H), 8.36-8.40 (m, Ar*H*, 2 H), 13.56 (bs, CO_2*H*, 1 H) ppm; ^{13}C-NMR (125 MHz, DMSO-d_6) δ 31.2 (C(*C*H_3)$_3$), 34.4 (*C*(CH_3)$_3$), 122.4 (Ar*C*), 123.4 (Ar*C*), 124.1 (Ar*C*H), 125.4 (Ar*C*H), 125.8 (Ar*C*H), 126.1 (Ar*C*H), 126.1 (Ar*C*H), 126.7 (Ar*C*H), 126.8 (Ar*C*), 127.2 (Ar*C*H), 128.5 (Ar*C*H), 128.7 (Ar*C*H), 129.0 (Ar*C*H), 129.5 (Ar*C*), 130.1 (Ar*C*), 130.7 (Ar*C*), 131.0 (Ar*C*), 136.2 (Ar*C*), 137.8 (Ar*C*), 150.0 (Ar*C*), 170.6 (Ar*C*O_2H) ppm; IR ν_{max} (neat/cm^{-1}): 2959, 1689, 1402, 1269, 841, 829; HRMS calcd for $C_{27}H_{21}O_2$ [M–H]$^-$: 377.1547, found 377.1546.

2-(4-Methoxyphenyl)pyrene-1-carboxylic acid (3e)

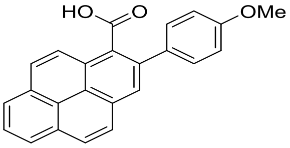

Prepared according to general procedure A, but using a tick glass wall pressure tube as reaction vessel with pyrene-1-carboxylic acid (739 mg, 3.00 mmol) and 4-iodoanisole (2.11 g, 9.00 mmol). The crude product was purified by silica flash column chromatography (hexane/EtOAc, 100:0 to 30:70) to yield the title product as an orange solid (790 mg, 75%), mp (CH_2Cl_2) 180-183 ºC. ^{1}H-NMR (500 MHz, DMSO-d_6) δ 3.85 (s, OC*H*$_3$, 3 H), 7.11 (d, *J* = 8.0 Hz, Ar*H*, 2 H), 7.61 (d, *J* = 8.0 Hz, Ar*H*, 2 H), 8.13 (t, *J* = 7.5 Hz, Ar*H*, 1 H), 8.18 (d, *J* = 9.5 Hz, Ar*H*, 1 H), 8.25 (d, *J* = 9.3 Hz, Ar*H*, 1 H), 8.28 (d, *J* = 9.3 Hz, Ar*H*, 1 H), 8.30-8.35 (m, Ar*H*, 2 H), 8.35-8.39 (m, Ar*H*, 2 H), 13.53 (bs, CO_2*H*, 1 H) ppm; ^{13}C-NMR (125 MHz, DMSO-d_6) δ 55.2 (O*C*H_3), 114.0 (Ar*C*H), 122.3 (Ar*C*), 123.4 (Ar*C*), 124.1 (Ar*C*H), 125.8 (Ar*C*H), 126.0 (Ar*C*H), 126.1 (Ar*C*H), 126.7 (Ar*C*H), 126.9 (Ar*C*), 127.2 (Ar*C*H), 128.5 (Ar*C*H), 128.9 (Ar*C*H), 129.5 (Ar*C*), 130.0 (Ar*C*), 130.2 (Ar*C*H), 103.7 (Ar*C*), 131.0 (Ar*C*), 132.9 (Ar*C*), 136.0 (Ar*C*), 159.0 (Ar*C*), 170.7 (Ar*C*O_2H) ppm; IR ν_{max} (neat/cm^{-1}): 2960, 1681, 1512, 1248, 1174, 1028, 827; HRMS calcd for $C_{24}H_{15}O_3$ [M–H]$^-$: 351.1027, found 351.1028.

2-Phenylpyrene-1-carboxylic acid (3f)

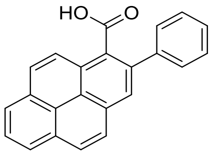

Prepared according to general procedure A using pyrene-1-carboxylic acid (100 mg, 0.406 mmol) and iodobenzene (137 μL, 1.22 mmol). The crude product was purified by silica flash column chromatography (hexane/EtOAc, 100:0 to 50:50) to yield the title product as a brown solid (109 mg, 83%), mp (CH_2Cl_2) 222-225 ºC. ^{1}H-NMR (500 MHz, DMSO-d_6) δ 7.49 (t, *J* = 7.4 Hz, ArH, 1 H), 7.55 (t, *J* = 7.4 Hz, Ar*H*, 2 H), 7.68 (d, *J* = 7.4 Hz, Ar*H*, 2 H), 8.15 (t, *J* = 7.6 Hz, Ar*H*, 1 H), 8.21 (d, *J* = 9.2 Hz, Ar*H*, 1 H), 8.27 (d, *J* = 9.0 Hz, Ar*H*, 1 H), 8.30 (d, *J* = 9.0 Hz, Ar*H*, 1 H), 8.32-8.43 (m, Ar*H*, 4 H), 13.57 (bs, CO_2*H*, 1 H) ppm; ^{13}C-NMR (125 MHz, DMSO-d_6) δ 122.5 (Ar*C*), 123.4 (Ar*C*), 124.1 (Ar*C*H), 125.9 (Ar*C*H), 126.1 (Ar*C*H), 126.1 (Ar*C*H), 126.8 (Ar*C*H), 126.9 (Ar*C*), 127.2 (Ar*C*H), 127.6 (Ar*C*H), 128.5 (Ar*C*H), 128.6 (Ar*C*H), 129.0 (Ar*C*H), 129.0 (Ar*C*H), 129.5 (Ar*C*), 130.1 (Ar*C*), 130.7 (Ar*C*), 131.0 (Ar*C*), 136.4 (Ar*C*), 140.7 (Ar*C*), 170.5 (Ar*C*O_2H) ppm; IR ν_{max} (neat/cm^{-1}): 1679, 1596, 1406, 1271, 882, 840, 827; HRMS calcd for $C_{23}H_{13}O_2$ [M–H]$^-$: 321.0921, found 321.0913.

2-(4-Bromophenyl)pyrene-1-carboxylic acid (3g)

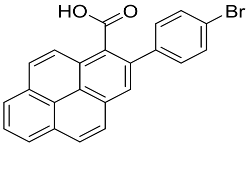

Prepared according to general procedure A using pyrene-1-carboxylic acid (100 mg, 0.406 mmol) and 4-bromoiodobenzene (345 mg, 1.22 mmol). The crude product was purified by silica flash column chromatography (hexane/EtOAc, 100:0 to 50:50) to yield the title product as a pale brown solid (106 mg, 65%), mp (EtOAc) 205-208 ºC. ^{1}H-NMR (500 MHz, DMSO-d$_6$) δ 7.61 (d, *J* = 8.4 Hz, Ar*H*, 2 H), 7.75 (d, *J* = 8.4 Hz, Ar*H*, 2 H), 8.15 (t, *J* = 7.6 Hz, Ar*H*, 1 H), 8.21 (d, *J* = 9.2 Hz, Ar*H*, 1 H), 8.26 (d, *J* = 9.0 Hz, Ar*H*, 1 H), 8.30 (d, *J* = 9.0 Hz, Ar*H*, 1 H), 8.33-8.37 (m, Ar*H*, 2 H), 8.37-8.42 (m, Ar*H*, 2 H), 13.65 (bs, CO_2*H*, 1 H) ppm; ^{13}C-NMR (125 MHz, DMSO-d$_6$) δ 121.8 (Ar*C*), 123.1 (Ar*C*), 123.8 (Ar*C*), 124.5 (Ar*C*H), 126.3 (Ar*C*H), 126.4 (Ar*C*H), 126.7 (Ar*C*H), 127.3 (Ar*C*H), 127.4 (Ar*C*), 127.7 (Ar*C*H), 129.2 (Ar*C*H), 129.6 (Ar*C*H), 130.6 (Ar*C*), 131.2 (Ar*C*), 131.6 (Ar*C*H), 131.9 (Ar*C*H), 135.6 (Ar*C*), 140.4 (Ar*C*), 170.8 (Ar*C*O_2H) ppm, 2 quaternary C were not observed; IR ν_{max} (neat/cm^{-1}): 3038, 1687, 1597, 1486, 1411, 1274, 1009, 880; HRMS calcd for $C_{23}H_{12}O_2Br$ [M–H]$^-$: 399.0026, found 399.0017.

2-(3-Bromophenyl)pyrene-1-carboxylic acid (3h)

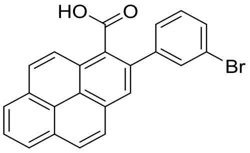

Prepared according to general procedure A using pyrene-1-carboxylic acid (100 mg, 0.406 mmol) and 3-bromoiodobenzene (156 μL, 1.22 mmol). The crude product was purified by silica flash column chromatography (hexane/EtOAc, 100:0 to 70:30) to yield the title product as a pale brown solid (112 mg, 69%), mp (CH_2Cl_2) 217-220 ºC. ^{1}H-NMR (400 MHz, DMSO-d$_6$) δ 7.52 (t, *J* = 8.0 Hz, Ar*H*, 1 H), 7.64-7.73 (m, Ar*H*, 2 H), 7.85 (t, *J* = 1.8 Hz, Ar*H*, 1 H), 8.16 (t, *J* = 7.6 Hz, Ar*H*, 1 H), 8.21 (d, *J* = 9.2 Hz, Ar*H*, 1 H), 8.28 (d, *J* = 8.8 Hz, Ar*H*, 1 H), 8.32 (d, *J* = 8.8 Hz, Ar*H*, 1 H), 8.36 (d, *J* = 9.6 Hz, Ar*H*, 1 H), 8.38-8.43 (m, Ar*H*, 3 H), 13.73 (s, CO_2*H*, 1 H) ppm; ^{13}C-NMR (125 MHz, DMSO-d$_6$) δ 121.7 (Ar*C*), 122.8 (Ar*C*), 123.3 (Ar*C*), 124.1 (Ar*C*H), 126.0 (Ar*C*H), 126.0 (Ar*C*H), 126.3 (Ar*C*H), 126.9 (Ar*C*H), 126.9 (Ar*C*), 127.2 (Ar*C*H), 128.2 (Ar*C*H), 128.8 (Ar*C*H), 129.2 (Ar*C*H), 129.2 (Ar*C*), 130.1 (Ar*C*), 130.5 (Ar*C*H), 130.7 (Ar*C*H), 130.8 (Ar*C*), 131.1 (Ar*C*), 131.5 (Ar*C*H), 134.8 (Ar*C*), 143.1 (Ar*C*), 170.3 (Ar*C*O_2H) ppm; IR ν_{max} (neat/cm^{-1}): 2919, 1662, 1593, 1385, 1350, 1295; HRMS calcd for $C_{23}H_{12}O_2Br$ [M–H]$^-$: 399.0015, found 399.0029.

2-(3-Chlorophenyl)pyrene-1-carboxylic acid (3i)

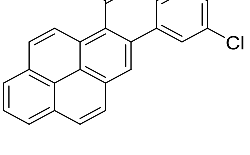

Prepared according to general procedure A using pyrene-1-carboxylic acid (100 mg, 0.406 mmol) and 3-chloroiodobenzene (151 μL, 1.22 mmol). The crude product was purified by silica flash column chromatography (hexane/EtOAc, 100:0 to 50:50) to yield the title product as a pale brown solid (116 mg, 80%), mp (CH_2Cl_2) 216-219 ºC. ^{1}H-NMR (500 MHz, DMSO-d$_6$) δ 7.56 (dt, *J* = 7.5, 1.8 Hz, Ar*H*, 1 H), 7.59 (t, *J* = 7.5 Hz, Ar*H*, 1 H), 7.63 (dt, *J* = 7.5, 1.8 Hz, Ar*H*, 1 H), 7.71 (t, *J* = 1.8 Hz, Ar*H*, 1 H), 8.16 (t, *J* = 7.8 Hz, Ar*H*, 1 H), 8.21 (d, *J* = 9.3 Hz, Ar*H*, 1 H), 8.28 (d, *J* = 9.0 Hz, Ar*H*, 1 H), 8.32

(d, J = 9.0 Hz, ArH, 1 H), 8.36 (d, J = 9.3 Hz, ArH, 1 H), 8.38-8.43 (ArH, 3 H), 13.71 (s, CO_2H, 1 H) ppm; ^{13}C-NMR (125 MHz, DMSO-d_6) δ 122.8 (ArC), 123.3 (ArC), 124.1 (ArCH), 126.0 (ArCH), 126.0 (ArCH), 126.3 (ArCH), 126.9 (ArCH), 127.0 (ArC), 127.2 (ArCH), 127.6 (ArCH), 127.8 (ArCH), 128.7 (ArCH), 128.8 (ArCH), 129.2 (ArCH), 130.1 (ArC), 130.4 (ArCH), 130.8 (ArC), 131.1 (ArC), 133.1 (ArC), 134.8 (ArC), 142.8 (ArC), 170.3 (ArCO$_2$H) ppm, one ArC was not observed; IR ν_{max} (neat/cm^{-1}): 2929, 1652, 1595, 1387, 1307, ; HRMS calcd for $C_{23}H_{12}O_2Cl$ $[M–H]^-$: 355.0531, found 355.0536.

2-(3,5-Dichlorophenyl)pyrene-1-carboxylic acid (3j)

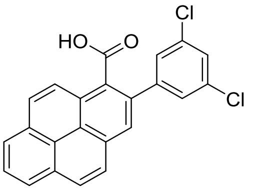

Prepared according to general procedure A using pyrene-1-carboxylic acid (100 mg, 0.406 mmol) and 3,5-dichloroiodobenzene (333 mg, 1.22 mmol). The crude product was purified by silica flash column chromatography (hexane/EtOAc, 100:0 to 60:40) to yield the title product as a brown solid (121 mg, 76%), mp (CH_2Cl_2) 215-218 °C. ^{1}H-NMR (400 MHz, DMSO-d_6) δ 7.70 (d, J = 2.0 Hz, ArH, 2 H), 7.77 (t, J = 2.0 Hz, ArH, 1 H), 8.17 (t, J = 7.6 Hz, ArH, 1 H), 8.23 (d, J = 9.2 Hz, ArH, 1 H), 8.28 (d, J = 9.2 Hz, ArH, 1 H), 8.34 (d, J = 9.2 Hz, ArH, 1 H), 8.37 (d, J = 9.6 Hz, ArH, 1 H), 8.39-8.45 (m, ArH, 3 H), 13.87 (s, CO_2H, 1 H) ppm; ^{13}C-NMR (125 MHz, DMSO-d_6) δ 123.0 (ArC), 123.2 (ArC), 124.0 (ArCH), 126.0 (ArCH), 126.1 (ArCH), 126.4 (ArCH), 127.0 (ArCH), 127.1 (ArC), 127.2 (ArCH), 127.3 (ArCH), 127.7 (ArCH), 129.0 (ArCH), 129.3 (ArCH), 130.2 (ArC), 130.8 (ArC), 131.2 (ArC), 133.5 (ArC), 134.1 (ArC), 144.2 (ArC), 170.2 (ArCO$_2$H) ppm, one ArC was not observed; IR ν_{max} (neat/cm^{-1}): 3038, 1712, 1589, 1559, 1201, 1148; HRMS calcd for $C_{23}H_{11}O_2Cl_2$ $[M–H]^-$: 389.0131, found 389.0147.

2-(3,5-Bis(trifluoromethyl)phenyl)pyrene-1-carboxylic acid (3k)

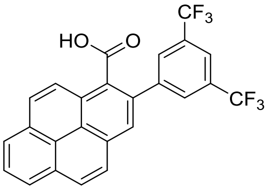

Prepared according to general procedure A using pyrene-1-carboxylic acid (100 mg, 0.406 mmol) and 1-iodo-3,5-bis(trifluoromethyl)benzene (216 μL, 1.22 mmol). The crude product was purified by silica flash column chromatography (hexane/EtOAc, 100:0 to 50:50) to yield the title product as a pale orange solid (161 mg, 87%), mp (CH_2Cl_2) >260 °C. ^{1}H-NMR (500 MHz, DMSO-d_6) δ 8.19 (t, J = 7.8 Hz, ArH, 1 H), 8.24-8.46 (m, ArH, 9 H), 8.52 (s, ArH, 1 H), 13.92 (bs, CO_2H, 1 H) ppm; ^{13}C-NMR (125 MHz, DMSO-d_6) δ 121.5 (m, ArCH), 123.2 (ArC), 123.2 (ArC), 123.4 (q, J = 271.1 Hz, ArCCF_3), 124.1 (ArCH), 126.1 (ArCH), 126.2 (ArCH), 126.4 (ArCH), 127.1 (ArCH), 127.2 (ArC), 127.2 (ArCH), 129.1 (ArCH), 129.3 (ArC), 129.4 (ArCH), 129.7 (q, J = 3.3 Hz, ArCH), 130.2 (ArC), 130.5 (q, J = 32.7 Hz, ArCCF$_3$), 130.8 (ArC), 131.2 (ArC), 133.3 (ArC), 143.3 (ArC), 170.2

($ArCO_2H$) ppm; ^{19}F-NMR (471 MHz, DMSO-d$_6$) δ –61.1 ppm; IR ν_{max} (neat/cm^{-1}): 2930, 1678, 1379, 1285, 1171, 1117; HRMS calcd for $C_{25}H_{11}O_2F_6$ [M–H]$^-$: 457.0669, found 457.0672.

2-(3-Nitrophenyl)pyrene-1-carboxylic acid (3l)

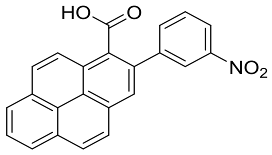

Prepared according to general procedure A using pyrene-1-carboxylic acid (100 mg, 0.406 mmol) and 3-nitroiodobenzene (304 mg, 1.22 mmol). The crude product was purified by silica flash column chromatography (hexane/EtOAc, 100:0 to 20:80) to yield the title product as an orange solid (123 mg, 82%), mp (CH_2Cl_2) 222-225 ºC. ^{1}H-NMR (500 MHz, DMSO-d$_6$) δ 7.88 (t, *J* = 8.0 Hz, Ar*H*, 1 H), 8.14 (ddd, *J* = 8.0, 2.0, 1.0 Hz, Ar*H*, 1 H), 8.18 (t, *J* = 8.0 Hz, Ar*H*, 1 H), 8.26 (d, *J* = 9.0 Hz, Ar*H*, 1 H), 8.31 (d, *J* = 9.0 Hz, Ar*H*, 1 H), 8.33-8.40 (m, Ar*H*, 3 H), 8.40-8.44 (m, Ar*H*, 2 H), 8.47 (s, Ar*H*, 1 H), 8.49 (t, *J* = 2.0 Hz, Ar*H*, 1 H), 13.82 (s, CO_2H, 1 H) ppm; ^{13}C-NMR (125 MHz, DMSO-d$_6$) δ 122.6 (Ar*C*H), 123.0 (Ar*C*), 123.2 (Ar*C*), 123.4 (Ar*C*H), 124.1 (Ar*C*H), 126.0 (Ar*C*H), 126.1 (Ar*C*H), 126.4 (Ar*C*H), 127.1 (Ar*C*H), 127.1 (Ar*C*), 127.2 (Ar*C*H), 129.0 (Ar*C*H), 129.4 (Ar*C*H), 130.2 (Ar*C*), 130.2 (Ar*C*H), 130.8 (Ar*C*), 131.3 (Ar*C*), 134.1 (Ar*C*), 135.7 (Ar*C*H), 142.3 (Ar*C*), 147.8 (Ar*C*), 170.2 ($ArCO_2H$) ppm, one Ar*C* was not observed; IR ν_{max} (neat/cm^{-1}): 1674, 1520, 1349; HRMS calcd for $C_{23}H_{12}O_4N$ [M–H]$^-$: 366.0772, found 366.0774.

2-(3-(Trifluoromethyl)phenyl)pyrene-1-carboxylic acid (3m)

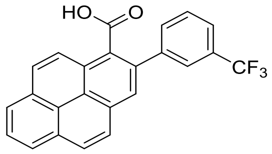

Prepared according to general procedure A using pyrene-1-carboxylic acid (100 mg, 0.406 mmol) and 3-trifluoromethyliodobenzene (176 μL, 1.22 mmol). The crude product was purified by silica flash column chromatography (hexane/EtOAc, 100:0 to 60:40) to yield the title product as a pale brown solid (133 mg, 84%), mp (CH_2Cl_2) 230-233 ºC. ^{1}H-NMR (400 MHz, DMSO-d$_6$) δ 7.81 (t, *J* = 8.0 Hz, Ar*H*, 1 H), 7.87 (d, *J* = 8.0 Hz, Ar*H*, 1 H), 7.95-8.01 (m, Ar*H*, 2 H), 8.17 (t, *J* = 7.6 Hz, Ar*H*, 1 H), 8.23 (d, *J* = 9.2 Hz, Ar*H*, 1 H), 8.30 (d, *J* = 8.8 Hz, Ar*H*, 1 H), 8.33 (d, *J* = 8.8 Hz, Ar*H*, 1 H), 8.37 (d, *J* = 9.2 Hz, Ar*H*, 1 H), 8.39-8.46 (m, Ar*H*, 3 H), 13.77 (s, CO_2H, 1 H) ppm; ^{13}C-NMR (125 MHz, DMSO-d$_6$) δ 122.8 (Ar*C*), 123.3 (Ar*C*), 124.1 (Ar*C*H), 124.2 (q, J = 271.3 Hz, ArCCF_3), 124.4 (q, J = Hz, Ar*C*H), 125.4 (q, J = Hz, Ar*C*H), 126.0 (Ar*C*H), 126.1 (Ar*C*H), 126.3 (Ar*C*H), 127.0 (Ar*C*H), 127.0 (Ar*C*), 127.2 (Ar*C*H), 128.9 (Ar*C*H), 129.3 (q, *J* = 31.3 Hz, Ar*C*CF_3), 129.3 (Ar*C*H), 129.7 (Ar*C*H), 130.1 (Ar*C*), 130.8 (Ar*C*), 131.2 (Ar*C*), 133.2 (Ar*C*H), 134.8 (Ar*C*), 141.7 (Ar*C*), 170.3 ($ArCO_2H$) ppm, one Ar*C* was not observed; ^{19}F-NMR (471 MHz, DMSO-d$_6$) –60.9 ppm; IR ν_{max} (neat/cm^{-1}): 3037, 1683, 1337, 1274, 1121; HRMS calcd for $C_{24}H_{12}O_2F_3$ [M–H]$^-$: 389.0795, found 389.0796.

2-(3-Cyanophenyl)pyrene-1-carboxylic acid (3n)

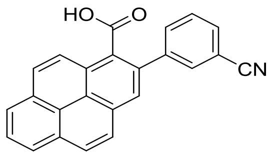

Prepared according to general procedure A using pyrene-1-carboxylic acid (100 mg, 0.406 mmol) and 3-iodobenzonitrile (279 mg, 1.22 mmol). The crude product was purified by silica flash column chromatography (hexane/EtOAc, 100:0 to 30:70) to yield the title product as a pale orange solid (104 mg, 74%), mp (CH_2Cl_2) 215-217 ºC. ^{1}H-NMR (500 MHz, acetone-d$_6$) δ 7.77 (t, *J* = 8.0 Hz, Ar*H*, 1 H), 7.89 (d, *J* = 8.0 Hz, Ar*H*, 1 H), 8.03 (d, *J* = 8.0 Hz, Ar*H*, 1 H), 8.08 (s, Ar*H*, 1 H), 8.16 (t, *J* = 7.5 Hz, Ar*H*, 1 H), 8.26 (d, *J* = 9.0 Hz, Ar*H*, 1 H), 8.29-8.42 (m, Ar*H*, 6 H), 12.06 (bs, CO_2*H*, 1 H) ppm; ^{13}C-NMR (125 MHz, acetone-d$_6$) δ 113.3 (Ar*C*), 119.3 (Ar*C*N), 124.5 (Ar*C*), 124.8 (Ar*C*), 125.2 (Ar*C*H), 126.9 (Ar*C*H), 127.0 (Ar*C*H), 127.2 (Ar*C*H), 127.8 (Ar*C*H), 128.1 (Ar*C*H), 128.9 (Ar*C*), 129.7 (Ar*C*), 130.0 (Ar*C*H), 130.2 (Ar*C*H), 130.5 (Ar*C*H), 131.6 (Ar*C*), 132.1 (Ar*C*H), 132.2 (Ar*C*), 132.8 (Ar*C*), 133.4 (Ar*C*H), 134.7 (Ar*C*H), 136.1 (Ar*C*), 143.6 (Ar*C*), 170.5 (Ar*C*O_2H) ppm; IR ν_{max} (neat/cm^{-1}): 3043, 2229, 1722, 1695, 1597, 1404, 1245; HRMS calcd for $C_{24}H_{12}O_2N$ [M–H]$^-$: 346.0874, found 346.0874.

2-(3-(Methoxycarbonyl)phenyl)pyrene-1-carboxylic acid (3o)

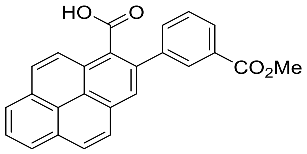

Prepared according to general procedure A using pyrene-1-carboxylic acid (100 mg, 0.406 mmol) and methyl 3-iodobenzoate (320 mg, 1.22 mmol). The crude product was purified by silica flash column chromatography (hexane/EtOAc, 100:0 to 30:70) to yield the title product as an orange solid (105 mg, 68%), mp (CH_2Cl_2) 201-204 ºC. ^{1}H-NMR (400 MHz, DMSO-d$_6$) δ 3.91 (s, CO_2C*H*$_3$, 3 H), 7.72 (t, *J* = 7.6 Hz, Ar*H*, 1 H), 7.95 (ddd, *J* = 7.6, 1.6, 1.2 Hz, Ar*H*, 1 H), 8.08 (dt, *J* = 7.6, 1.2 Hz, Ar*H*, 1 H), 8.16 (t, *J* = 7.6 Hz, Ar*H*, 1 H), 8.21-8.26 (m, Ar*H*, 2 H), 8.30 (d, *J* = 9.0 Hz, Ar*H*, 1 H), 8.33 (d, *J* = 9.0 Hz, Ar*H*, 1 H), 8.37 (d, *J* = 8.8 Hz, Ar*H*, 1 H), 8.38-8.43 (m, Ar*H*, 3 H), 13.69 (s, CO_2*H*, 1 H) ppm; ^{13}C-NMR (125 MHz, DMSO-d$_6$) δ 52.4 ($ArCO_2CH_3$), 122.8 (Ar*C*), 123.3 (Ar*C*), 124.1 (Ar*C*H), 124.1 (Ar*C*), 126.0 (Ar*C*H), 126.0 (Ar*C*H), 126.3 (Ar*C*H), 126.9 (Ar*C*H), 127.0 (Ar*C*), 127.2 (Ar*C*H), 128.3 (Ar*C*H), 128.8 (Ar*C*H), 129.2 (Ar*C*H), 129.2 (Ar*C*H), 129.5 (Ar*C*H), 129.9 (Ar*C*), 130.1 (Ar*C*), 130.7 (Ar*C*), 131.2 (Ar*C*), 133.8 (Ar*C*H), 135.4 (Ar*C*), 141.2 (Ar*C*), 166.1 (Ar*C*O_2CH_3), 170.3 (Ar*C*O_2H) ppm; IR ν_{max} (neat/cm^{-1}): 3166, 1716, 1683, 1594, 1436, 1207; HRMS calcd for $C_{25}H_{15}O_4$ [M–H]$^-$: 379.0976, found 379.0974.

2-(4-(Methoxycarbonyl)phenyl)pyrene-1-carboxylic acid (3p)

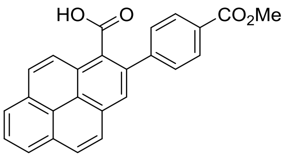

Prepared according to general procedure A, but using a tick glass wall pressure tube as reaction vessel with pyrene-1-carboxylic acid (739 mg, 3.00 mmol) and methyl 4-iodobenzoate (2.36 g, 9.00 mmol). The crude product was purified by silica flash column chromatography (hexane/EtOAc, 100:0 to 40:60) to yield the title product as an orange solid (1.06 g, 90%), mp (CH_2Cl_2) 225-227 ºC. ^{1}H-NMR (500 MHz, DMSO-d$_6$) δ 3.92 (s, OC*H*$_3$, 3 H), 7.82 (d, *J* = 7.5 Hz, Ar*H*, 2 H), 8.13 (d, *J* = 7.5 Hz, Ar*H*, 2 H), 8.17 (t, *J* = 7.5 Hz, Ar*H*, 1 H), 8.24 (d, *J* = 9.3 Hz, Ar*H*, 1 H), 8.29 (d, *J* = 9.0 Hz, Ar*H*, 1 H), 8.33 (d, *J* = 9.0 Hz, Ar*H*, 1 H), 8.37 (d, *J* = 9.3 Hz, Ar*H*, 1 H), 8.39-8.43 (m, Ar*H*, 3 H), 13.68 (bs, CO_2*H*, 1 H) ppm; ^{13}C-NMR (125 MHz, DMSO-d$_6$) δ 52.3 (O*C*H$_3$), 122.9 (Ar*C*), 123.3 (Ar*C*), 124.1 (Ar*C*H), 125.9 (Ar*C*H), 126.0 (Ar*C*H), 126.3 (Ar*C*H), 127.0 (Ar*C*H), 127.1 (Ar*C*), 127.2 (Ar*C*H), 128.7 (Ar*C*), 128.9 (Ar*C*H), 129.2 (Ar*C*H), 129.2 (Ar*C*), 129.3 (Ar*C*H), 129.4 (Ar*C*H), 130.1 (Ar*C*), 130.8 (Ar*C*), 131.1 (Ar*C*), 135.4 (Ar*C*), 145.6 (Ar*C*), 166.1 (*C*O$_2$CH$_3$), 170.3 (*C*O$_2$H) ppm; IR ν_{max} (neat/cm^{-1}): 3042, 2924, 1723, 1669, 1427, 1298, 1280; HRMS calcd for $C_{25}H_{15}O_4$ [M–H]$^-$: 379.0976, found 379.0977.

General Procedure B: Iododecarboxylation of pyrenecarboxylic acids

2-(3,5-Dimethylphenyl)-1-iodopyrene (4a)

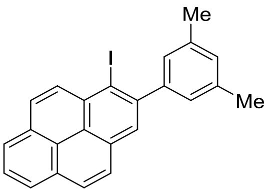

To a flame dried microwave vial fitted with a Teflon-coated stirring bar and loaded with **3a** (500 mg, 1.43 mmol), iodine (1.09 g, 4.29 mmol) and dry K_3PO_4 (303 mg, 1.43 mmol) was added dry 1,2-dichlorobenzene (7.2 mL). The vial was sealed under air and the mixture was stirred at 120 ˚C for 21 h. After cooling to room temperature, the reaction was quenched with 10% aqueous Na_2S2O_3 / saturated aqueous Na_2CO_3 (1:1, 20 mL) and extracted with CH_2Cl_2 (3 × 15 mL). The organic layer was washed with brine, dried ($MgSO_4$) and concentrated under reduced pressure. The crude product was purified by silica flash column chromatography (hexane) to yield the title product as an off-white solid (422 mg, 68%) , mp (CH_2Cl_2) 160-163 ºC. ^{1}H-NMR (400 MHz, CDCl$_3$) δ 2.45 (s, C*H*$_3$, 6 H), 7.13 (s, Ar*H*, 3 H), 8.00-8.09 (m, Ar*H*, 2 H), 8.10-8.17 (m, Ar*H*, 2 H), 8.18-8.30 (m, Ar*H*, 3 H), 8.58 (d, *J* = 9.2 Hz, Ar*H*, 1 H) ppm; ^{13}C-NMR (100 MHz, CDCl$_3$) δ 21.4 (*C*H$_3$), 101.7 (Ar*C*I), 123.7 (Ar*C*), 124.2 (Ar*C*), 125.5 (Ar*C*H), 125.8 (Ar*C*H), 125.9 (Ar*C*H), 126.4 (Ar*C*H), 127.0 (Ar*C*H), 127.8 (Ar*C*H), 128.3 (Ar*C*H), 129.2 (Ar*C*H), 129.6 (Ar*C*H), 130.8 (Ar*C*), 130.8 (Ar*C*), 131.1 (Ar*C*), 132.5 (Ar*C*H), 133.2 (Ar*C*), 137.3 (Ar*C*), 145.8 (Ar*C*), 146.1 (Ar*C*) ppm; IR ν_{max} (neat/cm^{-1}): 3033, 2915, 1602, 1587, 1422, 1008, 881; HRMS calcd for $C_{24}H_{17}I$ [M]$^+$: 432.0369, found 432.0359.

1-Iodo-2-(4-methylphenyl)pyrene (4b)

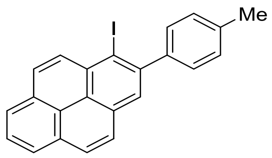

Prepared according to general procedure B using **3b** (404 mg, 1.20 mmol). The crude product was purified by silica flash column chromatography (hexane/CH_2Cl_2, 100:0 to 98:2) to yield the title product as a pale yellow solid (404 mg, 81%), mp (CH_2Cl_2) 90-93 ºC. ^{1}H-NMR (400 MHz, $CDCl_3$) δ 2.51 (s, C*H*$_3$, 3 H), 7.35 (d, *J* = 8.2 Hz, Ar*H*, 2 H), 7.42 (d, *J* = 8.2 Hz, Ar*H*, 2 H), 7.99-8.09 (m, Ar*H*, 2 H), 8.10-8.16 (m, Ar*H*, 2 H), 8.17-8.30 (m, Ar*H*, 3 H), 8.57 (d, *J* = 9.2 Hz, Ar*H*, 1 H) ppm; ^{13}C-NMR (125 MHz, $CDCl_3$) δ 21.4 (*C*H$_3$), 101.9 (Ar*C*I), 123.8 (Ar*C*), 124.3 (Ar*C*), 125.6 (Ar*C*H), 125.8 (Ar*C*H), 126.1 (Ar*C*H), 126.4 (Ar*C*H), 127.0 (Ar*C*H), 128.4 (Ar*C*H), 128.6 (Ar*C*H), 129.7 (Ar*C*H), 129.8 (Ar*C*H), 130.8 (Ar*C*), 130.8 (Ar*C*), 131.1 (Ar*C*), 132.5 (Ar*C*H), 133.3 (Ar*C*), 137.4 (Ar*C*), 143.4 (Ar*C*), 145.5 (Ar*C*) ppm; IR ν_{max} (neat/cm^{-1}): 2921, 2852, 1739, 1588, 1509, 1447, 1177, 1002; HRMS calcd for $C_{23}H_{16}I$ $[M+H]^+$: 419.0291, found 419.0284.

1-Iodo-2-(3-methylphenyl)pyrene (4c)

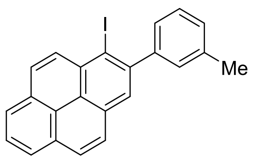

Prepared according to general procedure B using **3c** (34 mg, 0.100 mmol). The crude product was purified by silica flash column chromatography (hexane) to yield the title product as a pale yellow solid (31 mg, 74%), mp (CH_2Cl_2) 103-105 ºC. ^{1}H-NMR (400 MHz, $CDCl_3$) δ 2.50 (s, C*H*$_3$, 3 H), 7.29-7.36 (m, Ar*H*, 3 H), 7.40-7.46 (m, Ar*H*, 1 H), 8.01 (d, *J* = 8.8 Hz, Ar*H*, 1 H), 8.05 (t, *J* = 7.6 Hz, Ar*H*, 1 H), 8.09-8.15 (m, Ar*H*, 2 H), 8.18 (d, *J* = 9.4 Hz, Ar*H*, 1 H), 8.20-8.29 (m, Ar*H*, 2 H), 8.56 (d, *J* = 9.4 Hz, Ar*H*, 1 H) ppm; ^{13}C-NMR (125 MHz, $CDCl_3$) δ 21.6 (*C*H$_3$), 101.7 (Ar*C*I), 123.7 (Ar*C*), 124.2 (Ar*C*), 125.5 (Ar*C*H), 125.8 (Ar*C*H), 125.9 (Ar*C*H), 126.4 (Ar*C*H), 126.9 (Ar*C*H), 127.1 (Ar*C*H), 127.7 (Ar*C*H), 128.3 (Ar*C*H), 128.3 (Ar*C*H), 129.7 (Ar*C*H), 130.6 (Ar*C*H), 130.8 (Ar*C*), 130.8 (Ar*C*), 131.0 (Ar*C*), 132.5 (Ar*C*H), 133.2 (Ar*C*), 137.5 (Ar*C*), 145.6 (Ar*C*), 146.1 (Ar*C*) ppm; IR ν_{max} (neat/cm^{-1}): 3042, 2920, 1587, 1422; HRMS calcd for $C_{13}H_{16}I$ $[M+H]^+$: 419.0291, found 419.0285.

2-(4-*tert*-Butylphenyl)-1-iodopyrene (4d)

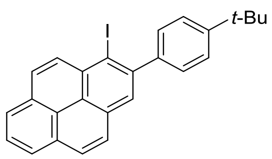

Prepared according to general procedure B using **3d** (38 mg, 0.100 mmol). The crude product was purified by silica flash column chromatography (hexane) to yield the title product as a white solid (28 mg, 61%), mp (CH_2Cl_2) 130-133 ºC. ^{1}H-NMR (400 MHz, $CDCl_3$) δ 1.45 (s, C(C*H*$_3$)$_3$, 9 H), 7.46 (d, *J*

= 8.2 Hz, Ar*H*, 2 H), 7.55 (d, *J* = 8.2 Hz, Ar*H*, 2 H), 7.99-8.09 (m, Ar*H*, 2 H), 8.11-8.16 (m, Ar*H*, 2 H), 8.18-8.31 (m, Ar*H*, 3 H), 8.58 (d, *J* = 9.2 Hz, Ar*H*, 1 H) ppm; ^{13}C-NMR (125 MHz, $CDCl_3$) δ 31.5 (C(CH_3)$_3$), 34.7 (*C*(CH_3)$_3$), 101.9 (Ar*C*I), 123.8 (Ar*C*), 124.3 (Ar*C*), 124.8 (Ar*C*H), 125.6 (Ar*C*H), 125.8 (Ar*C*H), 126.2 (Ar*C*H), 126.4 (Ar*C*H), 127.0 (Ar*C*H), 128.3 (Ar*C*H), 129.6 (Ar*C*H), 129.7 (Ar*C*H), 130.8 (Ar*C*), 130.9 (Ar*C*), 131.1 (Ar*C*), 132.6 (Ar*C*H), 133.3 (Ar*C*), 143.2 (Ar*C*), 145.6 (Ar*C*), 150.5 (Ar*C*) ppm; IR ν_{max} (neat/cm^{-1}): 3041, 2949, 1578, 1509, 1420, 1397, 1360, 1180, 1115, 877; HRMS calcd for $C_{26}H_{21}I$ [M]$^+$: 460.0682, found 460.0681.

1-Iodo-2-(4-methoxyphenyl)pyrene (4e)

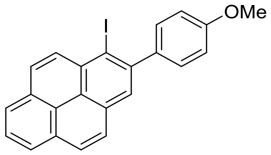

Prepared according to general procedure B using **3e** (400 mg, 1.13 mmol). The crude product was purified by silica flash column chromatography (hexane/CH_2Cl_2, 100:0 to 90:10) to yield the title product as a white solid (370 mg, 75%), mp (CH_2Cl_2) 139-142 ºC. ^{1}H-NMR (400 MHz, $CDCl_3$) δ 3.94 (s, OC*H*$_3$, 3 H),7.06 (d, *J* = 8.4 Hz, Ar*H*, 2 H), 7.45 (d, *J* = 8.4 Hz, Ar*H*, 2 H), 7.99-8.10 (m, Ar*H*, 2 H), 8.11-8.17 (m, Ar*H*, 2 H), 8.18-8.29 (m, Ar*H*, 3 H), 8.57 (d, *J* = 9.2 Hz, Ar*H*, 1 H) ppm; ^{13}C-NMR (125 MHz, $CDCl_3$) δ 55.4 (O*C*H$_3$), 102.4 (Ar*C*I), 113.3 (Ar*C*H), 115.0 (Ar*C*), 123.8 (Ar*C*), 124.3 (Ar*C*), 125.6 (Ar*C*H), 125.9 (Ar*C*H), 126.2 (Ar*C*H), 126.4 (Ar*C*H), 127.0 (Ar*C*H), 128.4 (Ar*C*H), 129.7 (Ar*C*H), 130.9 (Ar*C*), 130.9 (Ar*C*), 131.1 (Ar*C*H), 132.6 (Ar*C*H), 133.4 (Ar*C*), 138.9 (Ar*C*), 145.2 (Ar*C*), 159.1 (Ar*C*) ppm; IR ν_{max} (neat/cm^{-1}): 3033, 2836, 1609, 1511, 1286, 1242, 1174, 1024; HRMS calcd for $C_{23}H_{15}OI$ [M]$^+$: 434.0162, found 434.0162.

1-Iodo-2-phenylpyrene (3f)

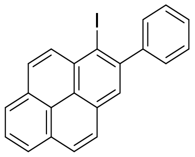

Prepared according to general procedure B using **3f** (81 mg, 0.250 mmol). The crude product was purified by silica flash column chromatography (hexane/CH_2Cl_2, 100:0 to 98:2) to yield the title product as a yellow solid (27 mg, 27%), mp (CH_2Cl_2) 137-140 ºC. ^{1}H-NMR (400 MHz, $CDCl_3$) δ 7.48-7.57 (m, Ar*H*, 5 H), 8.01 (d, *J* = 9.2 Hz, Ar*H*, 1 H), 8.05 (t, *J* = 7.6 Hz, Ar*H*, 1 H), 8.10-8.14 (m, Ar*H*, 2 H), 8.18 (d, *J* = 9.2 Hz, Ar*H*, 1 H), 8.20-8.28 (m, Ar*H*, 2 H), 8.56 (d, *J* = 9.2 Hz, Ar*H*, 1 H) ppm; ^{13}C-NMR (100 MHz, $CDCl_3$) δ 101.6 (Ar*C*I), 123.7 (Ar*C*), 124.3 (Ar*C*), 125.6 (Ar*C*H), 125.9 (Ar*C*H), 125.9 (Ar*C*H), 126.4 (Ar*C*H), 126.9 (Ar*C*H), 127.6 (Ar*C*H), 127.9 (Ar*C*H), 128.4 (Ar*C*H), 129.7 (Ar*C*H), 129.9 (Ar*C*H), 130.8 (Ar*C*), 130.8 (Ar*C*), 131.1 (Ar*C*), 132.4 (Ar*C*H), 133.3 (Ar*C*), 145.5 (Ar*C*), 146.2 (Ar*C*) ppm; IR ν_{max} (neat/cm^{-1}): 3047, 1587, 1495, 1421, 1180, 1005, 839, 820; HRMS calcd for $C_{24}H_{14}I$ [M+H]$^+$: 405.0135, found 405.0136.

2-(4-Bromophenyl)-1-iodopyrene (4g)

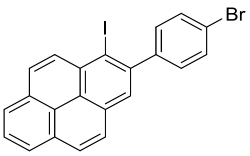

Prepared according to general procedure B using **3g** (100 mg, 0.250 mmol). The crude product was purified by silica flash column chromatography (hexane) to yield the title product as a pale yellow solid (37 mg, 31%), mp (CH_2Cl_2) 172-175 ºC. ^{1}H-NMR (400 MHz, $CDCl_3$) δ 7.39 (d, *J* = 8.4 Hz, Ar*H*, 2 H), 7.67 (d, *J* = 8.4 Hz, Ar*H*, 2 H), 7.98 (d, *J* = 8.8 Hz, Ar*H*, 1 H), 8.02-8.08 (m, Ar*H*, 2 H), 8.11 (d, *J* = 8.8 Hz, Ar*H*, 1 H), 8.14-8.27 (m, Ar*H*, 3 H), 8.52 (d, *J* = 9.2 Hz, Ar*H*, 1 H) ppm; ^{13}C-NMR (100 MHz, $CDCl_3$) δ 101.1 (Ar*C*I), 122.0 (Ar*C*), 123.6 (Ar*C*), 124.4 (Ar*C*), 125.6 (Ar*C*H), 125.7 (Ar*C*H), 126.0 (Ar*C*H), 126.5 (Ar*C*H), 126.8 (Ar*C*H), 128.5 (Ar*C*H), 129.9 (Ar*C*H), 130.8 (Ar*C*), 130.8 (Ar*C*), 131.0 (Ar*C*), 131.1 (Ar*C*H), 131.6 (Ar*C*H), 132.3 (Ar*C*H), 133.3 (Ar*C*), 144.2 (Ar*C*), 145.0 (Ar*C*) ppm; IR ν_{max} (neat/cm^{-1}): 3044, 2922, 1586, 1488, 1422, 1179, 1068, 1011, 840, 829, 819; HRMS calcd for $C_{22}H_{13}BrI$ $[M+H]^+$: 482.9240, found 482.9242.

1-Iodo-2-(3-bromophenyl)pyrene (4h)

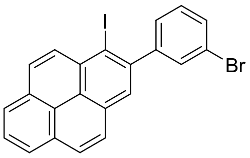

Prepared according to general procedure B using **3h** (40 mg, 0.100 mmol). The crude product was purified by silica flash column chromatography (hexane) to yield the title product as a pale yellow solid (34 mg, 70%), mp (CH_2Cl_2) 147-150 ºC. ^{1}H-NMR (400 MHz, $CDCl_3$) δ 7.40 (t, *J* = 7.6 Hz, Ar*H*, 1 H), 7.46 (dt, *J* = 7.6, 1.2 Hz, Ar*H*, 1 H), 7.64 (ddd, *J* = 7.6, 1.6, 1.2 Hz, Ar*H*, 1 H), 7.68 (t, *J* = 7.6, 1.6 Hz, Ar*H*, 1 H), 7.99 (d, *J* = 9.0 Hz, Ar*H*, 1 H), 8.02-8.09 (m, Ar*H*, 2 H), 8.12 (d, *J* = 9.0 Hz, Ar*H*, 1 H), 8.17 (d, *J* = 9.6 Hz, Ar*H*, 1 H), 8.20-8.28 (m, Ar*H*, 2 H), 8.51 (d, *J* = 9.2 Hz, Ar*H*, 1 H) ppm; ^{13}C-NMR (125 MHz, $CDCl_3$) δ 100.9 (Ar*C*I), 121.8 (Ar*C*), 123.5 (Ar*C*), 124.4 (Ar*C*), 125.6 (Ar*C*H), 125.7 (Ar*C*H), 126.0 (Ar*C*H), 126.5 (Ar*C*H), 126.8 (Ar*C*H), 128.5 (Ar*C*H), 128.7 (Ar*C*H), 129.4 (Ar*C*H), 129.9 (Ar*C*H), 130.7 (Ar*C*H), 130.8 (Ar*C*), 130.8 (Ar*C*), 131.0 (Ar*C*), 132.2 (Ar*C*H), 132.9 (Ar*C*H), 133.3 (Ar*C*), 143.8 (Ar*C*), 148.0 (Ar*C*) ppm; IR ν_{max} (neat/cm^{-1}): 3046, 2924, 2853, 1587, 1558, 1473, 1422; HRMS calcd for $C_{22}H_{12}BrI$ $[M]^+$: 481.9162, found 481.9155.

1-Iodo-2-(3-chlorophenyl)pyrene (4i)

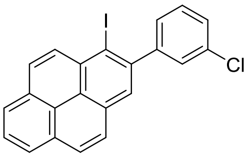

Prepared according to general procedure B using **3i** (36 mg, 0.100 mmol). The crude product was purified by silica flash column chromatography (hexane) to yield the title product as a pale yellow

solid (30 mg, 68%), mp (CH_2Cl_2) 135-138 ºC. ^{1}H-NMR (400 MHz, $CDCl_3$) δ 7.40 (dt, *J* = 6.6, 2.0 Hz, Ar*H*, 1 H), 7.43-7.50 (m, Ar*H*, 2 H), 7.50-7.53 (m, Ar*H*, 1 H), 7.99 (d, *J* = 9.0 Hz, Ar*H*, 1 H), 8.03-8.09 (m, Ar*H*, 2 H), 8.12 (d, *J* = 9.0 Hz, Ar*H*, 1 H), 8.18 (d, *J* = 9.2 Hz, Ar*H*, 1 H), 8.21-8.28 (m, Ar*H*, 2 H), 8.52 (d, *J* = 9.2 Hz, Ar*H*, 1 H) ppm; ^{13}C-NMR (125 MHz, $CDCl_3$) δ 100.9 (Ar*C*I), 123.6 (Ar*C*), 124.4 (Ar*C*), 125.6 (Ar*C*H), 125.7 (Ar*C*H), 126.0 (Ar*C*H), 126.6 (Ar*C*H), 126.8 (Ar*C*H), 127.8 (Ar*C*H), 128.3 (Ar*C*H), 128.5 (Ar*C*H), 129.1 (Ar*C*H), 129.9 (Ar*C*H), 130.0 (Ar*C*H), 130.8 (Ar*C*), 130.8 (Ar*C*), 131.0 (Ar*C*), 132.3 (Ar*C*H), 133.3 (Ar*C*), 133.7 (Ar*C*), 143.9 (Ar*C*), 147.7 (Ar*C*) ppm; IR ν_{max} (neat/cm^{-1}): 3046, 2925, 1587, 1563, 1474, 1422; HRMS calcd for $C_{22}H_{12}ClI$ $[M]^+$: 437.9667, found 437.9660.

1-Iodo-2-(3,5-dichlorophenyl)pyrene (4j)

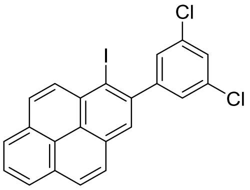

Prepared according to general procedure B using **3j** (39 mg, 0.100 mmol). The crude product was purified by silica flash column chromatography (hexane) to yield the title product as an off-white solid (19 mg, 42%), mp (CH_2Cl_2) 175-178 ºC. ^{1}H-NMR (500 MHz, $CDCl_3$) δ 7.41 (d, *J* = 1.5 Hz, Ar*H*, 2 H), 7.50 (bs, Ar*H*, 1 H), 8.00 (d, *J* = 8.8 Hz, Ar*H*, 1 H), 8.04 (s, Ar*H*, 1 H), 8.07 (t, *J* = 7.5 Hz, Ar*H*, 1 H), 8.15 (d, *J* = 8.8 Hz, Ar*H*, 1 H), 8.20 (d, *J* = 9.5 Hz, Ar*H*, 1 H), 8.23-8.29 (m, Ar*H*, 2 H), 8.52 (d, *J* = 9.5 Hz, Ar*H*, 1 H) ppm; ^{13}C-NMR (125 MHz, $CDCl_3$) δ 100.3 (Ar*C*I), 123.5 (Ar*C*), 124.6 (Ar*C*), 125.4 (Ar*C*H), 125.9 (Ar*C*H), 126.1 (Ar*C*H), 126.7 (Ar*C*H), 126.8 (Ar*C*H), 127.8 (Ar*C*H), 128.6 (Ar*C*H), 128.8 (Ar*C*H), 130.1 (Ar*C*H), 130.9 (Ar*C*), 130.9 (Ar*C*), 131.1 (Ar*C*), 132.2 (Ar*C*H), 133.4 (Ar*C*), 134.4 (Ar*C*Cl), 142.6 (Ar*C*), 148.6 (Ar*C*) ppm; IR ν_{max} (neat/cm^{-1}): 2923, 1584, 1558, 1411, 1097; HRMS calcd for $C_{22}H_{11}Cl_2I$ $[M]^+$: 471.9277, found 471.9277.

2-(3,5-Bis(trifluoromethyl)phenyl)-1-iodopyrene (4k)

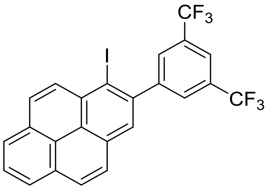

Prepared according to general procedure B using **3k** (46 mg, 0.100 mmol). The crude product was purified by silica flash column chromatography (hexane/CH_2Cl_2, 100:0 to 70:30) to yield the title product as an off-white solid (21 mg, 39%), mp (CH_2Cl_2) 220-222 ºC. ^{1}H-NMR (400 MHz, $CDCl_3$) δ 8.02 (s, Ar*H*, 3 H), 8.06 (d, *J* = 8.8 Hz, Ar*H*, 1 H), 8.09-8.14 (m, Ar*H*, 2 H), 8.20 (d, *J* = 8.8 Hz, Ar*H*, 1 H), 8.24-8.35 (m, Ar*H*, 3 H), 8.55 (d, *J* = 9.2 Hz, Ar*H*, 1 H) ppm; ^{13}C-NMR (100 MHz, $CDCl_3$) δ 100.1 (Ar*C*I), 121.5 (hept, *J* = 3.8 Hz, Ar*C*H), 123.4 (hept, *J* = 271.2 Hz, ArC*C*F$_3$), 123.5 (Ar*C*), 124.8 (Ar*C*), 125.5 (Ar*C*H), 126.0 (Ar*C*H), 126.3 (Ar*C*H), 126.7 (Ar*C*H), 126.9 (Ar*C*H), 129.0 (Ar*C*H), 130.4 (Ar*C*H), 130.4 (m, Ar*C*H), 130.9 (Ar*C*), 131.1 (Ar*C*), 131.2 (Ar*C*), 131.3 (q, *J* = 33.2 Hz, Ar*C*CF$_3$), 132.1 (Ar*C*H), 133.6 (Ar*C*), 142.1 (Ar*C*), 147.7 (Ar*C*), ppm; ^{19}F-NMR (376 MHz,

$CDCl_3$) δ –62.7 ppm; IR $ν_{max}$ (neat/cm^{-1}): 2921, 1375, 1278, 1172, 1117; HRMS calcd for $C_{24}H_{11}F_6I$ $[M]^+$: 539.9804, found 539.9781.

1-Iodo-2-(3-nitrophenyl)pyrene (4l)

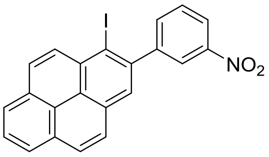

Prepared according to general procedure B using **3l** (37 mg, 0.100 mmol). The crude product was purified by silica flash column chromatography (hexane/CH_2Cl_2, 100:0 to 85:15) to yield the title product as a yellow solid (12 mg, 26%), mp (CH_2Cl_2) 195-198 °C. ^{1}H-NMR (400 MHz, $CDCl_3$) δ 7.71 (t, *J* = 7.8 Hz, Ar*H*, 1 H), 7.87 (d, *J* = 7.8 Hz, Ar*H*, 1 H), 8.04 (d, *J* = 9.2 Hz, Ar*H*, 1 H), 8.06-8.13 (m, Ar*H*, 2 H), 8.18 (d, *J* = 9.2 Hz, Ar*H*, 1 H), 8.23 (d, *J* = 9.4 Hz, Ar*H*, 1 H), 8.25-8.33 (m, Ar*H*, 2 H), 8.34-8.40 (m, Ar*H*, 1 H), 8.40-8.44 (m, Ar*H*, 1 H), 8.53 (d, *J* = 9.4 Hz, Ar*H*, 1 H) ppm; ^{13}C-NMR (125 MHz, $CDCl_3$) δ 100.4 (Ar*C*I), 122.7 (Ar*C*H), 123.6 (Ar*C*), 124.7 (Ar*C*), 125.0 (Ar*C*H), 125.6 (Ar*C*H), 126.0 (Ar*C*H), 126.3 (Ar*C*H), 126.8 (Ar*C*H), 126.8 (Ar*C*H), 128.9 (Ar*C*H), 128.9 (Ar*C*H), 130.2 (Ar*C*H), 130.9 (Ar*C*), 131.1 (Ar*C*), 131.2 (Ar*C*), 132.2 (Ar*C*H), 133.6 (Ar*C*), 136.3 (Ar*C*H), 142.8 (Ar*C*), 147.5 (Ar*C*), 147.9 (Ar*C*) ppm; IR $ν_{max}$ (neat/cm^{-1}): 2922, 2854, 1525, 1350; HRMS calcd for $C_{22}H_{12}O_2NI$ $[M]^+$: 448.9907, found 448.9898.

1-Iodo-2-(4-methoxyphenyl)pyrene (4m)

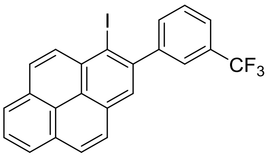

Prepared according to general procedure B using **3m** (39 mg, 0.100 mmol). The crude product was purified by silica flash column chromatography (hexane) to yield the title product as an off-white solid (33 mg, 71%), mp (CH_2Cl_2) 120-123 °C. ^{1}H-NMR (400 MHz, $CDCl_3$) δ 7.66 (t, *J* = 7.6 Hz, Ar*H*, 1 H), 7.72 (d, *J* = 7.6 Hz, Ar*H*, 1 H), 7.77 (d, *J* = 7.6 Hz, Ar*H*, 1 H), 7.81 (s, Ar*H*, 1 H), 8.02 (d, *J* = 9.0 Hz, Ar*H*, 1 H), 8.05-8.12 (m, Ar*H*, 2 H), 8.15 (d, *J* = 9.0 Hz, Ar*H*, 1 H), 8.20 (d, *J* = 9.2 Hz, Ar*H*, 1 H), 8.22-8.31 (m, Ar*H*, 2 H), 8.54 (d, *J* = 9.2 Hz, Ar*H*, 1 H) ppm; ^{13}C-NMR (125 MHz, $CDCl_3$) δ 100.9 (Ar*C*I), 123.6 (Ar*C*), 124.2 (q, *J* = 270.7 Hz, ArC*C*F_3), 124.4 (q, *J* = 3.8 Hz, Ar*C*H), 124.5 (Ar*C*), 125.7 (Ar*C*H), 125.8 (Ar*C*H), 126.1 (Ar*C*H), 126.6 (Ar*C*H), 126.8 (Ar*C*H), 126.9 (q, *J* = 3.8 Hz, Ar*C*H), 128.4 (Ar*C*H), 128.7 (Ar*C*H), 129.9 (Ar*C*H), 130.3 (q, *J* = 32.2 Hz, Ar*C*CF_3), 130.9 (Ar*C*), 130.9 (Ar*C*), 131.1 (Ar*C*), 132.3 (Ar*C*H), 133.4 (q, *J* = 1.2 Hz, Ar*C*H), 133.4 (Ar*C*), 143.8 (Ar*C*), 146.7 (Ar*C*) ppm; ^{19}F-NMR (471 MHz, DMSO-d_6) δ –62.4 ppm; IR $ν_{max}$ (neat/cm^{-1}): 3046, 1587, 1420, 1343, 1329, 1165, 1123; HRMS calcd for $C_{23}H_{12}F_3I$ $[M]^+$: 471.9930, found 471.9926.

1-Iodo-2-(3-cyanophenyl)pyrene (4n)

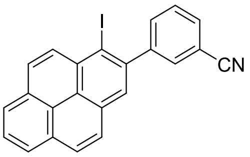

Prepared according to general procedure B using **3n** (35 mg, 0.100 mmol). The crude product was purified by silica flash column chromatography (hexane/CH_2Cl_2, 100:0 to 70:30) to yield the title product as a pale yellow solid (17 mg, 40%), mp (CH_2Cl_2) 146-148 ºC. ^{1}H-NMR (500 MHz, $CDCl_3$) δ 7.64 (t, *J* = 7.8 Hz, Ar*H*, 1 H), 7.74-7.83 (m, Ar*H*, 3 H), 8.03 (d, *J* = 8.8 Hz, Ar*H*, 1 H), 8.05-8.11 (m, Ar*H*, 2 H), 8.17 (d, *J* = 8.8 Hz, Ar*H*, 1 H), 8.23 (d, *J* = 9.2 Hz, Ar*H*, 1 H), 8.25-8.32 (m, Ar*H*, 2 H), 8.54 (d, *J* = 9.2 Hz, Ar*H*, 1 H) ppm; ^{13}C-NMR (100 MHz, $CDCl_3$) δ 100.5 (Ar*C*I), 112.2 (Ar*C*), 118.8 (Ar*C*N), 123.5 (Ar*C*), 124.6 (Ar*C*), 125.5 (Ar*C*H), 125.9 (Ar*C*H), 126.2 (Ar*C*H), 126.7 (Ar*C*H), 126.8 (Ar*C*H), 128.8 (Ar*C*H), 128.8 (Ar*C*H), 130.2 (Ar*C*H), 130.8 (Ar*C*), 131.0 (Ar*C*), 131.1 (Ar*C*), 131.3 (Ar*C*H), 132.1 (Ar*C*H), 133.5 (Ar*C*H + Ar*C*), 134.5 (Ar*C*H), 142.9 (Ar*C*), 147.1 (Ar*C*) ppm; IR ν_{max} (neat/cm^{-1}): 3046, 2923, 2228, 1579, 1481, 1414, 1263, 1007; HRMS calcd for $C_{23}H_{12}NI$ $[M]^+$: 429.0009, found 429.0018.

1-Iodo-2-(3-(methoxycarbonyl)phenyl)pyrene (4o)

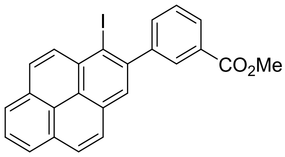

Prepared according to general procedure B using **3o** (38 mg, 0.100 mmol). The crude product was purified by silica flash column chromatography (hexane/CH_2Cl_2, 100:0 to 50:50) to yield the title product as an off-white solid (32 mg, 69%), mp (CH_2Cl_2) 135-138 ºC. ^{1}H-NMR (400 MHz, $CDCl_3$) δ 3.97 (s, CO_2CH_3, 3 H), 7.61 (t, *J* = 7.6 Hz, Ar*H*, 1 H), 7.73 (d, *J* = 7.6 Hz, Ar*H*, 1 H), 8.01 (d, *J* = 8.8 Hz, Ar*H*, 1 H), 8.06 (t, *J* = 7.6 Hz, Ar*H*, 1 H), 8.10 (1, Ar*H*, 1 H), 8.13 (d, *J* = 8.8 Hz, Ar*H*, 1 H), 8.16-8.30 (m, Ar*H*, 5 H), 8.53 (d, *J* = 9.2 Hz, Ar*H*, 1 H) ppm; ^{13}C-NMR (125 MHz, $CDCl_3$) δ 52.2 (CO_2CH_3), 101.1 (Ar*C*I), 123.6 (Ar*C*), 124.4 (Ar*C*), 125.7 (Ar*C*H), 125.8 (Ar*C*H), 126.0 (Ar*C*H), 126.5 (Ar*C*H), 126.9 (Ar*C*H), 128.0 (Ar*C*H), 128.5 (Ar*C*H), 128.8 (Ar*C*H), 129.9 (Ar*C*H), 129.9 (Ar*C*), 130.8 (Ar*C*), 130.9 (Ar*C*), 131.0 (Ar*C*H), 131.1 (Ar*C*), 132.3 (Ar*C*H), 133.4 (Ar*C*), 134.5 (Ar*C*H), 144.3 (Ar*C*), 146.3 (Ar*C*), 167.0 (CO_2CH_3) ppm; IR ν_{max} (neat/cm^{-1}): 3035, 2949, 1720, 1583, 1436, 1281, 1258; HRMS calcd for $C_{24}H_{16}O_2I$ $[M+H]^+$: 463.0189, found 463.0181.

1-Iodo-2-(4-(methoxycarbonyl)phenyl)pyrene (4p)

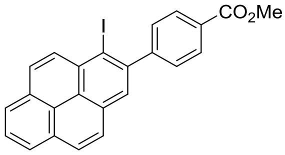

Prepared according to general procedure B using **3p** (100 mg, 0.263 mmol). The crude product was purified by silica flash column chromatography (hexane/CH_2Cl_2, 100:0 to 40:60) to yield the title product as a pale yellow solid (45 mg, 37%), mp (CH_2Cl_2) 170–172 ºC. ^{1}H-NMR (500 MHz, $CDCl_3$) δ 4.01 (s, CO_2CH_3, 3 H), 7.60 (d, *J* = 8.0 Hz, Ar*H*, 2 H), 8.04 (d, *J* = 8.8 Hz, Ar*H*, 1 H), 8.06-8.11 (m, Ar*H*, 2 H), 8.16 (d, *J* = 8.8 Hz, Ar*H*, 1 H), 8.19-8.24 (m, Ar*H*, 3 H), 8.25 (d, *J* = 7.5 Hz, Ar*H*, 1 H), 8.29 (d, *J* = 7.5 Hz, Ar*H*, 1 H), 8.55 (d, *J* = 9.5 Hz, Ar*H*, 1 H) ppm; ^{13}C-NMR (125 MHz, $CDCl_3$) δ 52.2 (CO_2CH_3), 100.5 (Ar*C*I), 123.7 (Ar*C*), 124.5 (Ar*C*), 125.5 (Ar*C*H), 125.8 (Ar*C*H), 126.1 (Ar*C*H), 126.6 (Ar*C*H), 126.9 (Ar*C*H), 128.6 (Ar*C*H), 129.3 (Ar*C*H), 129.4 (Ar*C*), 130.0 (Ar*C*H), 130.1 (Ar*C*H), 130.9 (Ar*C*), 130.9 (Ar*C*), 131.1 (Ar*C*), 132.3 (Ar*C*H), 133.4 (Ar*C*), 144.5 (Ar*C*), 150.6 (Ar*C*), 167.0 (CO_2CH_3) ppm; IR ν_{max} (neat/cm^{-1}): 3046, 2946, 1720, 1608, 1434, 1275, 1116, 1099; HRMS calcd for $C_{24}H_{16}O_2I$ $[M+H]^+$: 463.0189, found 463.0186.

General Procedure C: Palladium catalysed decarboxylative *ortho*-arylation of pyrene-1-carboxylic acid

2-(3,5-Dimethylphenyl)pyrene (5a)

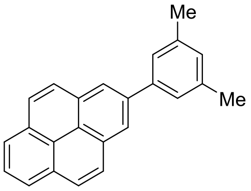

A microwave vial fitted with a Teflon-coated stirring bar was loaded with pyrene-1-carboxylic acid (50 mg, 0.203 mmol), PEPPSI-IPr (2.8 mg, 4.06 μmol), Ag_2CO_3 (56 mg, 0.203 mmol), 5-iodo-ortho-xylene (88 μL, 0.609 mmol) and acetic acid (0.2 mL). The vial was sealed under air and the mixture was stirred at 150 ˚C for 19 h. After cooling to room temperature the reaction mixture was diluted with CH_2Cl_2 and filtered through a short plug of Celite. The crude product was purified by silica flash column chromatography (hexane/CH_2Cl_2, 100:0 to 98:2) to yield the title product as a yellow solid (38 mg, 61%), mp (CH_2Cl_2) 140-143 ºC. ^{1}H-NMR (400 MHz, $CDCl_3$) δ 2.49 (s, C*H*$_3$, 6 H), 7.10 (s, Ar*H*, 1 H), 7.53 (s, Ar*H*, 2 H), 8.01 (t, *J* = 7.6 Hz, Ar*H*, 1 H), 8.11 (d, *J* = 9.0 Hz, Ar*H*, 2 H), 8.14 (d, *J* = 9.0 Hz, Ar*H*, 2 H), 8.20 (d, *J* = 7.6 Hz, Ar*H*, 2 H), 8.40 (s, Ar*H*, 2 H) ppm; ^{13}C-NMR (125 MHz, $CDCl_3$) δ 21.5 (*C*H$_3$), 123.8 (Ar*C*H), 123.8 (Ar*C*), 124.6 (Ar*C*), 125.0 (Ar*C*H), 125.8 (Ar*C*H), 126.0 (Ar*C*H), 127.5 (Ar*C*H), 127.7 (Ar*C*H), 129.0 (Ar*C*H), 131.1 (Ar*C*), 131.5 (Ar*C*), 138.5 (Ar*C*), 139.1 (Ar*C*), 141.5 (Ar*C*) ppm; IR ν_{max} (neat/cm^{-1}):3037, 2914, 1598, 1440, 877, 848, 841, 819 ; HRMS calcd for $C_{24}H_{19}$ $[M+H]^+$: 307.1481, found 307.1473.

2-(4-Methylphenyl)pyrene (5b)

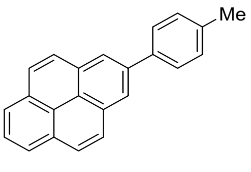

Prepared according to general procedure C using pyrene-1-carboxylic acid (50 mg, 0.203 mmol) and 4-iodotoluene (133 mg, 0.609 mmol). The crude product was purified by silica flash column

chromatography (hexane/CH_2Cl_2, 100:0 to 98:2) to yield the title product as a bright yellow solid (30 mg, 51%), mp (CH_2Cl_2) 110-114 ºC. ^{1}H-NMR (400 MHz, $CDCl_3$) δ 2.48 (s, C*H*$_3$, 3 H), 7.38 (d, *J* = 7.4 Hz, Ar*H*, 2 H), 7.81 (d, *J* = 7.4 Hz, Ar*H*, 2 H), 8.01 (t, *J* = 7.8 Hz, Ar*H*, 1 H), 8.11 (d, *J* = 8.8 Hz, Ar*H*, 2 H), 8.14 (d, *J* = 8.8 Hz, Ar*H*, 2 H), 8.20 (d, *J* = 7.8 Hz, Ar*H*, 2 H), 8.40 (s, Ar*H*, 2 H) ppm; ^{13}C-NMR (100 MHz, $CDCl_3$) δ 21.2 (*C*H$_3$), 123.5 (Ar*C*H), 123.7 (Ar*C*), 124.6 (Ar*C*), 125.1 (Ar*C*H), 125.8 (Ar*C*H), 127.5 (Ar*C*H), 127.7 (Ar*C*H), 127.9 (Ar*C*H), 129.7 (Ar*C*H), 131.1 (Ar*C*), 131.5 (Ar*C*), 137.2 (Ar*C*), 138.6 (Ar*C*), 138.8 (Ar*C*) ppm; IR ν_{max} (neat/cm^{-1}): 3039, 2920, 2853, 1601, 1513, 1441, 1178, 882, 825; HRMS calcd for $C_{23}H_{17}$ [M+H]$^+$: 293.1325, found 293.1315.

2-(3-Methylphenyl)pyrene (5c)

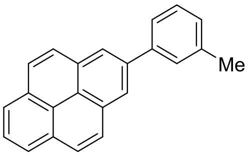

Prepared according to general procedure C using pyrene-1-carboxylic acid (50 mg, 0.203 mmol) and 3-iodotoluene (78 μL, 0.609 mmol). The crude product was purified by silica flash column chromatography (hexane/CH_2Cl_2, 100:0 to 98:2) to yield the title product as a bright yellow solid (30 mg, 51%), mp (CH_2Cl_2) 118-121 ºC. ^{1}H-NMR (400 MHz, $CDCl_3$) δ 2.53 (s, C*H*$_3$, 3 H), 7.26-7.30 (m, Ar*H*, 1 H), 7.47 (t, *J* = 7.6 Hz, Ar*H*, 1 H), 7.68-7.75 (m, Ar*H*, 2 H), 8.02 (t, *J* = 7.6 Hz, Ar*H*, 1 H), 8.11 (d, *J* = 8.8 Hz, Ar*H*, 2 H), 8.15 (d, *J* = 8.8 Hz, Ar*H*, 2 H), 8.20 (d, *J* = 7.6 Hz, Ar*H*, 2 H), 8.41 (s, Ar*H*, 2 H) ppm; ^{13}C-NMR (100 MHz, $CDCl_3$) δ 21.6 (*C*H$_3$), 123.7 (Ar*C*H), 123.8 (Ar*C*), 124.6 (Ar*C*), 125.1 (Ar*C*H), 125.2 (Ar*C*H), 125.9 (Ar*C*H), 127.5 (Ar*C*H), 127.7 (Ar*C*H), 128.2 (Ar*C*H), 128.9 (Ar*C*H), 128.9 (Ar*C*H), 131.1 (Ar*C*), 131.5 (Ar*C*), 138.6 (Ar*C*), 139.0 (Ar*C*), 141.5 (Ar*C*) ppm; IR ν_{max} (neat/cm^{-1}): 3038, 2923, 2852, 1601, 1491, 1442, 1178; HRMS calcd for $C_{23}H_{17}$ [M+H]$^+$: 293.1325, found 293.1316.

2-(4-*tert*-Butylphenyl)pyrene (5d)

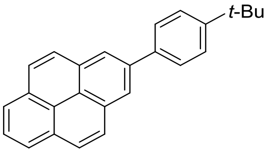

Prepared according to general procedure C using pyrene-1-carboxylic acid (50 mg, 0.203 mmol) and 4-*tert*-butyliodobenzene (110 μL, 0.609 mmol). The crude product was purified by silica flash column chromatography (hexane/CH_2Cl_2, 100:0 to 90:10) to yield the title product as a bright yellow solid (35 mg, 52%), mp (CH_2Cl_2) 188-190 ºC. ^{1}H-NMR (400 MHz, $CDCl_3$) δ 1.43 (s, C(C*H*$_3$)$_3$, 9 H), 7.60 (d, *J* = 8.4 Hz, Ar*H*, 2 H), 7.85 (d, *J* = 8.4 Hz, Ar*H*, 2 H), 8.01 (t, *J* = 7.4 Hz, Ar*H*, 1 H), 8.11 (d, *J* = 8.8 Hz, Ar*H*, 2 H), 8.14 (d, *J* = 8.8 Hz, Ar*H*, 2 H), 8.20 (d, *J* = 7.4 Hz, Ar*H*, 2 H), 8.41 (s, Ar*H*, 2 H) ppm; ^{13}C-NMR (125 MHz, $CDCl_3$) δ 31.4 (C(*C*H$_3$)$_3$), 34.6 (*C*(CH$_3$)$_3$), 123.6 (Ar*C*H), 123.8 (Ar*C*), 124.6 (Ar*C*), 125.1 (Ar*C*H), 125.8 (Ar*C*H), 126.0 (Ar*C*H), 127.5 (Ar*C*H), 127.7 (Ar*C*H), 127.7 (Ar*C*H), 131.1 (Ar*C*), 131.5 (Ar*C*), 138.6 (Ar*C*), 138.7 (Ar*C*), 150.5 (Ar*C*) ppm; IR ν_{max} (neat/cm^{-1}): 3039, 2962, 2904, 1600, 1511, 1464, 1370, 1360, 879, 831; HRMS calcd for $C_{26}H_{23}$ [M+H]$^+$: 335.1794, found 335.1790.

2-(4-methoxyphenyl)pyrene (5e)

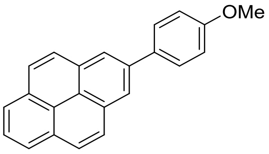

Prepared according to general procedure C using pyrene-1-carboxylic acid (50 mg, 0.203 mmol) and 4-iodoanisole (143 mg, 0.609 mmol). The crude product was purified by silica flash column chromatography (hexane/CH_2Cl_2, 100:0 to 88:12) to yield the title product as a yellow solid (30 mg, 48%), mp (CH_2Cl_2) 145-148 ºC. ^{1}H-NMR (400 MHz, $CDCl_3$) δ 3.92 (s, OC*H*$_3$, 3 H), 7.11 (d, *J* = 8.6 Hz, Ar*H*, 2 H), 7.84 (d, *J* = 8.6 Hz, Ar*H*, 2 H), 8.01 (t, *J* = 7.8 Hz, Ar*H*, 1 H), 8.07-8.15 (m, Ar*H*, 4 H), 8.19 (d, *J* = 7.8 Hz, Ar*H*, 2 H), 8.37 (s, Ar*H*, 2 H) ppm; ^{13}C-NMR (125 MHz, $CDCl_3$) δ 55.4 (O*C*H$_3$), 114.4 (Ar*C*H), 123.2 (Ar*C*H), 123.5 (Ar*C*), 124.6 (Ar*C*), 125.0 (Ar*C*H), 125.7 (Ar*C*H), 127.5 (Ar*C*H), 127.7 (Ar*C*H), 129.0 (Ar*C*H), 131.0 (Ar*C*), 131.5 (Ar*C*), 133.9 (Ar*C*), 138.5 (Ar*C*), 159.3 (Ar*C*) ppm; IR ν_{max} (neat/cm^{-1}): 3036, 3931, 1605, 1514, 1283, 1251, 1115; HRMS calcd for $C_{23}H_{17}O$ [M+H]$^+$: 309.1274, found 309.1267.

2-Phenylpyrene (5f)

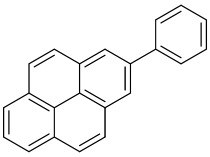

Prepared according to general procedure C using pyrene-1-carboxylic acid (50 mg, 0.203 mmol) and iodobenzene (68 μL, 0.609 mmol). The crude product was purified by silica flash column chromatography (hexane/CH_2Cl_2, 100:0 to 98:2) to yield the title product as a yellow solid (25 mg, 44%), mp (CH_2Cl_2) 158-162 ºC. ^{1}H-NMR (400 MHz, $CDCl_3$) δ 7.46 (t, *J* = 7.3 Hz, Ar*H*, 1 H), 7.58 (t, *J* = 7.3 Hz, Ar*H*, 2 H), 7.91 (d, *J* = 7.3 Hz, Ar*H*, 2 H), 8.03 (t, *J* = 7.8 Hz, Ar*H*, 1 H), 8.12 (d, *J* = 9.2 Hz, Ar*H*, 2 H), 8.15 (d, *J* = 9.2 Hz, Ar*H*, 2 H), 8.21 (d, *J* = 7.8 Hz, Ar*H*, 2 H), 8.42 (s, Ar*H*, 2 H) ppm; ^{13}C-NMR (100 MHz, $CDCl_3$) δ 123.7 (Ar*C*H), 123.9 (Ar*C*), 124.6 (Ar*C*), 125.1 (Ar*C*H), 125.9 (Ar*C*H), 127.4 (Ar*C*H), 127.5 (Ar*C*H), 127.8 (Ar*C*H), 128.1 (Ar*C*H), 129.0 (Ar*C*H), 131.1 (Ar*C*), 131.5 (Ar*C*), 138.9 (Ar*C*), 141.5 (Ar*C*) ppm; IR ν_{max} (neat/cm^{-1}): 3041, 2926, 1597, 1495, 1441, 1419, 881, 842, 823; HRMS calcd for $C_{22}H_{15}$ [M+H]$^+$: 279.1168, found 279.1159.

2-(4-Bromophenyl)pyrene (5g)

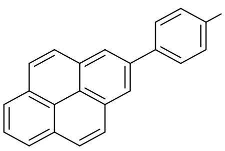

Prepared according to general procedure C using pyrene-1-carboxylic acid (50 mg, 0.203 mmol) and 4-bromoiodobenzene (172 mg, 0.609 mmol). The crude product was purified by silica flash column

chromatography (hexane/CH_2Cl_2, 100:0 to 98:2) to yield the title product as a bright yellow solid (46 mg, 63%), mp (CH_2Cl_2) 136-139 ºC. ^{1}H-NMR (400 MHz, $CDCl_3$) δ 7.69 (d, *J* = 8.4 Hz, Ar*H*, 2 H), 7.77 (d, *J* = 8.4 Hz, Ar*H*, 2 H), 8.03 (t, *J* = 7.6 Hz, Ar*H*, 1 H), 8.13 (s, Ar*H*, 4 H), 8.21 (d, *J* = 7.6 Hz, Ar*H*, 2 H), 8.36 (s, Ar*H*, 2 H) ppm; ^{13}C-NMR (100 MHz, $CDCl_3$) δ 121.8 (Ar*C*), 123.3 (Ar*C*H), 124.0 (Ar*C*), 124.5 (Ar*C*), 125.3 (Ar*C*H), 126.0 (Ar*C*H), 127.4 (Ar*C*H), 128.0 (Ar*C*H), 129.6 (Ar*C*H), 131.1 (Ar*C*), 131.6 (Ar*C*), 132.1 (Ar*C*H), 137.6 (Ar*C*), 140.5 (Ar*C*) ppm; IR ν_{max} (neat/cm^{-1}): 3040, 2922, 1602, 1491, 1440, 1179, 1074, 1008, 883, 841, 828; HRMS calcd for $C_{22}H_{14}Br$ $[M+H]^+$: 357.0273, found 357.0266.

2-(3-Bromophenyl)pyrene (5h)

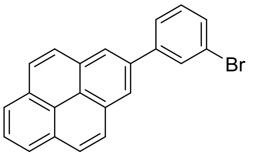

Prepared according to general procedure C using pyrene-1-carboxylic acid (50 mg, 0.203 mmol) and 3-bromoiodobenzene (78 μL, 0.609 mmol). The crude product was purified by silica flash column chromatography (hexane/CH_2Cl_2, 100:0 to 98:2) to yield the title product as a bright yellow solid (32 mg, 44%), mp (CH_2Cl_2) 133-136 ºC. ^{1}H-NMR (500 MHz, $CDCl_3$) δ 7.43 (t, *J* = 7.8 Hz, Ar*H*, 1 H), 7.58 (d, *J* = 7.8 Hz, Ar*H*, 1 H), 7.82 (d, *J* = 7.8 Hz, Ar*H*, 1 H), 8.01-8.07 (m, Ar*H*, 2 H), 8.09-8.16 (m, Ar*H*, 4 H), 8.21 (d, *J* = 7.8 Hz, Ar*H*, 2 H), 8.36 (s, Ar*H*, 2 H) ppm; ^{13}C-NMR (125 MHz, $CDCl_3$) δ 123.1 (Ar*C*), 123.5 (Ar*C*H), 124.1 (Ar*C*), 124.4 (Ar*C*), 125.3 (Ar*C*H), 126.1 (Ar*C*H), 126.6 (Ar*C*H), 127.4 (Ar*C*H), 128.0 (Ar*C*H), 130.3 (Ar*C*H), 130.5 (Ar*C*H), 131.0 (Ar*C*H), 131.1 (Ar*C*), 131.6 (Ar*C*), 137.3 (Ar*C*), 143.7 (Ar*C*) ppm; IR ν_{max} (neat/cm^{-1}): 3040, 2919, 1593, 1560, 1480, 1439, 871, 841; HRMS calcd for $C_{22}H_{14}Br$ $[M+H]^+$: 357.0273, found 357.0267.

2-(3-Chlorophenyl)pyrene (5i)

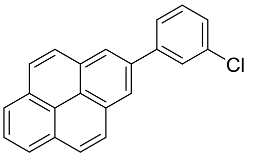

Prepared according to general procedure C using pyrene-1-carboxylic acid (50 mg, 0.203 mmol) and 3-chloroiodobenzene (75 μL, 0.609 mmol). The crude product was purified by silica flash column chromatography (hexane/CH_2Cl_2, 100:0 to 98:2) to yield the title product as a bright yellow solid (35 mg, 55%), mp (CH_2Cl_2) 116-119 ºC. ^{1}H-NMR (500 MHz, $CDCl_3$) δ 7.42 (ddd, *J* = 7.6, 2.0, 1.0 Hz, Ar*H*, 1 H), 7.49 (t, *J* = 7.6 Hz, Ar*H*, 1 H), 7.77 (d, *J* = 7.6 Hz, Ar*H*, 1 H), 7.89 (s, Ar*H*, 1 H), 8.03 (t, *J* = 7.3 Hz, Ar*H*, 1 H), 8.07-8.17 (m, Ar*H*, 4 H), 8.21 (d, *J* = 7.3 Hz, Ar*H*, 2 H), 8.37 (s, Ar*H*, 2 H) ppm; ^{13}C-NMR (125 MHz, $CDCl_3$) δ 123.5 (Ar*C*H), 124.1 (Ar*C*), 124.4 (Ar*C*), 125.3 (Ar*C*H), 126.1 (Ar*C*H), 126.2 (Ar*C*H), 127.4 (Ar*C*H), 127.4 (Ar*C*H), 128.0 (Ar*C*H), 128.1 (Ar*C*H), 130.2 (Ar*C*H), 131.1 (Ar*C*), 131.6 (Ar*C*), 134.8 (Ar*C*), 137.4 (Ar*C*), 143.4 (Ar*C*) ppm; IR ν_{max} (neat/cm^{-1}): 3040, 2923, 1594, 1565, 1440, 871, 841; HRMS calcd for $C_{22}H_{13}Cl$ $[M]^+$: 312.0700, found 312.0700.

2-(3,5-Dichlorophenyl)pyrene (5j)

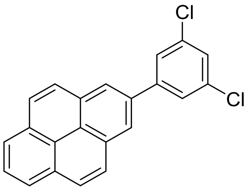

Prepared according to general procedure C using pyrene-1-carboxylic acid (50 mg, 0.203 mmol) and 3,5-dichloroiodobenzene (166 mg, 0.609 mmol). The crude product was purified by silica flash column chromatography (hexane) to yield the title product as a yellow solid (40 mg, 57%), mp (CH_2Cl_2) 200-203 ºC. ^{1}H-NMR (400 MHz, $CDCl_3$) δ 7.44 (bs, Ar*H*, 1 H), 7.77 (d, *J* = 1.2 Hz, Ar*H*, 2 H), 8.05 (t, *J* = 7.6 Hz, Ar*H*, 1 H), 8.10-8.18 (m, Ar*H*, 4 H), 8.23 (d, *J* = 7.6 Hz, Ar*H*, 2 H), 8.34 (s, Ar*H*, 2 H) ppm; ^{13}C-NMR (125 MHz, $CDCl_3$) δ 123.3 (Ar*C*H), 124.4 (Ar*C*), 124.4 (Ar*C*), 125.4 (Ar*C*H), 126.3 (Ar*C*H), 126.5 (Ar*C*H), 127.3 (Ar*C*H), 127.3 (Ar*C*H), 128.2 (Ar*C*H), 131.2 (Ar*C*), 131.7 (Ar*C*), 135.4 (Ar*C*), 136.0 (Ar*C*), 144.6 (Ar*C*) ppm; IR ν_{max} (neat/cm^{-1}): 3033, 1588, 1559, 1426, 1411, 849; HRMS calcd for $C_{22}H_{13}Cl_2$ $[M+H]^+$: 347.0389, found 347.0387.

2-(3,5-Bis(trifluoromethyl)phenyl)pyrene (5k)

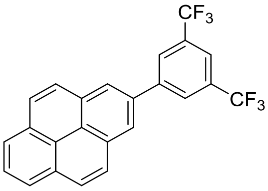

Prepared according to general procedure C using pyrene-1-carboxylic acid (50 mg, 0.203 mmol) and 3,5-bis(trifluoromethyl)iodobenzene (108 µL, 0.609 mmol). The crude product was purified by silica flash column chromatography (hexane) to yield the title product as an off-white solid (10 mg, 12%), mp (CH_2Cl_2) 210-213 ºC. ^{1}H-NMR (400 MHz, $CDCl_3$) δ 7.96 (bs, Ar*H*, 1 H), 8.07 (t, *J* = 8.0 Hz, Ar*H*, 1 H), 8.14-8.19 (m, Ar*H*, 4 H), 8.25 (d, *J* = 8.0 Hz, Ar*H*, 2 H), 8.32 (bs, Ar*H*, 2 H), 8.39 (s, Ar*H*, 2 H) ppm; ^{13}C-NMR (125 MHz, $CDCl_3$) δ 121.0 (hept, *J* = 3.9 Hz, Ar*C*H), 123.3 (Ar*C*H), 123.5 (q, *J* = 271.0 Hz, ArC*C*F$_3$), 124.3 (Ar*C*), 124.5 (Ar*C*), 125.6 (Ar*C*H), 126.4 (Ar*C*H), 127.2 (Ar*C*H), 128.1 (q, *J* = 2.6 Hz, Ar*C*H), 128.5 (Ar*C*H), 131.2 (Ar*C*), 131.8 (Ar*C*), 132.3 (q, *J* = 33.0 Hz, Ar*C*CF$_3$), 135.6 (Ar*C*), 143.7 (Ar*C*) ppm; ^{19}F-NMR (471 MHz, $CDCl_3$) –62.7 ppm; IR ν_{max} (neat/cm^{-1}): 2922, 1375, 1276, 1161, 1123; HRMS calcd for $C_{24}H_{13}F_6$ $[M+H]^+$: 415.0916, found 415.0910.

Alternatively, the product was prepared by decarboxylation of **3i** in the following manner: A microwave vial fitted with a Teflon-coated stirring bar was loaded with **3i** (20 mg, 43.6 µmol), Ag_2CO_3 (12 mg, 43.6 µmol) and dry DMSO (620 µL). The vial was sealed under air and the resulting mixture was stirred at 140 ˚C for 15 h. After cooling to room temperature the mixture was loaded into a silica column and eluted with hexane. The title compound was obtained as an off-white solid (12 mg, 66%). Analysis of the product showed the same spectroscopic data as the detailed above.

2-(3-nitrophenyl)pyrene (5l)

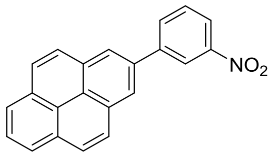

Prepared according to general procedure C using pyrene-1-carboxylic acid (50 mg, 0.203 mmol) and 3-iodonitrobenzene (152 mg, 0.609 mmol). The crude product was purified by silica flash column chromatography (hexane/CH_2Cl_2, 100:0 to 70:30) to yield the title product as a bright yellow solid (26 mg, 40%), mp (CH_2Cl_2) 222-224 ºC. ^{1}H-NMR (500 MHz, $CDCl_3$) δ 7.74 (t, *J* = 8.0 Hz, Ar*H*, 1 H), 8.06 (t, *J* = 7.5 Hz, Ar*H*, 1 H), 8.16 (s, Ar*H*, 4 H), 8.20-8.27 (m, Ar*H*, 3 H), 8.30 (d, *J* = 8.0 Hz, Ar*H*, 1 H), 8.42 (s, Ar*H*, 2 H), 8.77 (s, Ar*H*, 1 H) ppm; ^{13}C-NMR (125 MHz, $CDCl_3$) δ 122.1 (Ar*C*H), 122.8 (Ar*C*H), 123.4 (Ar*C*H), 124.3 (Ar*C*), 124.4 (Ar*C*), 125.5 (Ar*C*H), 126.3 (Ar*C*H), 127.3 (Ar*C*H), 128.3 (Ar*C*H), 129.9 (Ar*C*H), 131.2 (Ar*C*), 131.8 (Ar*C*), 133.9 (Ar*C*H), 136.1 (Ar*C*), 143.3 (Ar*C*), 148.9 (Ar*C*) ppm; IR ν_{max} (neat/cm^{-1}): 3043, 1525, 1346, 1311, 870; HRMS calcd for $C_{22}H_{14}O_2N$ $[M+H]^+$: 324.1019, found 324.1016.

2-(3-trifluoromethylphenyl)pyrene (5m)

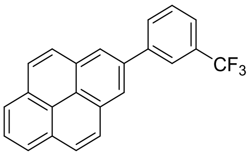

Prepared according to general procedure C using pyrene-1-carboxylic acid (50 mg, 0.203 mmol) and 3-trifluoromethyliodobenzene (88 μL, 0.609 mmol). The crude product was purified by silica flash column chromatography (hexane) to yield the title product as a yellow solid (29 mg, 41%), mp (CH_2Cl_2) 128-131 ºC. ^{1}H-NMR (400 MHz, $CDCl_3$) δ 7.64-7.76 (m, Ar*H*, 2 H), 8.00-8.07 (m, Ar*H*, 2 H), 8.08-8.18 (m, Ar*H*, 5 H), 8.21 (d, *J* = 7.8 Hz, Ar*H*, 2 H), 8.37 (s, Ar*H*, 2 H) ppm; ^{13}C-NMR (125 MHz, $CDCl_3$) δ 123.5 (Ar*C*H), 124.1 (q, *J* = 3.8 Hz, Ar*C*H), 124.2 (Ar*C*), 124.3 (q, *J* = 270.8 Hz, ArC*C*F$_3$), 124.4 (Ar*C*), 124.8 (q, *J* = 3.8 Hz, Ar*C*H), 125.3 (Ar*C*H), 126.2 (Ar*C*H), 127.4 (Ar*C*H), 128.1 (Ar*C*H), 129.4 (Ar*C*H), 131.1 (Ar*C*), 131.3 (q, *J* = 1.2 Hz, Ar*C*H), 131.4 (q, *J* = 32.0 Hz, Ar*C*CF$_3$), 131.7 (Ar*C*), 137.2 (Ar*C*), 142.3 (Ar*C*) ppm; ^{19}F-NMR (471 MHz, $CDCl_3$) –62.4 ppm; IR ν_{max} (neat/cm^{-1}): 3041, 1598, 1435, 1336, 1292, 1165, 1122; HRMS calcd for $C_{23}H_{14}F_3$ $[M+H]^+$: 347.1042, found 347.1037.

2-(3-Cyanophenyl)pyrene (5n)

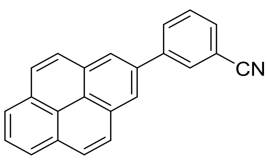

A microwave vial fitted with a Teflon-coated stirring bar was loaded with **3i** (20 mg, 57.6 μmol), Ag_2CO_3 (16 mg, 57.6 μmol) and dry DMSO (820 μL). The vial was sealed under air and the resulting

mixture was stirred at 140 ˚C for 15 h. After cooling to room temperature the mixture was loaded into a silica column and eluted with hexane/CH_2Cl_2 (80:20). The title compound was obtained as a pale yellow solid (9 mg, 52%), mp (CH_2Cl_2) 190-192 ºC. ^{1}H-NMR (500 MHz, $CDCl_3$) δ 7.66 (t, *J* = 7.7 Hz, Ar*H*, 1 H), 7.73 (d, *J* = 7.7 Hz, Ar*H*, 1 H), 8.05 (t, *J* = 7.5 Hz, Ar*H*, 1 H), 8.08-8.20 (m, Ar*H*, 6 H), 8.23 (d, *J* = 7.5 Hz, Ar*H*, 2 H), 8.35 (s, Ar*H*, 2 H) ppm; ^{13}C-NMR (125 MHz, $CDCl_3$) δ 113.2 (Ar*C*), 118.9 (Ar*C*N), 123.3 (Ar*C*H), 124.3 (Ar*C*), 124.3 (Ar*C*), 125.4 (Ar*C*H), 126.3 (Ar*C*H), 127.3 (Ar*C*H), 128.3 (Ar*C*H), 129.8 (Ar*C*H), 130.8 (Ar*C*H), 131.2 (Ar*C*), 131.5 (Ar*C*H), 131.7 (Ar*C*), 132.3 (Ar*C*H), 136.3 (Ar*C*), 142.8 (Ar*C*) ppm; IR ν_{max} (neat/cm^{-1}): 3042, 2921, 2228, 1599, 1483, 1445, 1414, 1178; HRMS calcd for $C_{23}H_{14}N$ [M+H]$^+$: 304.1121, found 304.1117.

Methyl 3-(pyren-2-yl)benzoate (5o)

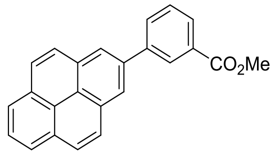

Prepared according to general procedure C using pyrene-1-carboxylic acid (50 mg, 0.203 mmol) and methyl 3-iodobenzoate (160 mg, 0.609 mmol). The crude product was purified by silica flash column chromatography (hexane/CH_2Cl_2, 100:0 to 60:40) to yield the title product as a yellow solid (38 mg, 56%), mp (CH_2Cl_2) 145-148 ºC. ^{1}H-NMR (400 MHz, $CDCl_3$) δ 4.02 (s, CO_2CH_3, 3 H), 7.62 (t, *J* = 7.6 Hz, Ar*H*, 1 H), 8.02 (t, *J* = 7.6 Hz, Ar*H*, 1 H), 8.05-8.15 (m, Ar*H*, 6 H), 8.19 (d, *J* = 7.6 Hz, Ar*H*, 2 H), 8.40 (s, Ar*H*, 2 H), 8.58 (bs, Ar*H*, 1 H) ppm; ^{13}C-NMR (125 MHz, $CDCl_3$) δ 52.3 (CO_2OCH_3), 123.5 (Ar*C*H), 124.0 (Ar*C*), 124.4 (Ar*C*), 125.2 (Ar*C*H), 126.0 (Ar*C*H), 127.4 (Ar*C*H), 127.9 (Ar*C*H), 128.4 (Ar*C*H), 129.0 (Ar*C*H), 129.0 (Ar*C*H), 130.8 (Ar*C*), 131.1 (Ar*C*), 131.5 (Ar*C*), 132.3 (ArCH), 137.5 (Ar*C*), 141.7 (Ar*C*), 167.1 (CO_2OCH_3) ppm; IR ν_{max} (neat/cm^{-1}): 3036, 2951, 1719, 1435, 1281, 1260; HRMS calcd for $C_{24}H_{16}O_2$ [M]$^+$: 336.1145, found 336.1142.

Methyl 4-(pyren-2-yl)benzoate (5p)

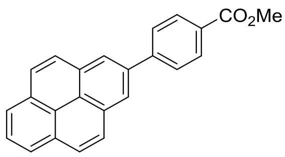

Prepared according to general procedure C using pyrene-1-carboxylic acid (50 mg, 0.203 mmol) and methyl 4-iodobenzoate (160 mg, 0.609 mmol). The crude product was purified by silica flash column chromatography (hexane/CH_2Cl_2, 100:0 to 60:40) to yield the title product as a bright yellow solid (42 mg, 62%), mp (CH_2Cl_2) 168-170 ºC. ^{1}H-NMR (500 MHz, $CDCl_3$) δ 4.00 (s, CO_2CH_3, 3 H), 7.98 (d, *J* = 8.5 Hz, Ar*H*, 2 H), 8.04 (t, *J* = 7.8 Hz, Ar*H*, 1 H), 8.13 (d, *J* = 9.0 Hz, Ar*H*, 2 H), 8.15 (d, *J* = 9.0 Hz, Ar*H*, 2 H), 8.19-8.27 (m, Ar*H*, 4 H), 8.43 (s, Ar*H*, 2 H) ppm; ^{13}C-NMR (125 MHz, $CDCl_3$) δ 52.2 (CO_2CH_3), 123.6 (Ar*C*H), 124.3 (Ar*C*), 124.4 (Ar*C*), 125.3 (Ar*C*H), 126.2 (Ar*C*H), 127.4 (Ar*C*H), 127.9 (Ar*C*H), 128.0 (Ar*C*H), 129.0 (Ar*C*), 130.3 (Ar*C*H), 131.2 (Ar*C*), 131.6 (Ar*C*), 137.5 (Ar*C*), 146.0 (Ar*C*), 167.1 (CO_2CH_3) ppm; IR ν_{max} (neat/cm^{-1}): 3041, 2950, 1722, 1608, 1434, 1285, 1275, 1112; HRMS calcd for $C_{24}H_{17}O_2$ [M+H]$^+$: 337.1223, found 337.1219.

General Procedure D: Ruthenium catalysed non-decarbolylative *ortho*-arylation of pyrene-1-carboxylic acid

2-(Thiophen-2-yl)pyrene-1-carboxylic acid (3q)

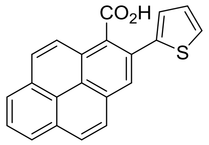

A microwave vial fitted with a Teflon-coated stirring bar was loaded with pyrene-1-carboxylic acid (50 mg, 0.203 mmol), 2-bromothiophene (24 μL, 0.254 mmol), $[Ru(p\text{-cymene})Cl_2]_2$ (5.0 mg, 0.008 mmol), PCy_3 (4.5 mg, 0.016 mmol), K_2CO_3 (28 mg, 0.203 mmol) and NMP (800 μL, 0.25 M) inside the glovebox. The vial was sealed and taken out of the glovebox. The reaction mixture was stirred at 100 ˚C for 24 h. After cooling to room temperature, the reaction mixture was quenched with 2 M aqueous HCl (100 μL). Internal standard dibromomethane (0.2 mmol) was added to the crude. Aliquot of approximately 50 μL was taken out by syringe. Each aliquot was diluted with DMSO-d6 (600 μL) and passed through a short plug of Celite into a NMR tube. The crude was monitored by quantitative ^{1}H NMR. The crude product was purified by silica flash column chromatography (hexane/EtOAc, 100:0 to 30:70) to yield the title product as an off white solid (30 mg, 45%), mp (CH_2Cl_2) 220-222 ºC. ^{1}H-NMR (500 MHz, DMSO-d_6) δ 7.24 (dd, *J* = 5.0, 3.5 Hz, Ar*H*, 1 H), 7.50 (d, *J* = 3.5 Hz, Ar*H*, 1 H), 7.74 (d, *J* = 5.0 Hz, Ar*H*, 1 H), 8.09 (d, *J* = 10.5 Hz, Ar*H*, 1 H), 8.12 (t, *J* = 7.8 Hz, Ar*H*, 1 H), 8.23-8.31 (m, Ar*H*, 3 H), 8.32-8.38 (m, Ar*H*, 2 H), 8.44 (s, Ar*H*, 1 H), 14.03 (bs, CO_2H, 1 H) ppm; ^{13}C-NMR (125 MHz, DMSO-d_6) δ 123.2 (Ar*C*), 123.8 (Ar*C*), 124.7 (Ar*C*H), 126.1 (Ar*C*H), 126.3 (Ar*C*H), 126.6 (Ar*C*H), 127.3 (Ar*C*H), 127.3 (Ar*C*), 127.5 (Ar*C*), 127.5 (Ar*C*H), 127.6 (Ar*C*H), 127.9 (Ar*C*H), 128.6 (Ar*C*H), 128.6 (Ar*C*), 129.0 (Ar*C*H), 129.5 (Ar*C*H), 130.6 (Ar*C*), 131.2 (Ar*C*), 131.3 (Ar*C*), 142.0 (Ar*C*), 171.1 (*C*O_2H) ppm; IR ν_{max} (neat/cm^{-1}): 3402, 2255, 1652, 1247, 1049, 1023, 997; HRMS calcd for $C_{21}H_{13}O_2S$ $[M+H]^+$: 329.0636, found 329.0641.

2-(5-Methylthiophen-2-yl)pyrene-1-carboxylic acid (3r)

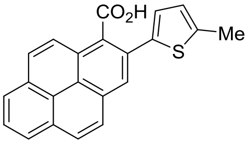

Prepared according to general procedure D using pyrene-1-carboxylic acid (50 mg, 0.203 mmol) and 2-iodo-5-methylthiophene (31 μL, 0.254 mmol). The crude product was purified by silica flash column chromatography (hexane/EtOAc, 100:0 to 60:40) to yield the title product as an orange solid (24 mg, 35%), mp (CH_2Cl_2) 236-238 ºC. ^{1}H-NMR (500 MHz, DMSO-d_6) δ 2.55 (s, C*H*$_3$, 3 H), 6.94 (d, *J* = 3.5 Hz, Ar*H*, 1 H), 7.27 (d, *J* = 3.5 Hz, Ar*H*, 1 H), 8.07-8.15 (m, Ar*H*, 2 H), 8.21-8.29 (m, Ar*H*, 2 H), 8.33 (d, *J* = 9.5 Hz, Ar*H*, 1 H), 8.34-8-38 (m, Ar*H*, 2 H), 8.39 (s, Ar*H*, 1 H), 13.79 (bs, CO_2H, 1 H), ppm; ^{13}C-NMR (125 MHz, DMSO-d_6) δ 15.4 (*C*H_3), 123.0 (Ar*C*), 123.8 (Ar*C*), 124.4 (Ar*C*H), 125.8 (Ar*C*H), 126.4 (Ar*C*H), 126.7 (Ar*C*H), 127.0 (Ar*C*H), 127.3 (Ar*C*H), 127.3 (Ar*C*), 127.4 (Ar*C*H), 127.5 (Ar*C*H), 129.0 (Ar*C*), 129.2 (Ar*C*H), 129.5 (Ar*C*), 129.7 (Ar*C*H), 130.5 (Ar*C*), 131.1 (Ar*C*), 131.5 (Ar*C*), 139.4 (Ar*C*), 141.5 (Ar*C*), 171.0 (*C*O_2H) ppm; IR ν_{max} (neat/cm^{-1}): 3407,

1732, 1652, 1374, 1243, 1045, 1024, 1003; HRMS calcd for $C_{22}H_{15}O_2S$ $[M+H]^+$: 343.0793, found 343.0798.

2-(1-Methylindol-5-yl)pyrene-1-carboxylic acid (3s)

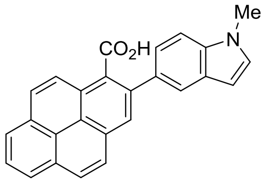

Prepared according to general procedure D using pyrene-1-carboxylic acid (50 mg, 0.203 mmol) and 5-bromo-1-methylindole (53 mg, 0.254 mmol). The crude product was purified by silica flash column chromatography (hexane/EtOAc, 100:0 to 40:60) to yield the title product as an orange solid (32 mg, 42%), mp (CH_2Cl_2) >220 ºC. ^{1}H-NMR (500 MHz, DMSO-d$_6$) δ 3.87 (s, C*H*$_3$, 3 H), 6.54 (d, *J* = 3.0 Hz, Ar*H*, 1 H), 7.43 (d, *J* = 3.0 Hz, Ar*H*, 1 H), 7.49 (d, *J* = 8.5 Hz, Ar*H*, 1 H), 7.59 (d, *J* = 8.5 Hz, Ar*H*, 1 H), 7.86 (s, Ar*H*, 1 H), 8.12 (t, *J* = 7.8 Hz, Ar*H*, 1 H), 8.20 (d, *J* = 9.5 Hz, Ar*H*, 1 H), 8.24-8.29 (m, Ar*H*, 2 H), 8.30-8.40 (m, Ar*H*, 4 H), 13.45 (bs, CO$_2$*H*, 1 H) ppm; ^{13}C-NMR (125 MHz, DMSO-d$_6$) δ 33.1 (*C*H$_3$), 101.2 (Ar*C*H), 110.2 (Ar*C*H), 121.3 (Ar*C*H), 122.6 (Ar*C*), 123.0 (Ar*C*H), 123.9 (Ar*C*), 124.6 (Ar*C*H), 126.1 (Ar*C*H), 126.4 (Ar*C*H), 127.0 (Ar*C*H), 127.0 (Ar*C*H), 127.3 (Ar*C*), 127.7 (Ar*C*H), 128.7 (Ar*C*), 128.8 (Ar*C*H), 129.2 (Ar*C*H), 130.4 (Ar*C*), 130.5 (Ar*C*), 130.9 (Ar*C*H), 131.1 (Ar*C*), 131.4 (Ar*C*), 132.1 (Ar*C*), 136.4 (Ar*C*), 138.2 (Ar*C*), 171.3 (*C*O$_2$H) ppm; IR ν_{max} (neat/cm^{-1}): 3402 2255, 2129, 1652, 1149, 1023, 996; HRMS calcd for $C_{26}H_{17}NO_2Na$ $[M+Na]^+$: 398.1157, found 398.1152.

2-(2,6-Dichloropyridin-4-yl)pyrene-1-carboxylic acid (3t)

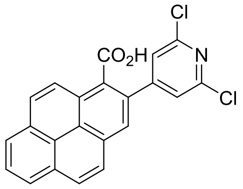

Prepared according to general procedure D using pyrene-1-carboxylic acid (50 mg, 0.203 mmol) and 2,6-dichloro-4-iodopyridine (70 mg, 0.254 mmol). The crude product was purified by silica flash column chromatography (hexane/EtOAc, 100:0 to 60:40) to yield the title product as an orange solid (46 mg, 58%), mp (CH_2Cl_2) >250 ºC. ^{1}H-NMR (500 MHz, DMSO-d$_6$) δ 7.83 (s, Ar*H*, 2 H), 8.16 (t, *J* = 7.8 Hz, Ar*H*, 1 H), 8.20-8.42 (m, Ar*H*, 6 H), 8.43 (s, Ar*H*, 1 H), 14.00 (bs, CO$_2$*H*, 1 H) ppm; ^{13}C-NMR (125 MHz, DMSO-d$_6$) δ 123.5 (Ar*C*), 123.9 (Ar*C*H), 124.0 (Ar*C*), 124.5 (Ar*C*H), 126.2 (Ar*C*H), 126.7 (Ar*C*H), 127.0 (Ar*C*H), 127.5 (Ar*C*H), 127.7 (Ar*C*H), 127.8 (Ar*C*), 129.0 (Ar*C*), 129.7 (Ar*C*H), 130.0 (Ar*C*H), 130.6 (Ar*C*), 131.3 (Ar*C*), 131.7 (Ar*C*), 131.8 (Ar*C*), 149.7 (Ar*C*), 155.6 (Ar*C*), 170.3 (*C*O$_2$H) ppm; IR ν_{max} (neat/cm^{-1}): 3394, 1651, 1023, 999; ESI-MS found for $C_{22}H_{13}O_2S$ $[M–H]^-$: 390.0 (100%), 392.1, 394.0.

General Procedure E: Sonogashira coupling of 2-aryl-1-iodopyrenes

2-(3,5-Dimethylphenyl)-1-(phenylethynyl)pyrene (6a)

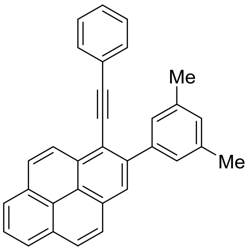

A flame dried Schlenk tube fitted with a Teflon coated stirring bar and loaded with **4a** (20 mg, 46.3 μmol), $PdCl_2(PPh_3)_2$ (6.5 mg, 9.26 μmol) and CuI (1.8 mg, 9.26 μmol) was evacuated and refilled with N_2. Then, degassed Et_3N (1.1 mL) and phenylacetylene (15 μL, 0.139 mmol) were added and the tube was sealed under N_2 before stirring it at room temperature. After 19 h the reaction mixture was diluted with CH_2Cl_2 and filtered through Celite. The organic layer was washed with saturated aqueous NH_4Cl (2 × 3 mL) and H_2O (2 × 3 mL), dried ($MgSO_4$) and concentrated under reduced pressure. The crude product was purified by silica flash column chromatography (hexane/EtOAc, 100:0 to 96.4) to yield the title product as a pale yellow solid (11 mg, 58%), mp (CH_2Cl_2) 129-132 ºC. ^{1}H-NMR (500 MHz, $CDCl_3$) δ 2.49 (s, Ar(C*H*$_3$)$_2$, 6 H), 7.15 (s, Ar*H*, 1 H), 7.32-7.41 (m, Ar*H*, 3 H), 7.49-7.56 (m, Ar*H*, 4 H), 8.04 (t, *J* = 7.5 Hz, Ar*H*, 1 H), 8.07 (d, *J* = 9.0 Hz, Ar*H*, 1 H), 8.12 (d, *J* = 9.0 Hz, Ar*H*, 1 H), 8.18-8.27 (m, Ar*H*, 4 H), 8.82 (d, *J* = 9.0 Hz, Ar*H*, 1 H) ppm; ^{13}C-NMR (125 MHz, $CDCl_3$) δ 21.5 (Ar(*C*H$_3$)$_2$), 88.5 (*C*≡C), 98.6 (C≡*C*), 116.3 (Ar*C*), 123.5 (Ar*C*), 123.8 (Ar*C*), 124.2 (Ar*C*), 125.6 (Ar*C*H), 125.6 (Ar*C*H), 125.8 (Ar*C*H), 126.1 (Ar*C*H), 126.2 (Ar*C*H), 127.3 (Ar*C*H), 128.1 (Ar*C*H), 128.2 (Ar*C*H), 128.3 (Ar*C*H), 128.4 (Ar*C*H), 128.6 (Ar*C*H), 129.1 (Ar*C*H), 130.9 (Ar*C*), 131.0 (Ar*C*), 131.2 (Ar*C*), 131.4 (Ar*C*H), 132.3 (Ar*C*), 137.3 (Ar*C*), 141.2 (Ar*C*), 142.3 (Ar*C*) ppm; IR ν_{max} (neat/cm^{-1}): 3037, 2919, 2857, 1597, 1490, 1442, 1179; HRMS calcd for $C_{32}H_{23}N$ $[M+H]^+$: 407.1794, found 407.1775.

2-(3,5-Dimethylphenyl)-1-(4-trifluoromethylphenylethynyl)pyrene (6b)

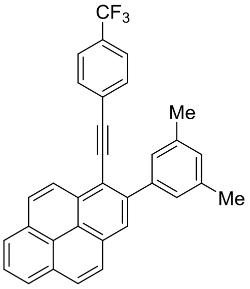

Prepared according to general procedure E using **4a** (20 mg, 46.3 μmol) and 4-(trifluoromethyl)phenylacetylene (23 μL, 0.139 mmol). The crude product was purified by silica flash column chromatography (hexane) to yield the title product as a bright yellow solid (11 mg, 50%), mp (CH_2Cl_2) 145-148 ºC. ^{1}H-NMR (400 MHz, $CDCl_3$) δ 2.48 (s, Ar(C*H*$_3$)$_2$, 6 H), 7.16 (s, Ar*H*, 1 H), 7.50 (s, Ar*H*, 2 H), 7.56 (d, *J* = 8.2 Hz, Ar*H*, 2 H), 7.63 (d, *J* = 8.2 Hz, Ar*H*, 2 H), 8.02-8.11 (m, Ar*H*, 2 H), 8.14 (d, *J* = 8.8 Hz, Ar*H*, 1 H), 8.19-8.31 (m, Ar*H*, 4 H), 8.77 (d, *J* = 8.8 Hz, Ar*H*, 1 H) ppm; ^{13}C-NMR (125 MHz, $CDCl_3$) δ 21.5 (*C*H$_3$), 91.1 (*C*≡C), 97.0 (C≡*C*), 115.5 (Ar*C*), 122.9 (Ar*C*), 124.0 (q,

J = 270.4 Hz, Ar*C*), 124.2 (Ar*C*), 125.3 (q, *J* = 3.8 Hz, Ar*C*H), 125.8 (Ar*C*H), 125.8 (Ar*C*H), 125.8 (Ar*C*H), 125.9 (Ar*C*H), 126.3 (Ar*C*H), 127.3 (Ar*C*H), 127.6 (q, *J* = 1.4 Hz, Ar*C*), 128.1 (Ar*C*H), 128.8 (Ar*C*H), 128.9 (Ar*C*H), 129.2 (Ar*C*H), 129.7 (q, *J* = 32.4 Hz, Ar*C*), 130.9 (Ar*C*), 131.2 (Ar*C*), 131.4 (Ar*C*), 131.5 (Ar*C*H), 132.5 (Ar*C*), 137.4 (Ar*C*), 141.1 (Ar*C*), 142.7 (Ar*C*) ppm; ^{19}F-NMR (376 MHz, $CDCl_3$) δ –62.7 ppm; IR ν_{max} (neat/cm^{-1}): 1312, 1322, 1166, 1125, 1066, 840; HRMS calcd for $C_{33}H_{22}F_3$ $[M+H]^+$: 475.1668, found 475.1649.

2-(3,5-Dimethylphenyl)-1-(trimethylsilylethynyl)pyrene (6c)

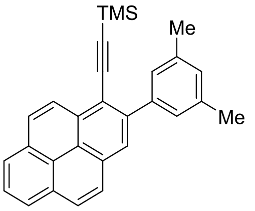

Prepared according to general procedure E using **4a** (20 mg, 46.3 μmol) and trimethylsilylacetylene (19 μL, 0.139 mmol). The crude product was purified by silica flash column chromatography (hexane) to yield the title product as a yellow solid (11 mg, 59%), mp (CH_2Cl_2) 128-131 °C. ^{1}H-NMR (500 MHz, $CDCl_3$) δ 0.28 (s, Si(C*H*$_3$)$_3$, 9 H), 2.46 (s, Ar(C*H*$_3$)$_2$, 6 H), 7.10 (s, Ar*H*, 1 H), 7.47 (s, Ar*H*, 2 H), 8.01-8.07 (m, Ar*H*, 2 H), 8.11 (d, *J* = 8.5 Hz, Ar*H*, 1 H), 8.17 (s, Ar*H*, 1 H), 8.19-8.23 (m, Ar*H*, 2 H), 8.24 (d, *J* = 7.5 Hz, Ar*H*, 1 H), 8.72 (d, *J* = 9.0 Hz, Ar*H*, 1 H) ppm; ^{13}C-NMR (125 MHz, $CDCl_3$) δ 0.0 (Si(*C*H$_3$)$_3$), 21.4 (Ar(C*H*$_3$)$_2$), 103.3 (*C*≡C), 104.0 (C≡*C*), 116.2 (Ar*C*), 123.3 (Ar*C*), 124.1 (Ar*C*), 125.6 (Ar*C*H), 125.7 (Ar*C*H), 125.8 (Ar*C*H), 126.1 (Ar*C*H), 126.2 (Ar*C*H), 127.3 (Ar*C*H), 128.0 (Ar*C*H), 128.5 (Ar*C*H), 128.6 (Ar*C*H), 129.0 (Ar*C*H), 130.9 (Ar*C*), 131.0 (Ar*C*), 131.1 (Ar*C*), 132.8 (Ar*C*), 137.2 (Ar*C*H), 141.0 (Ar*C*), 142.6 (Ar*C*) ppm; IR ν_{max} (neat/cm^{-1}): 3039, 2957, 2917, 2145, 1597, 1436, 1247, 1180, 879; HRMS calcd for $C_{29}H_{27}Si$ $[M+H]^+$: 403.1877, found 403.1875.

2-(4-Methylphenyl)-1-(phenylethynyl)pyrene (6d)

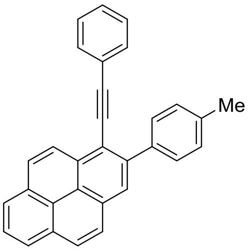

Prepared according to general procedure E using **4b** (30 mg, 71.7 μmol) and phenylacetylene (24 μL, 0.215 mmol). The crude product was purified by silica flash column chromatography (hexane/EtOAc, 100:0 to 96.4) to yield the title product as a yellow solid (21 mg, 75%), mp (CH_2Cl_2) 120-123 °C. ^{1}H-NMR (400 MHz, $CDCl_3$) δ 2.52 (s, C*H*$_3$, 3 H), 7.32-7.44 (m, Ar*H*, 5 H), 7.53 (dd, *J* = 7.8, 1.8 Hz, Ar*H*, 2 H), 7.81 (d, *J* = 8.0 Hz, Ar*H*, 2 H), 7.99-8.10 (m, Ar*H*, 2 H), 8.12 (d, *J* = 8.8 Hz, Ar*H*, 1 H), 8.18-8.29 (m, Ar*H*, 4 H), 8.82 (d, *J* = 9.2 Hz, Ar*H*, 1 H) ppm; ^{13}C-NMR (125 MHz, $CDCl_3$) δ 21.4 (*C*H$_3$), 88.5 (*C*≡C), 98.4 (C≡*C*), 116.4 (Ar*C*), 123.5 (Ar*C*), 123.8 (Ar*C*), 124.2 (Ar*C*), 125.6 (Ar*C*H),

125.7 (Ar*C*H), 125.9 (Ar*C*H), 126.1 (Ar*C*H), 126.2 (Ar*C*H), 127.3 (Ar*C*H), 128.2 (Ar*C*H), 128.4 (Ar*C*H), 128.4 (Ar*C*H), 128.6 (Ar*C*H), 128.7 (Ar*C*H), 130.1 (Ar*C*H), 131.0 (Ar*C*), 131.0 (Ar*C*), 131.2 (Ar*C*), 131.4 (Ar*C*H), 132.4 (Ar*C*), 137.2 (Ar*C*), 138.5 (Ar*C*), 142.0 (Ar*C*) ppm; IR ν_{max} (neat/cm^{-1}): 3040, 1512, 841, 827; HRMS calcd for $C_{31}H_{21}$ $[M+H]^+$: 393.1638, found 393.1634.

2-(4-Methylphenyl)-1-(trimethylsilylethynyl)pyrene (6e)

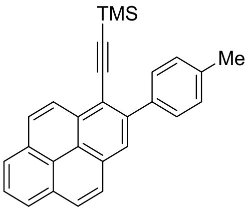

Prepared according to general procedure E using **4b** (30 mg, 71.7 μmol) and trimethylsilylacetylene (30 μL, 0.215 mmol). The crude product was purified by silica flash column chromatography (hexane) to yield the title product as a pale yellow solid (14 mg, 50%), mp (CH_2Cl_2) 134-138 °C. ^{1}H-NMR (400 MHz, $CDCl_3$) δ 0.26 (s, Si(C*H*$_3$)$_3$, 9 H), 2.48 (s, ArC*H*$_3$, 3 H), 7.32 (d, *J* = 7.8 Hz, Ar*H*, 2 H), 7.74 (d, *J* = 7.8 Hz, Ar*H*, 2 H), 8.00-8.05 (m, Ar*H*, 2 H), 8.10 (d, *J* = 9.0 Hz, Ar*H*, 1 H), 8.14-8.25 (m, Ar*H*, 4 H), 8.71 (d, *J* = 9.5 Hz, Ar*H*, 1 H) ppm; ^{13}C-NMR (125 MHz, $CDCl_3$) δ –0.1 (Si(*C*H$_3$)$_3$), 21.3 (Ar*C*H$_3$), 103.4 (*C*≡C), 104.2 (C≡*C*), 116.2 (Ar*C*), 123.3 (Ar*C*), 124.1 (Ar*C*), 125.6 (Ar*C*H), 125.7 (Ar*C*H), 125.7 (Ar*C*H), 126.1 (Ar*C*H), 126.2 (Ar*C*H), 127.3 (Ar*C*H), 128.5 (Ar*C*H), 128.5 (Ar*C*H), 128.6 (Ar*C*H), 130.1 (Ar*C*H), 130.9 (Ar*C*), 131.0 (Ar*C*), 131.1 (Ar*C*), 132.8 (Ar*C*), 137.1 (Ar*C*), 138.2 (Ar*C*), 142.3 (Ar*C*) ppm; IR ν_{max} (neat/cm^{-1}): 3040, 2956, 2919, 2144, 1596, 1581, 1512, 1434, 1248, 1184; HRMS calcd for $C_{28}H_{25}Si$ $[M+H]^+$: 389.1720, found 389.1715.

1-(4-Fluorophenylethynyl)-2-(4-methylphenyl)pyrene (6f)

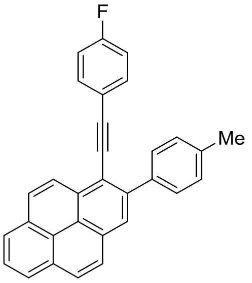

Prepared according to general procedure E using **4b** (209 mg, 0.50 mmol) and 1-ethynyl-4-fluorobenzene (172 μL, 1.50 mmol). The crude product was purified by silica flash column chromatography (hexane/EtOAc, 100:0 to 90:10) to yield the title product as a pale yellow solid (139 mg, 69%), mp (CH_2Cl_2) 160–163 °C. ^{1}H-NMR (500 MHz, $CDCl_3$) δ 2.52 (s, ArC*H*$_3$, 3 H), 7.08 (t, *J* = 8.0 Hz, Ar*H*, 2 H), 7.38 (d, *J* = 7.8 Hz, Ar*H*, 2 H), 7.49 (dd, *J* = 8.0, 6.0 Hz, Ar*H*, 2 H), 7.79 (d, *J* = 7.8 Hz, Ar*H*, 2 H), 8.01-8.09 (m, Ar*H*, 2 H), 8.12 (d, *J* = 9.0 Hz, Ar*H*, 1 H), 8.18-8.28 (m, Ar*H*, 4 H), 8.77 (d, *J* = 9.0 Hz, Ar*H*, 1 H) ppm; ^{13}C-NMR (125 MHz, $CDCl_3$) δ 21.3 (Ar*C*H$_3$), 88.2 (*C*≡C), 97.3 (C≡*C*), 115.7 (d, *J* = 21.9 Hz, Ar*C*H), 116.2 (Ar*C*), 119.9 (d, *J* = 3.5 Hz, Ar*C*), 123.5 (Ar*C*), 124.2 (Ar*C*), 125.7 (Ar*C*H), 125.7 (Ar*C*H), 125.8 (Ar*C*H), 126.0 (Ar*C*H), 126.2 (Ar*C*H), 127.3 (Ar*C*H),

128.5 (Ar*C*H), 128.6 (Ar*C*H), 128.7 (Ar*C*H), 130.1 (Ar*C*H), 130.9 (Ar*C*), 131.0 (Ar*C*), 131.2 (Ar*C*), 132.4 (Ar*C*), 133.2 (d, *J* = 8.3 Hz, Ar*C*H), 137.3 (Ar*C*), 138.5 (Ar*C*), 142.0 (Ar*C*), 162.5 (d, *J* = 248.0 Hz, Ar*C*F) ppm; ^{19}F-NMR (471 MHz, $CDCl_3$) δ –110.9 ppm; IR ν_{max} (neat/cm^{-1}): 3040, 1717, 1597, 1508, 1226, 1177, 1152, 821; HRMS calcd for $C_{31}H_{20}F$ [M+H]$^+$: 411.1544, found 411.1542.

2-(4-Methoxyphenyl)-1-(phenylethynyl)pyrene (6g)

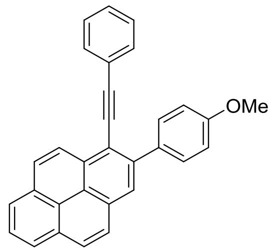

Prepared according to general procedure E using **4e** (217 mg, 0.50 mmol) and phenylacetylene (165 μL, 1.50 mmol). The crude product was purified by silica flash column chromatography (hexane) to yield the title product as a yellow solid (148 mg, 72%), mp (CH_2Cl_2) 158–161 °C. ^{1}H-NMR (500 MHz, $CDCl_3$) δ 3.95 (s, OC*H*$_3$, 3 H), 7.12 (d, *J* = 8.3 Hz, Ar*H*, 2 H), 7.32-7.44 (m, Ar*H*, 3 H), 7.55 (d, *J* = 7.0 Hz, Ar*H*, 2 H), 7.86 (d, *J* = 8.3 Hz, Ar*H*, 2 H), 7.99-8.07 (m, Ar*H*, 2 H), 8.10 (d, *J* = 9.0 Hz, Ar*H*, 1 H), 8.17-8.28 (m, Ar*H*, 4 H), 8.80 (d, *J* = 9.0 Hz, Ar*H*, 1 H) ppm; ^{13}C-NMR (125 MHz, $CDCl_3$) δ 55.4 (O*C*H$_3$), 88.5 (*C*≡C), 98.4 (C≡*C*), 113.4 (Ar*C*H), 116.3 (Ar*C*), 123.4 (Ar*C*), 123.8 (Ar*C*), 124.2 (Ar*C*), 125.6 (Ar*C*H), 125.7 (Ar*C*H), 125.7 (Ar*C*H), 126.0 (Ar*C*H), 126.1 (Ar*C*H), 127.3 (Ar*C*H), 128.2 (Ar*C*H), 128.4 (Ar*C*H), 128.4 (Ar*C*H), 128.6 (Ar*C*H), 130.9 (Ar*C*), 131.0 (Ar*C*), 131.2 (Ar*C*), 131.4 (Ar*C*H × 2), 132.4 (Ar*C*), 133.9 (Ar*C*), 141.6 (Ar*C*), 159.2 (Ar*C*) ppm; IR ν_{max} (neat/cm^{-1}): 1597, 1509, 1440, 1243, 1178, 1028, 894; HRMS calcd for $C_{31}H_{21}O$ [M+H]$^+$: 409.1587, found 409.1583.

General Procedure F: Suzuki coupling of 2-aryl-1-iodopyrenes

2-(3,5-Dimethylphenyl)-1-(4-ethoxycarbonylphenyl)pyrene (7a)

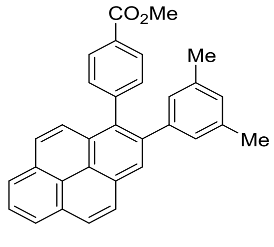

A flame dried Schlenk tube fitted with a Teflon coated stirring bar and loaded with **4a** (15 mg, 34.7 μmol), 4-(methoxycarbonyl)phenylboronic acid (7.5 mg, 41.6 μmol), $Pd(PPh_3)_4$ (4.8 mg, 4.16 μmol) and Na_2CO_3 (26 mg, 0.250 mmol) was evacuated and refilled with N_2. Then, degassed THF (5.0 mL) and degassed H_2O (1.2 mL) were added and the tube was sealed under N_2 before stirring it at 100 ˚C for 19 h. After cooling to room temperature, the reaction was quenched with saturated aqueous NH_4Cl and extracted with CH_2Cl_2 (3 × 3 mL). The organic layer was washed with brine, dried ($MgSO_4$) and concentrated under reduced pressure. The crude product was purified by silica flash column chromatography (hexane/CH_2Cl_2, 100:0 to 0:100) to yield the title product as a pale yellow solid (10

mg, 65%), mp (CH_2Cl_2) 190-193 ºC. ^{1}H-NMR (400 MHz, $CDCl_3$) δ 2.23 (s, Ar(C*H*$_3$)$_2$, 6 H), 3.97 (s, CO_2C*H*$_3$, 3 H), 6.86 (s, Ar*H*, 1 H), 6.88 (s, Ar*H*, 2 H), 7.41 (d, *J* = 8.0 Hz, Ar*H*, 2 H), 7.84 (d, *J* = 9.2 Hz, Ar*H*, 1 H), 7.98-8.09 (m, Ar*H*, 4 H), 8.11 (d, *J* = 9.2 Hz, Ar*H*, 1 H), 8.14 (d, *J* = 9.2 Hz, Ar*H*, 1 H), 8.18 (d, *J* = 7.2 Hz, Ar*H*, 1 H), 8.23 (d, *J* = 7.6 Hz, Ar*H*, 1 H), 8.25 (s, Ar*H*, 1 H) ppm; ^{13}C-NMR (100 MHz, $CDCl_3$) δ 21.2 Ar(*C*H$_3$)$_2$), 52.1 (CO_2*C*H$_3$), 123.9 (Ar*C*), 124.6 (Ar*C*), 125.1 (Ar*C*H), 125.4 (Ar*C*H), 125.5 (Ar*C*H), 126.2 (Ar*C*H), 126.6 (Ar*C*H), 127.3 (Ar*C*H), 127.8 (Ar*C*H), 128.0 (Ar*C*H), 128.1 (Ar*C*H), 128.4 (Ar*C*H), 128.4 (Ar*C*), 129.0 (Ar*C*H), 129.3 (Ar*C*), 130.7 (Ar*C*), 130.7 (Ar*C*), 131.3 (Ar*C*), 131.9 (Ar*C*H), 134.9 (Ar*C*), 137.1 (Ar*C*H), 139.4 (Ar*C*), 141.6 (Ar*C*), 144.9 (Ar*C*), 167.2 (*C*O$_2$CH$_3$) ppm; IR ν_{max} (neat/cm^{-1}): 3038, 2921, 1721, 1607, 1434, 1274, 1111, 1100; HRMS calcd for $C_{32}H_{25}O_2$ [M+H]$^+$: 441.1849, found 441.1830.

2-(3,5-Dimethylphenyl)-1-(4-methoxyphenyl)pyrene (7b)

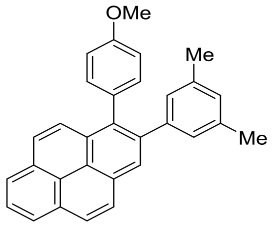

A flame dried Schlenk tube fitted with a Teflon coated stirring bar and loaded with **4a** (15 mg, 34.7 µmol), 4-(methoxy)phenylboronic acid (7.5 mg, 41.6 µmol), Pd(PPh$_3$)$_4$ (4.0 mg, 3.47 µmol) and K_2CO_3 (48 mg, 0.347 mmol) was evacuated and refilled with N_2. Then, degassed THF (1.7 mL), degassed EtOH (0.4 mL) and degassed H_2O (0.5 mL) were added and the tube was sealed under N_2 before stirring it at 100 ˚C for 14 h. After cooling to room temperature, the reaction was quenched with saturated aqueous NH_4Cl and extracted with CH_2Cl_2 (3 × 3 mL). The organic layer was washed with brine, dried ($MgSO_4$) and concentrated under reduced pressure. The crude product was purified by silica flash column chromatography (hexane/EtOAc, 100:0 to 99.5:0.5) to yield the title product as a pale yellow solid (10 mg, 70%), mp (CH_2Cl_2) 156-159 ºC. ^{1}H-NMR (400 MHz, $CDCl_3$) δ 2.25 (s, Ar(C*H*$_3$)$_2$, 6 H), 3.87 (s, OC*H*$_3$, 3 H), 6.84-6.96 (m, Ar*H*, 5 H), 7.23 (d, *J* = 8.4 Hz, Ar*H*, 2 H), 7.94-8.04 (m, Ar*H*, 3 H), 8.11 (s, Ar*H*, 2 H), 8.16 (d, *J* = 7.6 Hz, Ar*H*, 1 H), 8.20 (d, *J* = 7.6 Hz, Ar*H*, 1 H), 8.24 (s, Ar*H*, 1 H) ppm; ^{13}C-NMR (100 MHz, $CDCl_3$) δ 21.3 (Ar(C*H*$_3$)$_2$), 55.3 (O*C*H$_3$), 113.2 (Ar*C*H), 124.0 (Ar*C*), 124.7 (Ar*C*), 124.8 (Ar*C*H), 125.0 (Ar*C*H), 125.9 (Ar*C*H), 126.2 (Ar*C*H), 126.6 (Ar*C*H), 127.3 (Ar*C*H), 127.4 (Ar*C*H), 127.5 (Ar*C*H), 127.8 (Ar*C*H), 128.4 (Ar*C*H), 129.9 (Ar*C*), 130.3 (Ar*C*), 130.8 (Ar*C*), 131.4 (Ar*C*), 131.7 (Ar*C*), 132.8 (Ar*C*H), 135.9 (Ar*C*), 136.9 (Ar*C*), 139.8 (Ar*C*), 142.2 (Ar*C*), 158.4 (Ar*C*) ppm; IR ν_{max} (neat/cm^{-1}): 3035, 2914, 1607, 1515, 1437, 1291, 1243, 1175, 1036; HRMS calcd for $C_{31}H_{25}O$ [M+H]$^+$: 413.1900, found 413.1899.

1-(4-Methoxyphenyl)-2-(4-methylphenyl)pyrene (7c)

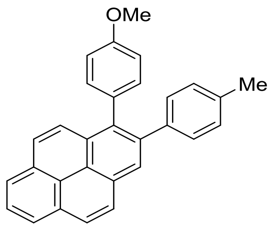

Prepared according to general procedure F using **4b** (102 mg, 0.243 mmol), 4-(methoxy)phenylboronic acid (44 mg, 0.292 mmol), $Pd(PPh_3)_4$ (28 mg, 24.3 μmol) and K_2CO_3 (336 mg, 2.43 mmol). The crude product was purified by silica flash column chromatography (hexane/EtOAc, 100:0 to 98.:2) to yield the title product as a yellow solid (78 mg, 81%), mp (CH_2Cl_2) 197–200 ºC. ^{1}H-NMR (500 MHz, $CDCl_3$) δ 2.35 (s, ArC*H*$_3$, 3 H), 3.88 (s, ArOC*H*$_3$, 3 H), 6.92 (d, *J* = 8.8 Hz, Ar*H*, 2 H), 7.08 (d, *J* = 7.8 Hz, Ar*H*, 2 H), 7.18 (d, *J* = 7.8 Hz, Ar*H*, 2 H), 7.22 (d, *J* = 8.8 Hz, Ar*H*, 2 H), 7.96 (d, *J* = 9.5 Hz, Ar*H*, 1 H), 7.98-8.04 (m, Ar*H*, 2 H), 8.08-8.12 (m, Ar*H*, 2 H), 8.16 (d, *J* = 7.0 Hz, Ar*H*, 1 H), 8.20 (d, *J* = 7.5 Hz, Ar*H*, 1 H), 8.24 (s, Ar*H*, 1 H), ppm; ^{13}C-NMR (125 MHz, $CDCl_3$) δ 21.1 (Ar*C*H$_3$), 55.2 (ArO*C*H$_3$), 113.3 (Ar*C*H), 123.9 (Ar*C*), 124.7 (Ar*C*), 124.8 (Ar*C*H), 125.0 (Ar*C*H), 125.9 (Ar*C*H), 126.1 (Ar*C*H), 126.7 (Ar*C*H), 127.4 (Ar*C*H), 127.4 (Ar*C*H), 127.6 (Ar*C*H), 128.4 (Ar*C*H), 129.9 (Ar*C*), 130.3 (Ar*C*H), 130.3 (Ar*C*), 130.8 (Ar*C*), 131.4 (Ar*C*), 131.7 (Ar*C*), 132.8 (Ar*C*H), 135.7 (Ar*C*), 135.9 (Ar*C*), 139.4 (Ar*C*), 139.5 (Ar*C*), 158.4 (Ar*C*) ppm; IR ν_{max} (neat/cm^{-1}): 3039, 2957, 2916, 2852, 1733, 1607, 1512, 1461, 1435, 1236, 1175; HRMS calcd for $C_{30}H_{23}O$ $[M+H]^+$: 399.1743, found 399.1742.

2-(6-(4-Methoxyphenyl)-7-(p-tolyl)pyren-2-yl)-4,4,5,5-tetramethyl-1,3,2-dioxaborolane (8)

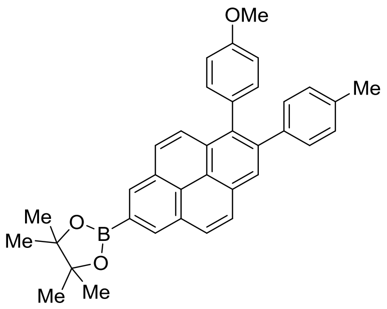

In an argon-filled glovebox, to a microwave vial charged with **7c** (50 mg, 0.125 mmol), bispinacolatodiboron (32 mg, 0.125), 4,4'-di-*tert*-butyl-2,2'-bipyridine (0.6 mg, 2.26 μmol) and [Ir(μ-MeOH)cod]$_2$ (0.75 mg, 1.13 μmol) was added cyclohexane (0.38 mL). The vial was sealed, taken out of the glovebox and the mixture was stirred at 80 ˚C for 22 h. After cooling to room temperature, the mixture was filtered through Celite with CH_2Cl_2 and volatiles were removed under reduced pressure. The crude product was purified by silica column chromatography (hexane/CH_2Cl_2, 100:0 to 0:100, then CH_2Cl_2/MeOH, 80:20) to yield the title product as a pale yellow solid (39 mg, 60%), mp (CH_2Cl_2) 133–135 ºC. ^{1}H-NMR (400 MHz, $CDCl_3$) δ 1.48 (s, ArB[OC(C*H*$_3$)$_2$]$_2$, 12 H), 2.35 (s, ArC*H*$_3$, 3 H), 3.87 (s, ArOC*H*$_3$, 3 H), 6.92 (d, *J* = 8.6 Hz, Ar*H*, 2 H), 7.07 (d, *J* = 8.0 Hz, Ar*H*, 2 H), 7.18 (d, *J* = 8.0 Hz, Ar*H*, 2 H), 7.22 (d, *J* = 8.6 Hz, Ar*H*, 2 H), 7.94 (d, *J* = 9.2 Hz, Ar*H*, 1 H), 8.02 (d, *J* = 9.2 Hz, Ar*H*, 1 H), 8.08 (d, *J* = 8.8 Hz, Ar*H*, 1 H), 8.13 (d, *J* = 8.8 Hz, Ar*H*, 1 H), 8.22 (s, Ar*H*, 1 H), 8.61 (s, Ar*H*, 1 H), 8.64 (s, Ar*H*, 1 H) ppm; ^{13}C-NMR (125 MHz, $CDCl_3$) δ 21.1 (Ar*C*H$_3$), 25.0 (ArB[OC(*C*H$_3$)$_2$]$_2$), 55.2 (ArO*C*H$_3$), 84.1 (ArB[O*C*(CH$_3$)$_2$]$_2$), 113.3 (Ar*C*H), 123.9 (Ar*C*), 126.0 (Ar*C*H), 126.4 (Ar*C*), 126.6 (Ar*C*H), 127.2 (Ar*C*H), 127.8 (Ar*C*H), 127.9 (Ar*C*H), 128.4 (Ar*C*H), 130.1 (Ar*C*), 130.3 (Ar*C*H), 130.4 (Ar*C*), 130.6 (Ar*C*), 130.8 (Ar*C*), 131.3 (Ar*C*H), 131.4 (Ar*C*H), 131.6 (Ar*C*), 132.8 (Ar*C*H), 135.8 (Ar*C*), 135.8 (Ar*C*), 139.4 (Ar*C*), 140.0 (Ar*C*), 158.4 (Ar*C*) ppm, one Ar*C* was not observed; IR ν_{max} (neat/cm^{-1}): 2976, 1514, 1463, 1415, 1356, 1235, 1140; HRMS calcd for $C_{36}H_{34}O_3B$ $[M+H]^+$: 525.2596, found 525.2595.

1-(Hydroxymethyl)-2-(*p*-tolyl)pyrene (9a)

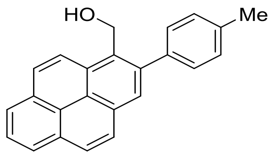

To a flame dried Schlenk tube charged with a solution of **3b** (50 mg, 0.149 mmol) in dry THF (1.5 mL) under N_2 was added dropwise $BH_3{\cdot}SMe_2$ (113 μL, 1.19 mmol). The tube was sealed under N_2 and the resulting mixture was stirred at 50 ˚C for 18 h. After cooling to room temperature the reaction was carefully quenchedby dropwise addition of H_2O (1.5 mL) and extracted with EtOAc (3 × 3 mL). The organic layer was washed with brine, dried ($MgSO_4$) and concentrated under reduced pressure. The crude product was loaded onto a short silica plug, washed with hexane/EtOAc (98:2) and collected with hexane/EtOAc (50:50). The title product was obtained as a pale orange solid (35 mg, 73%), mp (CH_2Cl_2) 170-173 ºC. ^{1}H-NMR (500 MHz, $CDCl_3$) δ 1.78 (bs, O*H*, 1 H), 2.50 (s, C*H*$_3$, 3 H), 5.27-5.33 (m, C*H*$_2$OH, 2 H), 7.35 (d, *J* = 7.3 Hz, Ar*H*, 2 H), 7.51 (d, *J* = 7.3 Hz, Ar*H*, 2 H), 8.01-8.07 (m, Ar*H*, 2 H), 8.08-8.15 (m, Ar*H*, 2 H), 8.18-8.28 (m, Ar*H*, 3 H), 8.57 (d, *J* = 9.5 Hz, Ar*H*, 1 H) ppm; ^{13}C-NMR (125 MHz, $CDCl_3$) δ 21.2 (*C*H$_3$), 59.8 (C*H*$_2$OH), 123.9 (Ar*C*H), 124.3 (Ar*C*), 124.6 (Ar*C*), 125.4 (Ar*C*H), 125.4 (Ar*C*H), 126.0 (Ar*C*H), 126.7 (Ar*C*H), 127.3 (Ar*C*H), 128.1 (Ar*C*H), 128.5 (Ar*C*H), 128.9 (Ar*C*H), 129.8 (Ar*C*H), 130.3 (Ar*C*), 130.6 (Ar*C*), 130.9 (Ar*C*), 131.0 (Ar*C*), 131.1 (Ar*C*), 137.1 (Ar*C*), 138.7 (Ar*C*), 140.7 (Ar*C*) ppm; IR ν_{max} (neat/cm^{-1}): 3347, 3031, 2919, 1599, 1511, 1437, 1180, 996, 839, 825; HRMS calcd for $C_{24}H_{18}OK$ $[M+K]^+$: 361.0989, found 361.0977.

Methyl 4-(1-(hydroxymethyl)pyren-2-yl)benzoate (9b)

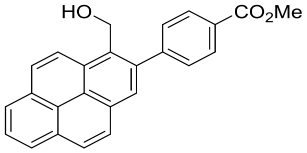

To a vial charged with **3p** (57 mg, 0.15 mmol) at 0 ˚C under N_2 was added thionyl chloride (220 μL, 3.0 mmol) and the resulting mixture was then stirred at 70 ˚C for 16 h. After cooling to room temperature the reaction mixture was diluted with toluene before removing volatiles under vaccum. The crude acid chloride was then dissolved in dry THF (1.1 mL) under N_2 before adding $NaBH_4$ (28 mg, 0.75 mmol) in one portion. The vial was sealed and the resulting mixture was stirred at 50 ˚C for 44 h. After cooling to 0˚C the reaction was carefully quenched with H_2O. The aqueous layer was extracted with EtOAc (3 × 10 mL) and the combined organic fractions were dried ($MgSO_4$) and concentrated under vaccum. The crude product was purified by silica column chromatography (pentane/EtOAc, 100:0 to 75:25) to yield the title product as a pale yellow solid (39 mg, 71%), mp (CH_2Cl_2) 164-168 ºC. ^{1}H-NMR (500 MHz, $CDCl_3$) δ 1.83 (t, *J* = 4.5 Hz, CH$_2$O*H*, 1 H), 4.01 (s, CO$_2$C*H*$_3$, 3 H), 5.27 (d, *J* = 4.5 Hz, C*H*$_2$OH, 2 H), 7.72 (d, *J* = 8.0 Hz, Ar*H*, 2 H), 8.02-8.10 (m, Ar*H*, 2 H), 8.11-8.17 (m, Ar*H*, 2 H), 8.19-8.29 (m, Ar*H*, 5 H), 8.57 (d, *J* = 9.0 Hz, Ar*H*, 1 H) ppm; ^{13}C-NMR (125 MHz, $CDCl_3$) δ 52.3 (CO$_2$*C*H$_3$), 59.7 (C*H*$_2$OH), 123.7 (Ar*C*H), 124.5 (Ar*C*), 124.6 (Ar*C*), 125.6 (Ar*C*H), 125.7 (Ar*C*H), 126.2 (Ar*C*H), 126.2 (Ar*C*H), 127.2 (Ar*C*H), 128.4 (Ar*C*H), 128.8 (Ar*C*H), 129.1 (Ar*C*), 129.5 (Ar*C*H), 130.1 (Ar*C*H), 130.4 (Ar*C*), 130.5 (Ar*C*), 130.6 (Ar*C*), 131.0

(Ar*C*), 131.2 (Ar*C*), 139.6 (Ar*C*), 146.5 (Ar*C*), 167.0 (*C*O_2CH_3) ppm; IR ν_{max} (neat/cm^{-1}): 3333, 2928, 1725, 1610, 1434, 1274, 1180, 1115; HRMS calcd for $C_{25}H_{17}O_3$ [M–H]$^-$: 365.1183, found 365.1184.

(2-(4-(Hydroxymethyl)phenyl)pyren-1-yl)methanol (9c)

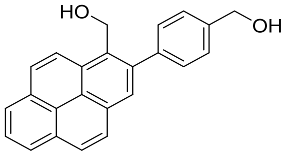

To a flame dried Schlenk tube charged with a solution of **3p** (50 mg, 0.131 mmol) in dry THF (1.3 mL) under N_2 was added dropwise BH_3·SMe_2 (100 μL, 1.05 mmol). The tube was sealed under N_2 and the resulting mixture was stirred at 55 ˚C for 18 h. After cooling to room temperature the reaction was quenched with H_2O (1.5 mL) and extracted with EtOAc (3 × 3 mL). The organic layer was washed with brine, dried ($MgSO_4$) and concentrated under reduced pressure. The crude product was purified by silica flash column chromatography (hexane/EtOAc, 50:50 to hexane/MeOH, 95:5) to yield the title product as a yellow solid (29 mg, 65%), mp (EtOAc) 210–212 ºC. ^{1}H-NMR (500 MHz, MeOD-d$_4$) δ 4.74 (s, C*H*$_2$OH, 2 H), 5.19 (s, C*H*$_2$OH, 2 H), 7.53 (d, *J* = 7.5 Hz, Ar*H*, 2 H), 7.64 (d, *J* = 7.5 Hz, Ar*H*, 2 H), 8.04 (t, *J* = 7.8 Hz, Ar*H*, 1 H), 8.06-8.15 (m, Ar*H*, 3 H), 8.20-8.29 (m, Ar*H*, 3 H), 8.62 (d, *J* = 9.0 Hz, Ar*H*, 1 H) ppm; ^{13}C-NMR (125 MHz, MeOD-d$_4$) δ 59.7 (C*H*$_2$OH), 65.1 (C*H*$_2$OH), 125.5 (Ar*C*), 125.6 (Ar*C*H), 125.7 (Ar*C*), 126.4 (Ar*C*H × 2), 127.2 (Ar*C*H), 127.5 (Ar*C*H), 127.8 (Ar*C*H), 128.4 (Ar*C*H), 129.0 (Ar*C*H), 129.1 (Ar*C*H), 131.2 (Ar*C*H), 132.0 (Ar*C*), 132.1 (Ar*C*), 132.3 (Ar*C*), 132.3 (Ar*C*), 132.6 (Ar*C*), 141.8 (Ar*C*), 142.1 (Ar*C*), 142.2 (Ar*C*) ppm; IR ν_{max} (neat/cm^{-1}): 3324, 2924, 2854, 1411, 1178, 994; HRMS calcd for $C_{24}H_{18}O_2Na$ [M+Na]$^+$: 361.1199, found 361.1199.

1-(Hydroxymethyl)-2-(3,5-bis(trifluoromethyl)phenyl)pyrene (9d)

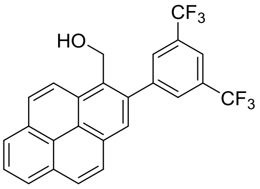

To a flame dried Schlenk tube charged with a solution of **3k** (46 mg, 0.100 mmol) in dry THF (1.0 mL) under N_2 was added dropwise BH_3·SMe_2 (76 μL, 0.800 mmol). The tube was sealed under N_2 and the resulting mixture was stirred at 50 ˚C for 18 h. After cooling to room temperature the reaction was carefully quenchedby dropwise addition of H_2O (1.5 mL) and extracted with EtOAc (3 × 3 mL). The organic layer was washed with brine, dried ($MgSO_4$) and concentrated under reduced pressure. The crude product was purified by silica column chromatography (hexane/EtOAc, 100:0 to 60:40). The title product was obtained as an off-white solid (31 mg, 70%), mp (CH_2Cl_2) >260 ºC. ^{1}H-NMR (500 MHz, acetone-d$_6$) δ 5.16-5.21 (m, ArC*H*$_2$OH, 2 H), 8.12 (t, *J* = 7.5 Hz, Ar*H*, 1 H), 8.19 (bs, Ar*H*, 1 H), 8.21 (d, *J* = 9.0 Hz, Ar*H*, 1 H), 8.24 (d, *J* = 9.0 Hz, Ar*H*, 1 H), 8.31-8.39 (m, Ar*H*, 4 H), 8.43 (bs, Ar*H*, 2 H), 8.71 (d, *J* = 9.5 Hz, Ar*H*, 1 H) ppm; ^{13}C-NMR (125 MHz, acetone-d$_6$) δ 59.2 and 59.3 (Ar*C*H$_2$OH), 121.9 (hept, *J* = 4.0 Hz, Ar*C*H), 124.6 (q, *J* = 270.4 Hz, Ar*C*F$_3$), 125.1 (Ar*C*), 125.5 (Ar*C*H), 125.5 (Ar*C*), 126.5 (Ar*C*H), 126.6 (Ar*C*H), 127.2 (Ar*C*H), 127.5 (Ar*C*H), 128.2 (Ar*C*H),

129.1 (Ar*C*H), 129.2 (Ar*C*H), 131.5-131.6 (m, Ar*C*H), 131.7 (Ar*C*), 131.8 (Ar*C*), 131.8 (q, *J* = 32.9 Hz, Ar*C*), 131.9 (Ar*C*), 132.3 (Ar*C*), 132.9 and 132.9 (Ar*C*), 138.5 (Ar*C*), 145.3 (Ar*C*) ppm; ^{19}F-NMR (471 MHz, acetone-d$_6$) –63.1 ppm; IR ν_{max} (neat/cm^{-1}): 3356, 2924, 2851, 1382, 1280, 1175, 1120; HRMS calcd for $C_{25}H_{14}OF_6$ $[M]^+$: 444.0943, found 444.0935.

N,N,N-Trimethyl-1-(2-(*p*-tolyl)pyren-1-yl)methanammonium bromide (10a)

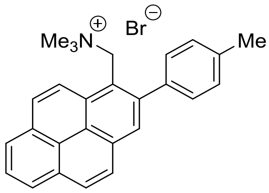

To a solution of **9a** (15 mg, 46.5 μmol), in dry THF (470 μL) at 0 ˚C under N_2 was added PBr_3 (2.2 μL, 23.3 μmol). The resulting mixture was allowed to slowly warm to room temperature. After 18 h the reaction was quenched with H_2O (0.5 mL) and extracted with EtOAc (3 × 3 mL). The organic layer was washed with brine, dried ($MgSO_4$) and concentrated under reduced pressure. The crude product was filtered through a short plug of silica (hexane/EtOAc, 90:10) to yield the benzylic bromide as a yellow solid, which was used whithout further purification. The bromide was then dissolved in THF (0.3 mL) and a 4.2 M solution of trimethylamine in EtOH (0.3 mL, 1.26 mmol) was added. The vial was sealed and the mixture stirred at 55 ˚C for 3 h. After cooling to room temperature, volatiles were removed under vaccum and the crude product was triturated in THF/Et_2O. The resulting solid was filtered under vaccum and washed with Et_2O. The solid was dried and the title product was obtained as a pale yellow solid (10 mg, 48% over 2 steps), mp (Et_2O) 151-154 ºC. ^{1}H-NMR (500 MHz, MeOD-d$_4$) δ 2.50 (s, ArC*H*$_3$, 3 H), 2.87 (s, ArCH$_2$N(C*H*$_3$)$_3$, 9 H), 5.50 (d, *J* = 14.5 Hz, ArC*H*$_a$H$_b$N, 1 H), 5.70 (d, *J* = 14.5 Hz, ArCH$_a$*H*$_b$N, 1 H), 7.25-7.76 (br, ArC$_6$*H*$_4$CH$_3$, 4 H), 8.15 (t, *J* = 7.8 Hz, Ar*H*, 1 H), 8.20 (d, *J* = 9.0 Hz, Ar*H*, 1 H), 8.27-8.34 (m, Ar*H*, 2 H), 8.35-8.45 (m, Ar*H*, 3 H), 8.71 (d, *J* = 9.5 Hz, Ar*H*, 1 H) ppm; ^{13}C-NMR (125 MHz, MeOD-d$_4$) δ 21.3 (Ar*C*H$_3$), 54.0 (t, *J* = 3.6 Hz, ArCH$_2$N(*C*H$_3$)$_3$), 63.0 (t, *J* = 1.9 Hz, Ar*C*H$_2$N(CH$_3$)$_3$), 119.1 (Ar*C*), 124.6 (Ar*C*H), 125.3 (Ar*C*), 125.4 (Ar*C*), 127.5 (Ar*C*H), 128.0 (Ar*C*H), 128.0 (Ar*C*H), 128.3 (Ar*C*H), 129.4 (Ar*C*H), 131.0 (Ar*C*H), 131.1 (Ar*C*H), 131.1 (br, Ar*C*H), 131.5 (Ar*C*), 131.7 (br, Ar*C*H), 132.6 (Ar*C*), 134.3 (Ar*C*), 134.5 (Ar*C*), 139.4 (Ar*C*), 140.0 (Ar*C*), 145.3 (Ar*C*) ppm; IR ν_{max} (neat/cm^{-1}): 3407, 3012, 2924, 2853, 1596, 1485, 1184, 970, 873, 846, 831; HRMS calcd for $C_{27}H_{26}N$ $[M]^+$: 364.2060, found 364.2054.

1-(2-(4-(Methoxycarbonyl)phenyl)pyren-1-yl)-*N,N,N*-trimethylmethanammonium bromide (10b)

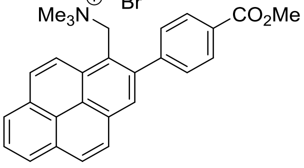

To a solution of **9b** (35 mg, 95.5 μmol), in dry CH_2Cl_2 (1.0 mL) at 0 ˚C under N_2 was added PBr_3 (4.5 μL, 47.8 μmol). The resulting mixture was allowed to slowly warm to room temperature. After 5

h the reaction was quenched with H_2O (0.5 mL) and extracted with EtOAc (3 × 3 mL). The organic layer was washed with brine, dried ($MgSO_4$) and concentrated under reduced pressure. The crude product was filtered through a short plug of silica (hexane/EtOAc, 50:50) to yield the benzylic bromide as a yellow solid, which was used whithout further purification. The bromide was then dissolved in THF (0.6 mL) and a 4.2 M solution of trimethylamine in EtOH (0.6 mL, 2.52 mmol) was added. The vial was sealed under air and the mixture stirred at 55 ˚C for 17 h. After cooling to room temperature, the solid residue was re-dissolved in as little MeOH as possible and Et_2O was added to precipitate the product. The precipitate was filtered amd washed with Et_2O. The solid was dried and the title product was obtained as a pale brown solid (37 mg, 79% over 2 steps), mp (Et_2O), decomposes above 160 ºC. ^{1}H-NMR (400 MHz, MeOD-d$_4$) δ 2.88 (s, $CH_2N(CH_3)_3$, 9 H), 3.99 (s, CO_2CH_3, 3 H), 5.38 (d, J = 14.4 Hz, $CH_aH_bN(CH_3)_3$, 1 H), 5.76 (d, J = 14.4 Hz, $CH_aH_bN(CH_3)_3$, 1 H), 7.57-8.02 (m, Ar*H*, 2 H), 8.12-8.49 (m, Ar*H*, 9 H), 8.75 (d, J = 9.6 Hz, Ar*H*, 1 H) ppm; ^{13}C-NMR (125 MHz, MeOD-d$_4$) δ 52.9 (CO_2CH_3), 54.0 ($CH_2N(CH_3)_3$), 62.9 ($CH_2N(CH_3)_3$), 118.7 (Ar*C*), 124.6 (Ar*C*H), 125.2 (Ar*C*), 125.6 (Ar*C*), 127.7 (Ar*C*H), 128.1 (Ar*C*H), 128.2 (Ar*C*H), 128.2 (Ar*C*H), 129.1 (Ar*C*H), 131.0 (Ar*C*), 131.2 (Ar*C*H), 131.3 (Ar*C*H), 131.6 (Ar*C*), 131.8-132.4 (Ar*C*H × 2), 132.6 (Ar*C*), 134.5 (Ar*C*), 134.5 (Ar*C*), 144.1 (Ar*C*), 147.6 (Ar*C*), 168.0 (CO_2CH_3) ppm; IR ν_{max} (neat/cm^{-1}): 3424, 3016, 2950, 1717, 1608, 1598, 1485, 1434, 1286, 1182, 1104; HRMS calcd for $C_{28}H_{26}O_2N$ $[M]^+$: 408.1958, found 408.1946.

***N,N,N*-trimethyl-1-(2-(4-((trimethylammonio)methyl)phenyl)pyren-1-yl)methanammonium bromide (10c)**

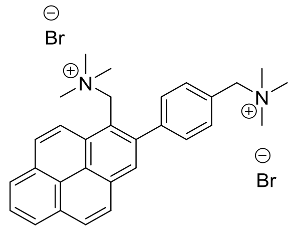

To a solution of **9c** (23 mg, 68.0 μmol), in dry CH_2Cl_2 (680 μL) at 0 ˚C under N_2 was added PBr_3 (6.8 μL, 68.0 μmol). The resulting mixture was allowed to slowly warm to room temperature. After 5 h the reaction was quenched with H_2O (0.5 mL) and extracted with EtOAc (3 × 3 mL). The organic layer was washed with brine, dried ($MgSO_4$) and concentrated under reduced pressure. The crude product was filtered through a short plug of silica (hexane/CH_2Cl_2, 50:50) to yield the dibromide as a pale yellow solid, which was used whithout further purification. The dibromide was then dissolved in THF (1.0 mL) and a 4.2 M solution of trimethylamine in EtOH (1.0 mL, 4.20 mmol) was added. The vial was sealed and the mixture stirred at 55 ˚C for 6 h. After cooling to room temperature, volatiles were removed under vaccum and the crude product was triturated in THF. The resulting solid was filtered under vaccum and washed with THF. The solid was dried and the title product was obtained as a yellow solid (16 mg, 40% over 2 steps), mp (MeOH), decomposes above 190 ºC. ^{1}H-NMR (400 MHz, MeOD-d$_4$) δ 2.92 (s, $ArCH_aH_bN(CH_3)_3$, 9 H), 3.26 (s, $ArCH_2N(CH_3)_3$, 9 H), 4.72 (s, $ArCH_2N$, 2 H), 5.42 (d, J = 14.2 Hz, $ArCH_aH_bN$, 1 H), 5.79 (d, J = 14.2 Hz, $ArCH_aH_bN$, 1 H), 7.71-7.91 (br, Ar*H*, 4 H), 8.18 (t, J = 7.6 Hz, Ar*H*, 1 H), 8.23 (d, J = 8.8 Hz, Ar*H*, 1 H), 8.31-8.37 (m, Ar*H*, 2 H), 8.39-8.44 (m, Ar*H*, 2 H), 8.46 (d, J = 9.0 Hz, Ar*H*, 1 H), 8.77 (d, J = 9.0 Hz, Ar*H*, 1 H) ppm; ^{13}C-NMR (125 MHz, MeOD-d$_4$) δ 53.4 (J = 3.9 Hz, $ArCH_2N(CH_3)_3$), 54.1 ($ArCH_aH_bN(CH_3)_3$), 63.0 ($ArCH_aH_bN$), 70.0 ($ArCH_2N$), 118.8 (Ar*C*), 124.7 (Ar*C*H), 125.2 (Ar*C*), 125.6 (Ar*C*), 127.7 (Ar*C*H), 128.1 (Ar*C*H), 128.2 (Ar*C*H), 128.2 (Ar*C*H), 129.0 (Ar*C*), 129.2 (Ar*C*H), 131.2 (Ar*C*H), 131.3

(Ar*C*H), 131.6 (Ar*C*), 132.6 (Ar*C*), 132.7 (Ar*C*H), 134.5 (Ar*C*), 134.5 (Ar*C*), 135.2 (Ar*C*H), 144.0 (Ar*C*), 145.4 (Ar*C*) ppm; IR ν_{max} (neat/cm^{-1}): 3393, 3007, 2922, 1596, 1476, 1414, 1377, 970, 892, 864, 845; HRMS calcd for $C_{30}H_{34}N_2$ $[M]^{2+}$: 211.1356, found 211.1348.

1-(2-(3,5-Bis(trifluoromethyl)phenyl)pyren-1-yl)-*N*,*N*,*N*-trimethylmethanammonium bromide (10d)

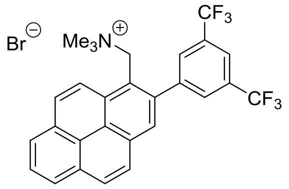

To a solution of **9d** (30 mg, 67.5 μmol), in dry CH_2Cl_2 (680 μL) at 0 ˚C under N_2 was added PBr_3 (3.0 μL, 33.8 μmol). The resulting mixture was allowed to slowly warm to room temperature. After 13 h the reaction was quenched with H_2O (0.5 mL) and extracted with EtOAc (3 × 3 mL). The organic layer was washed with brine, dried ($MgSO_4$) and concentrated under reduced pressure. The crude product was purified by silica column chromatography (hexane/EtOAc, 100:0 to 80:20) to yield the benzylic bromide as a pale yellow solid. The benzylic bromide was then dissolved in THF (0.5 mL) and a 4.2 M solution of trimethylamine in EtOH (245 μL, 1.03 mmol) was added. The vial was sealed and the mixture stirred at 55 ˚C for 5 h. After cooling to room temperature, volatiles were removed under vaccum and the crude product was triturated in Et_2O. The resulting solid was allowed to settle, the supernatant was removed and the solid dried under vacuum. The title product was obtained as an off-white solid (19 mg, 50% over 2 steps), mp (Et_2O) 145-148 ºC. ^{1}H-NMR (400 MHz, MeOD-d_4) δ 2.93 (s, $CH_2N(CH_3)_3$, 9 H), 5.11 (d, *J* = 14.4 Hz, $CH_aH_bN(CH_3)_3$, 1 H), 5.86 (d, *J* = 14.4 Hz, $CH_aH_bN(CH_3)_3$, 1 H), 8.12-8.27 (m, Ar*H*, 4 H), 8.29-8.36 (m, Ar*H*, 2 H), 8.37-8.44 (m, Ar*H*, 2 H), 8.46 (d, *J* = 9.6 Hz, Ar*H*, 1 H), 8.53 (bs, Ar*H*, 1 H), 8.80 (d, *J* = 9.6 Hz, Ar*H*, 1 H) ppm; ^{13}C-NMR (125 MHz, MeOD-d_4) δ 53.9 ($ArCH_2N(CH_3)_3$), 63.2 ($ArCH_2N(CH_3)_3$), 118.8 (Ar*C*), 123.1 (hept, *J* = 3.9 Hz, Ar*C*H), 124.8 (q, *J* = 269.9 Hz, $ArCF_3$), 124.8 (Ar*C*H), 125.1 (Ar*C*), 125.9 (Ar*C*), 127.8 (Ar*C*H), 128.2 (Ar*C*H), 128.3 (Ar*C*H), 128.4 (Ar*C*H), 129.4 (Ar*C*H), 131.3 (Ar*C*H), 131.5 (Ar*C*H), 131.7 (Ar*C*), 132.4 (bs, Ar*C*H), 132.7 (Ar*C*), 133.3 (q, *J* = 30.8 Hz, Ar*C*), 134.6 (Ar*C*), 142.0 (Ar*C*), 145.4 (Ar*C*) ppm, one Ar*C* was not observed; ^{19}F-NMR (376 MHz, MeOD-d_4) –64.1 ppm; IR ν_{max} (neat/cm^{-1}): 3409, 3012, 1486, 1376, 1278, 1177, 1135; HRMS calcd for $C_{28}H_{22}NF_6$ $[M]^+$: 486.1651, found 486.1644.

1-(Bromomethyl)pyrene (S1)

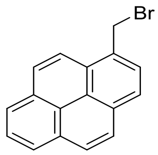

To a suspension of 1-pyrenemethanol (5.00 g, 21.5 mmol) in dry toluene (250 mL) at 0 ˚C, PBr_3 (1.0 mL, 10.8 mmol) was added dropwise. The resulting mixture was stirred at 0 ˚C for 1.5 h and then at room temperature for 1 h. The reaction was quenched by careful addition of saturated aqueous Na_2CO_3 (25 mL). Layers were separated and the organic fraction was washed with H_2O (2 × 12 mL)

and brine (2 × 12 mL). The organic layer was concentrated to obtain the title product as an off-white solid (6.21 g, 98%), mp (toluene): decomposes above 125 ºC. ^{1}H-NMR (400 MHz, $CDCl_3$) δ 5.28 (s, C*H*$_2$, 2 H), 8.02-8.16 (m, Ar*H*, 5 H), 8.21-8.30 (m, Ar*H*, 3 H), 8.41 (d, *J* = 9.2 Hz, Ar*H*, 1 H) ppm; ^{13}C-NMR (125 MHz, $CDCl_3$) δ 32.2 (*C*H$_2$), 122.8 (Ar*C*H), 124.6 (Ar*C*), 124.9 (Ar*C*H), 125.1 (Ar*C*), 125.6 (Ar*C*H), 125.6 (Ar*C*H), 126.3 (Ar*C*H), 127.3 (Ar*C*H), 127.7 (Ar*C*H), 128.0 (Ar*C*H), 128.2 (Ar*C*H), 129.1 (Ar*C*), 130.6 (Ar*C*), 130.8 (Ar*C*), 131.2 (Ar*C*), 132.0 (Ar*C*) ppm; IR ν_{max} (neat/cm^{-1}): 3037, 1917, 1587, 1418, 1311, 1201, 1184, 1084, 840; HRMS calcd for $C_{17}H_{12}Br$ $[M]^+$: 295.0117, found 295.0112.

_N,N,N_-Trimethyl-_N_-(1-pyrenylmethyl)ammonium bromide (11)

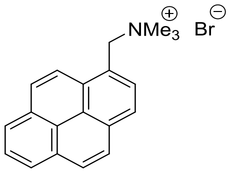

A solution of trimethylamine in ethanol (4.2 M, 14.7 mL, 62.0 mmol) was added to a flask charged with **S1** (1.00 g, 3.39 mmol). The flask was sealed under air and the mixture stirred at 60 ºC for 18 h. After cooling to room temperature volatiles were removed under vacuum. The resulting solid was triturated in Et_2O, filtered and washed with more Et_2O. The title product was obtained as an off-white solid (1.25 mg, 98%), mp (MeOH): decomposes above 170 ºC. ^{1}H-NMR (500 MHz, MeOD-d$_4$) δ 3.24 (s, N(C*H*$_3$)$_3$, 9 H), 5.36 (s, C*H*$_2$, 2 H), 8.13 (t, *J* = 7.8 Hz, Ar*H*, 1 H), 8.19 (d, *J* = 9.0 Hz, Ar*H*, 1 H), 8.22-8.29 (m, Ar*H*, 2 H), , 8.31-8.40 (m, Ar*H*, 4 H), 8.60 (d, *J* = 9.5 Hz, Ar*H*, 1 H) ppm; ^{13}C-NMR (125 MHz, MeOD-d$_4$) δ 53.6 (t, *J* = 4.0 Hz, N(*C*H$_3$)$_3$), 67.0 (t, *J* = 2.4 Hz, Ar*C*H$_2$N), 121.9 (Ar*C*), 123.6 (Ar*C*H), 125.5 (Ar*C*), 125.9 (Ar*C*H), 126.2 (Ar*C*), 127.3 (Ar*C*H), 127.7 (Ar*C*H), 127.9 (Ar*C*H), 128.3 (Ar*C*H), 130.5 (Ar*C*H), 1230.8 (Ar*C*H), 131.7 (Ar*C*), 132.6 (Ar*C*), 133.1 (Ar*C*H), 133.2 (Ar*C*), 134.8 (Ar*C*) ppm

4. NMR spectra

2-(3,5-Dimethylphenyl)pyrene-1-carboxylic acid (3a)

^{1}H-NMR (400 MHz, DMSO-d_6)

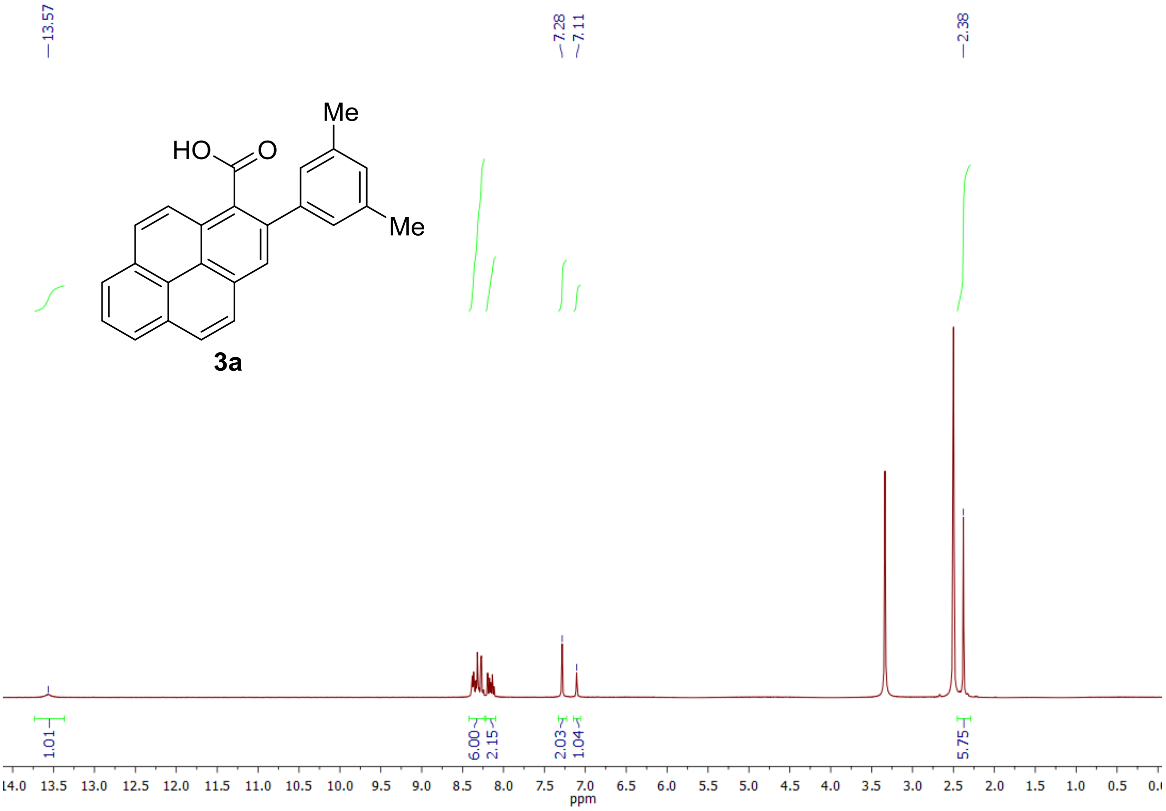

^{13}C-NMR (125 MHz, DMSO-d_6)

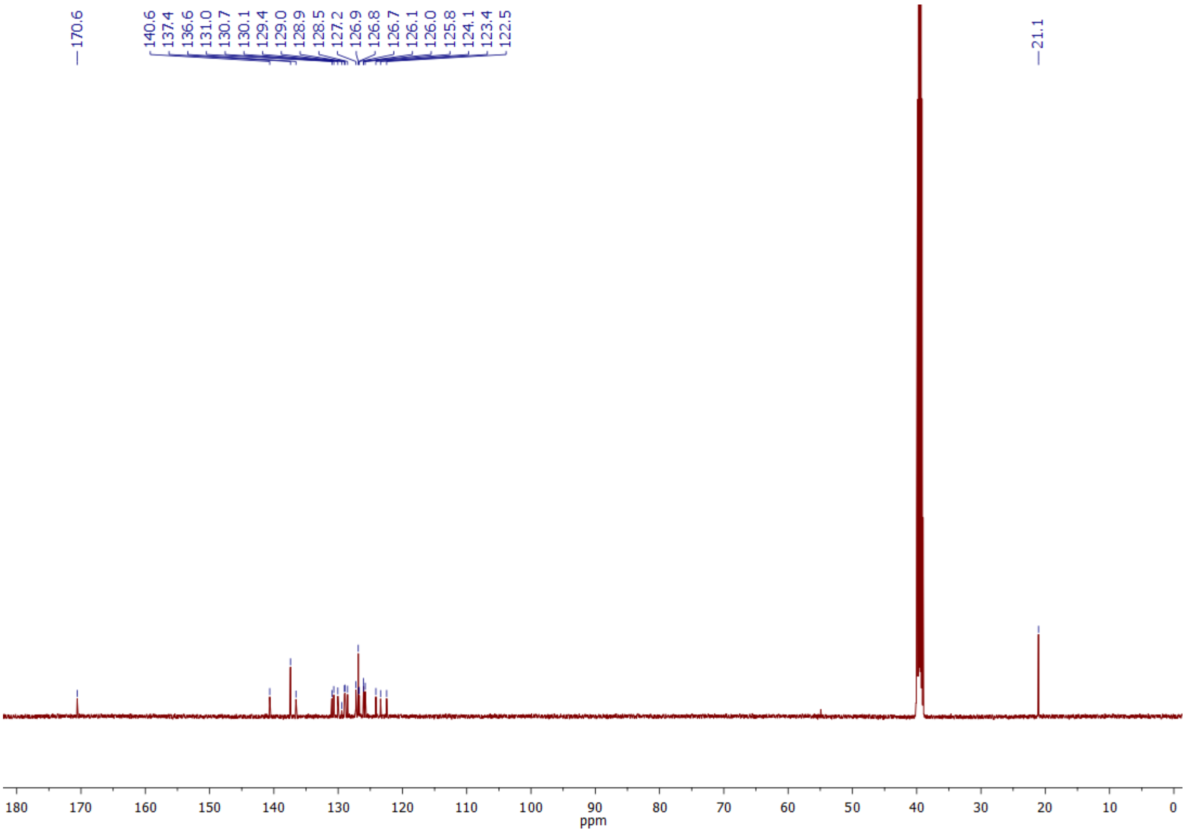

2-(4-Methylphenyl)pyrene-1-carboxylic acid (3b)

^{1}H-NMR (500 MHz, DMSO-d$_6$)

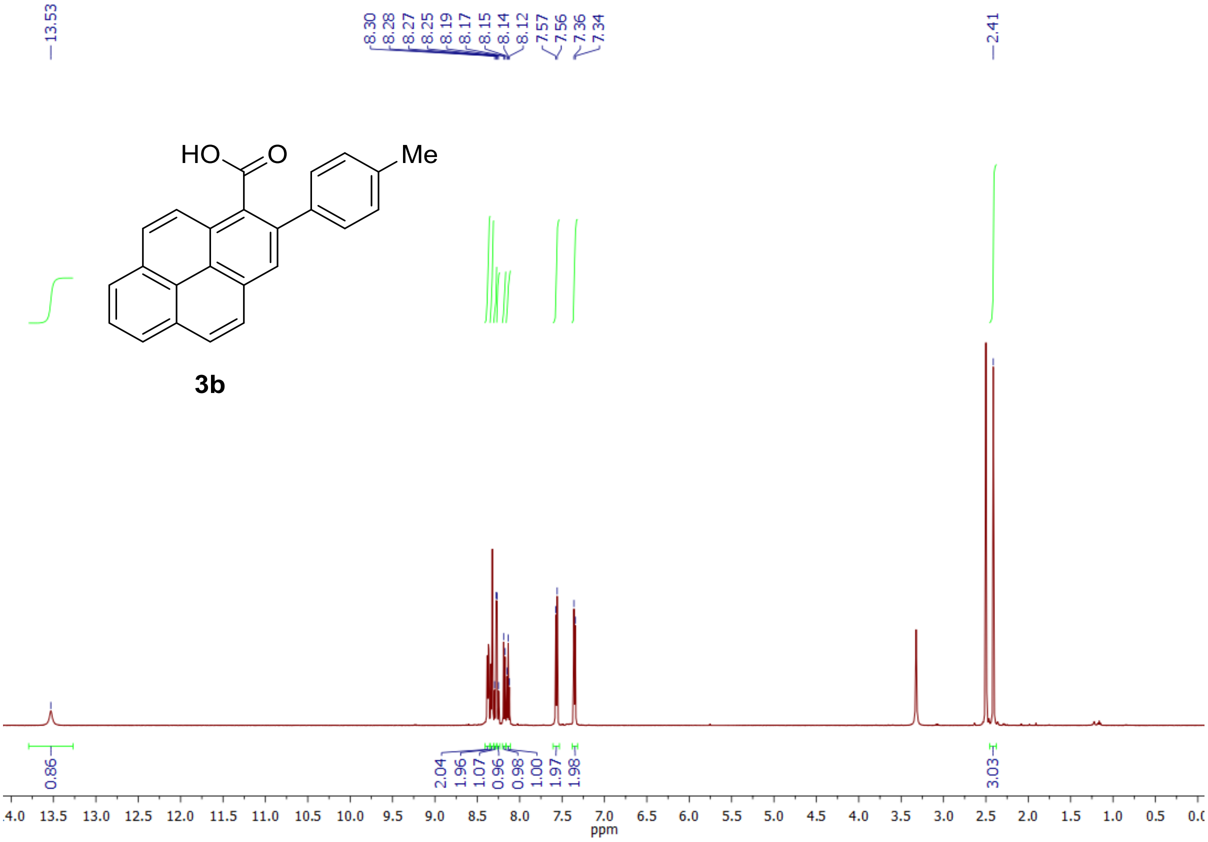

^{13}C-NMR (125 MHz, DMSO-d$_6$)

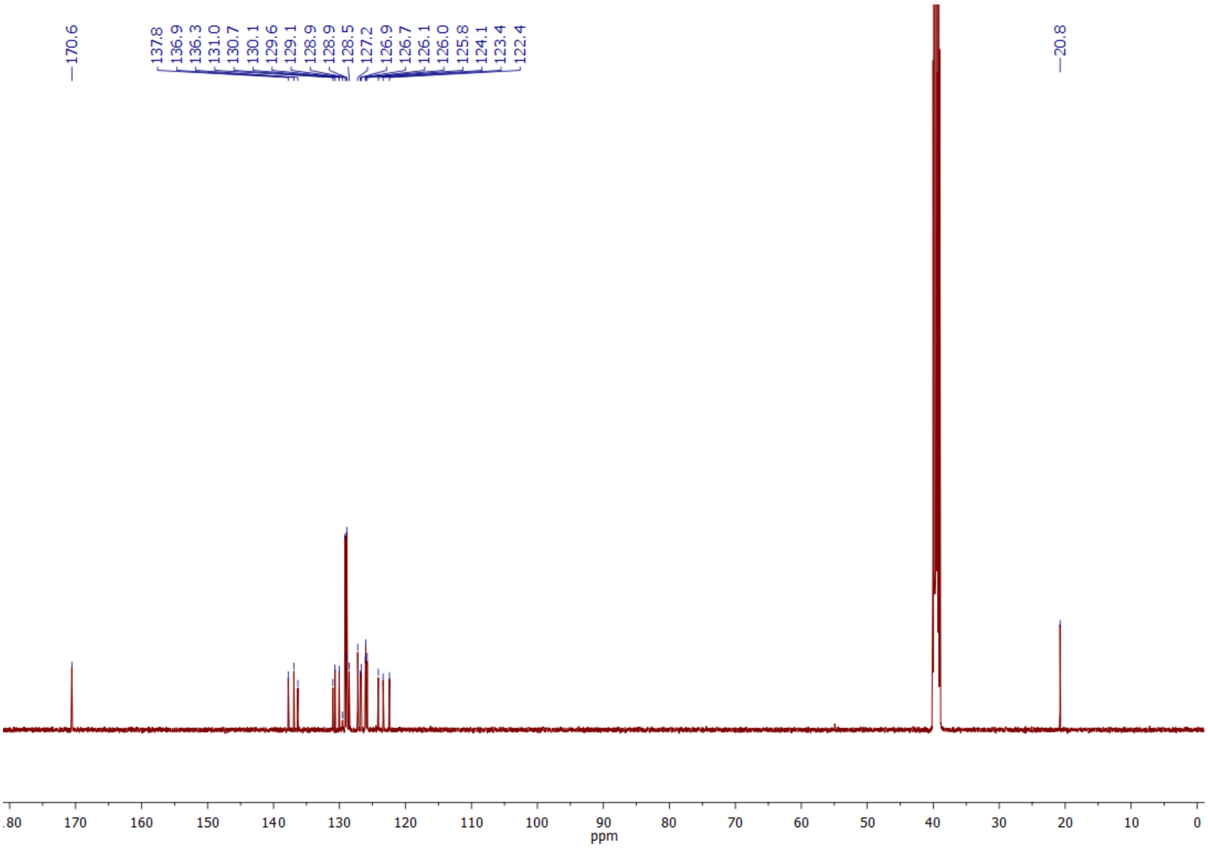

2-(3-Tolyl)pyrene-1-carboxylic acid (3c)

^{1}H-NMR (400 MHz, DMSO-d$_6$)

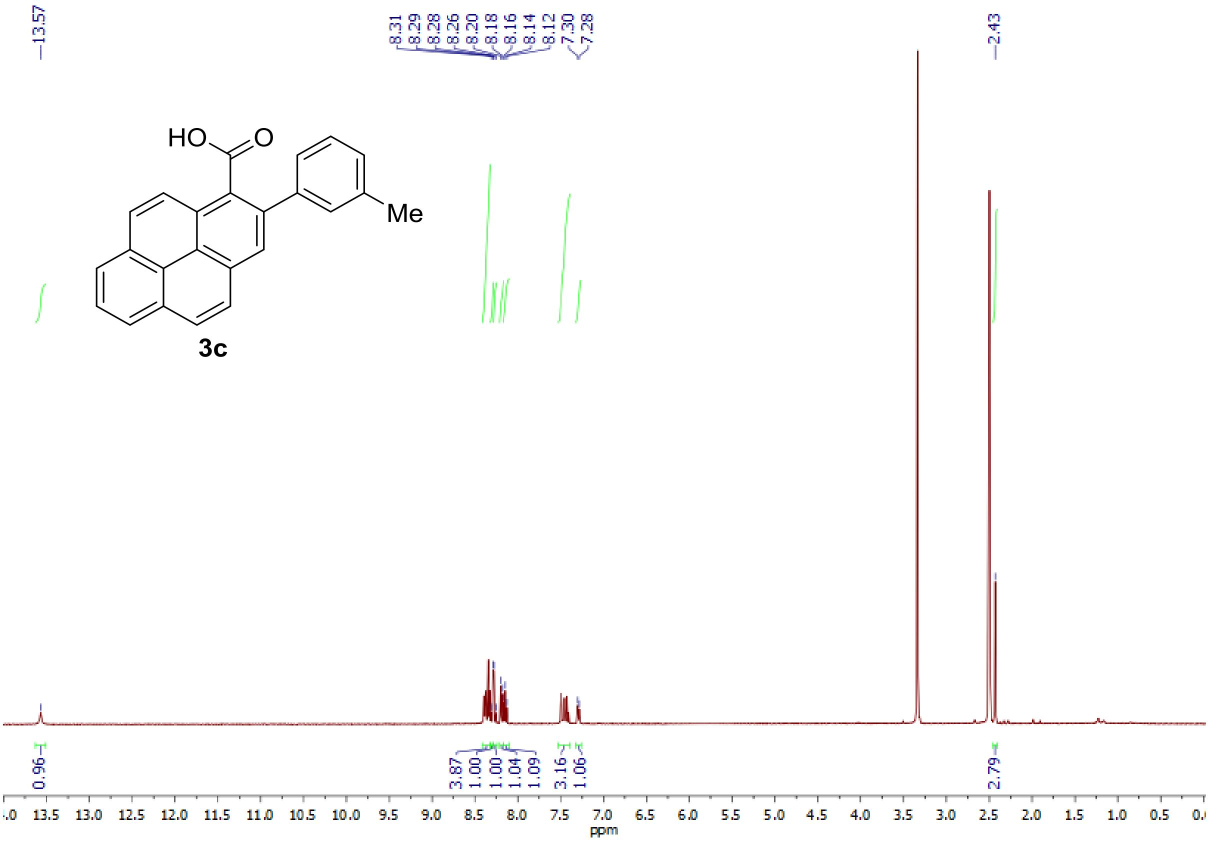

^{13}C-NMR (125 MHz, DMSO-d$_6$)

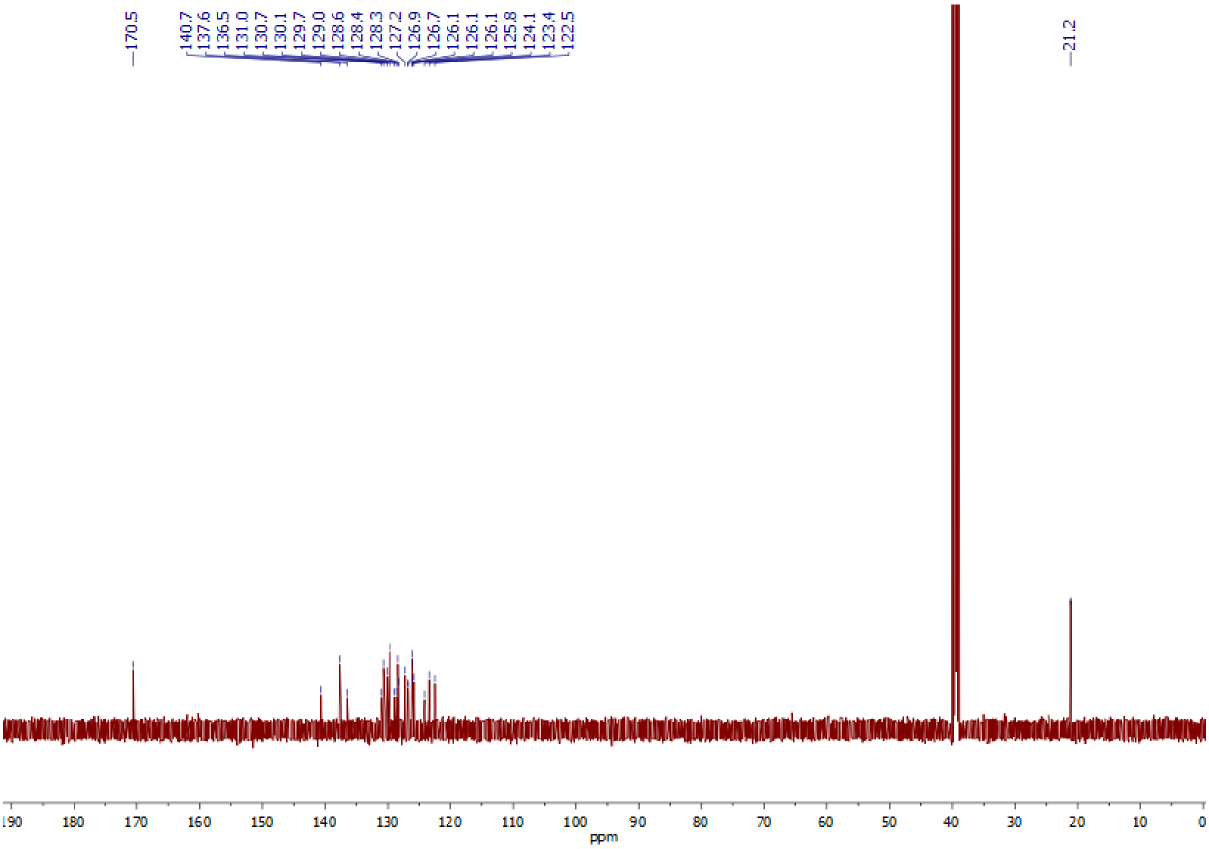

2-(4-*tert*-Butylphenyl)pyrene-1-carboxylic acid (3d)

^{1}H-NMR (500 MHz, DMSO-d$_6$)

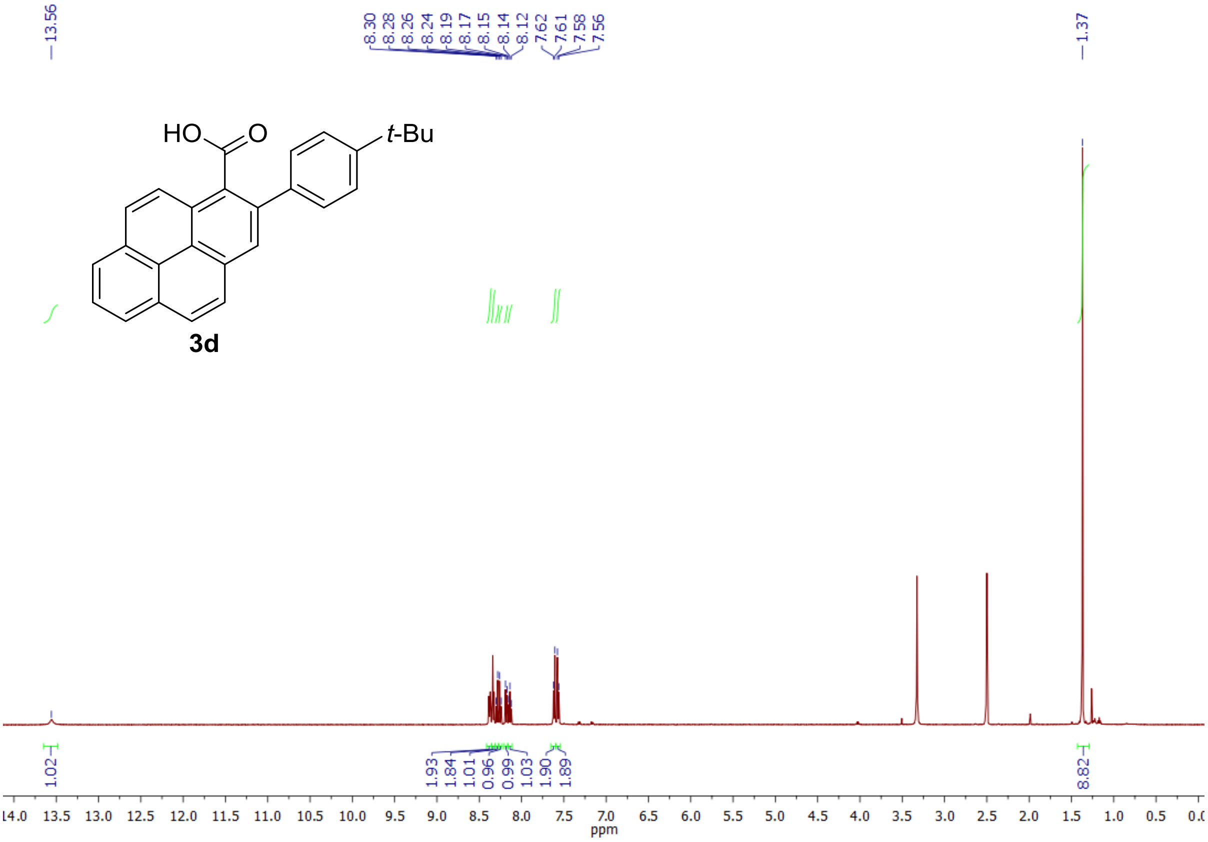

^{13}C-NMR (125 MHz, DMSO-d$_6$)

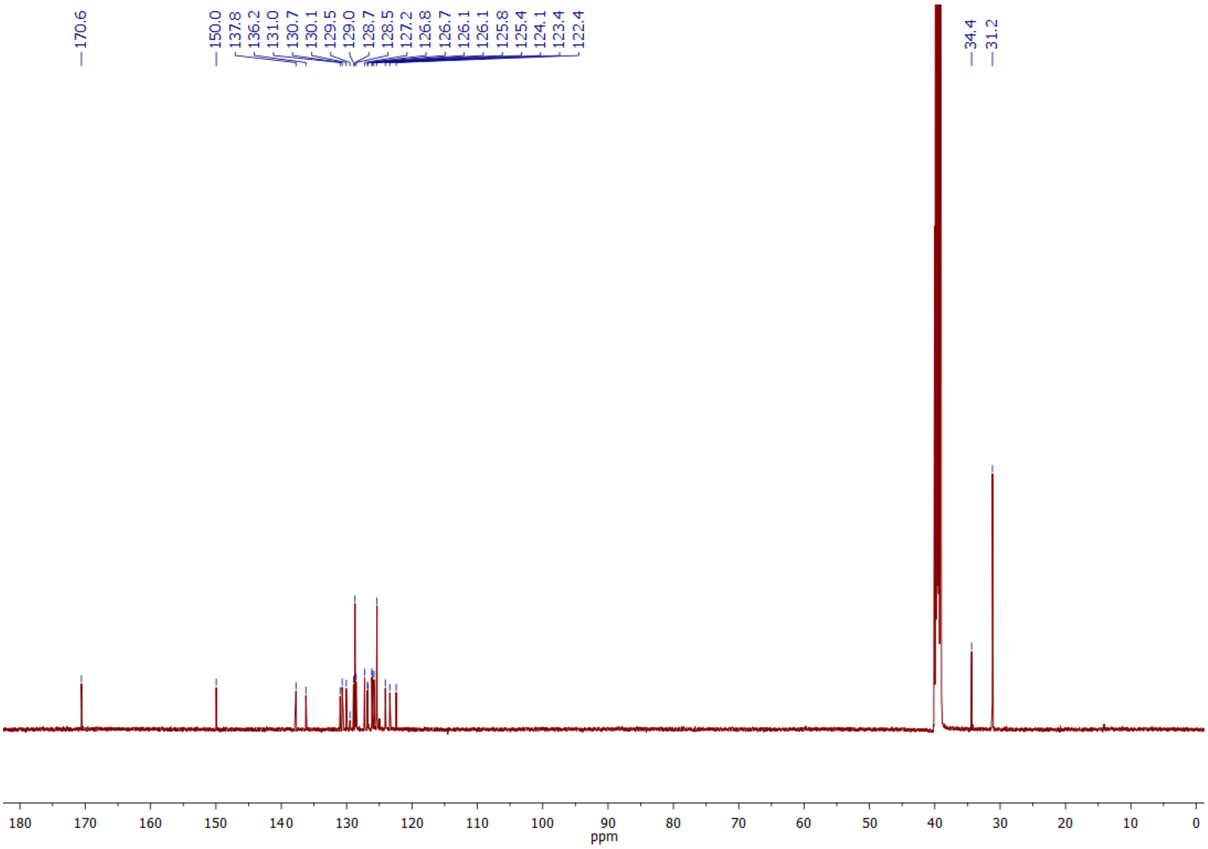

2-(4-Methoxyphenyl)pyrene-1-carboxylic acid (3e)

^{1}H-NMR (500 MHz, DMSO-d_6)

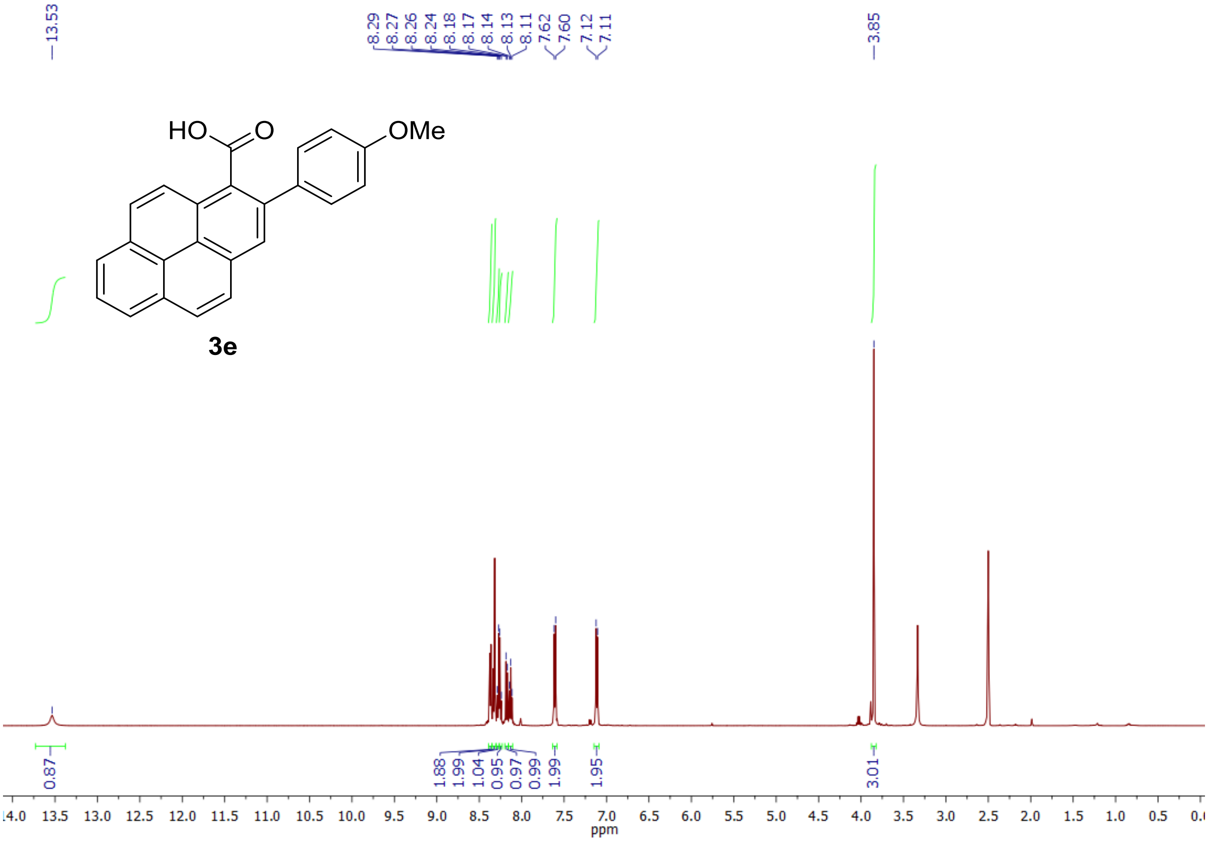

^{13}C-NMR (125 MHz, DMSO-d_6)

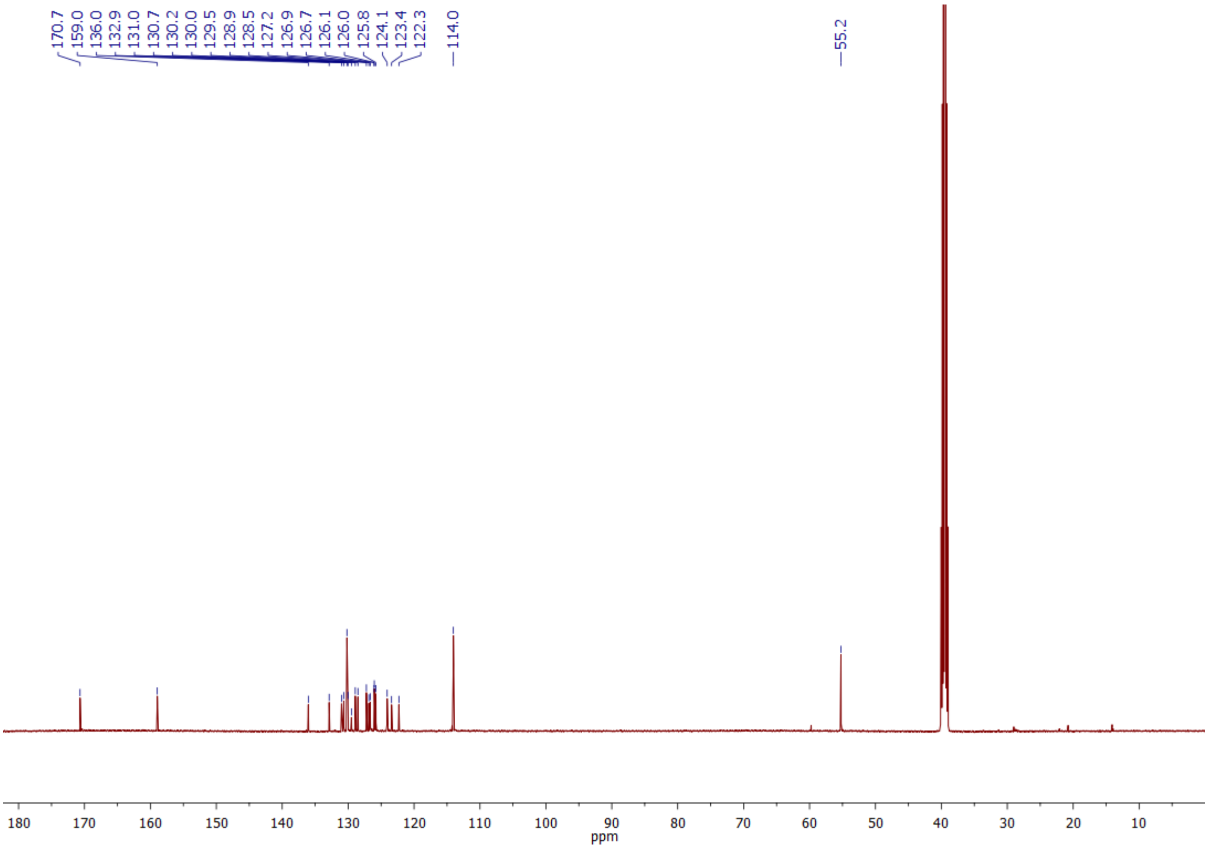

2-Phenylpyrene-1-carboxylic acid (3f)

^{1}H-NMR (500 MHz, DMSO-d$_6$)

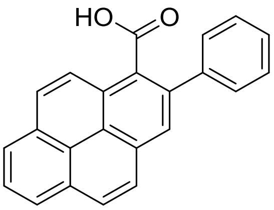

3f

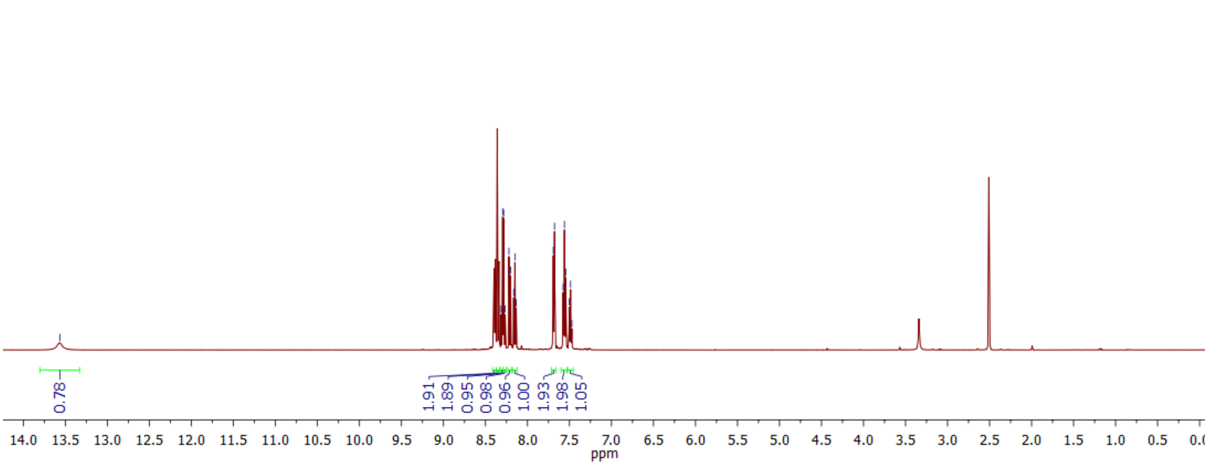

^{13}C-NMR (125 MHz, DMSO-d$_6$)

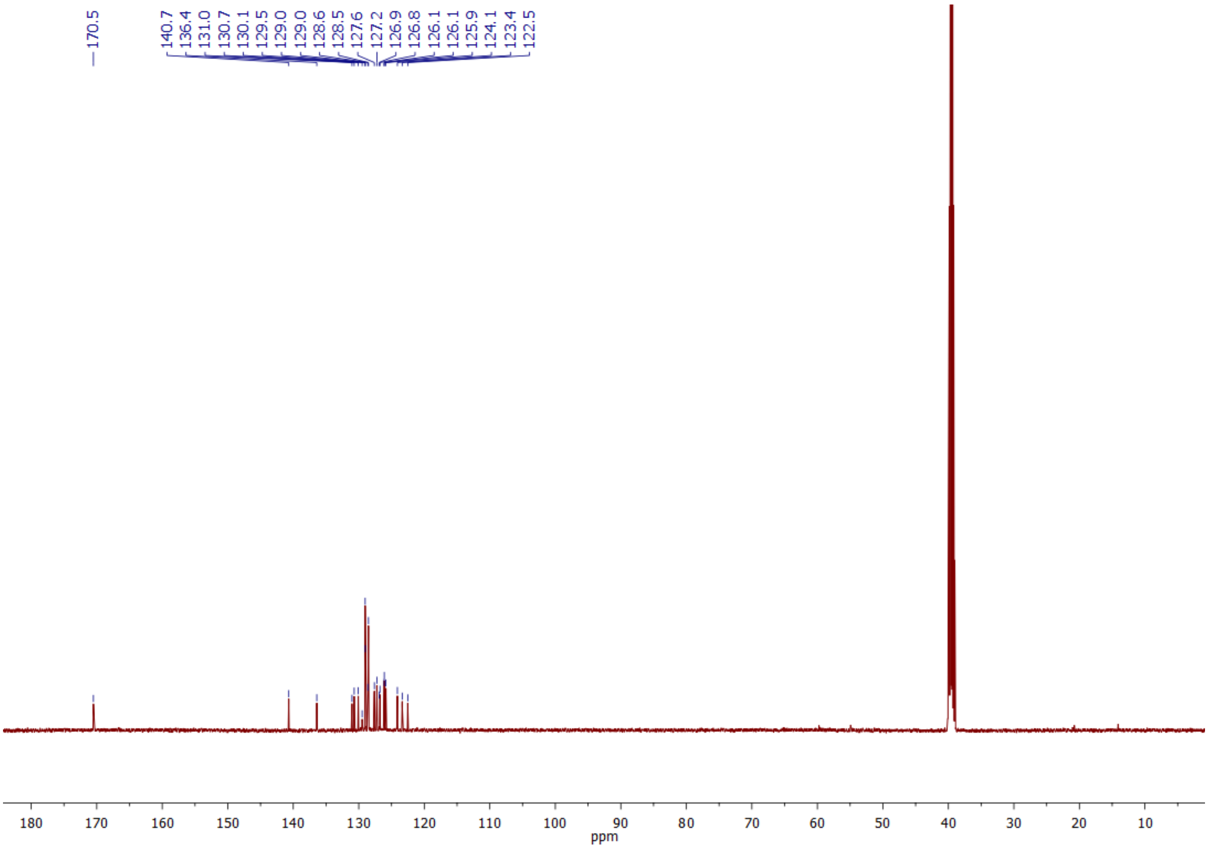

2-(4-Bromophenyl)pyrene-1-carboxylic acid (3g)

^{1}H-NMR (500 MHz, DMSO-d$_6$)

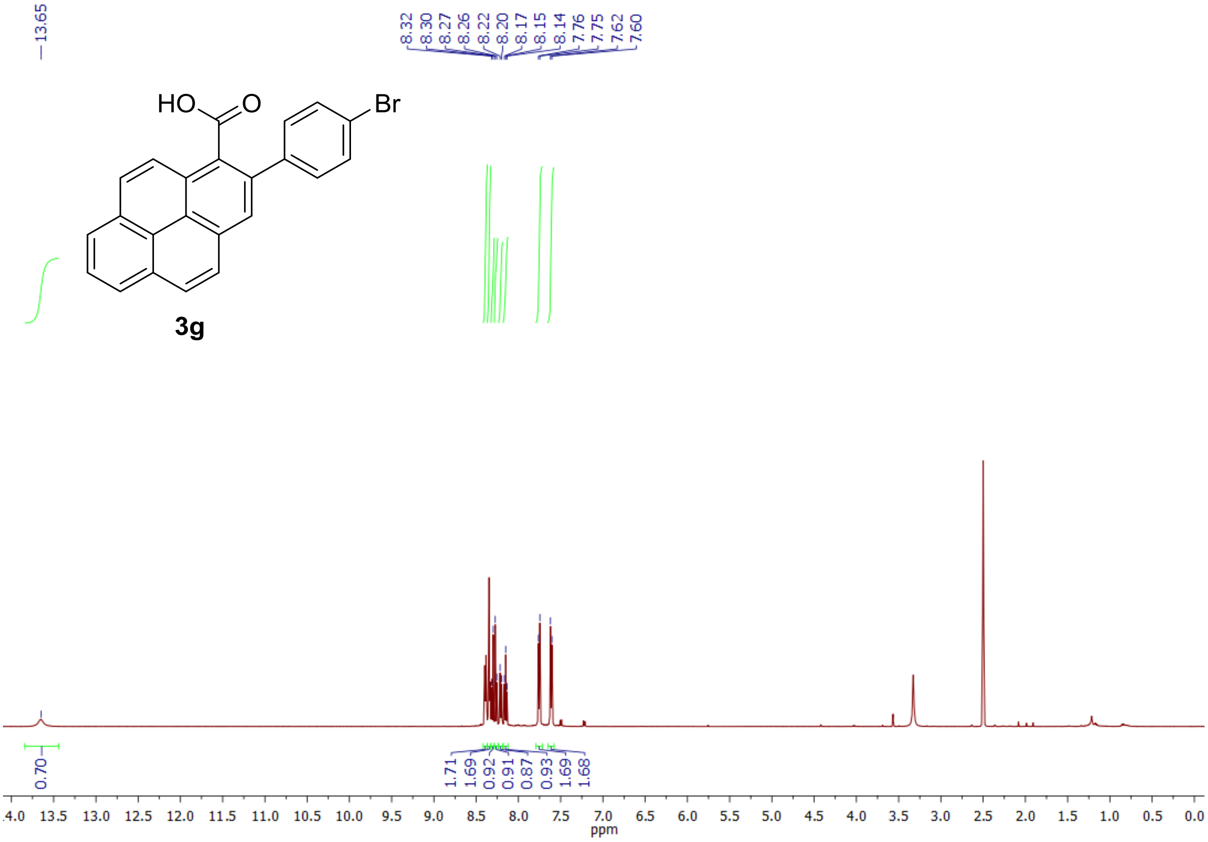

^{13}C-NMR (125 MHz, DMSO-d$_6$)

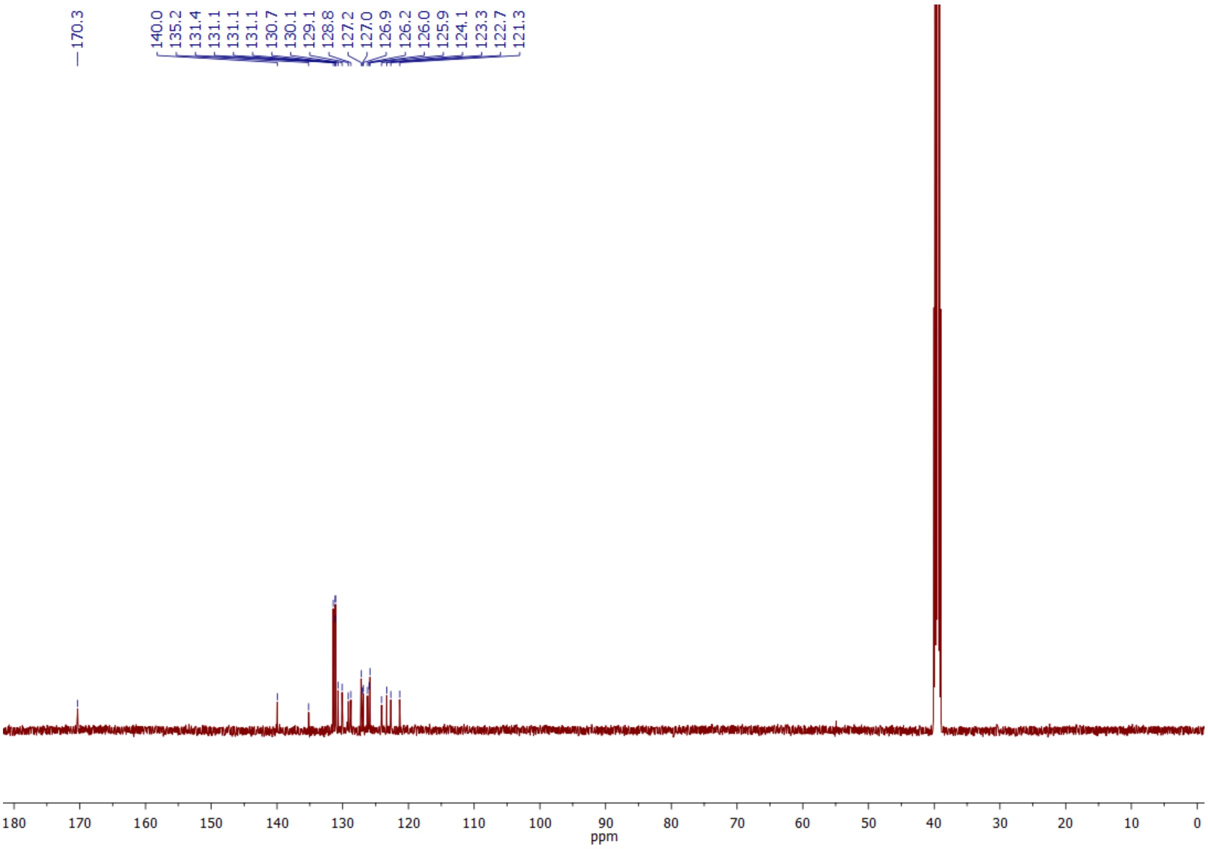

2-(3-Bromophenyl)pyrene-1-carboxylic acid (3h)

^{1}H-NMR (400 MHz, DMSO-d$_6$)

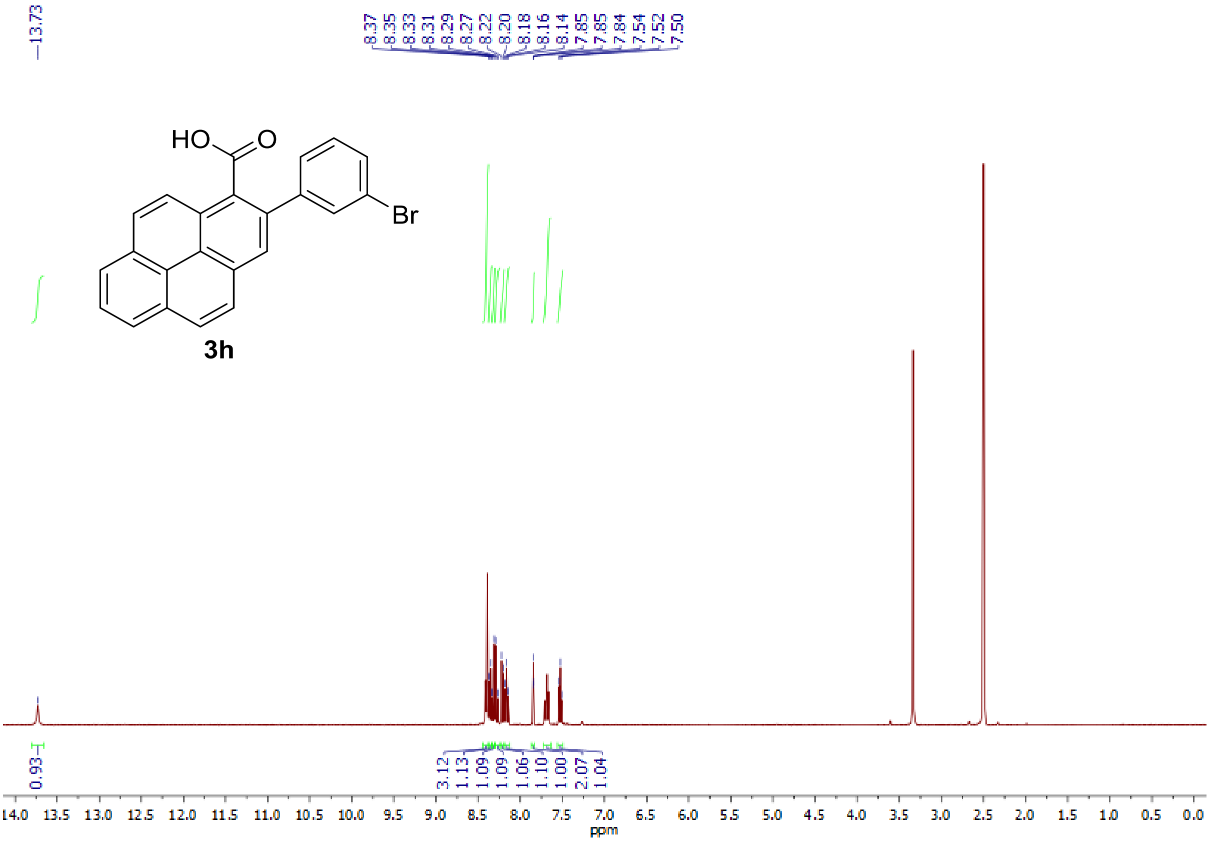

^{13}C-NMR (125 MHz, DMSO-d$_6$)

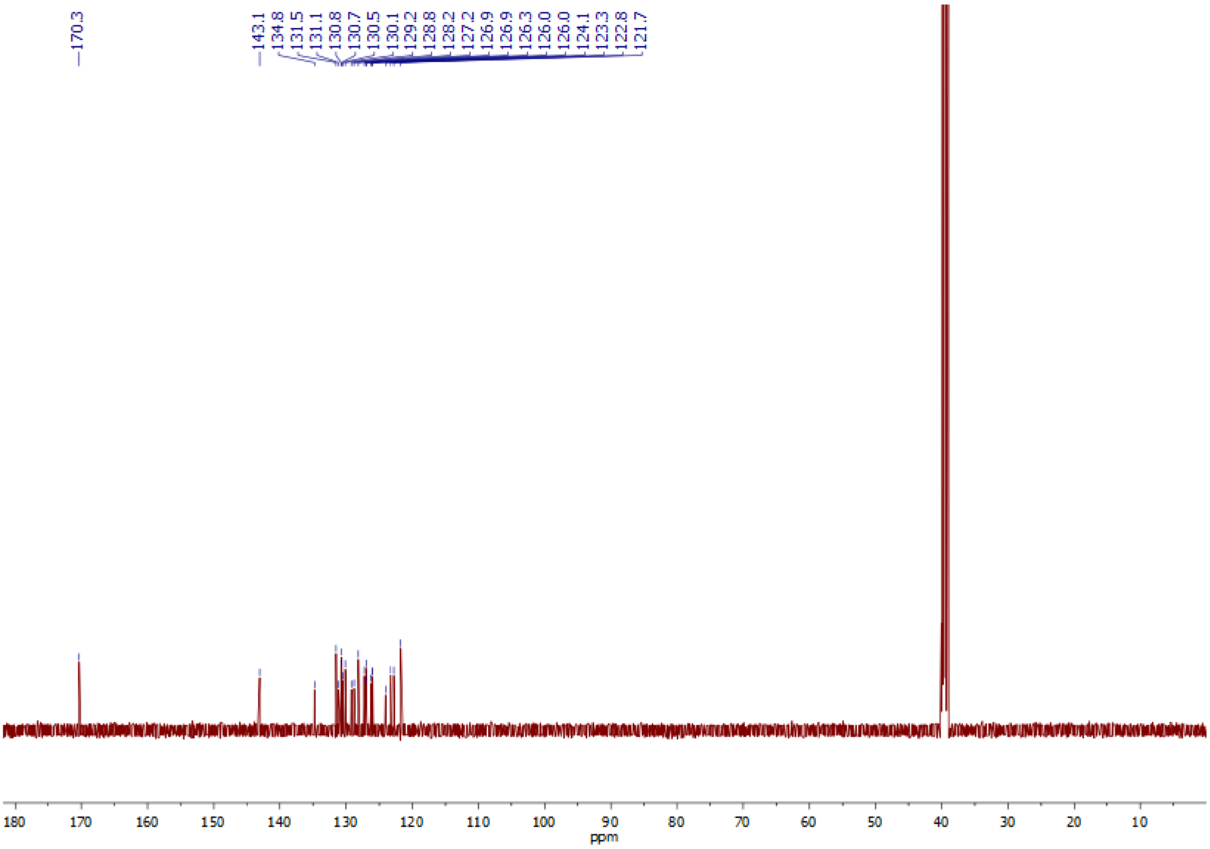

2-(3-Chlorophenyl)pyrene-1-carboxylic acid (3i)

^{1}H-NMR (400 MHz, DMSO-d_6)

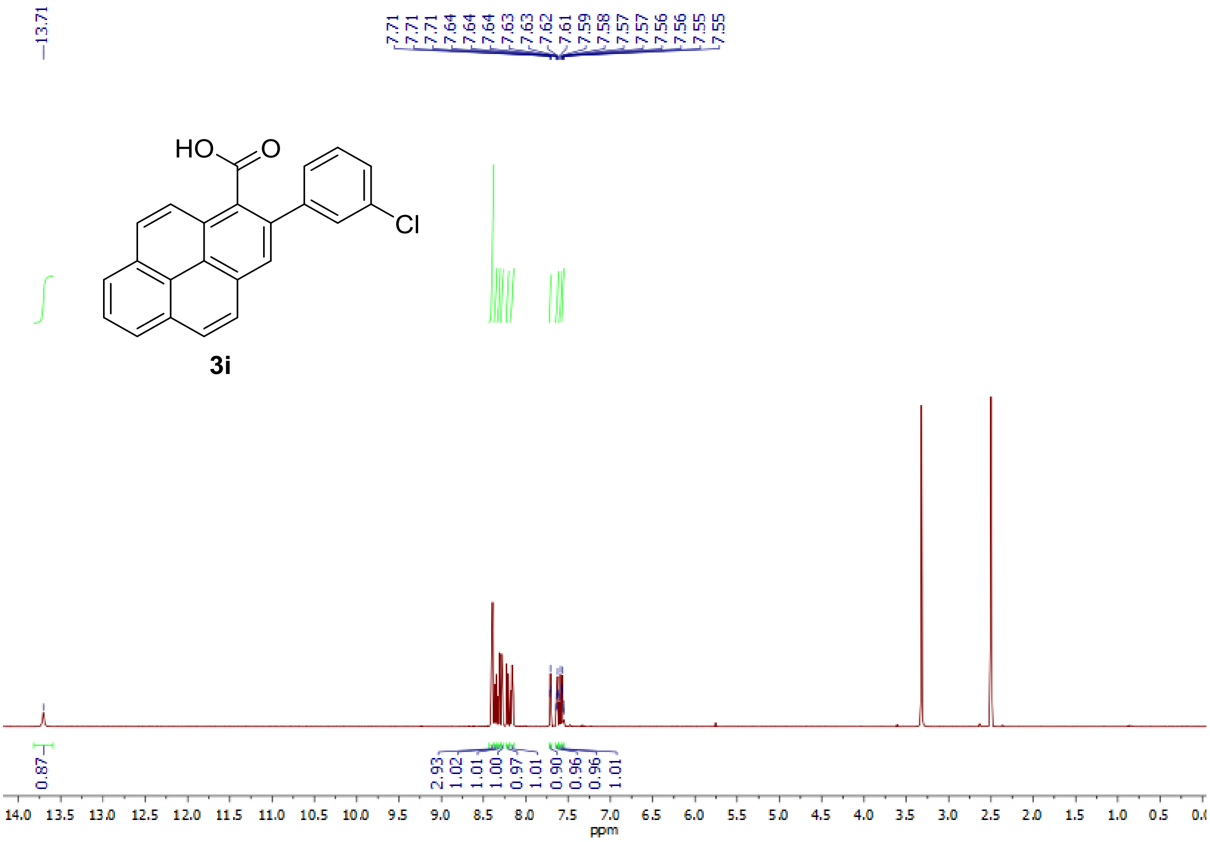

^{13}C-NMR (125 MHz, DMSO-d_6)

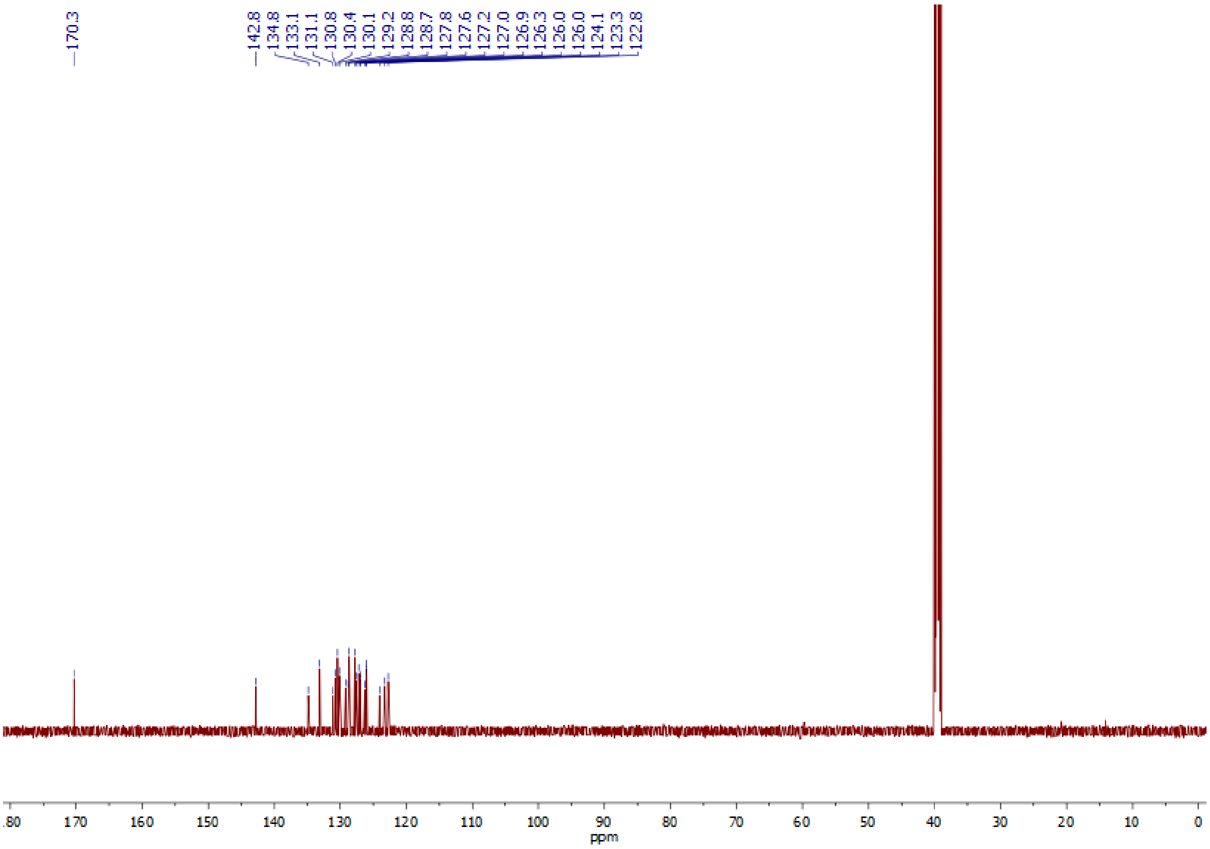

2-(3,5-Dichlorophenyl)pyrene-1-carboxylic acid (3j)

^{1}H-NMR (400 MHz, DMSO-d$_6$)

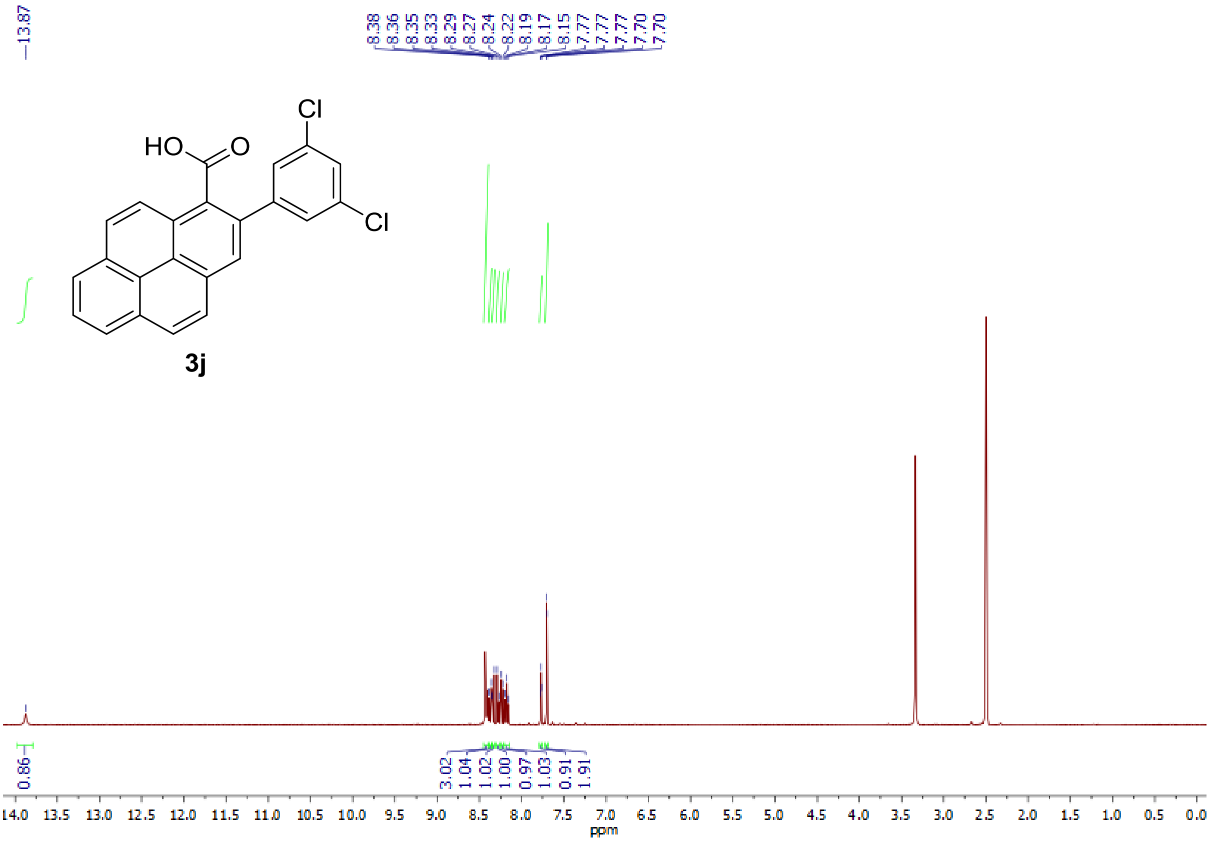

^{13}C-NMR (125 MHz, DMSO-d$_6$)

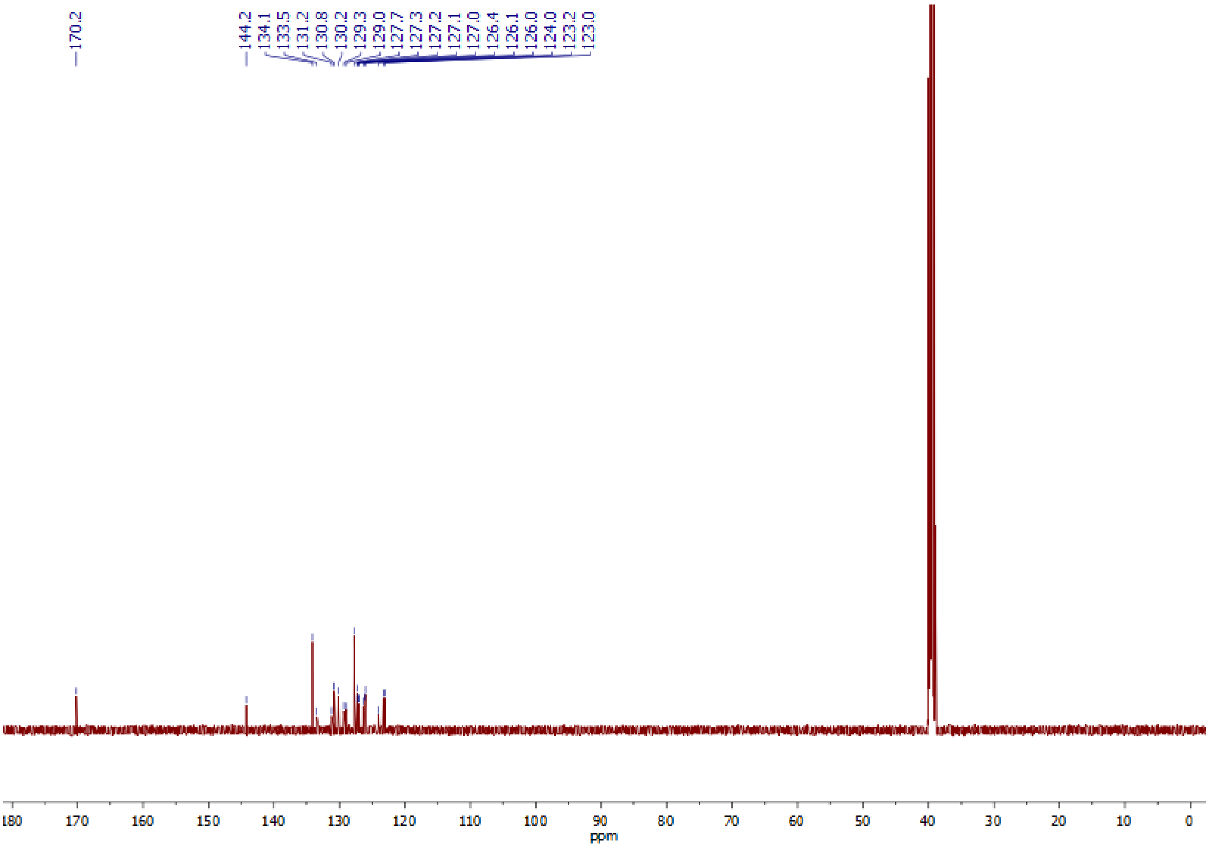

2-(3,5-Bis(trifluoromethyl)phenyl)pyrene-1-carboxylic acid (3k)

^{1}H-NMR (500 MHz, DMSO-d$_6$)

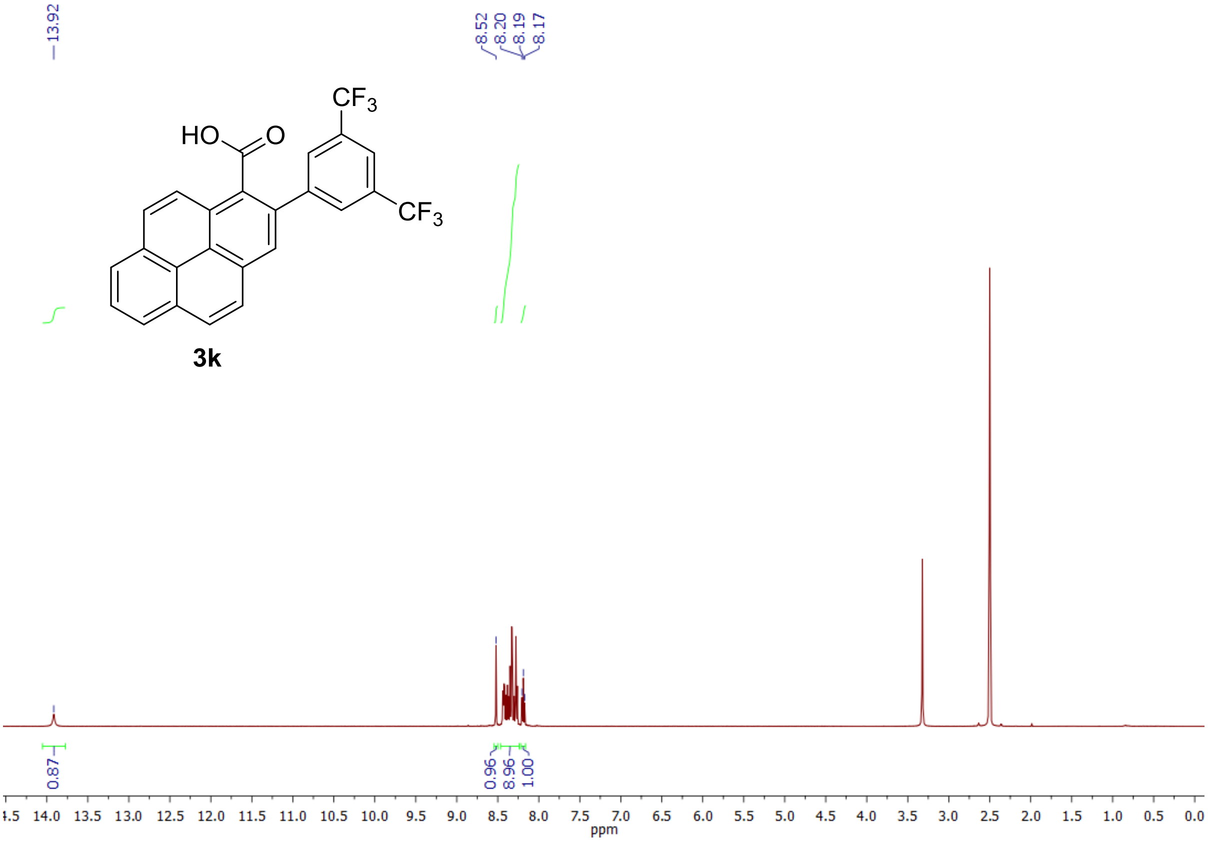

^{13}C-NMR (125 MHz, DMSO-d$_6$)

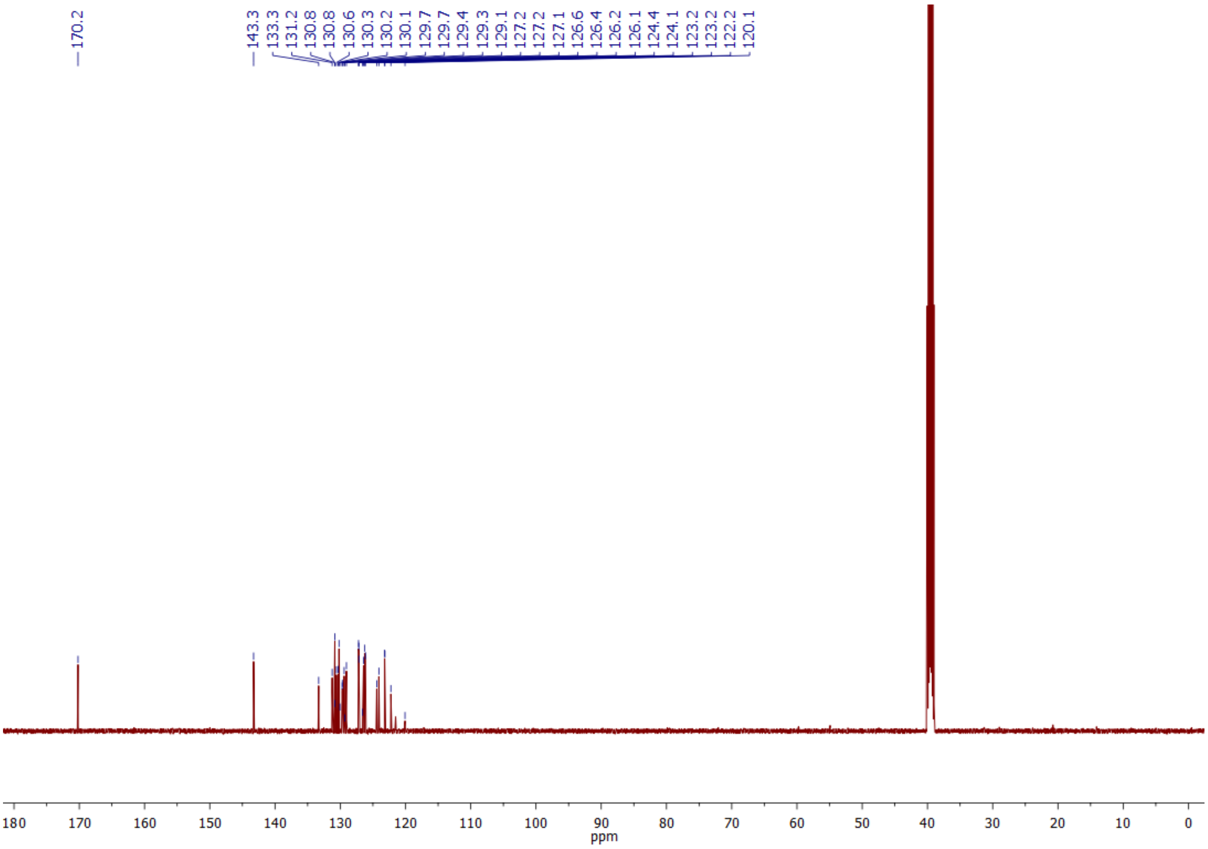

^{19}F-NMR (471 MHz, DMSO-d$_6$)

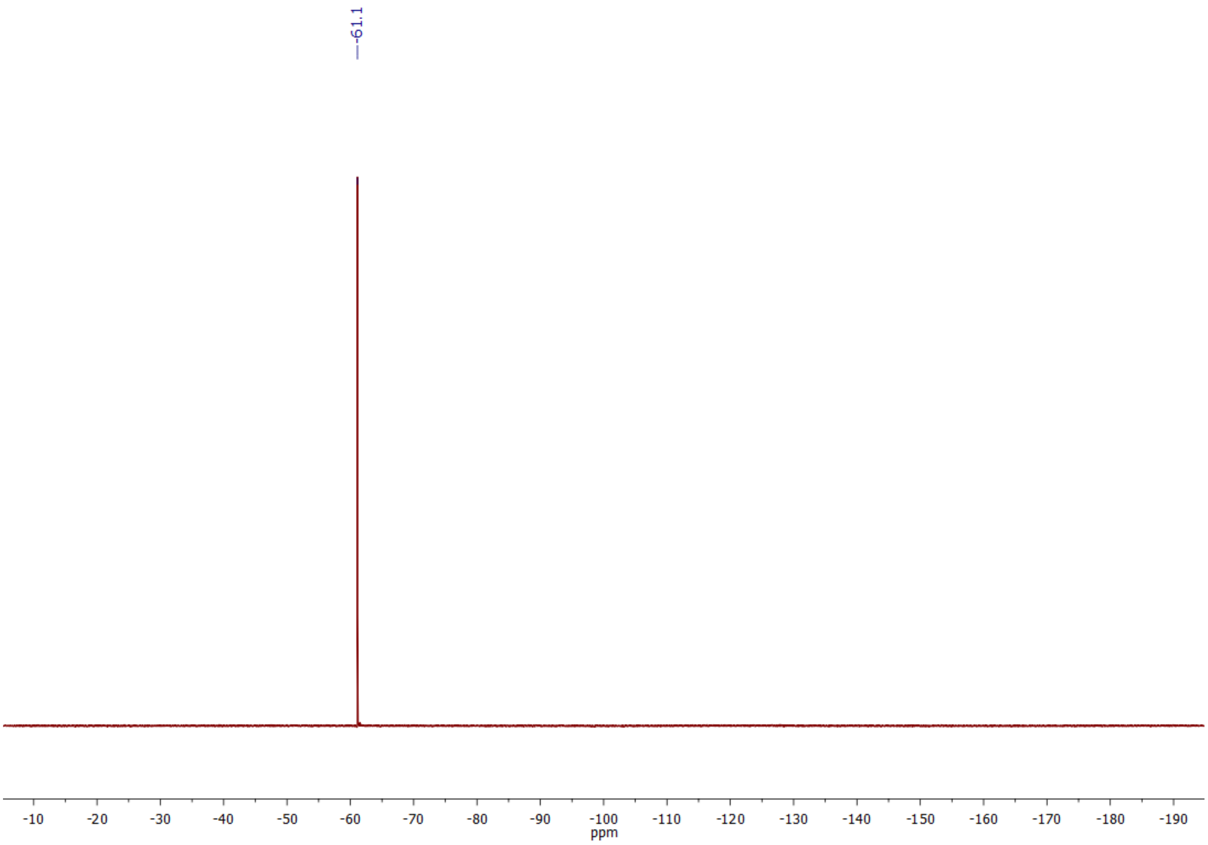

2-(3-Nitrophenyl)pyrene-1-carboxylic acid (3l)

^{1}H-NMR (400 MHz, DMSO-d$_6$)

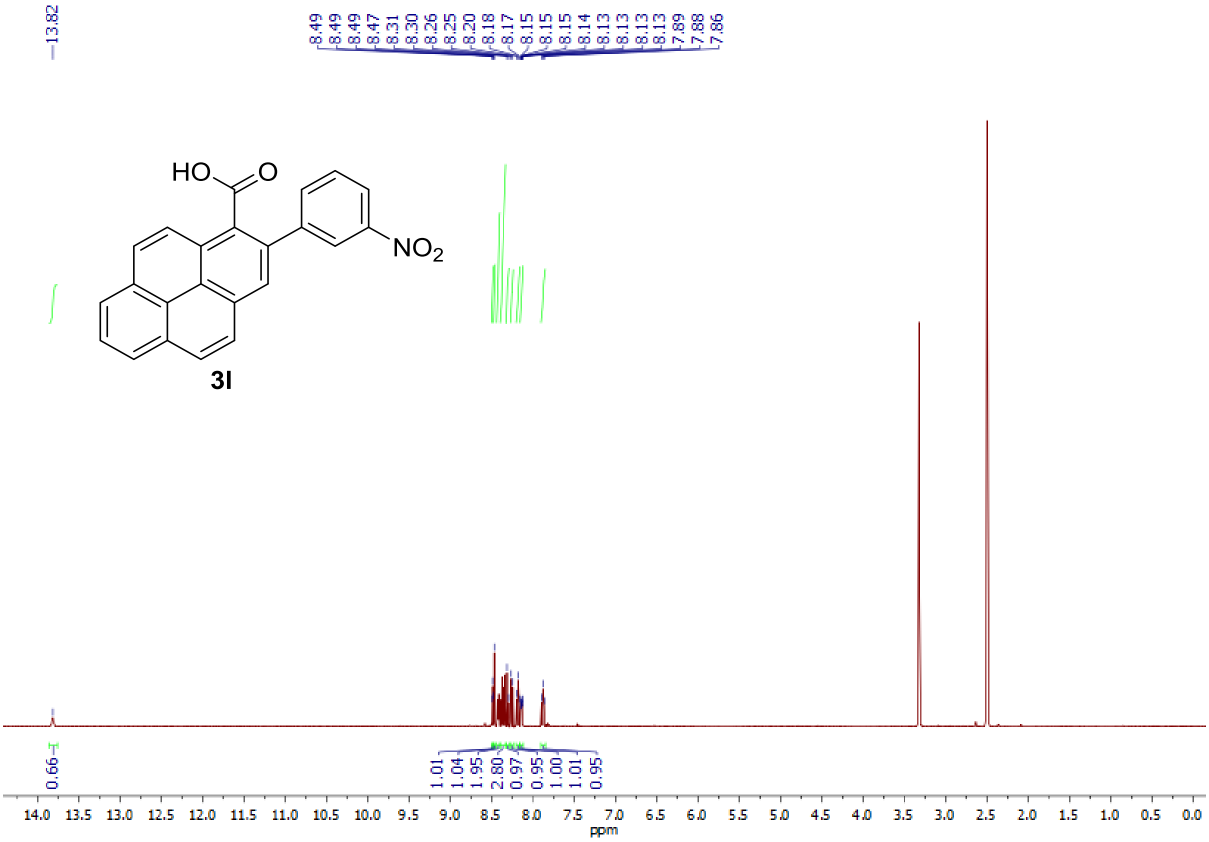

^{13}C-NMR (125 MHz, DMSO-d$_6$)

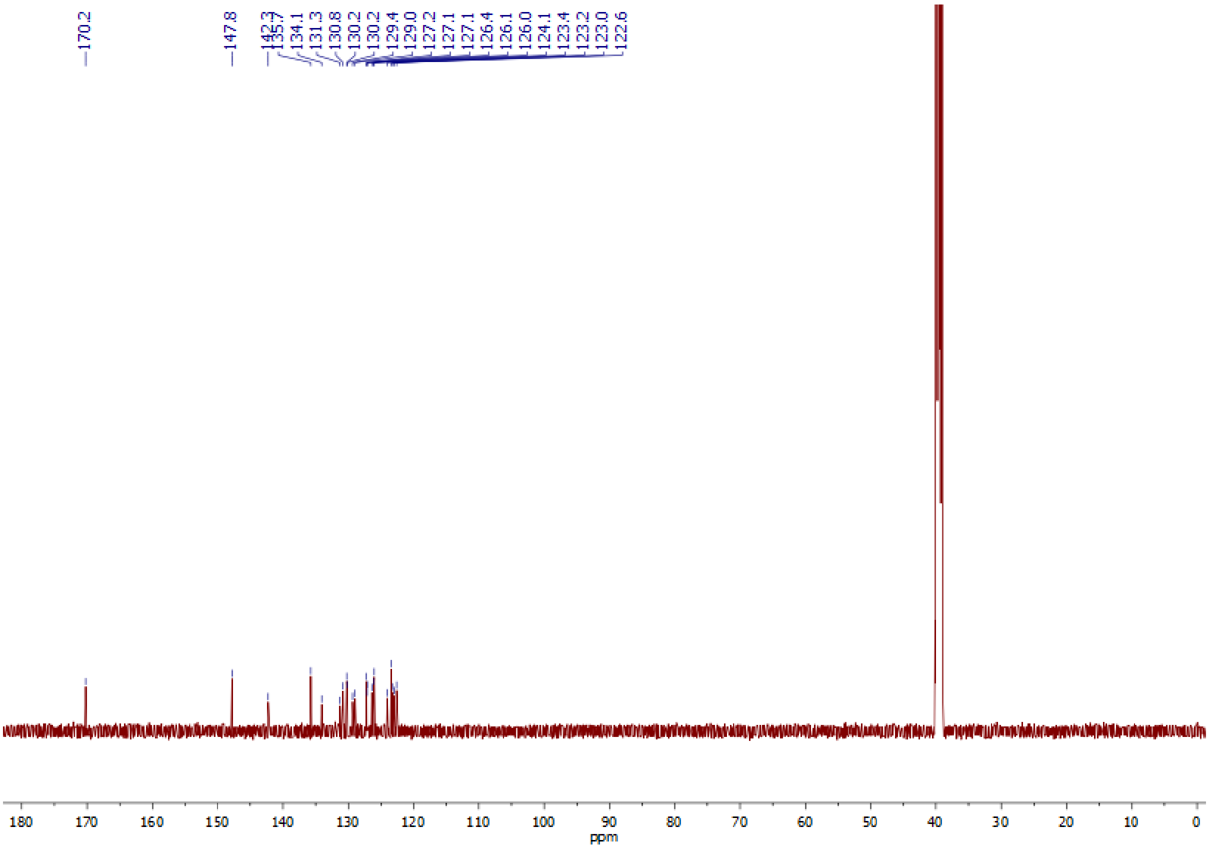

2-(3-(Trifluoromethyl)phenyl)pyrene-1-carboxylic acid (3m)

^{1}H-NMR (400 MHz, DMSO-d$_6$)

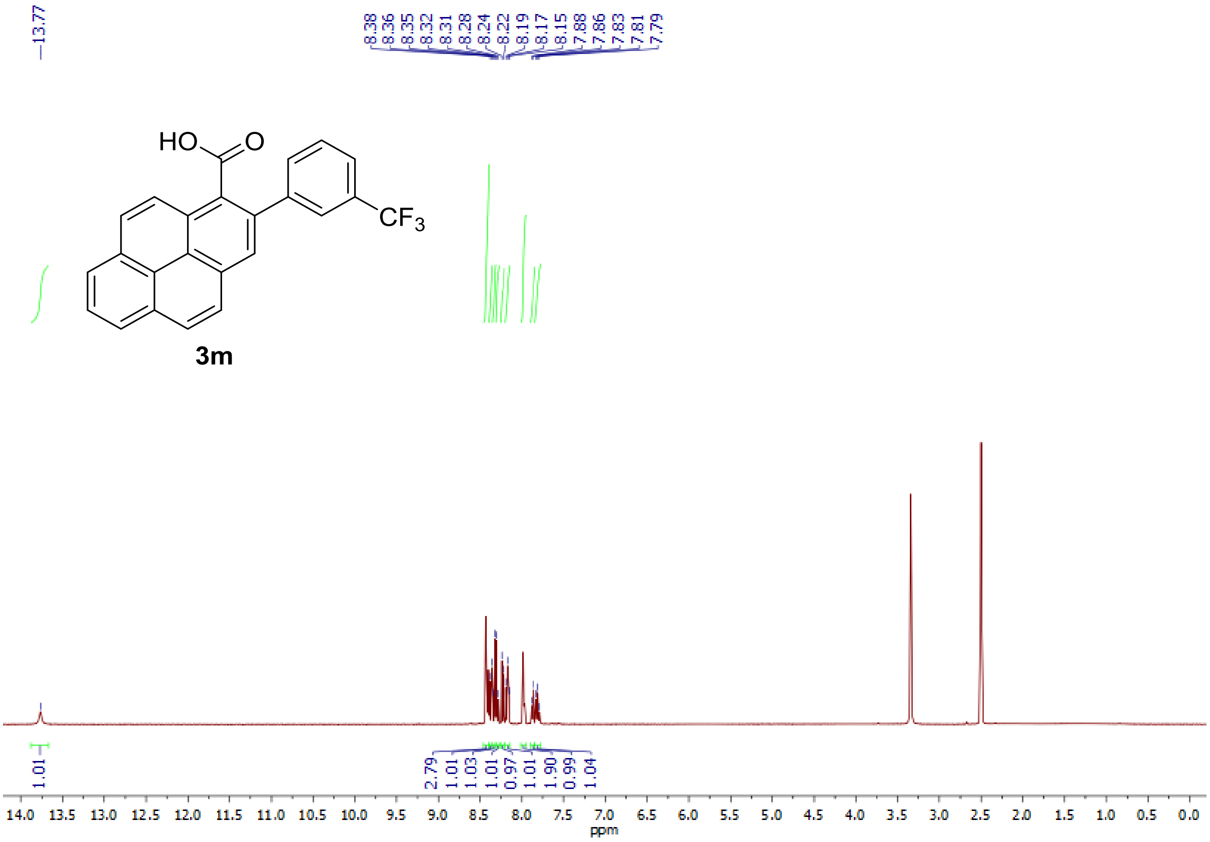

^{13}C-NMR (125 MHz, DMSO-d$_6$)

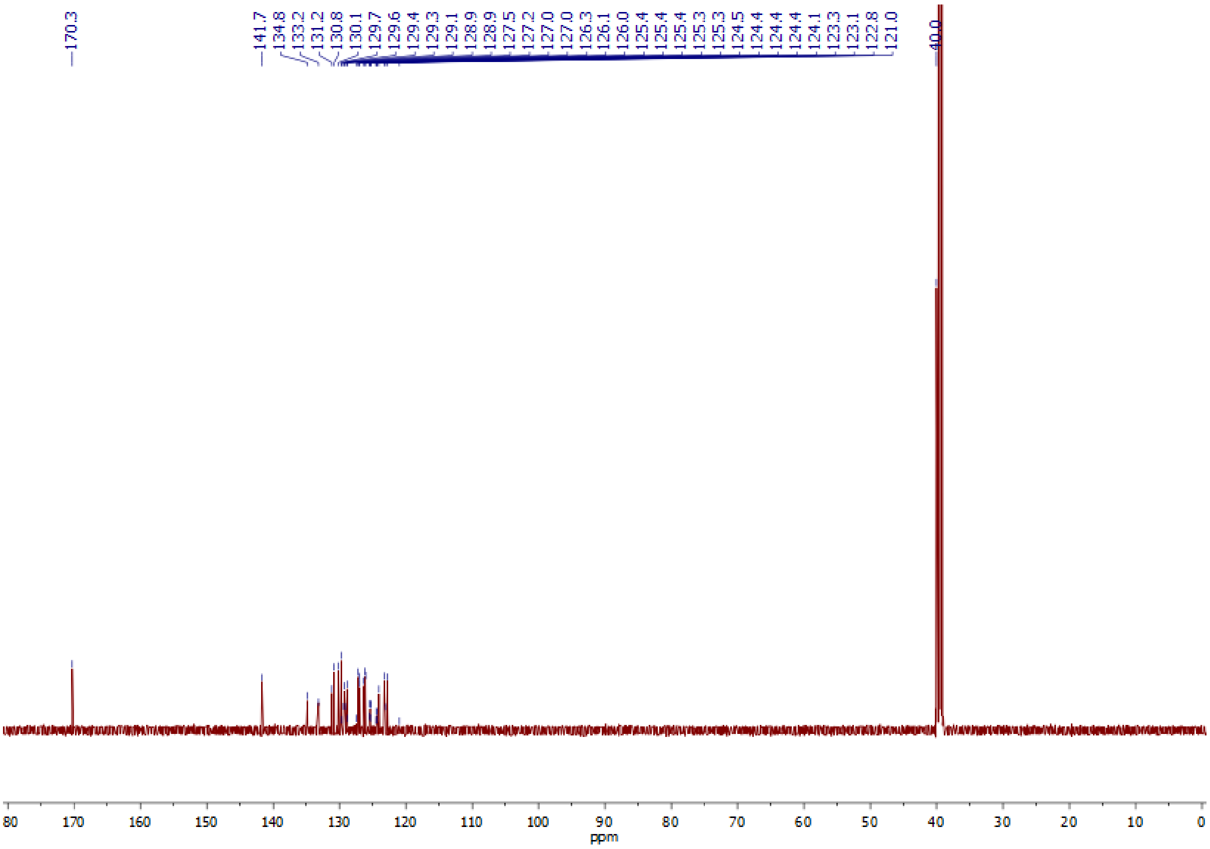

^{19}F-NMR (471 MHz, DMSO-d_6)

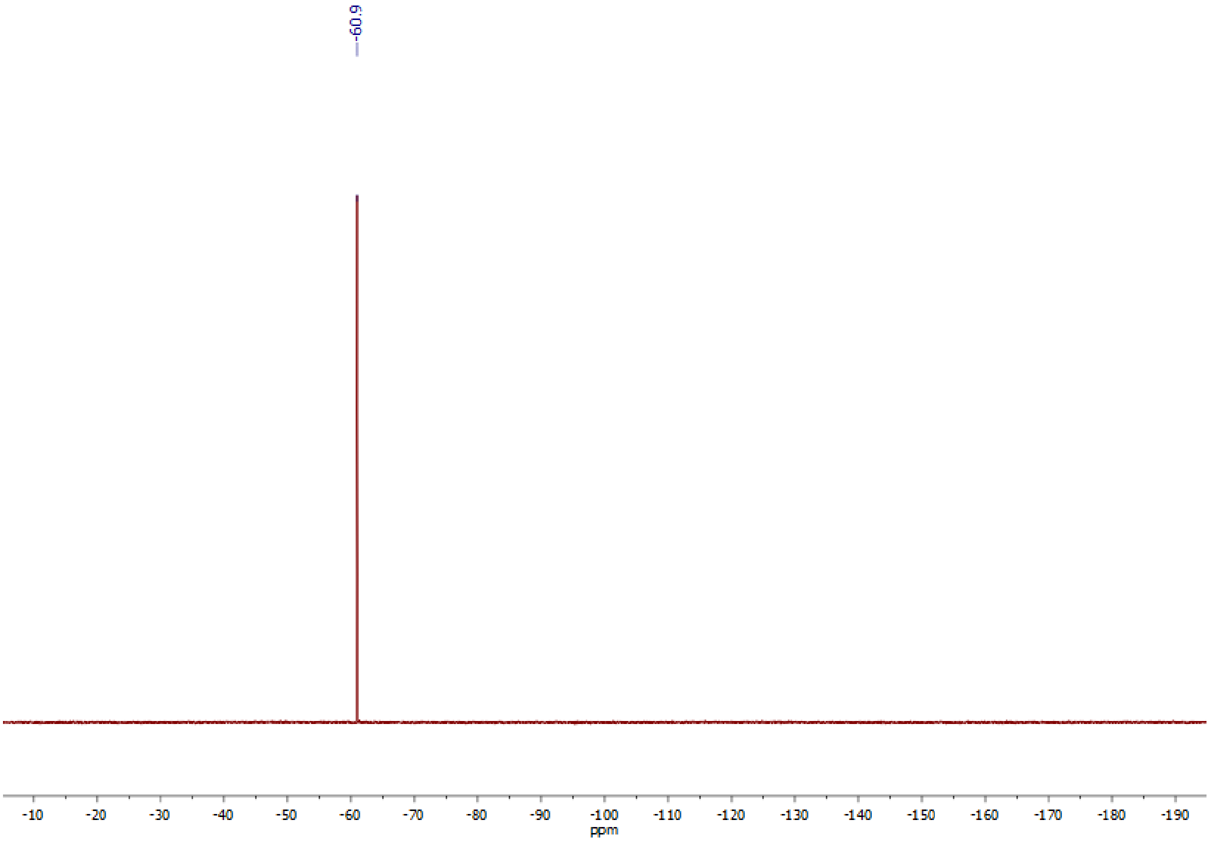

2-(3-Cyanophenyl)pyrene-1-carboxylic acid (3n)

^{1}H-NMR (500 MHz, acetone-d_6)

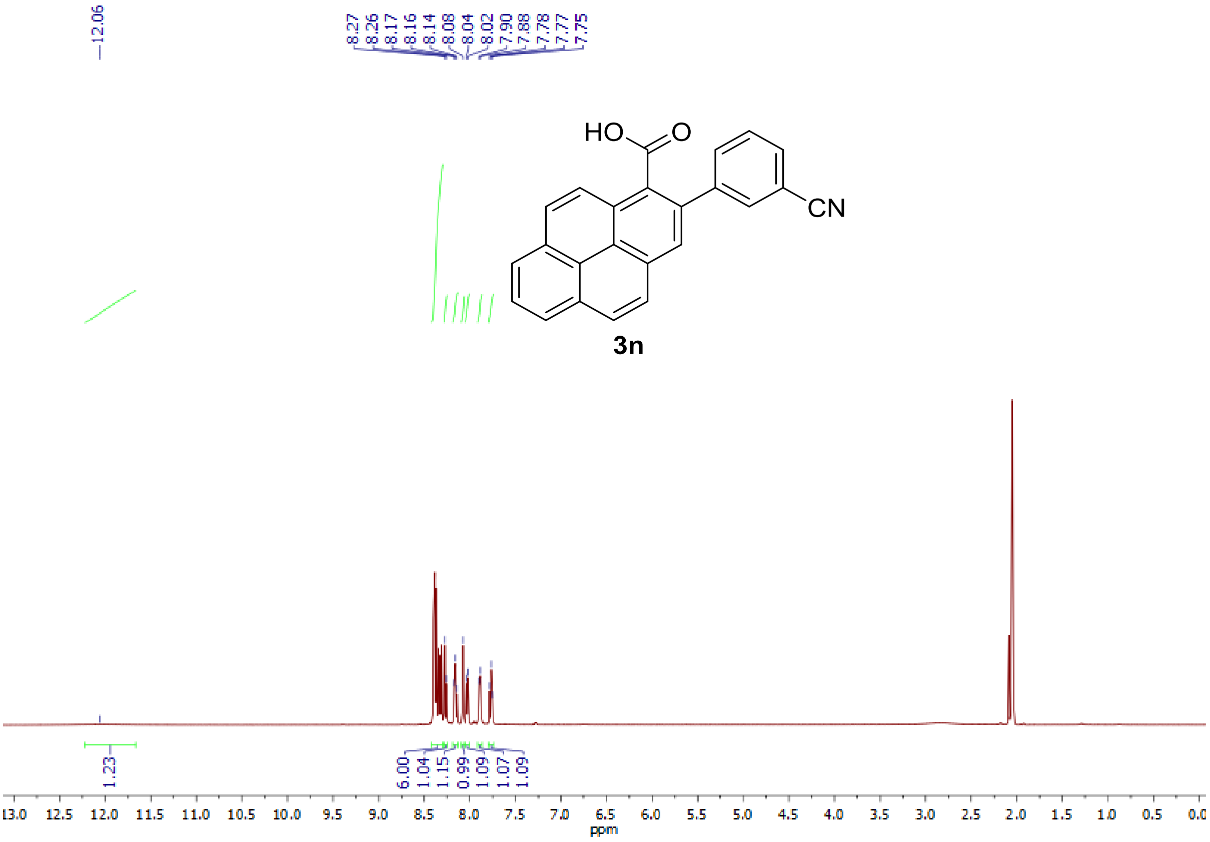

^{13}C-NMR (125 MHz, acetone-d_6)

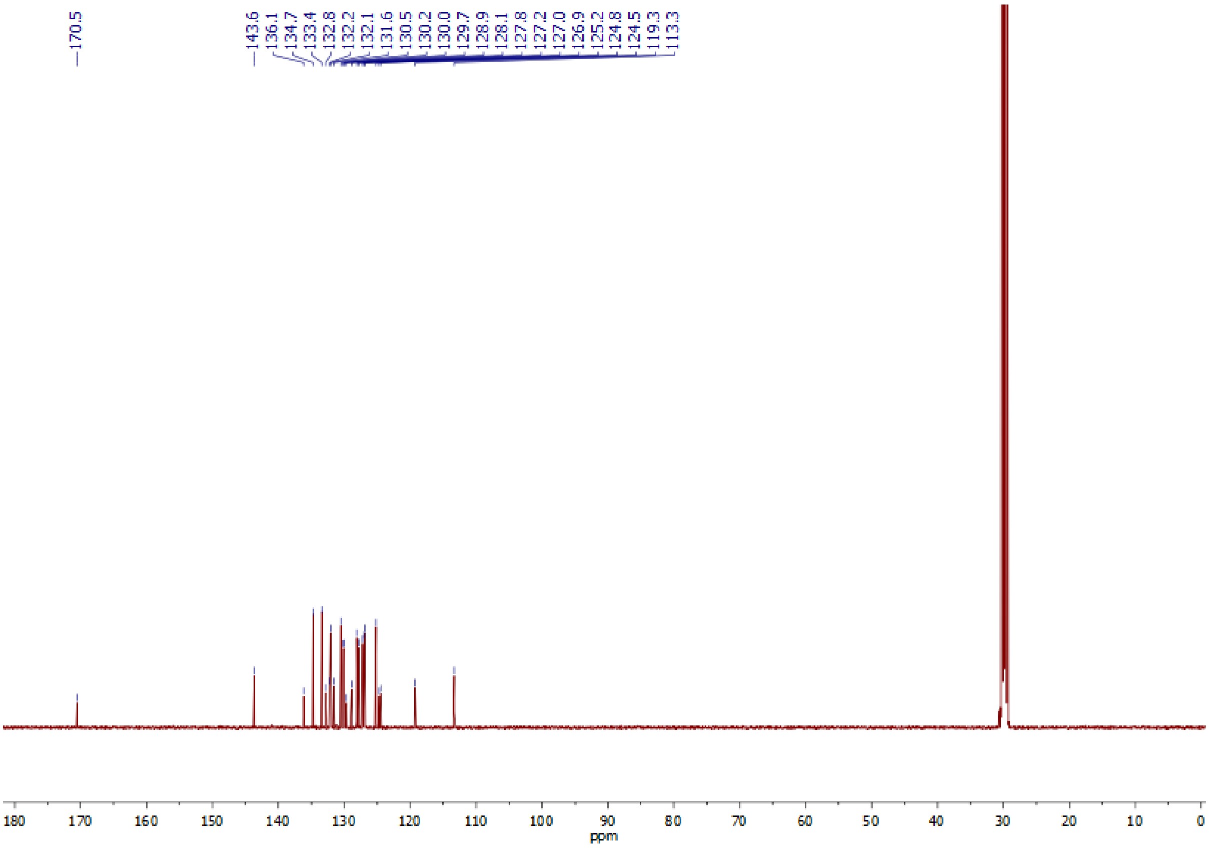

2-(3-(Methoxycarbonyl)phenyl)pyrene-1-carboxylic acid (3o)

^{1}H-NMR (400 MHz, DMSO-d$_6$)

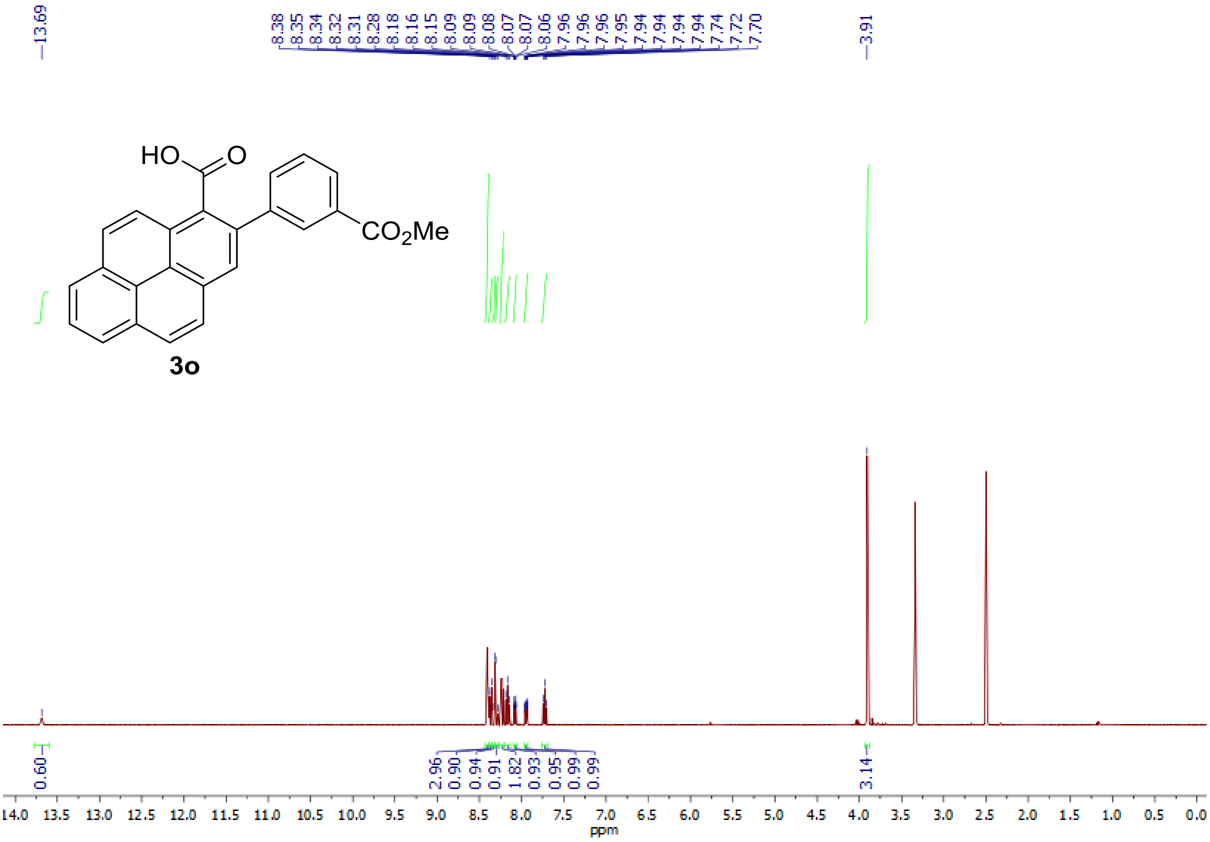

^{13}C-NMR (125 MHz, DMSO-d$_6$)

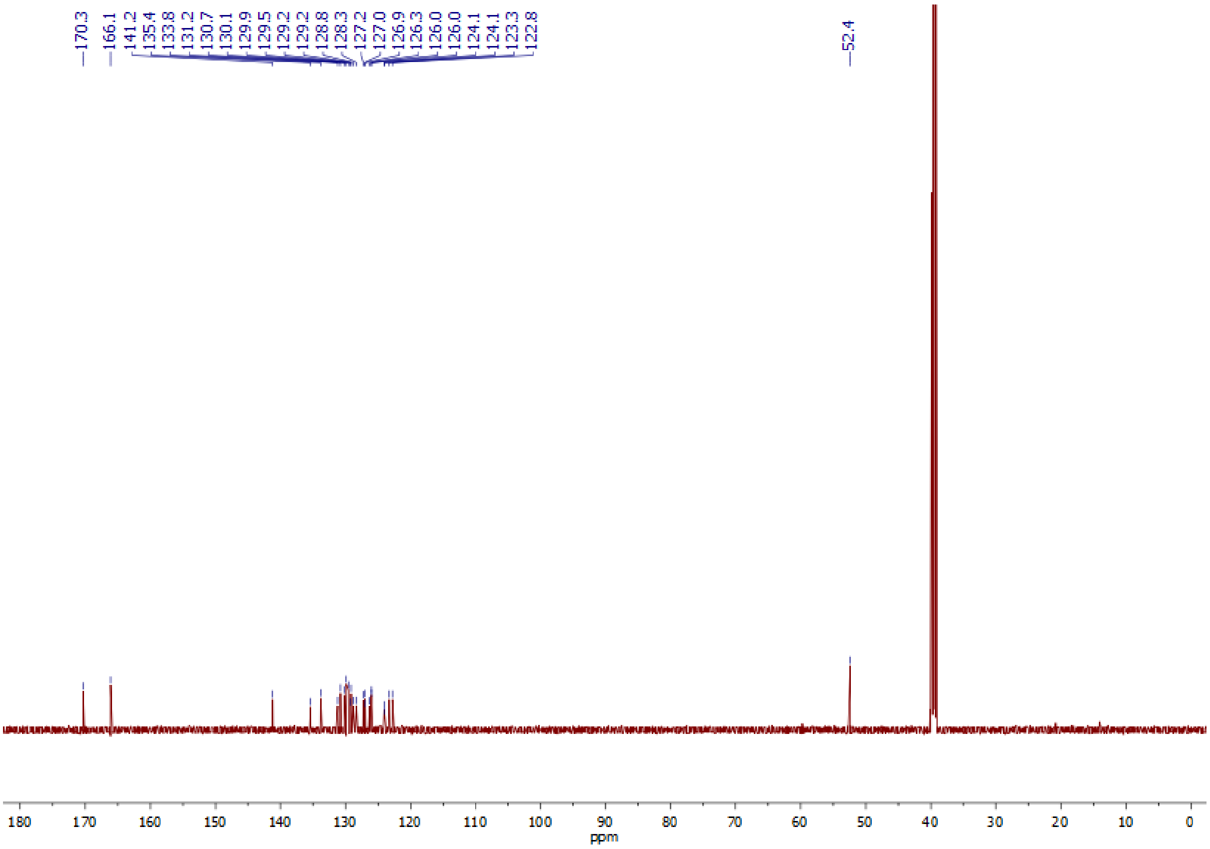

2-(4-(Methoxycarbonyl)phenyl)pyrene-1-carboxylic acid (3p)

^{1}H-NMR (500 MHz, DMSO-d_6)

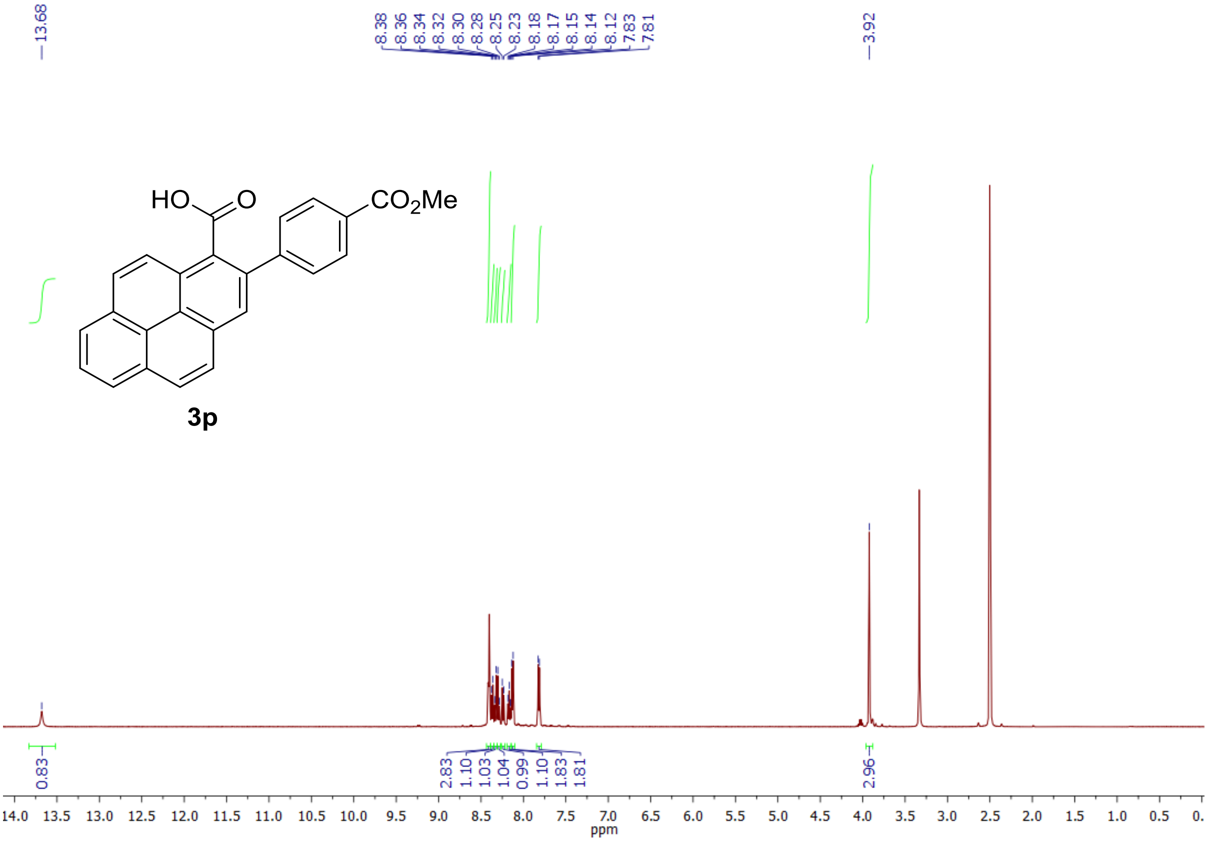

^{13}C-NMR (125 MHz, DMSO-d_6)

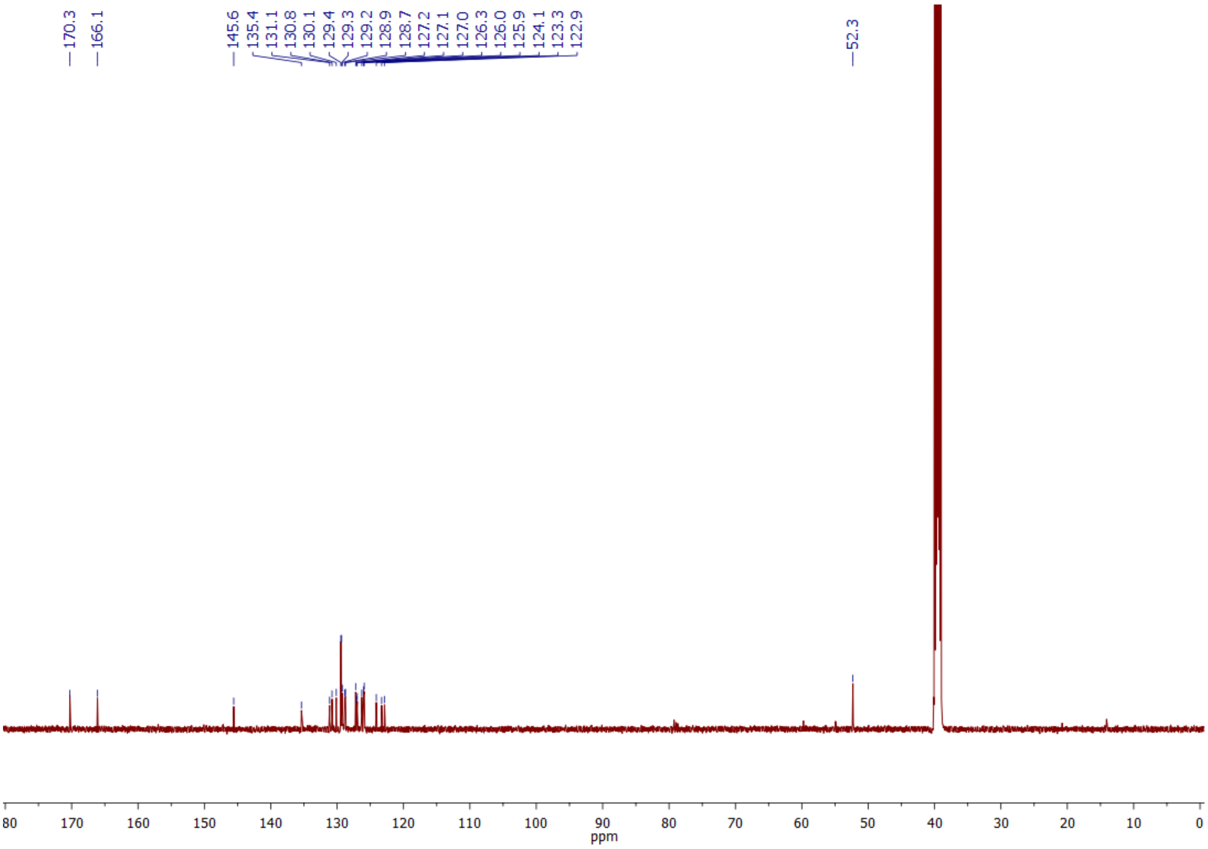

2-(Thiophen-2-yl)pyrene-1-carboxylic acid (3q)

^{1}H-NMR (500 MHz, DMSO-d$_6$)

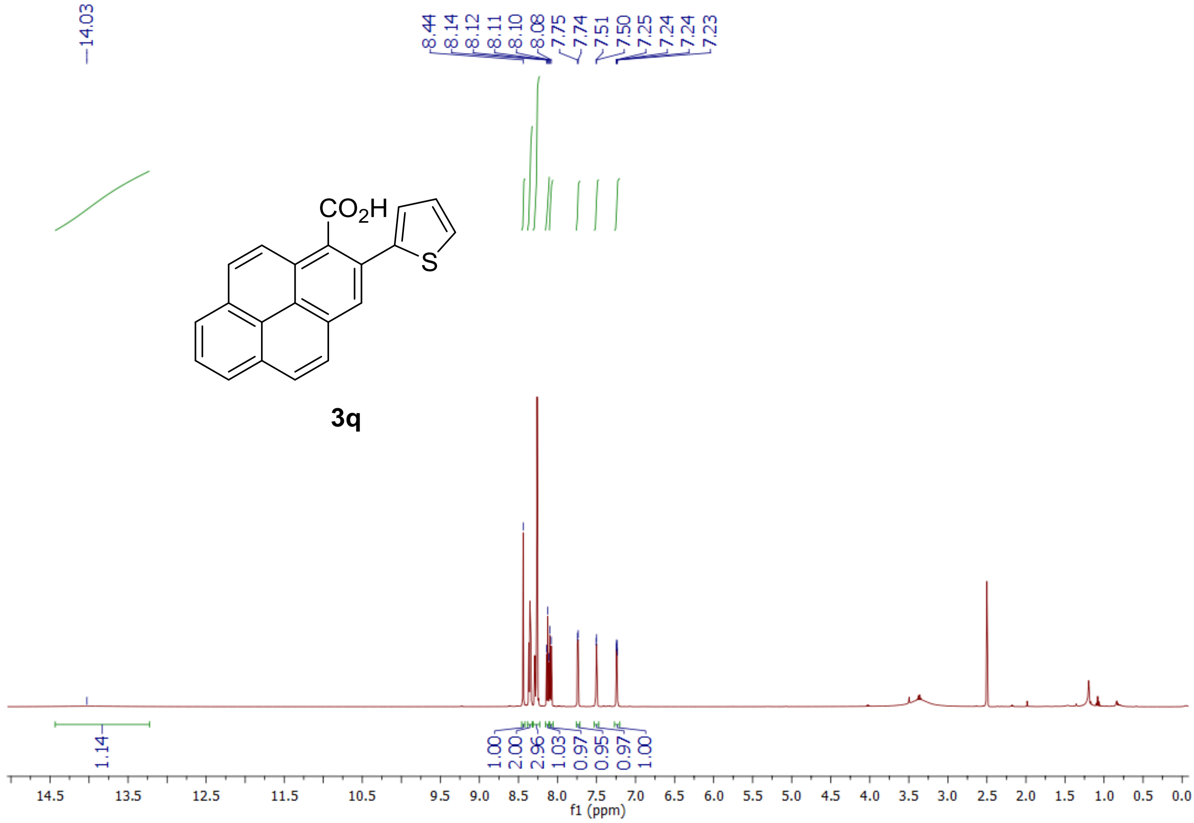

^{13}C-NMR (125 MHz, DMSO-d$_6$)

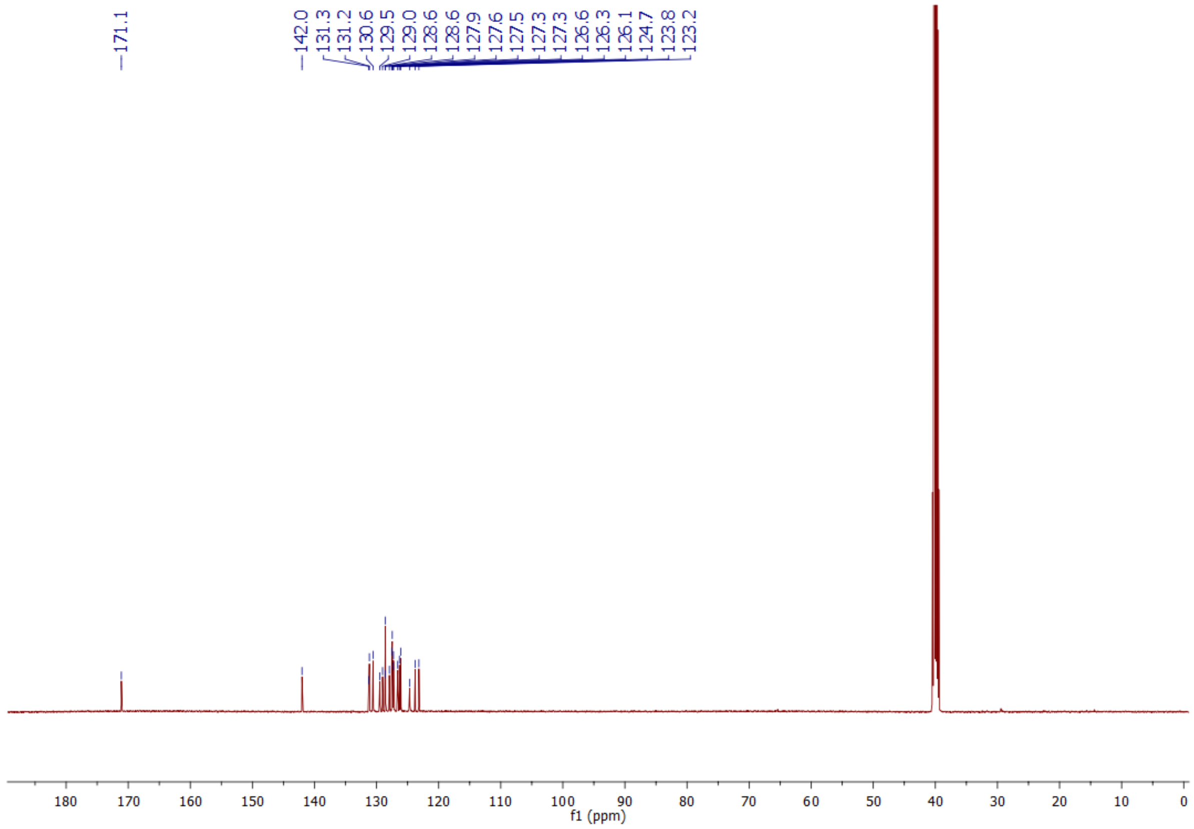

2-(5-Methylthiophen-2-yl)pyrene-1-carboxylic acid (3r)

^{1}H-NMR (500 MHz, DMSO-d_6)

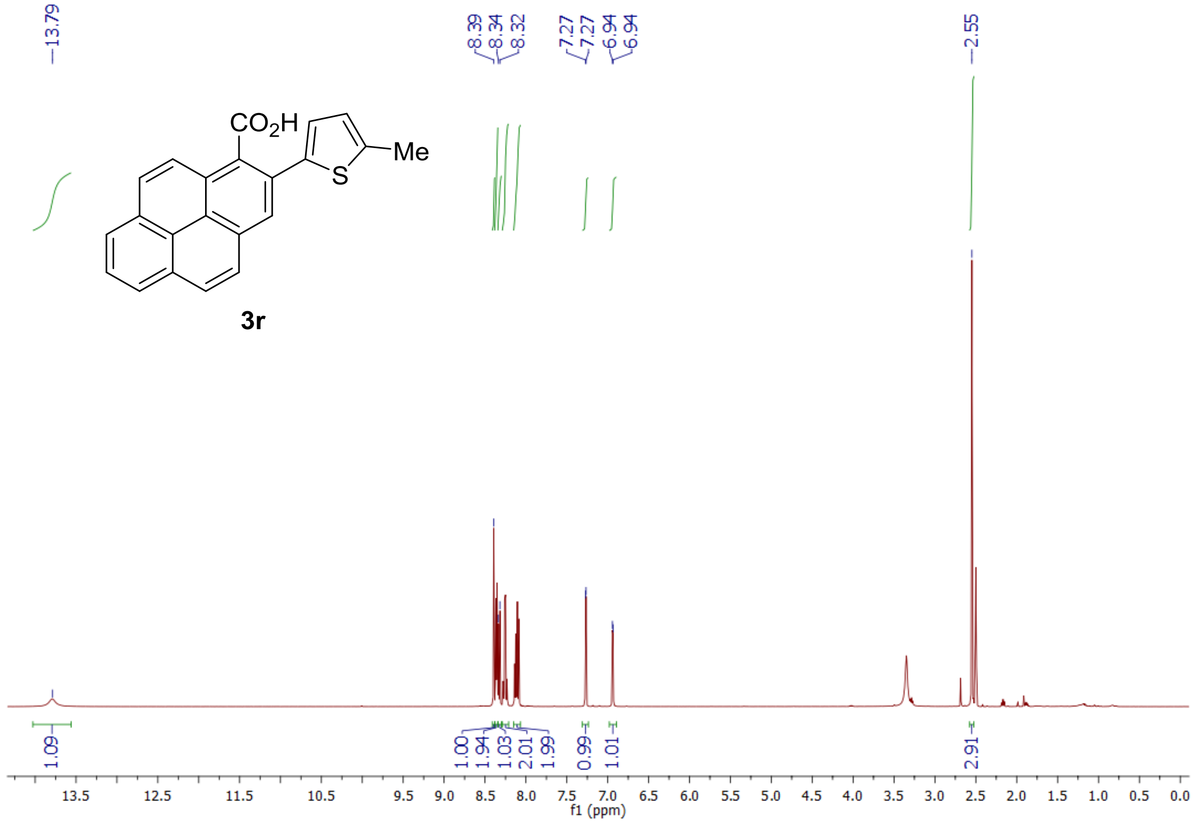

^{13}C-NMR (125 MHz, DMSO-d_6)

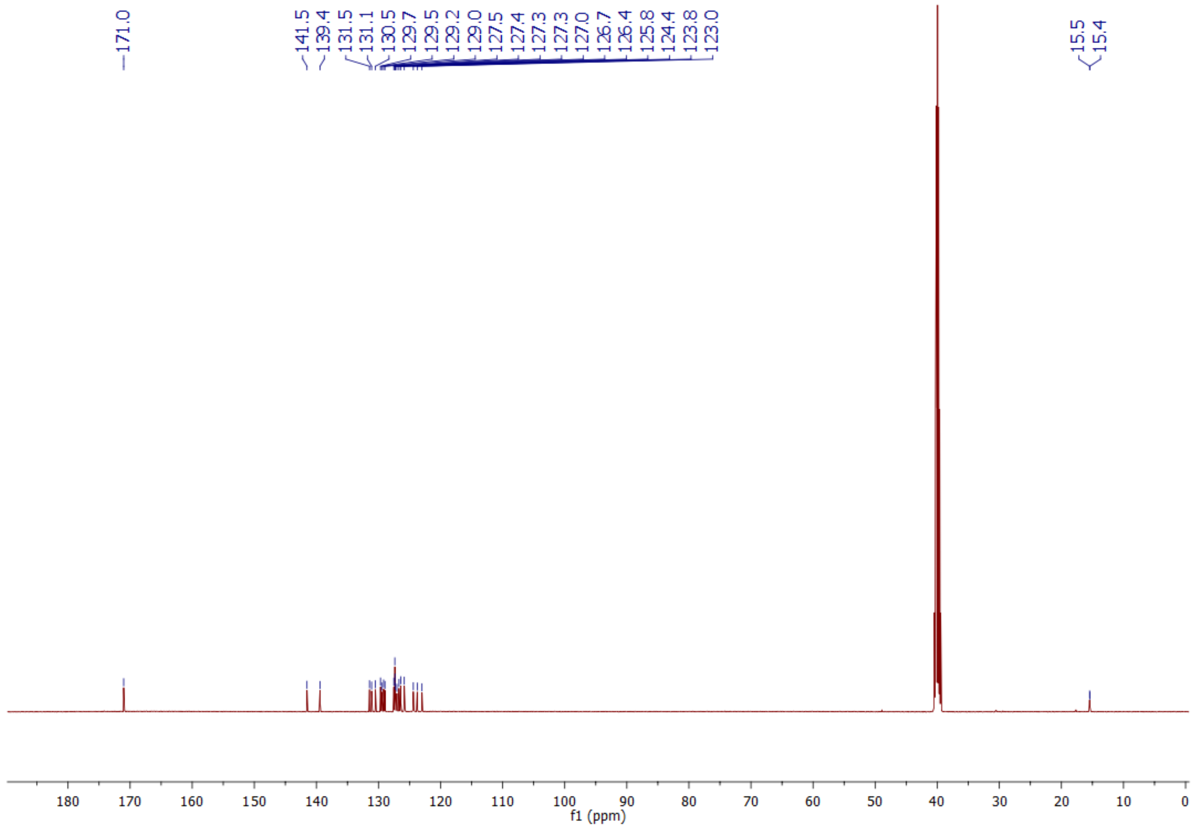

2-(1-Methylindol-5-yl)pyrene-1-carboxylic acid (3s)

^{1}H-NMR (500 MHz, DMSO-d_6)

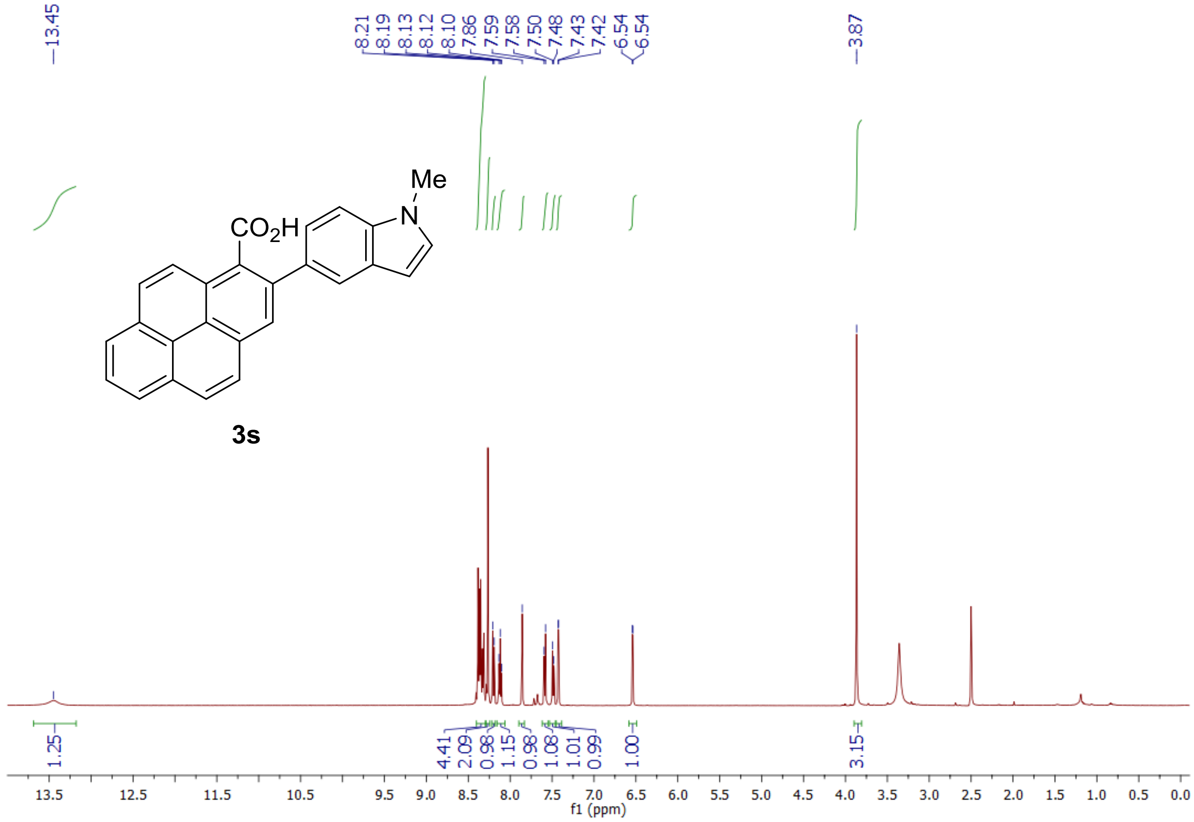

^{13}C-NMR (125 MHz, DMSO-d_6)

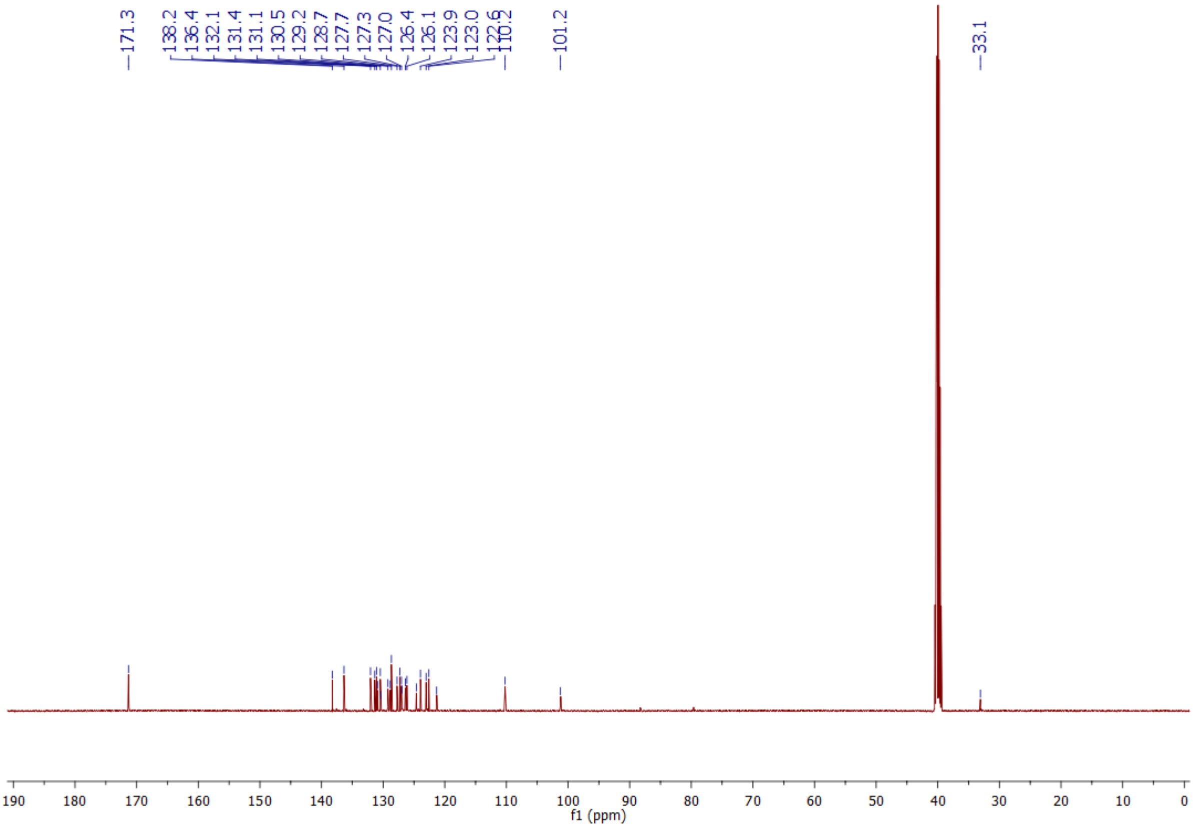

2-(2,6-Dichloropyridin-4-yl)pyrene-1-carboxylic acid (3t)

^{1}H-NMR (500 MHz, DMSO-d$_6$)

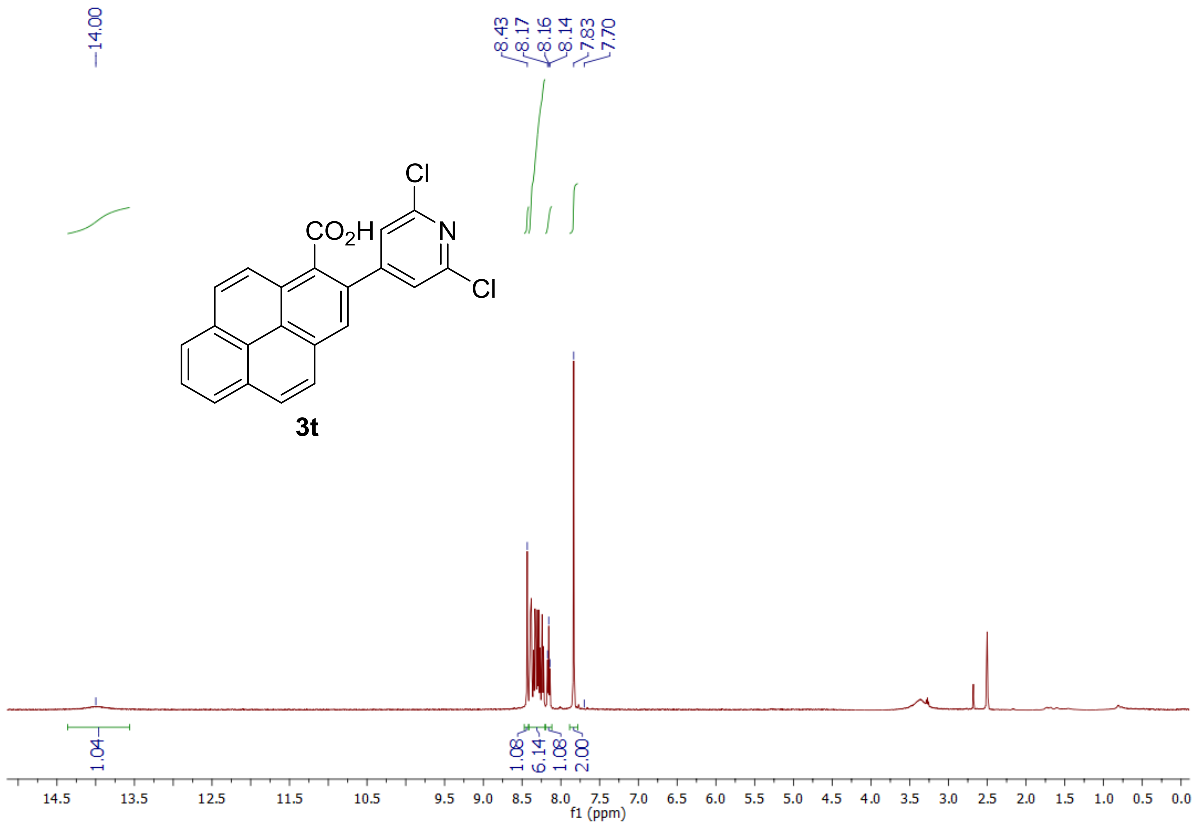

^{13}C-NMR (125 MHz, DMSO-d$_6$)

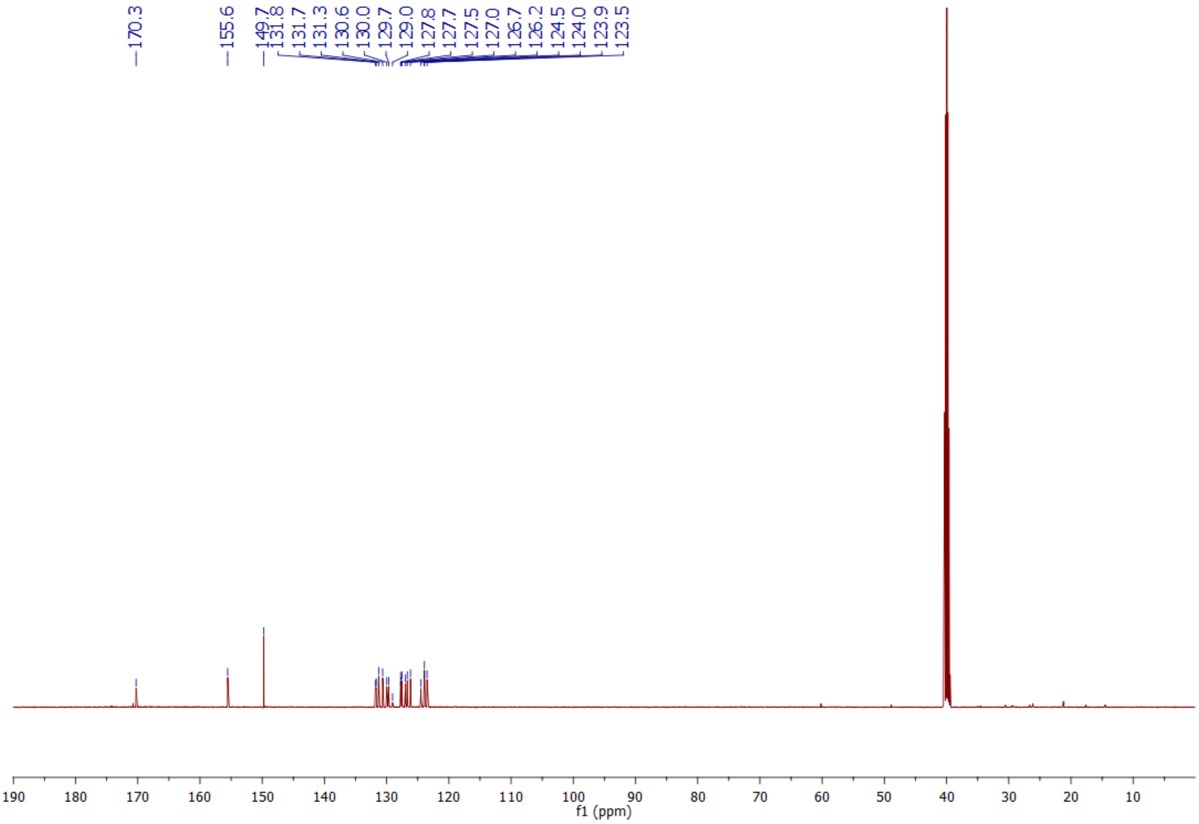

2-(3,5-Dimethylphenyl)-1-iodopyrene (4a)

^{1}H-NMR (400 MHz, $CDCl_3$)

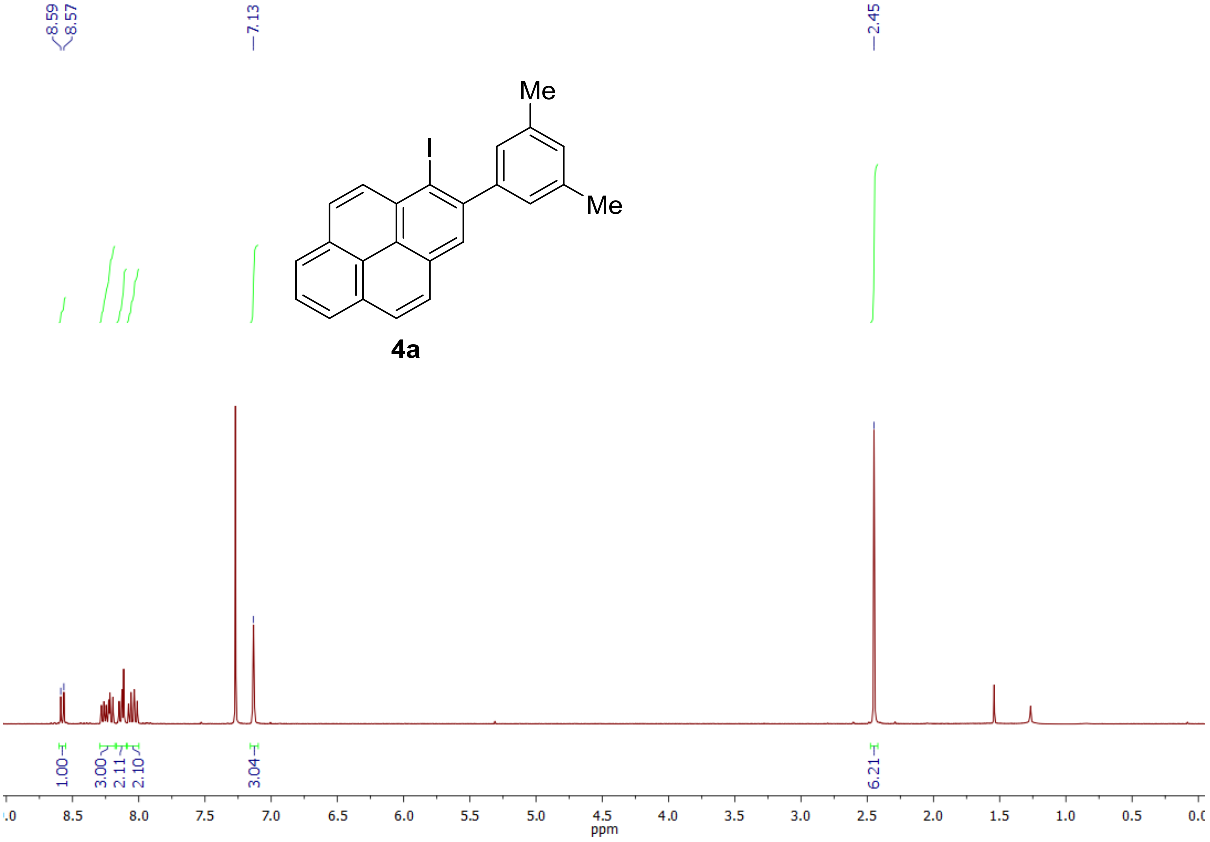

^{13}C-NMR (100 MHz, $CDCl_3$)

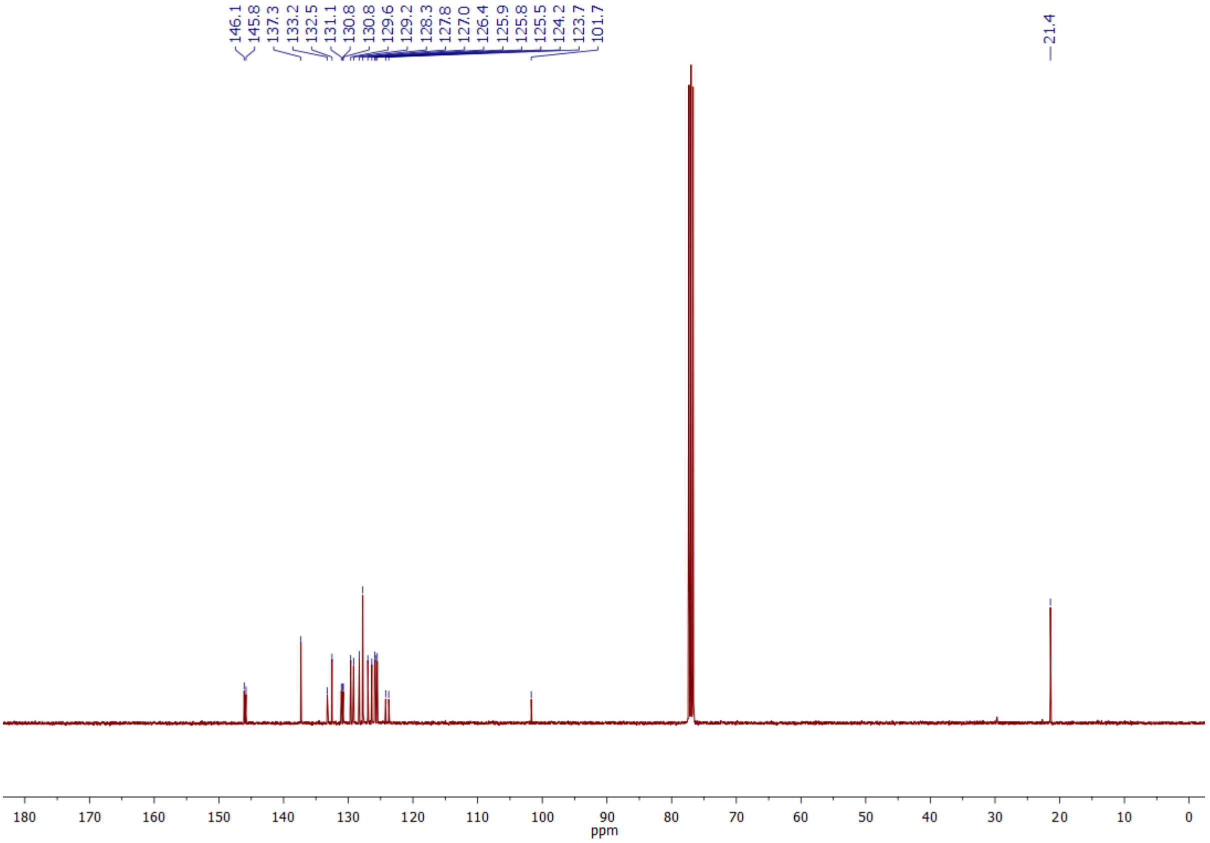

1-Iodo-2-(4-methylphenyl)pyrene (4b)

^{1}H-NMR (400 MHz, $CDCl_3$)

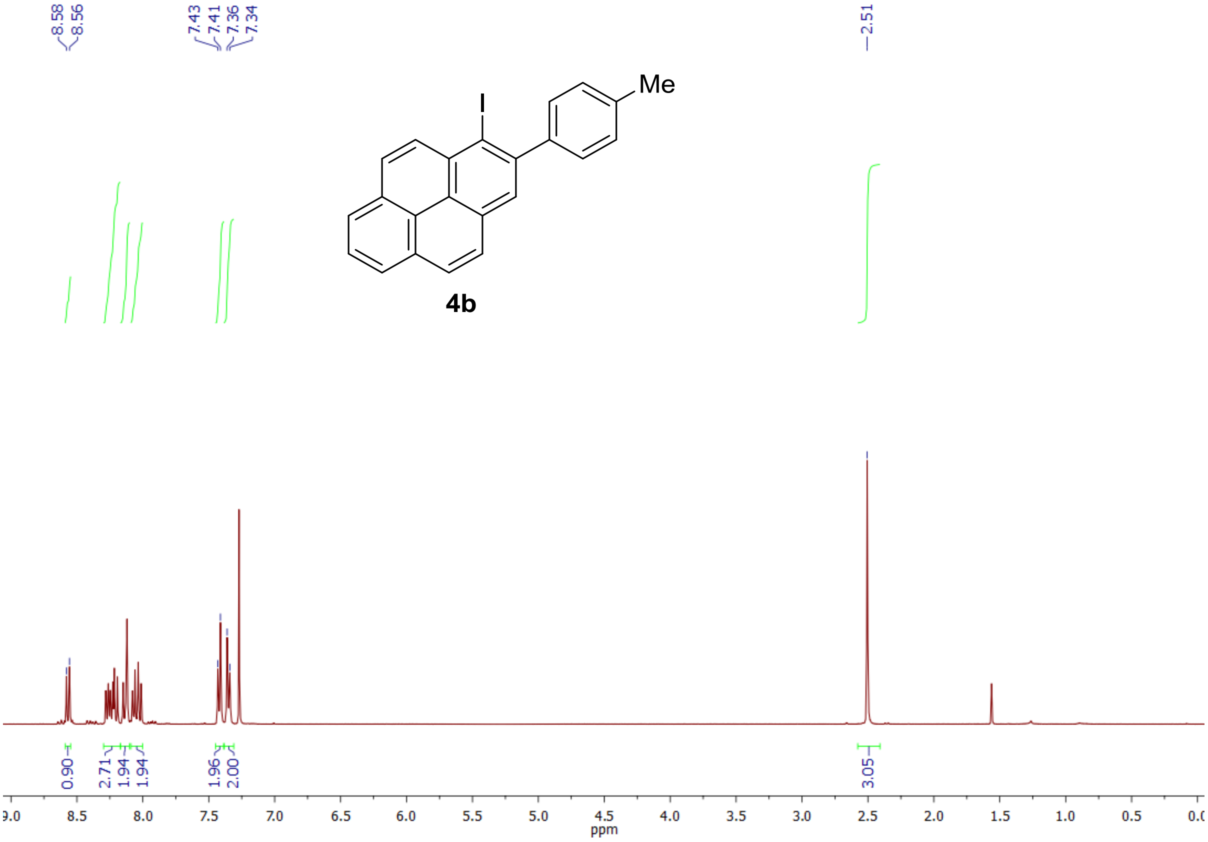

^{13}C-NMR (125 MHz, $CDCl_3$)

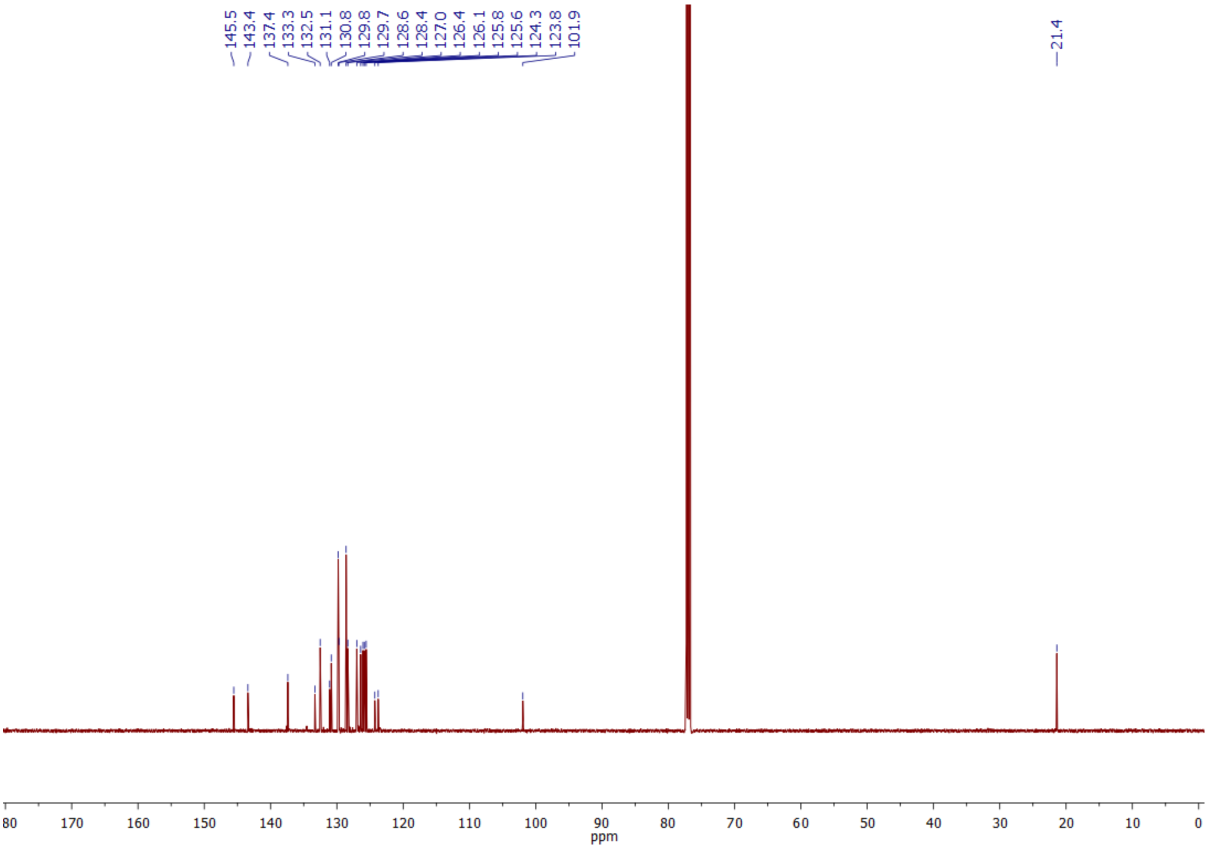

1-Iodo-2-(3-methylphenyl)pyrene (4c)

^{1}H-NMR (400 MHz, $CDCl_3$)

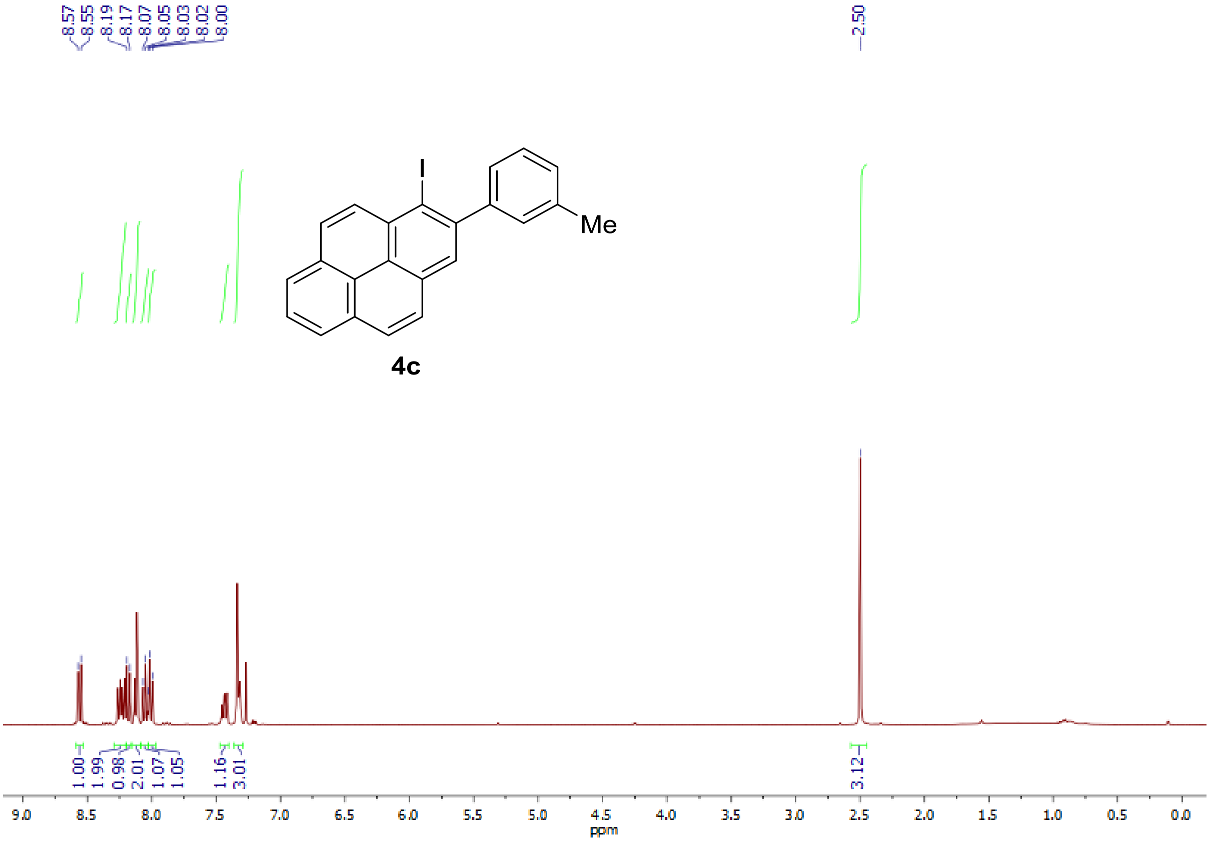

^{13}C-NMR (125 MHz, $CDCl_3$)

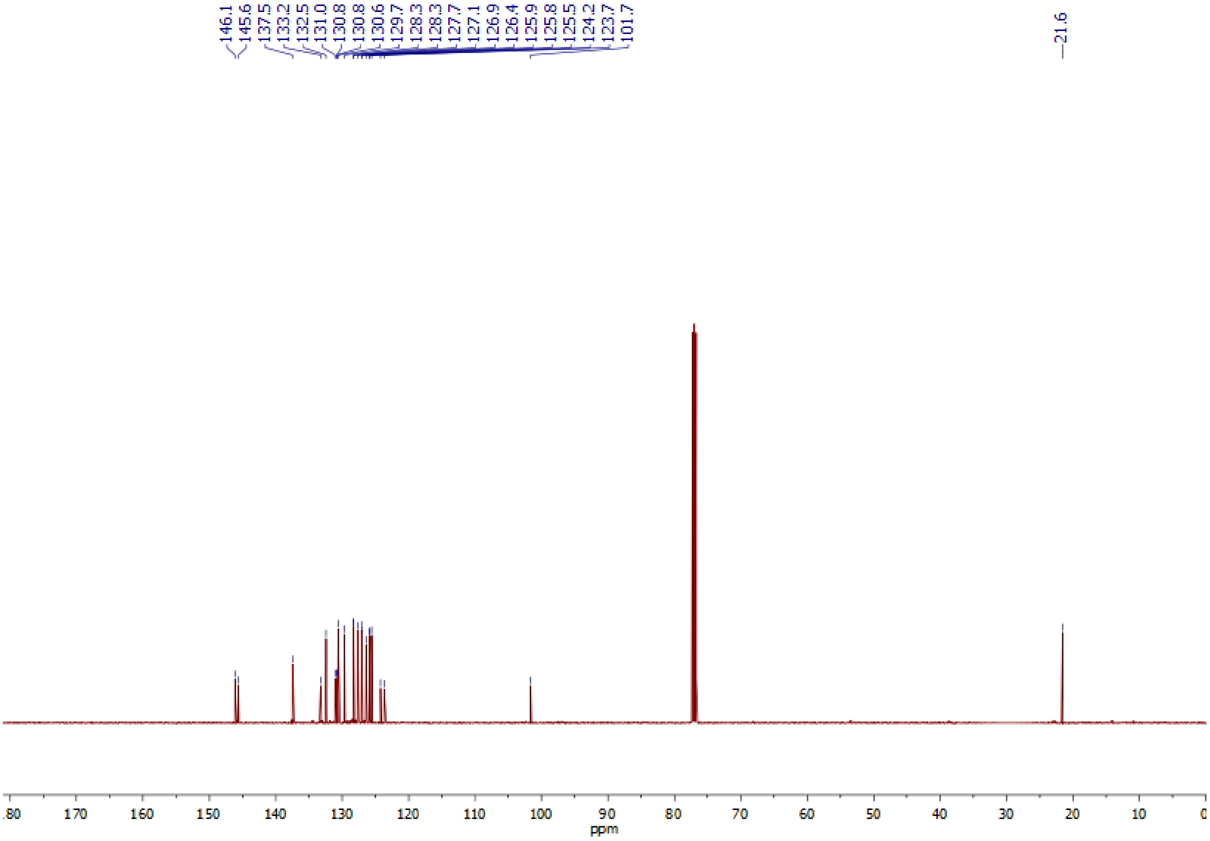

2-(4-*tert*-Butylphenyl)-1-iodopyrene (4d)

^{1}H-NMR (400 MHz, $CDCl_3$)

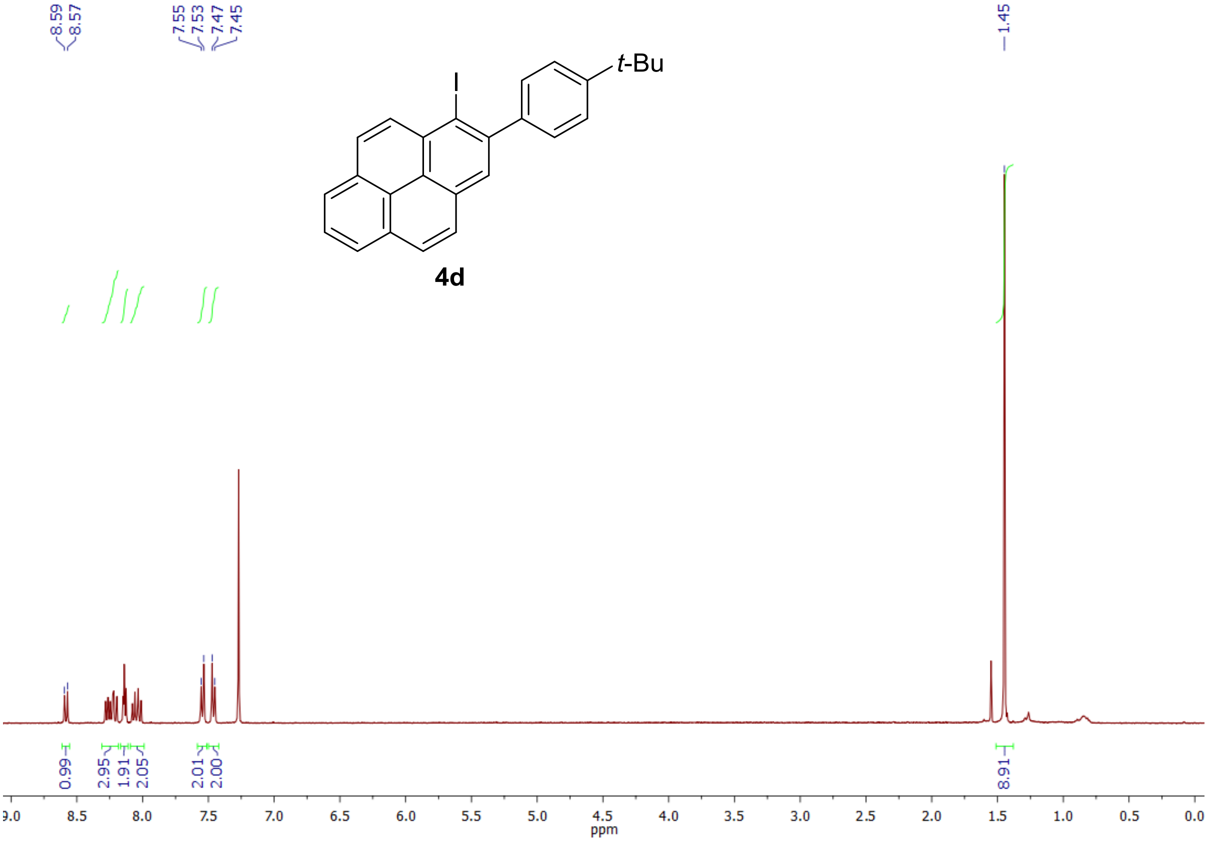

^{13}C-NMR (125 MHz, $CDCl_3$)

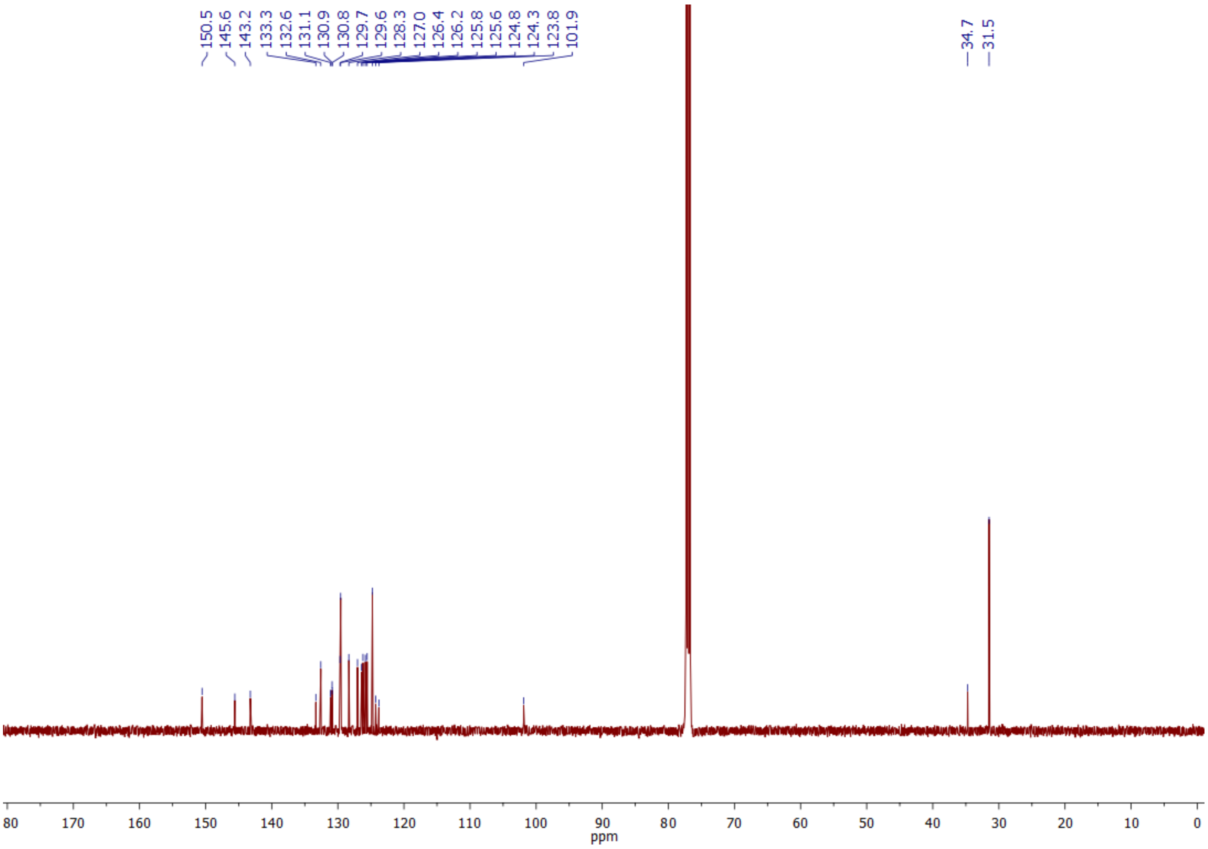

1-Iodo-2-(4-methoxyphenyl)pyrene (4e)

^{1}H-NMR (400 MHz, $CDCl_3$)

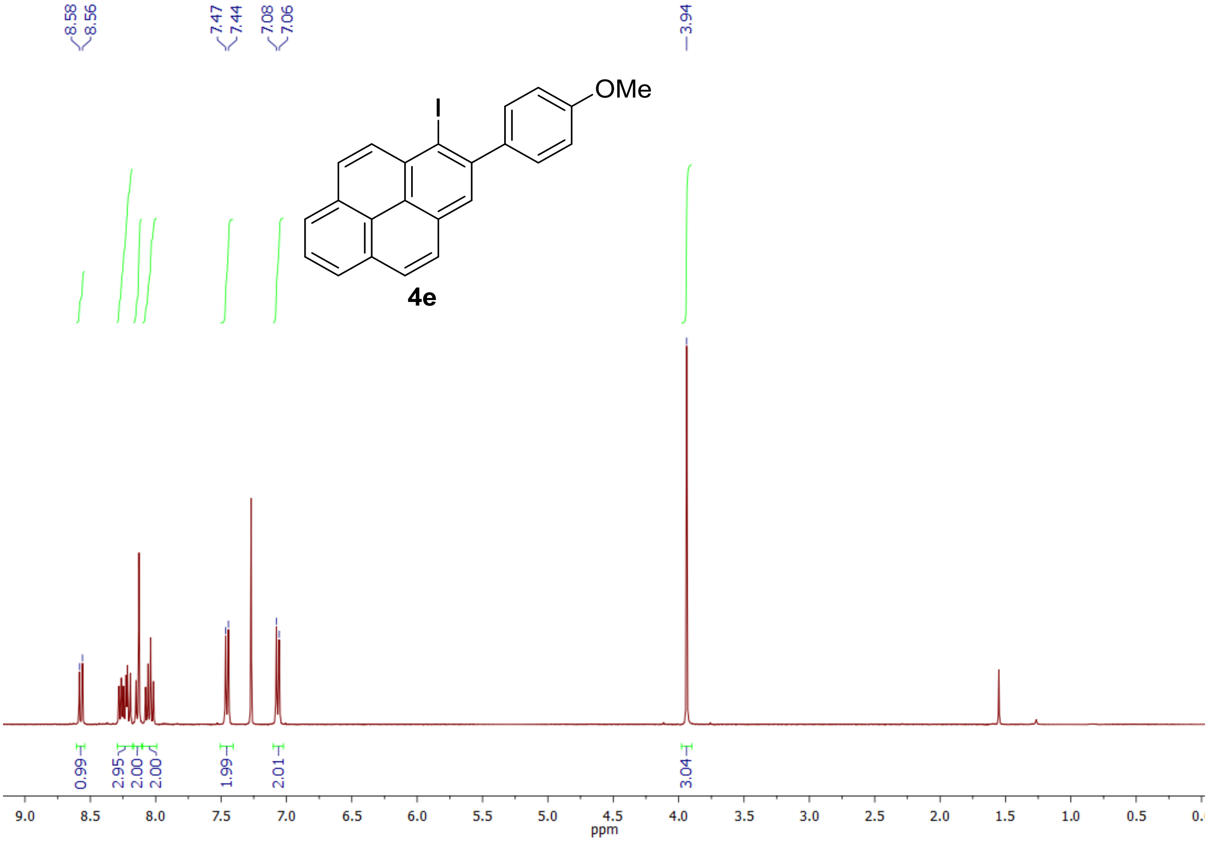

^{13}C-NMR (125 MHz, $CDCl_3$)

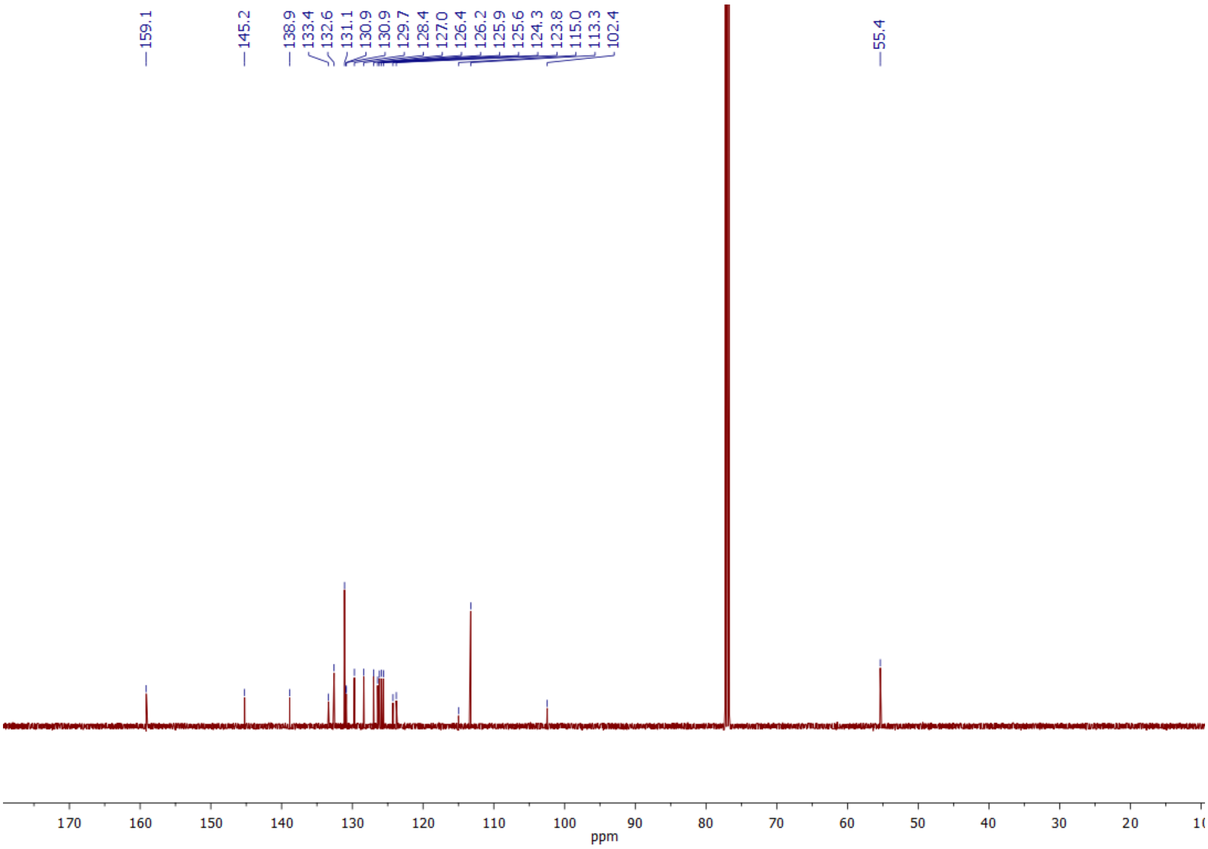

1-Iodo-2-phenylpyrene (4f)

^{1}H-NMR (400 MHz, $CDCl_3$)

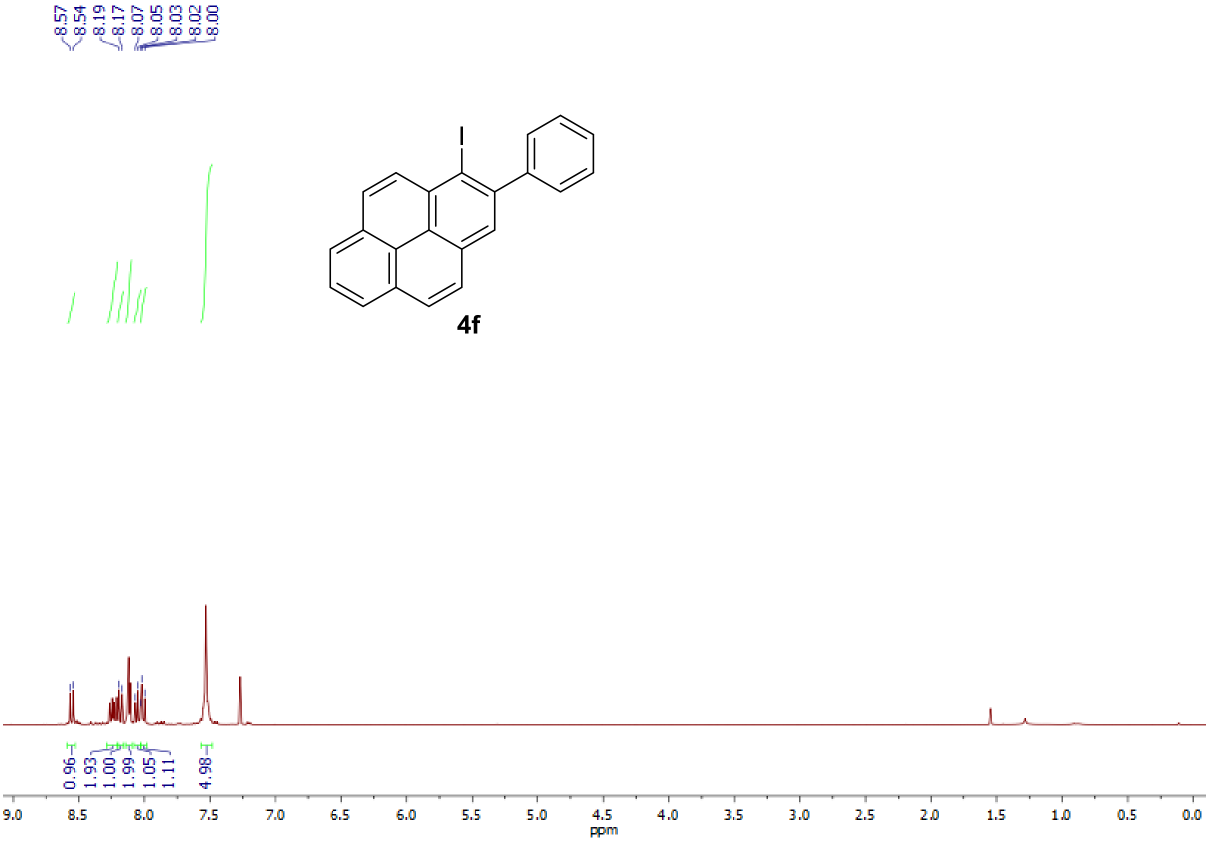

^{13}C-NMR (100 MHz, $CDCl_3$)

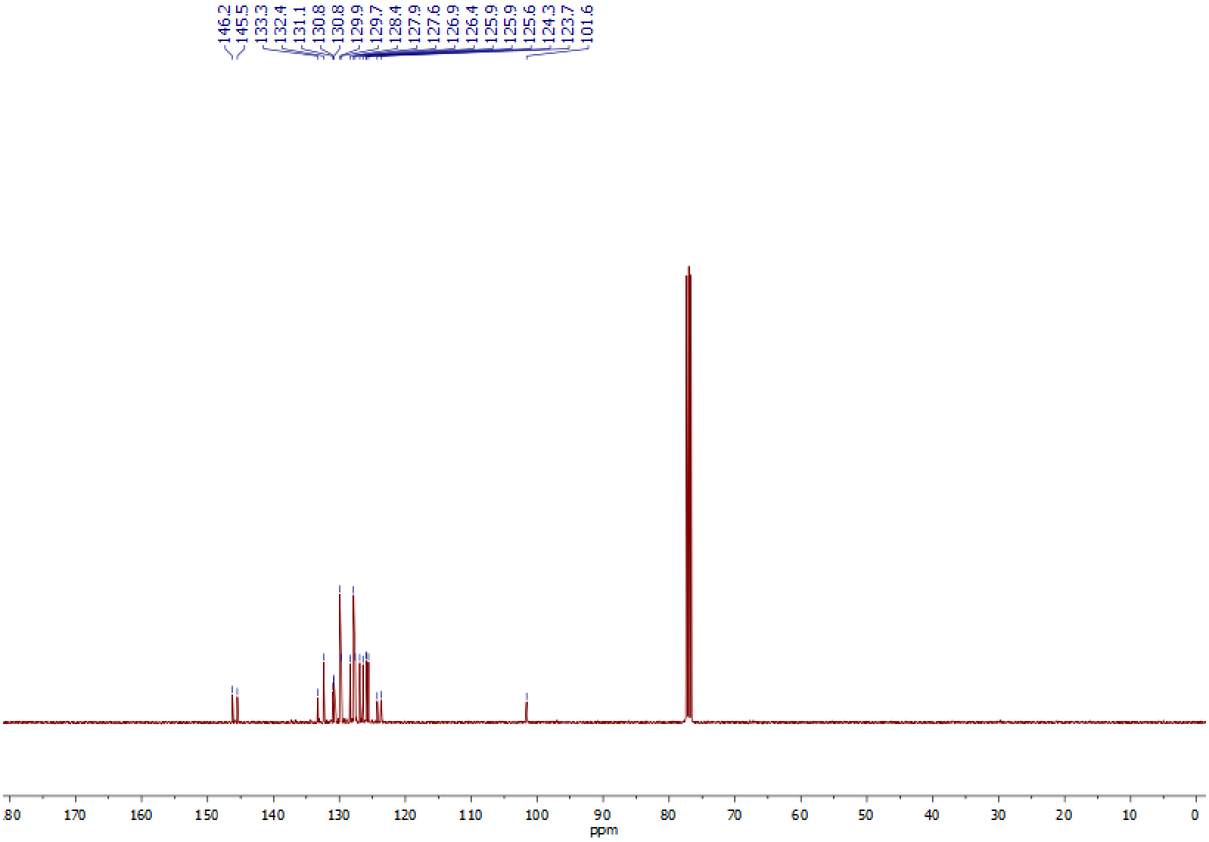

2-(4-Bromophenyl)-1-iodopyrene (4g)

^{1}H-NMR (400 MHz, $CDCl_3$)

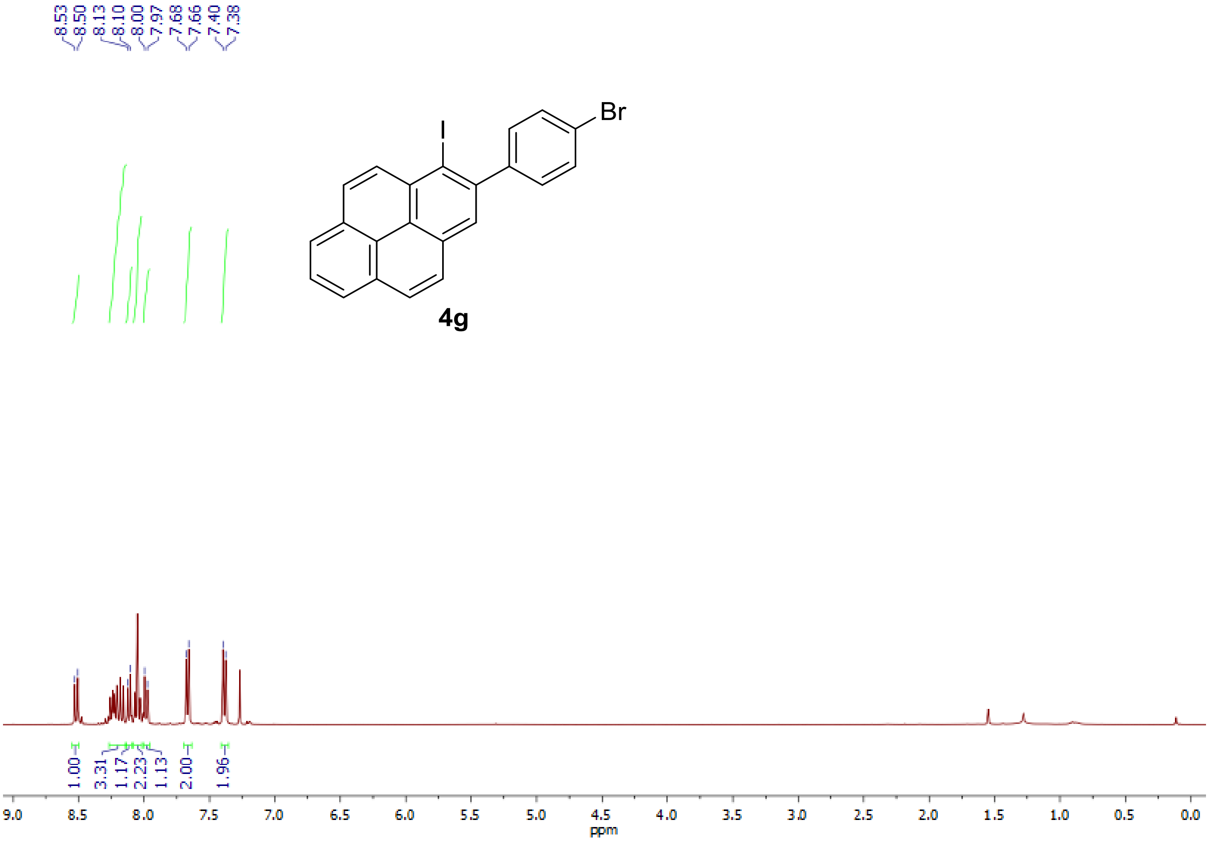

^{13}C-NMR (100 MHz, $CDCl_3$)

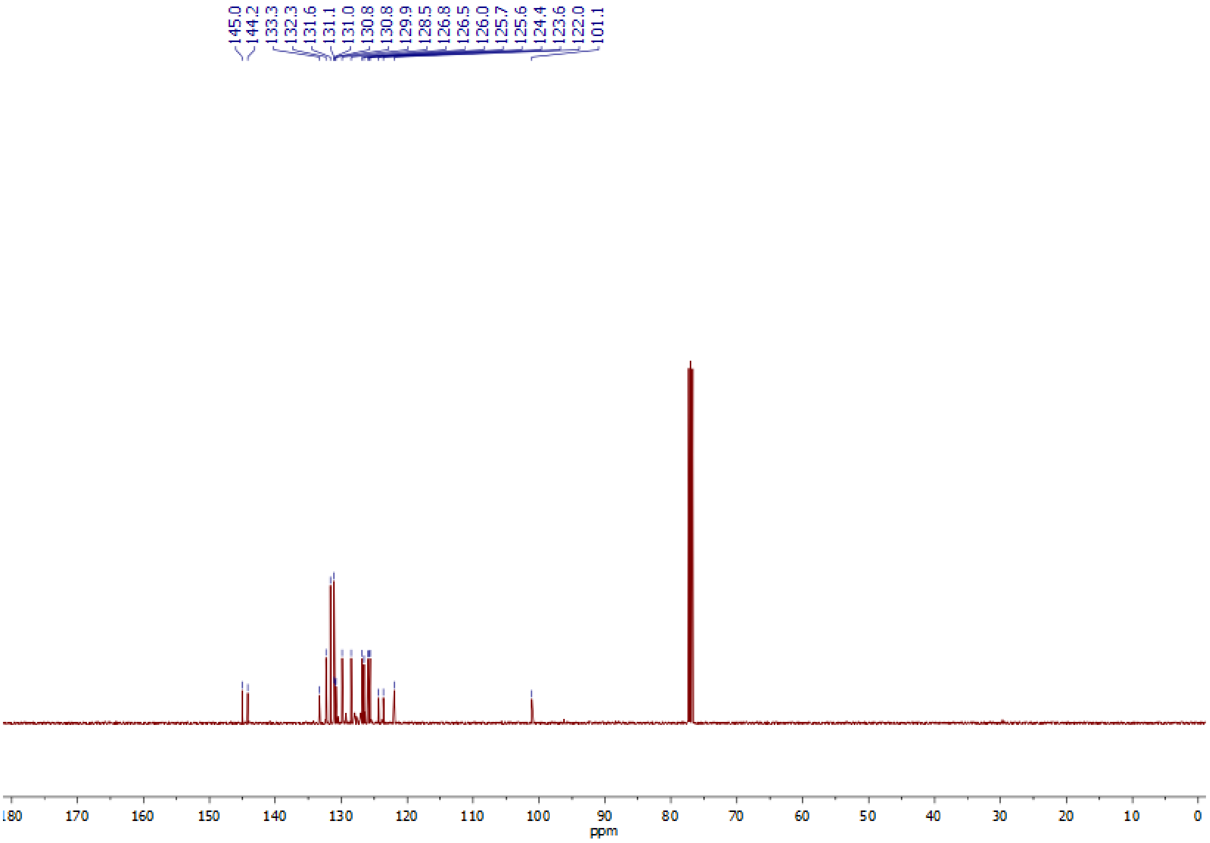

1-Iodo-2-(3-bromophenyl)pyrene (4h)

^{1}H-NMR (400 MHz, $CDCl_3$)

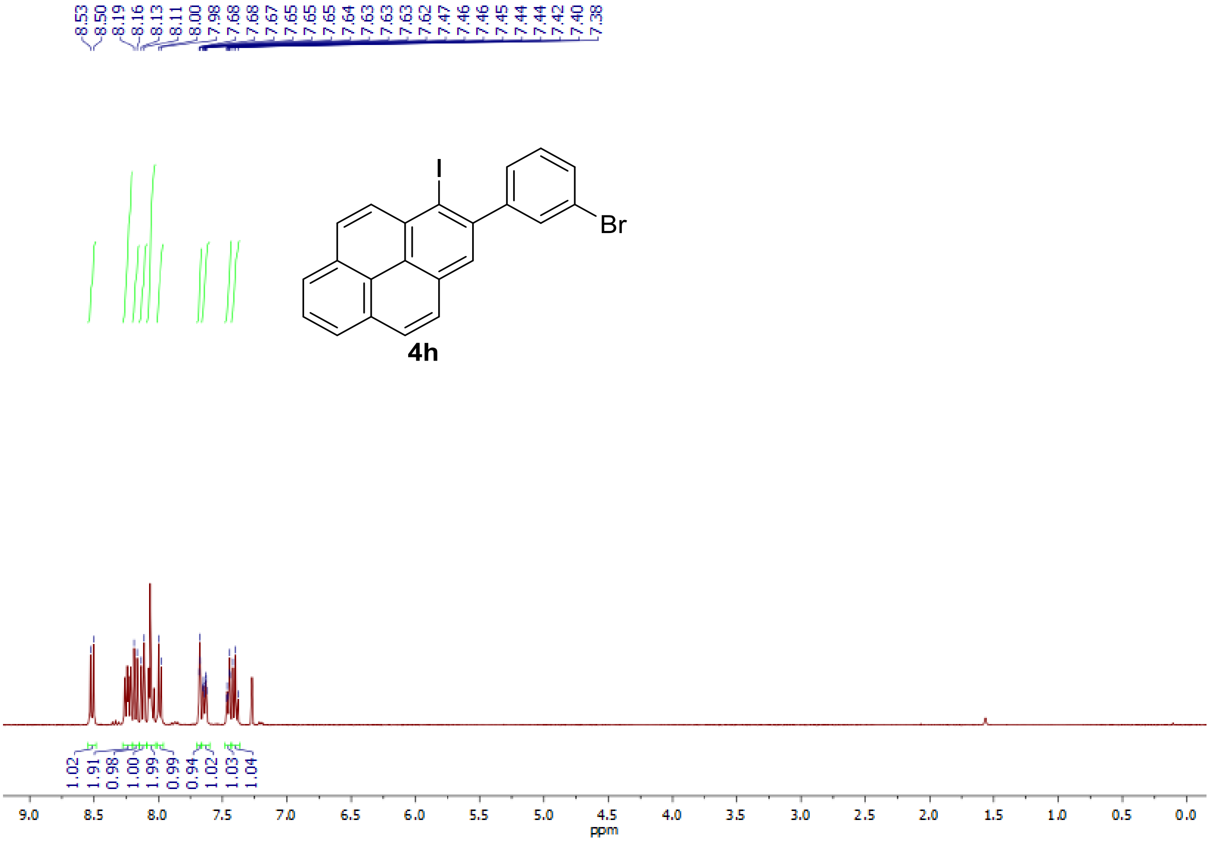

^{13}C-NMR (125 MHz, $CDCl_3$)

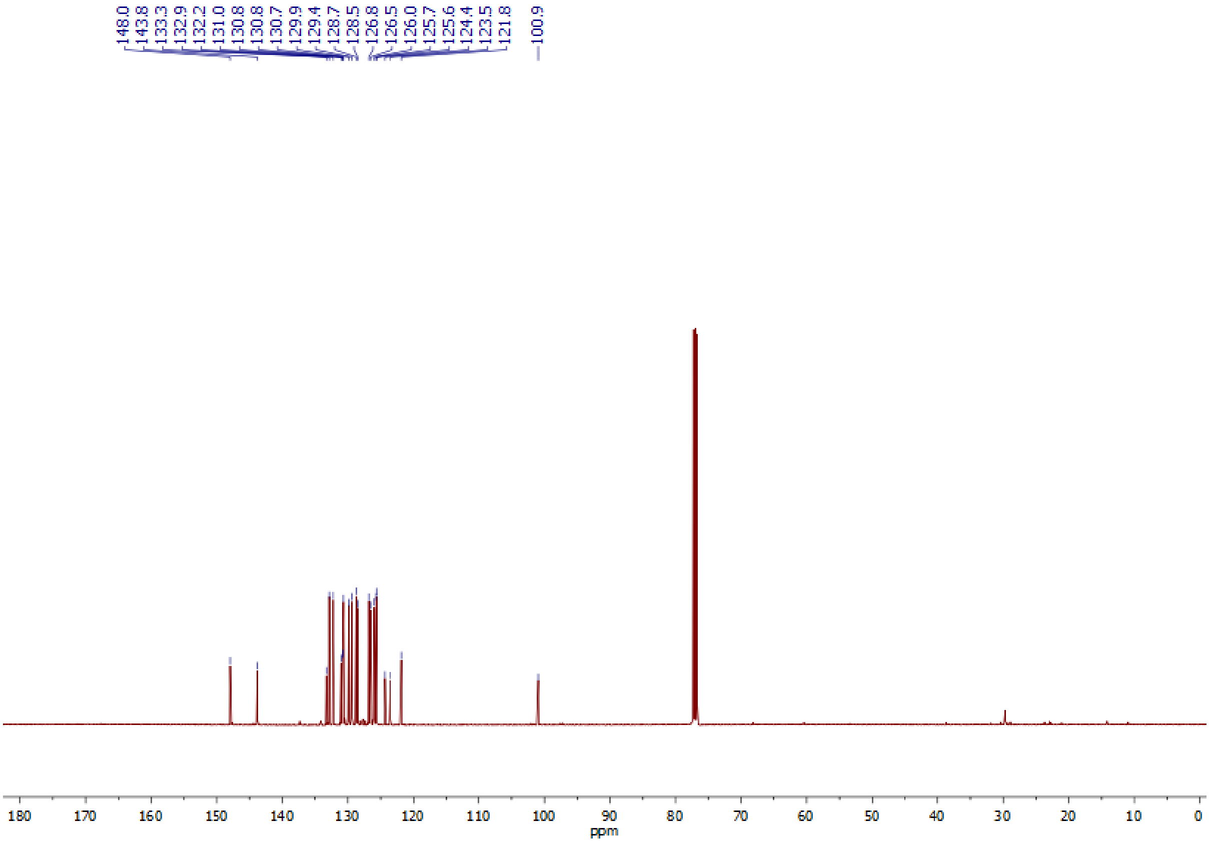

1-Iodo-2-(3-chlorophenyl)pyrene (4i)

^{1}H-NMR (400 MHz, $CDCl_3$)

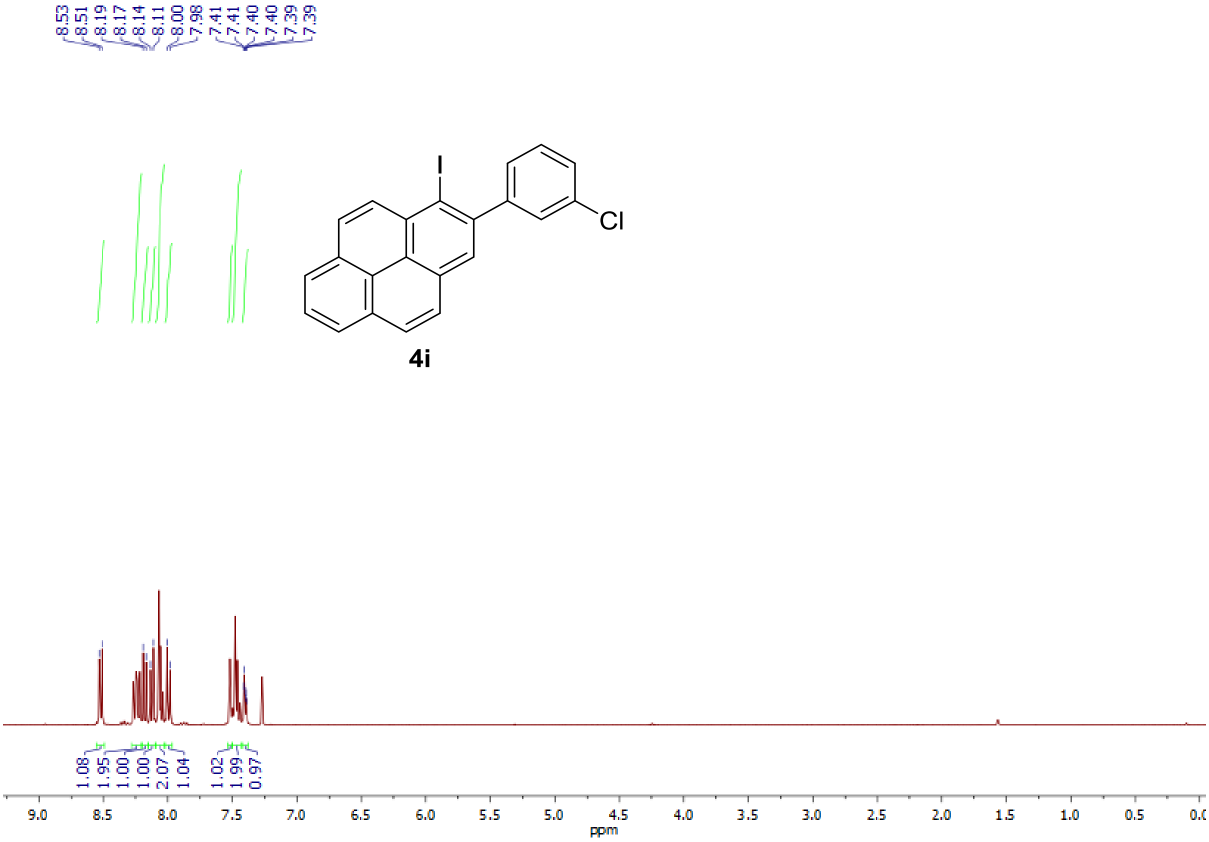

^{13}C-NMR (125 MHz, $CDCl_3$)

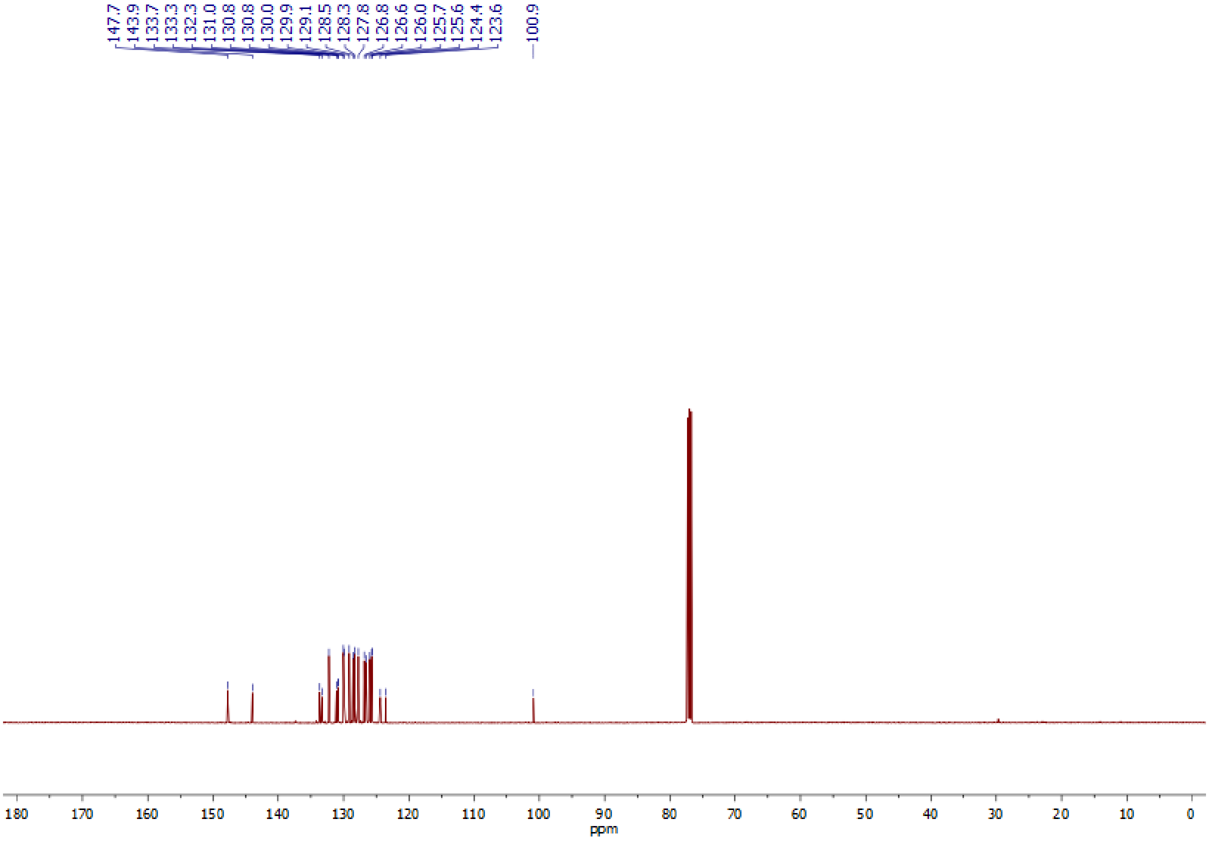

1-Iodo-2-(3,5-dichlorophenyl)pyrene (4j)

^{1}H-NMR (500 MHz, $CDCl_3$)

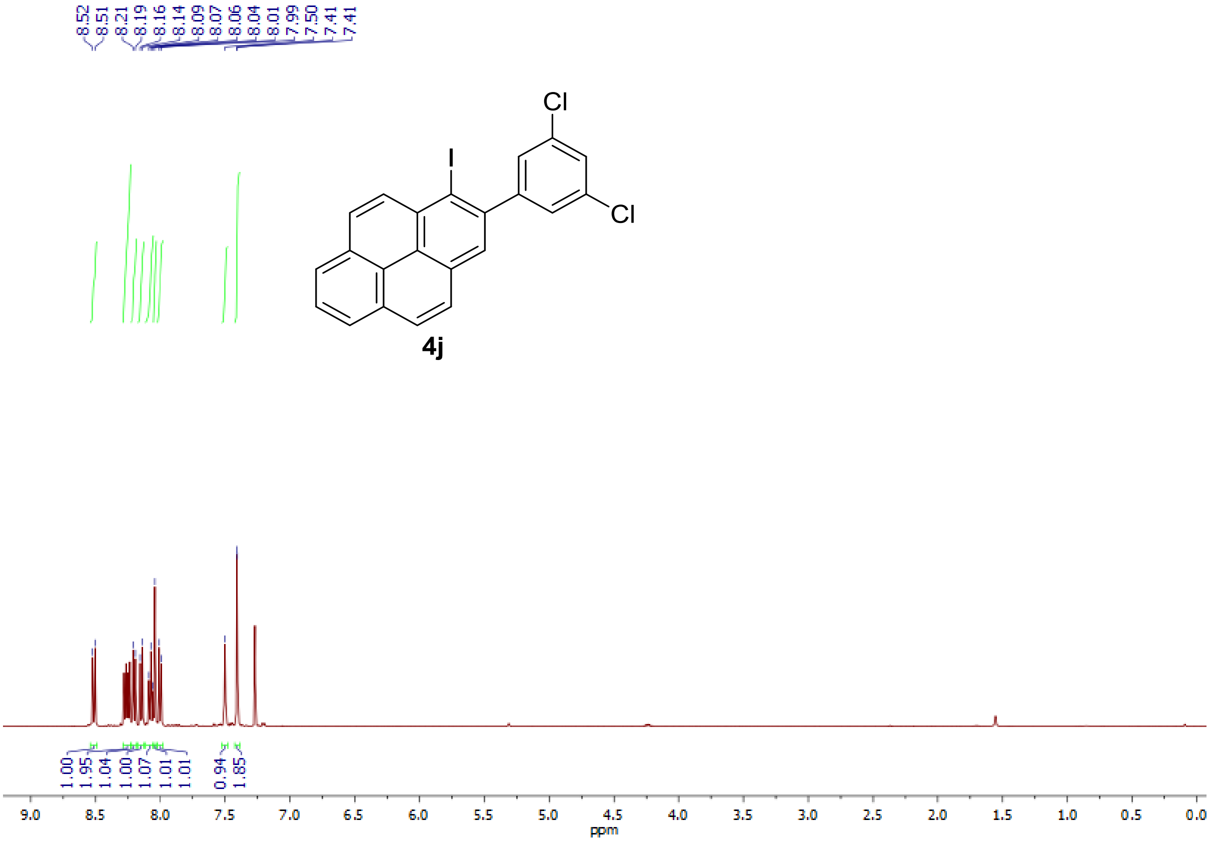

^{13}C-NMR (125 MHz, $CDCl_3$)

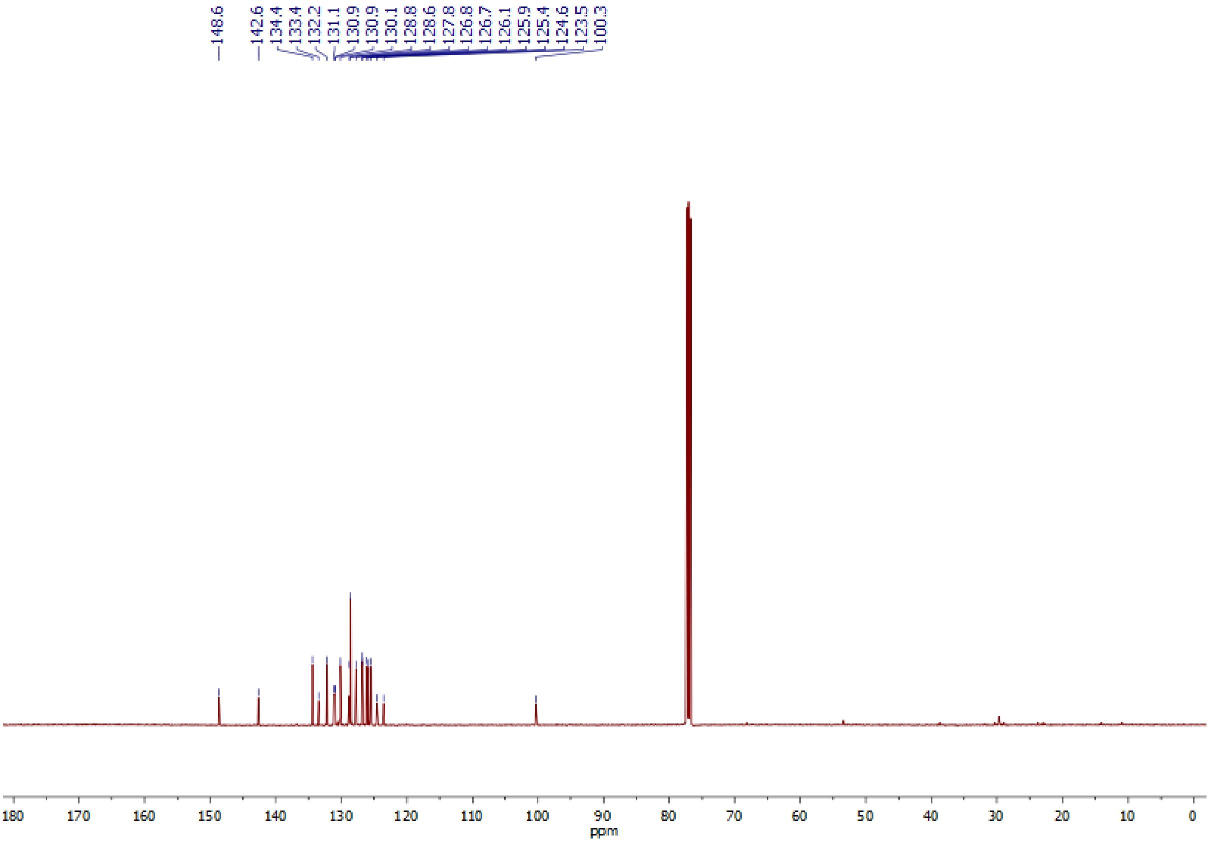

2-(3,5-Bis(trifluoromethyl)phenyl)-1-iodopyrene (4k)

^{1}H-NMR (400 MHz, $CDCl_3$)

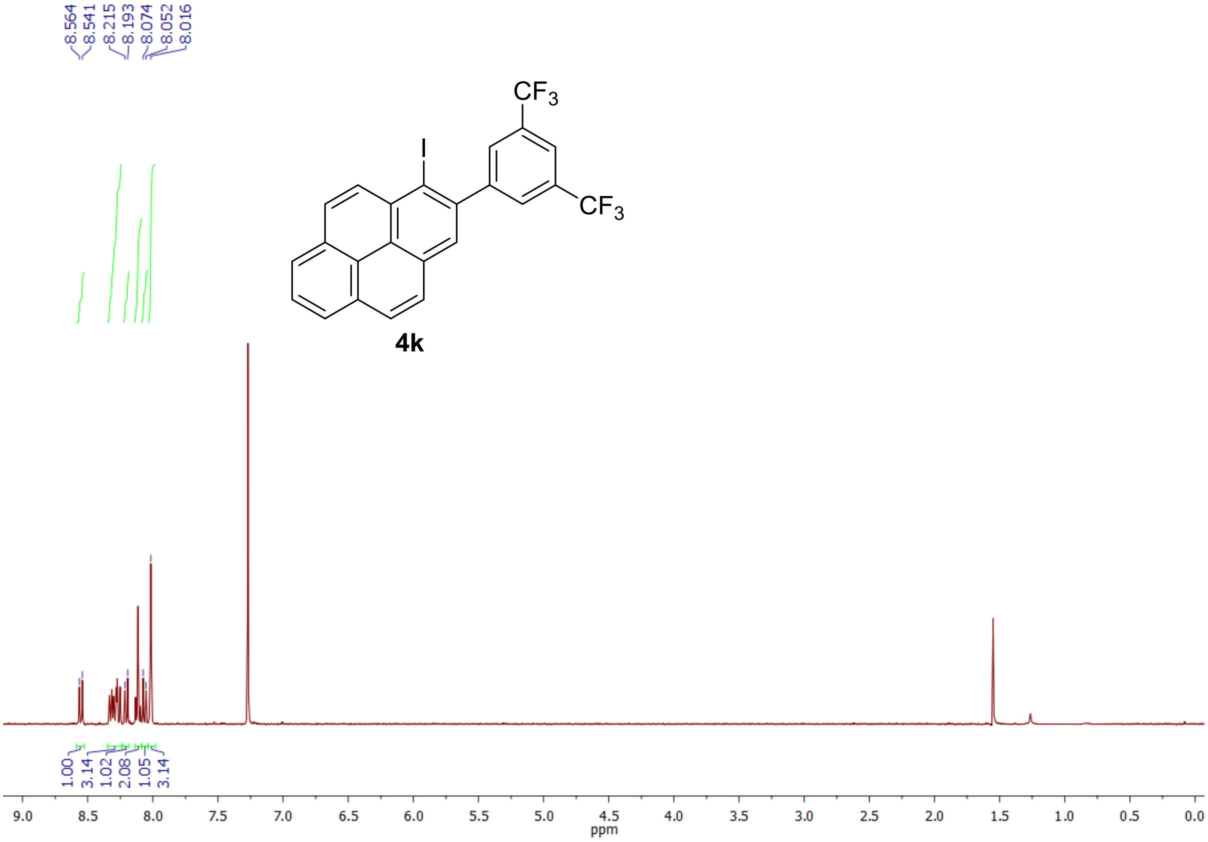

^{13}C-NMR (100 MHz, $CDCl_3$)

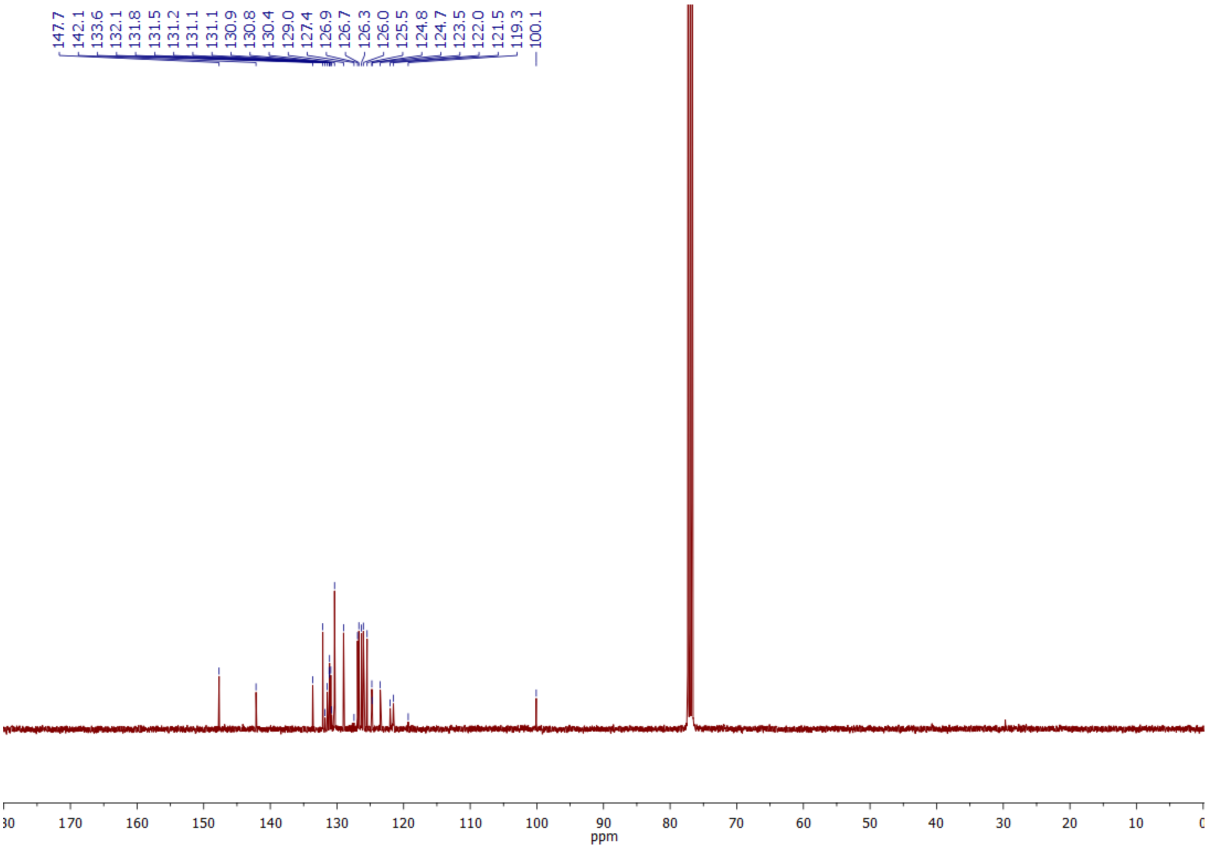

^{19}F-NMR (376 MHz, DMSO-d$_6$)

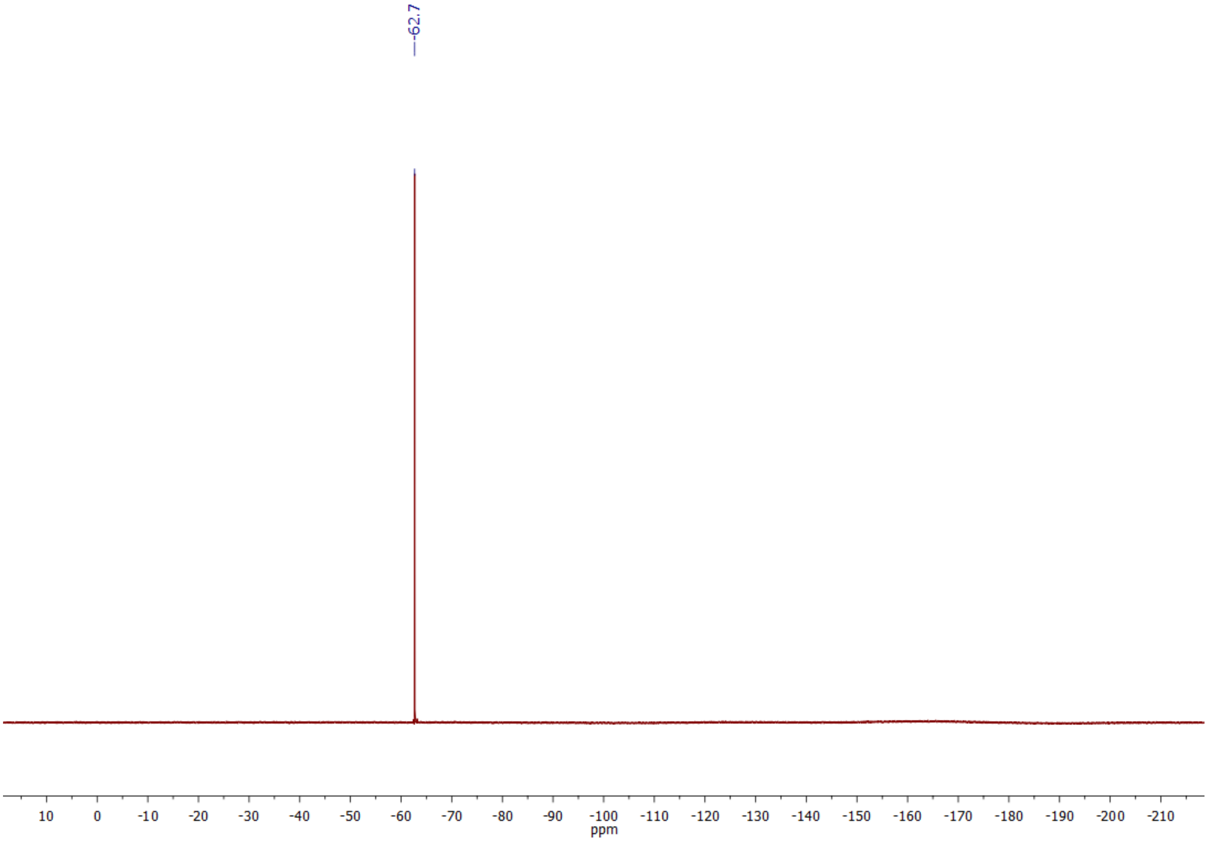

1-Iodo-2-(3-nitrophenyl)pyrene (4l)

^{1}H-NMR (400 MHz, $CDCl_3$)

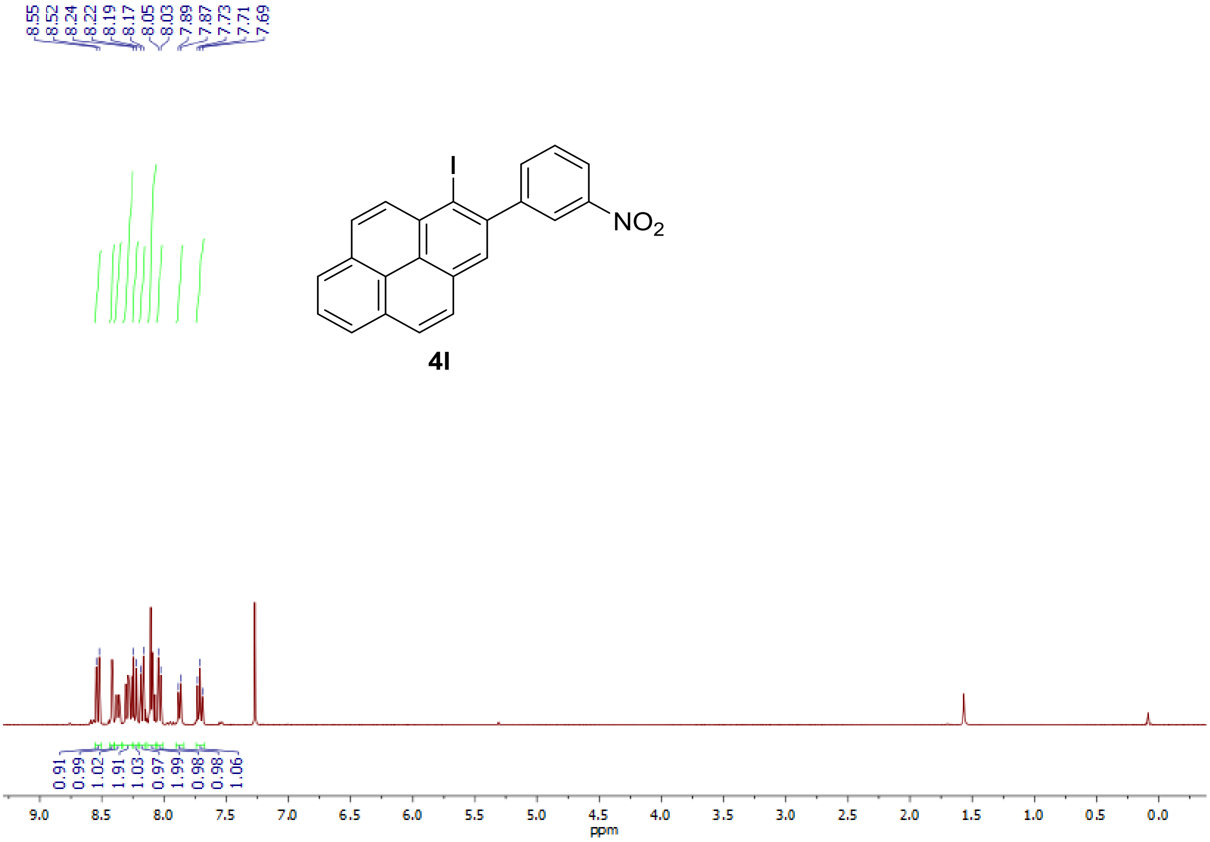

4l

^{13}C-NMR (125 MHz, $CDCl_3$)

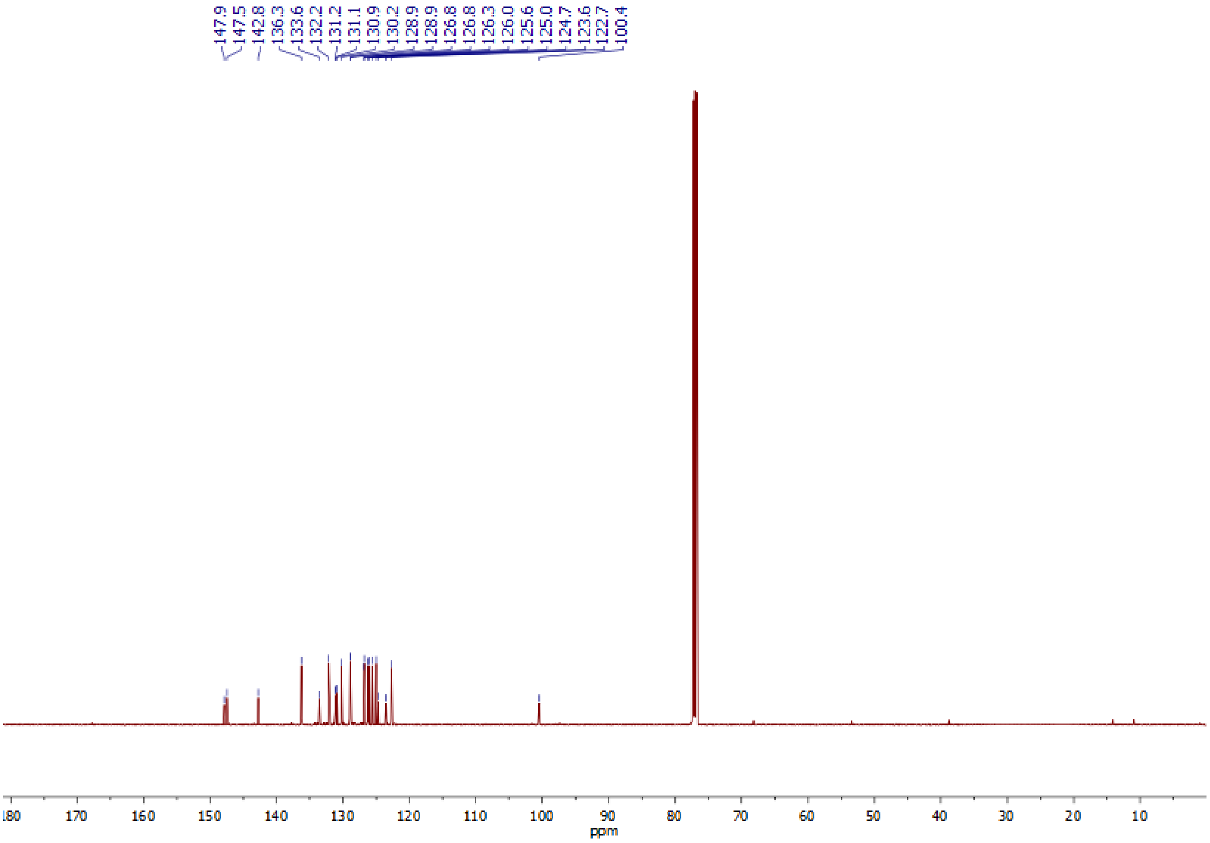

1-Iodo-2-(4-methoxyphenyl)pyrene (4m)

^{1}H-NMR (400 MHz, $CDCl_3$)

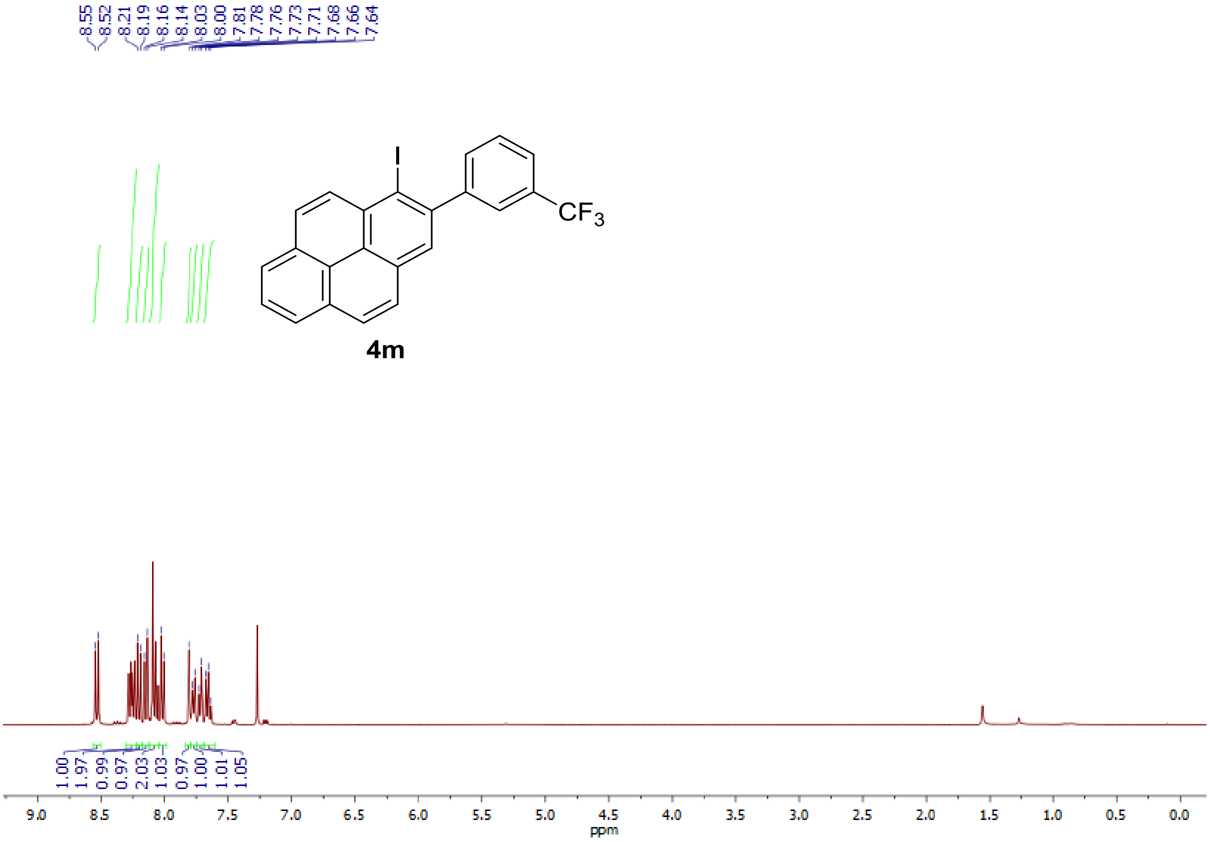

^{13}C-NMR (125 MHz, $CDCl_3$)

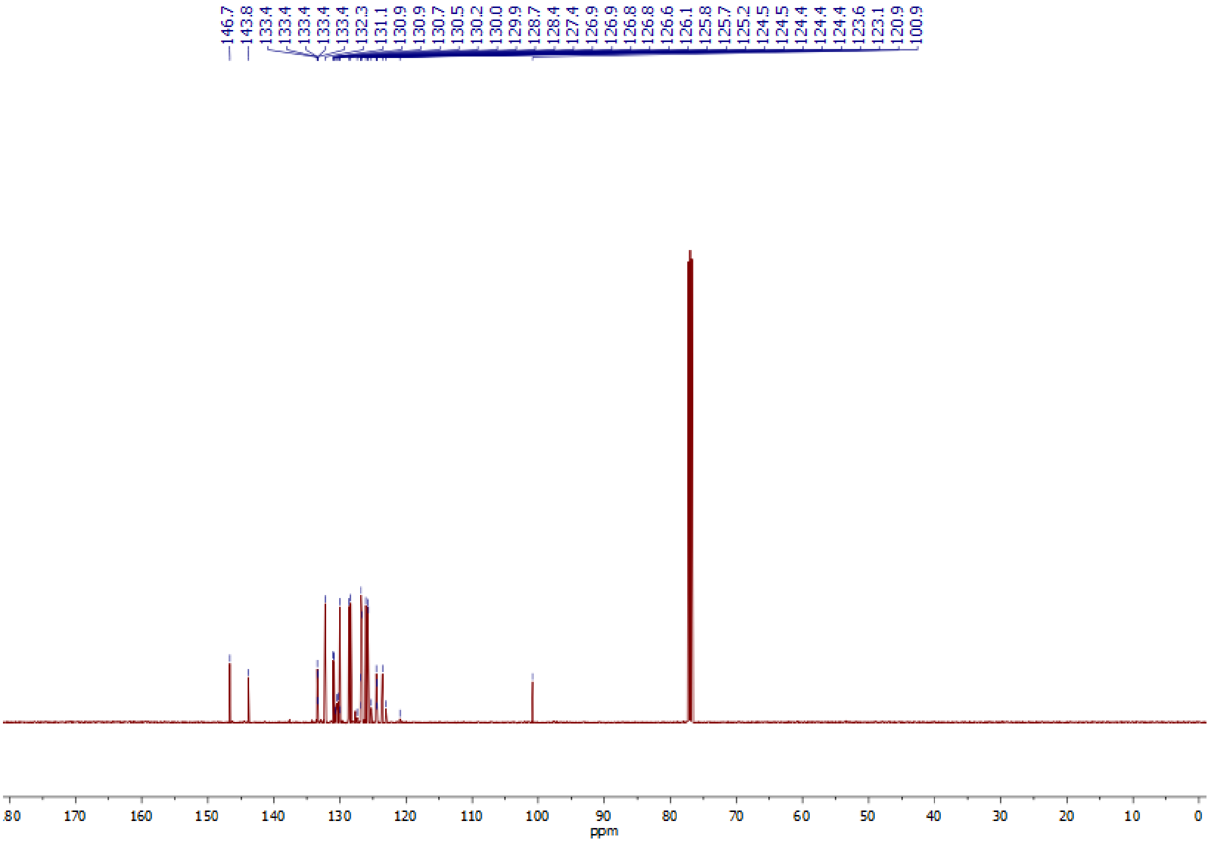

^{19}F-NMR (471 MHz, DMSO-d_6)

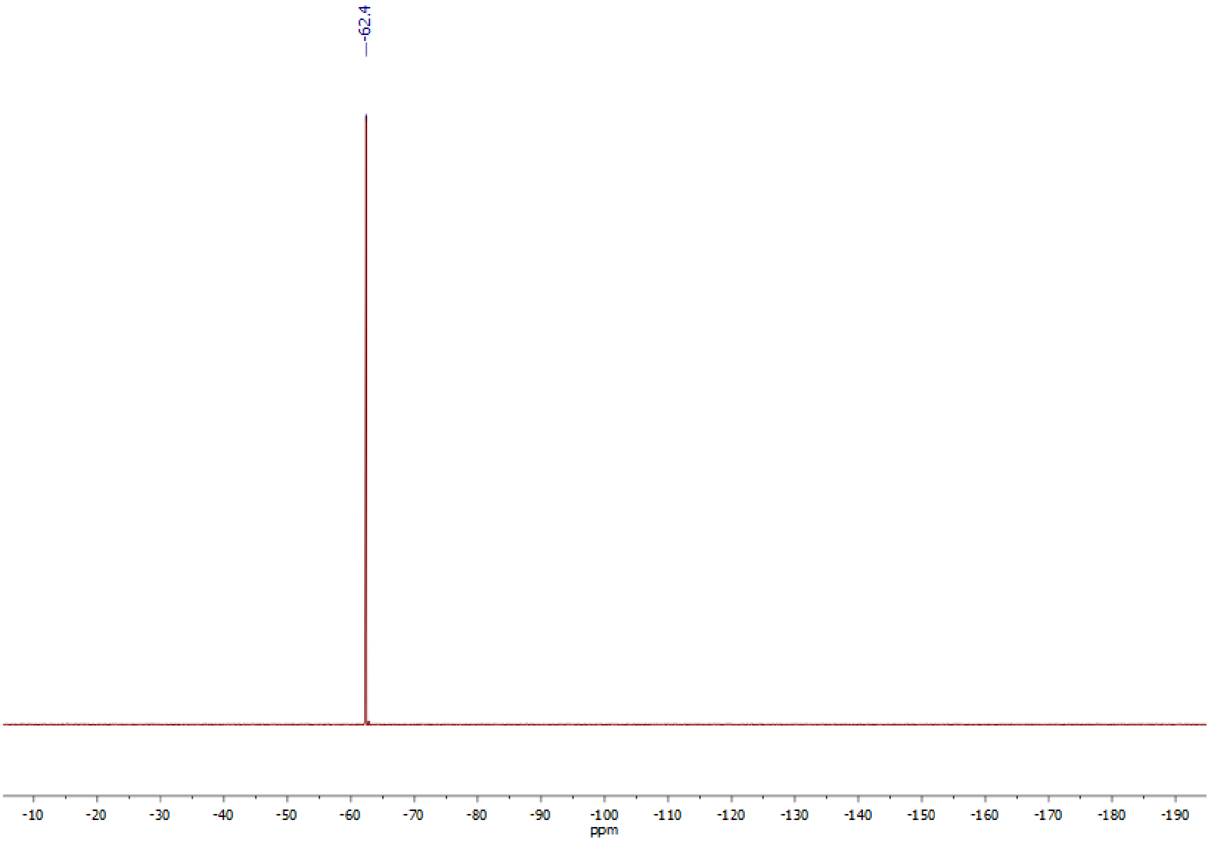

1-Iodo-2-(3-cyanophenyl)pyrene (4n)

^{1}H-NMR (400 MHz, $CDCl_3$)

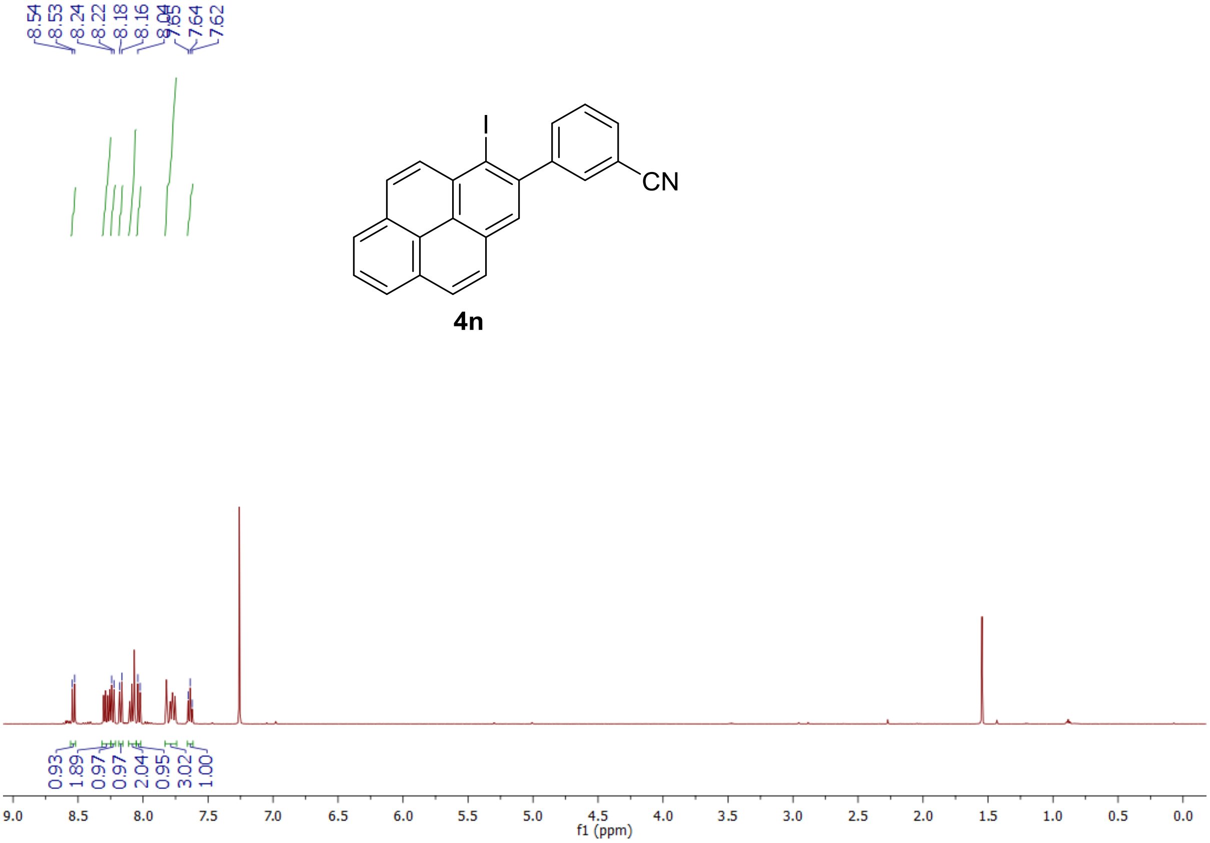

^{13}C-NMR (100 MHz, $CDCl_3$)

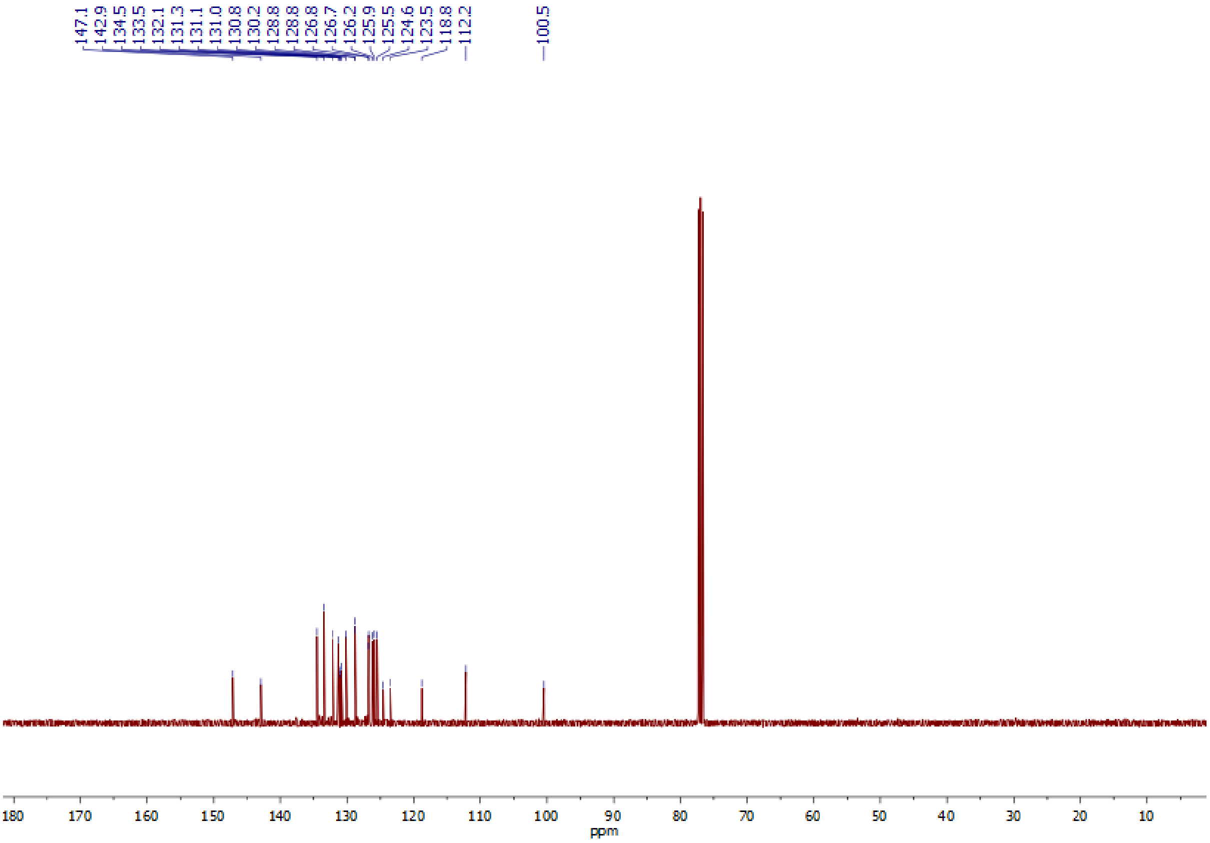

1-Iodo-2-(3-(methoxycarbonyl)phenyl)pyrene (4o)

^{1}H-NMR (400 MHz, $CDCl_3$)

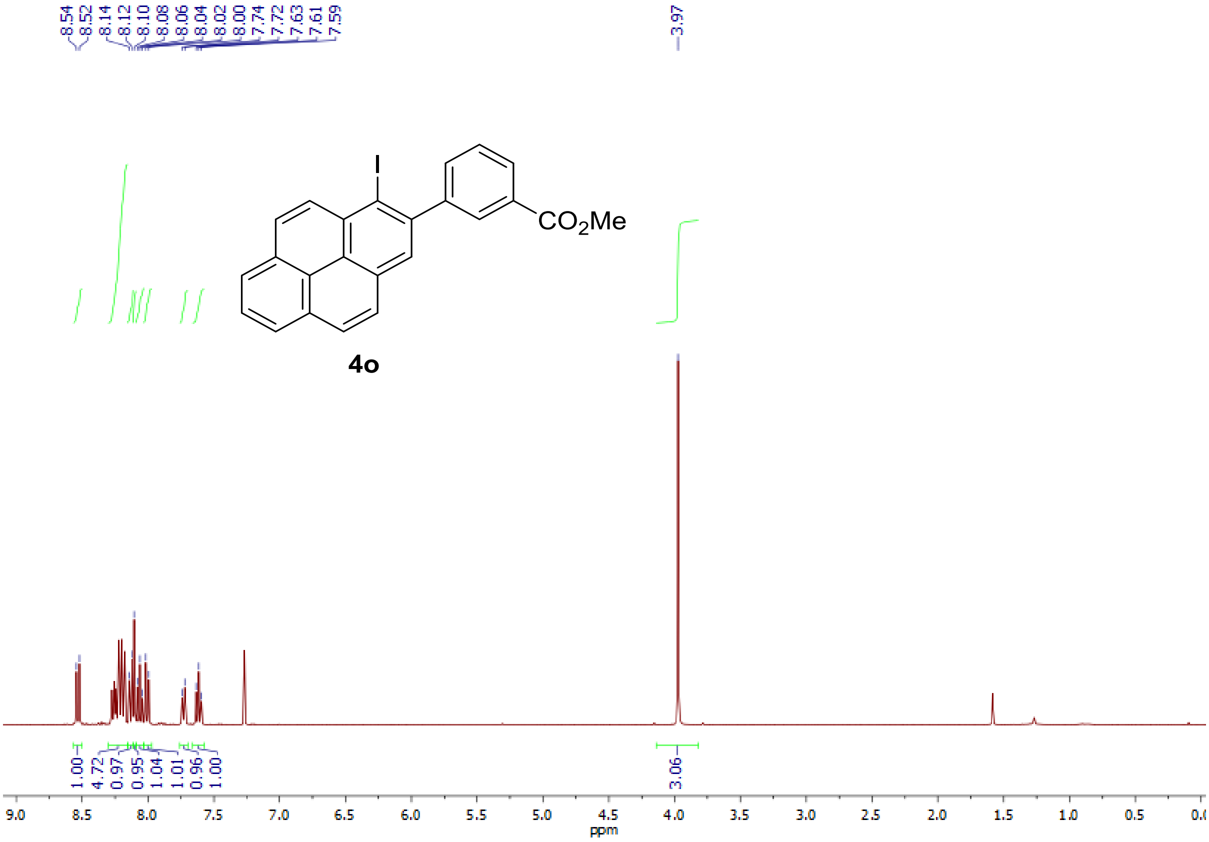

^{13}C-NMR (125 MHz, $CDCl_3$)

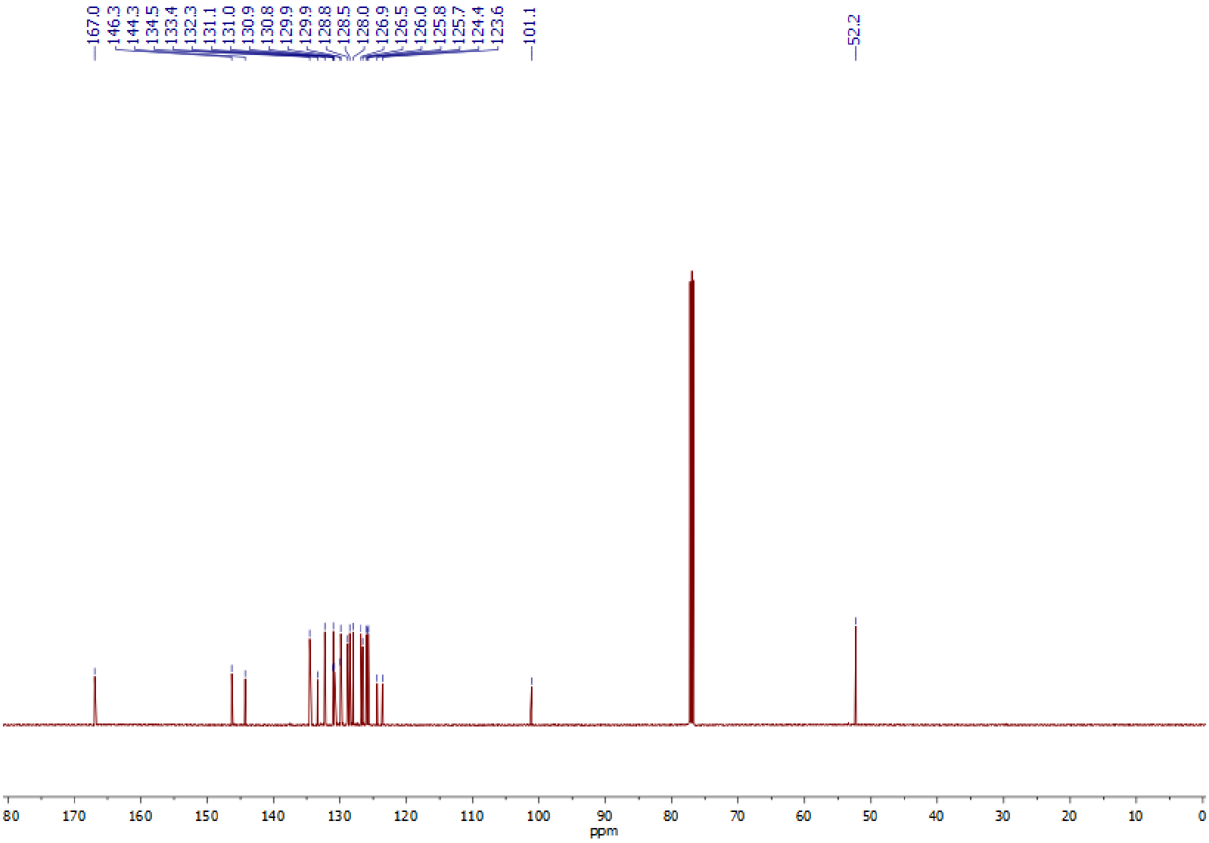

1-Iodo-2-(4-(methoxycarbonyl)phenyl)pyrene (4p)

^{1}H-NMR (500 MHz, $CDCl_3$)

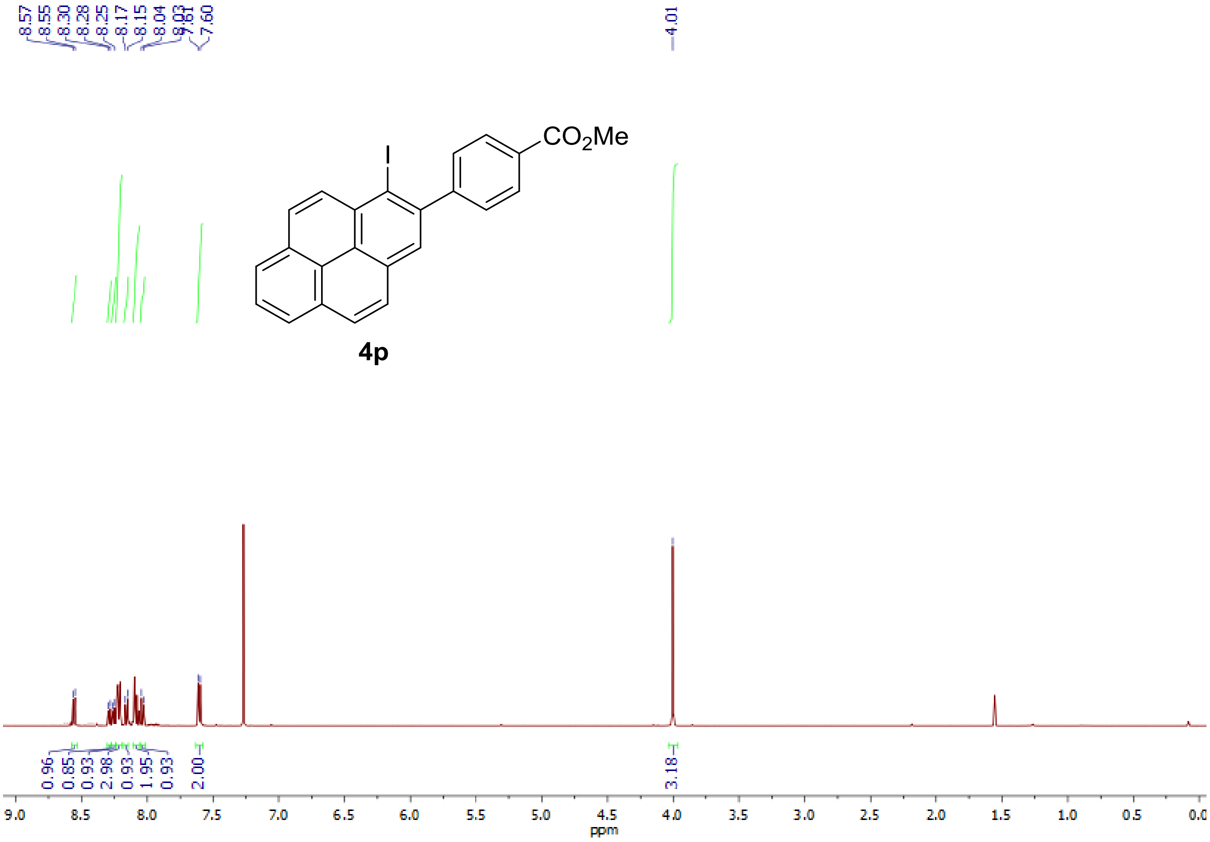

^{13}C-NMR (125 MHz, $CDCl_3$)

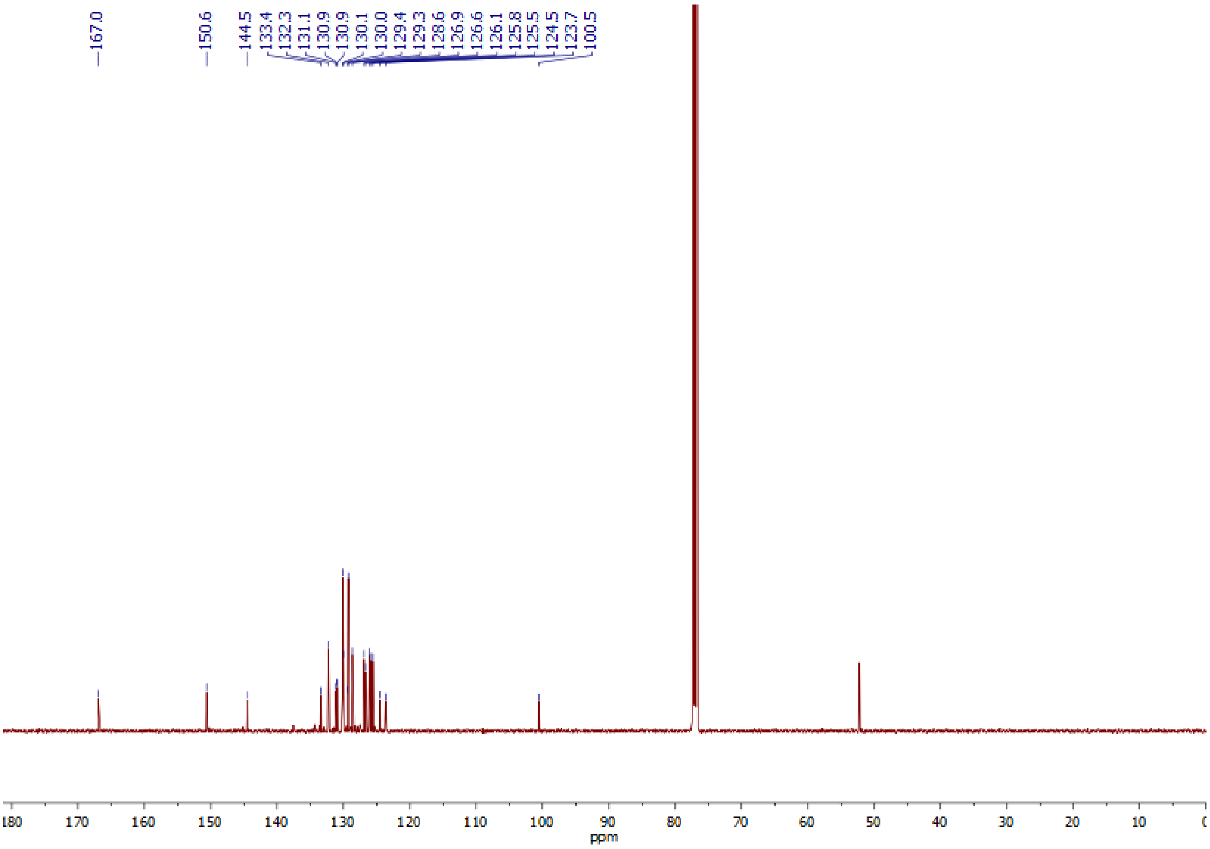

(3,5-Dimethylphenyl)pyrene (5a)

^{1}H-NMR (400 MHz, $CDCl_3$)

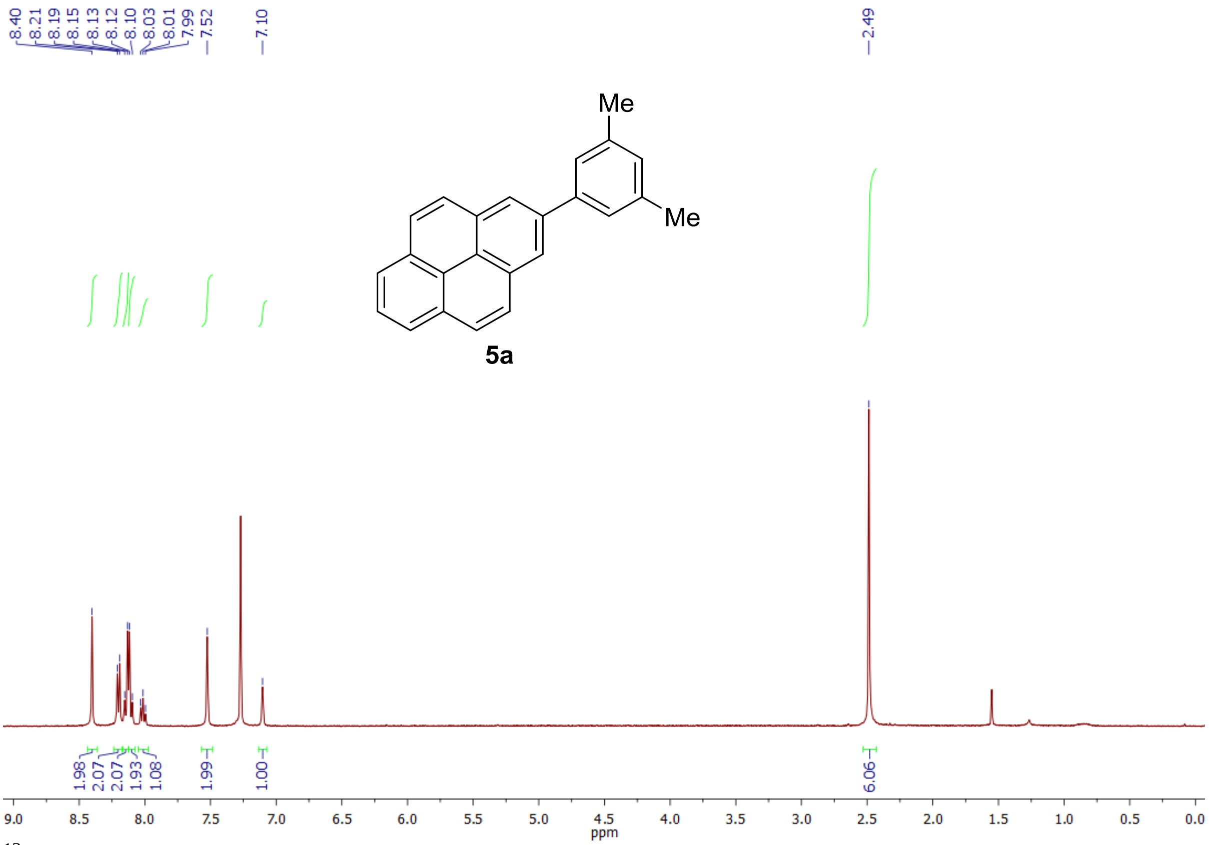

^{13}C-NMR (125 MHz, $CDCl_3$)

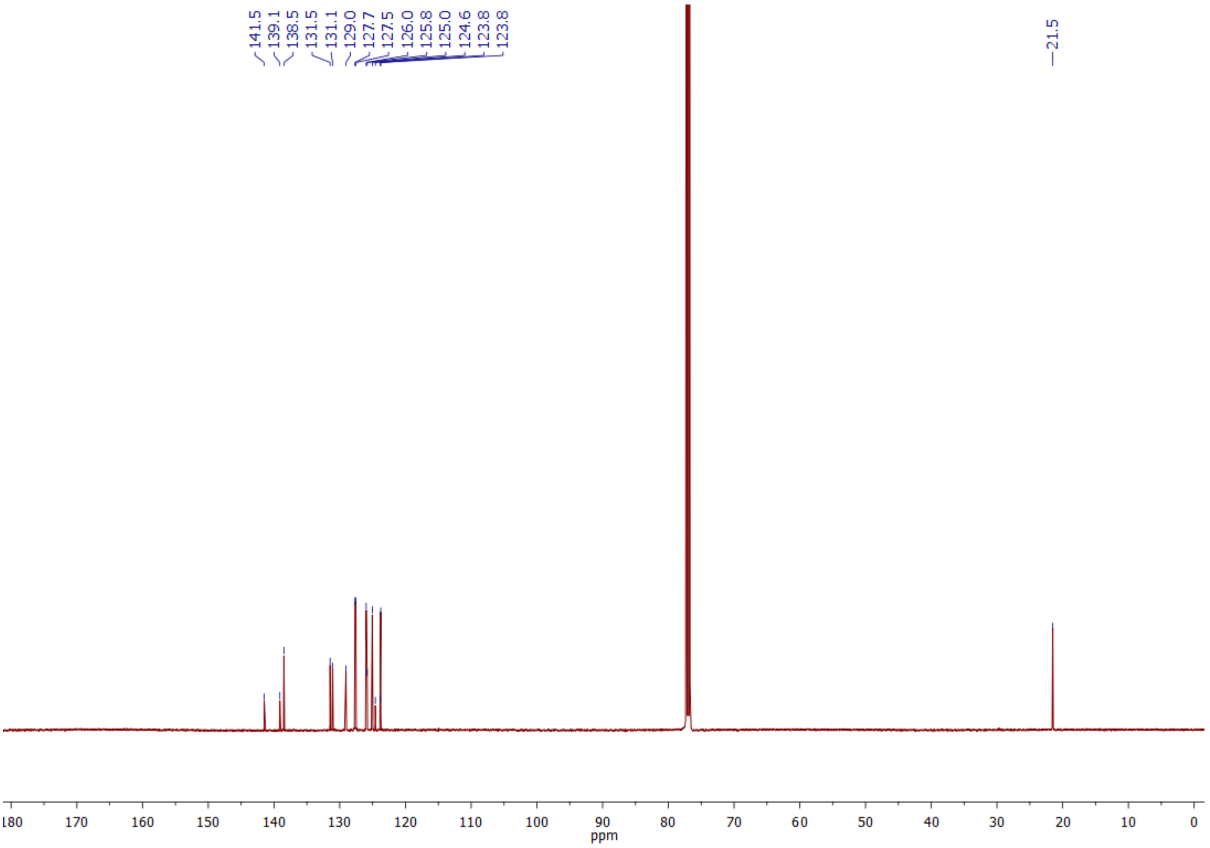

2-(4-Methylphenyl)pyrene (5b)

^{1}H-NMR (400 MHz, $CDCl_3$)

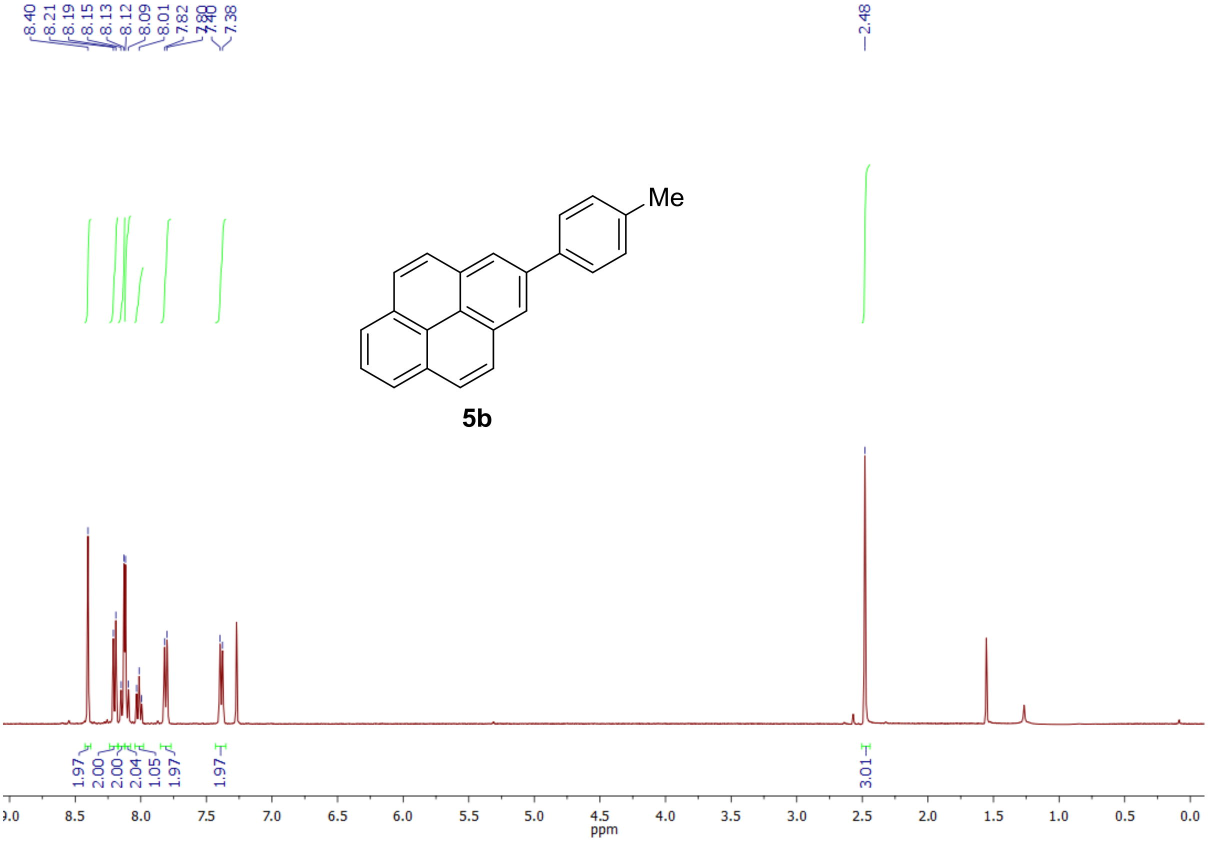

^{13}C-NMR (100 MHz, $CDCl_3$)

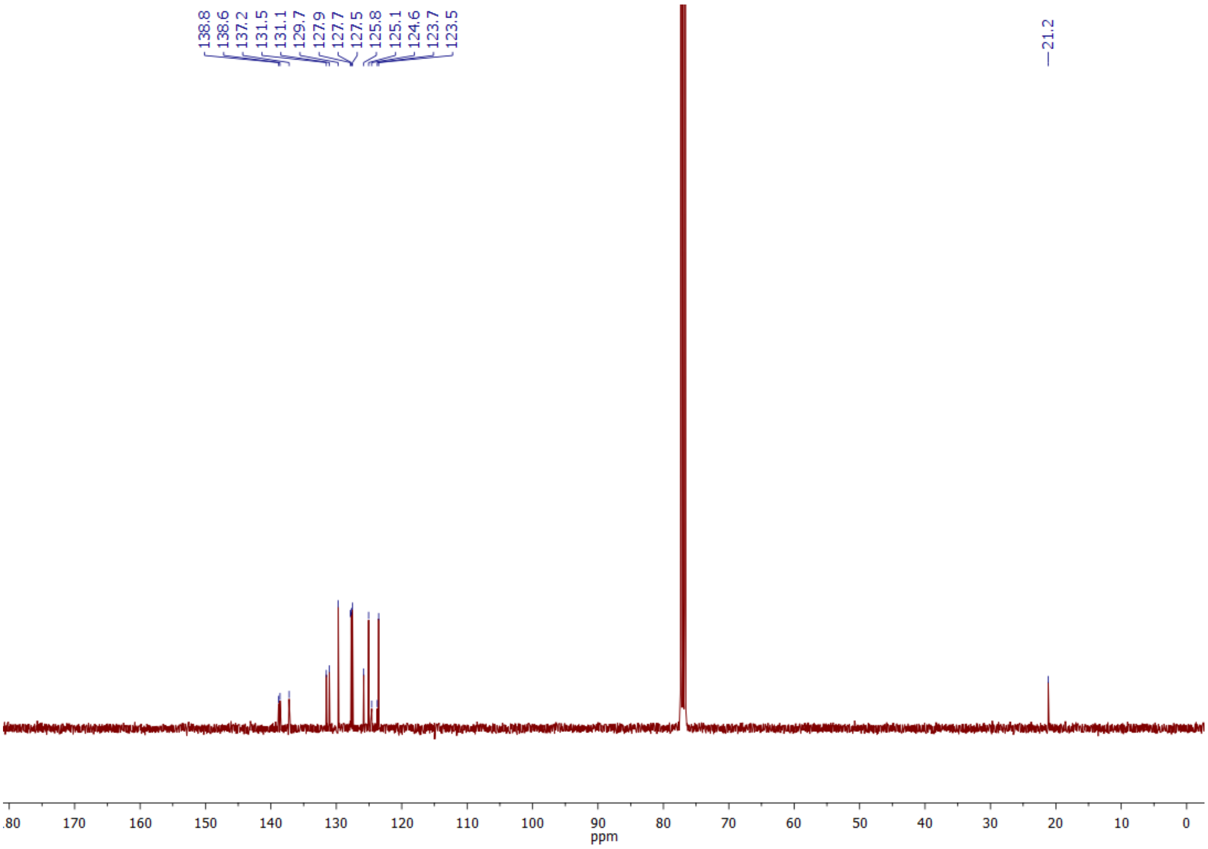

2-(3-Methylphenyl)pyrene (5c)

^{1}H-NMR (400 MHz, $CDCl_3$)

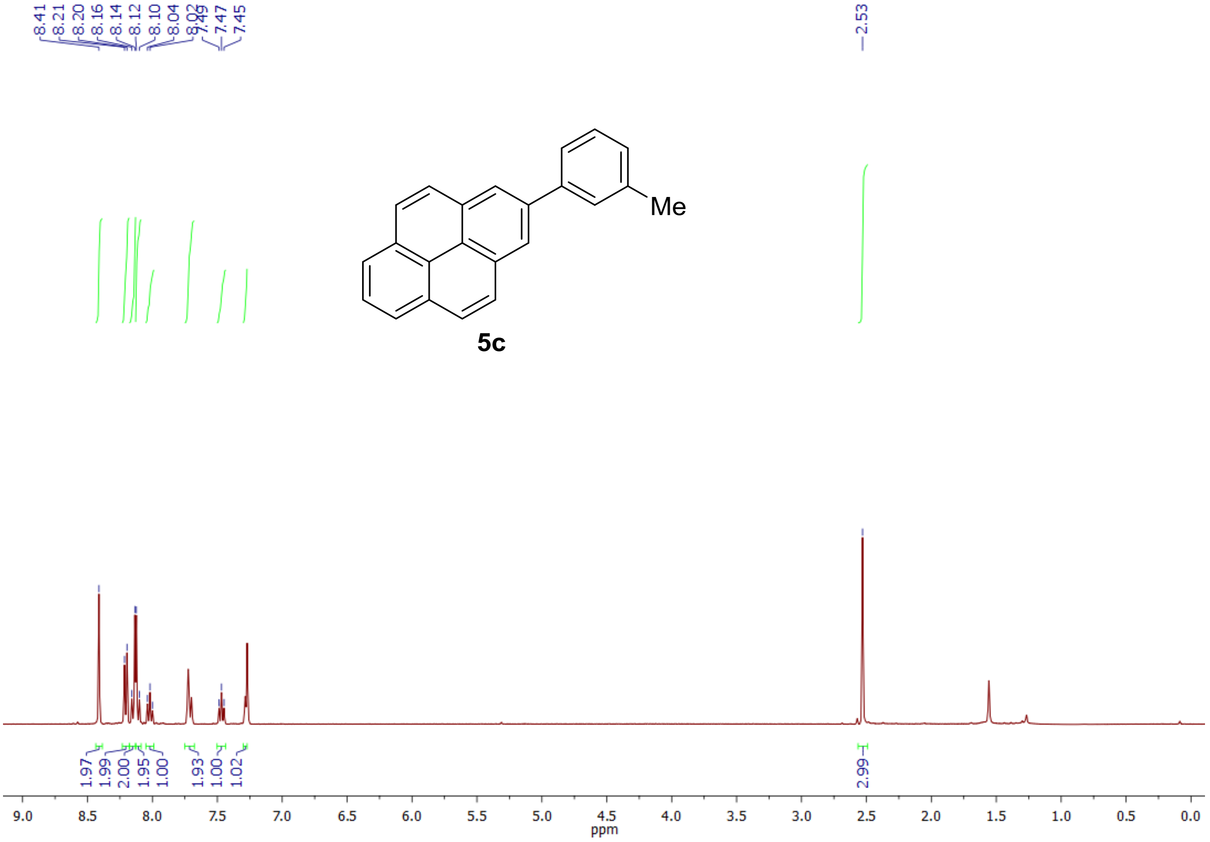

^{13}C-NMR (100 MHz, $CDCl_3$)

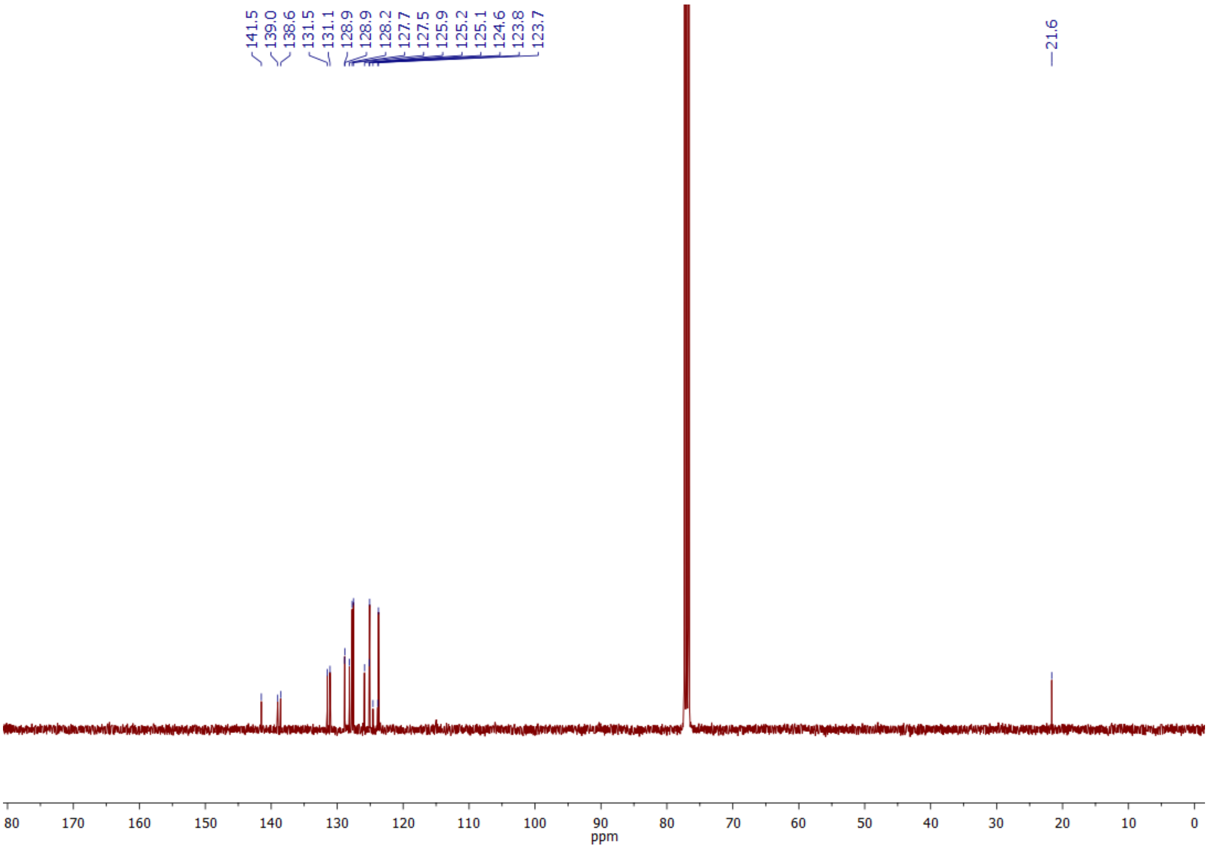

2-(4-*tert*-Butylphenyl)pyrene (5d)

^{1}H-NMR (400 MHz, $CDCl_3$)

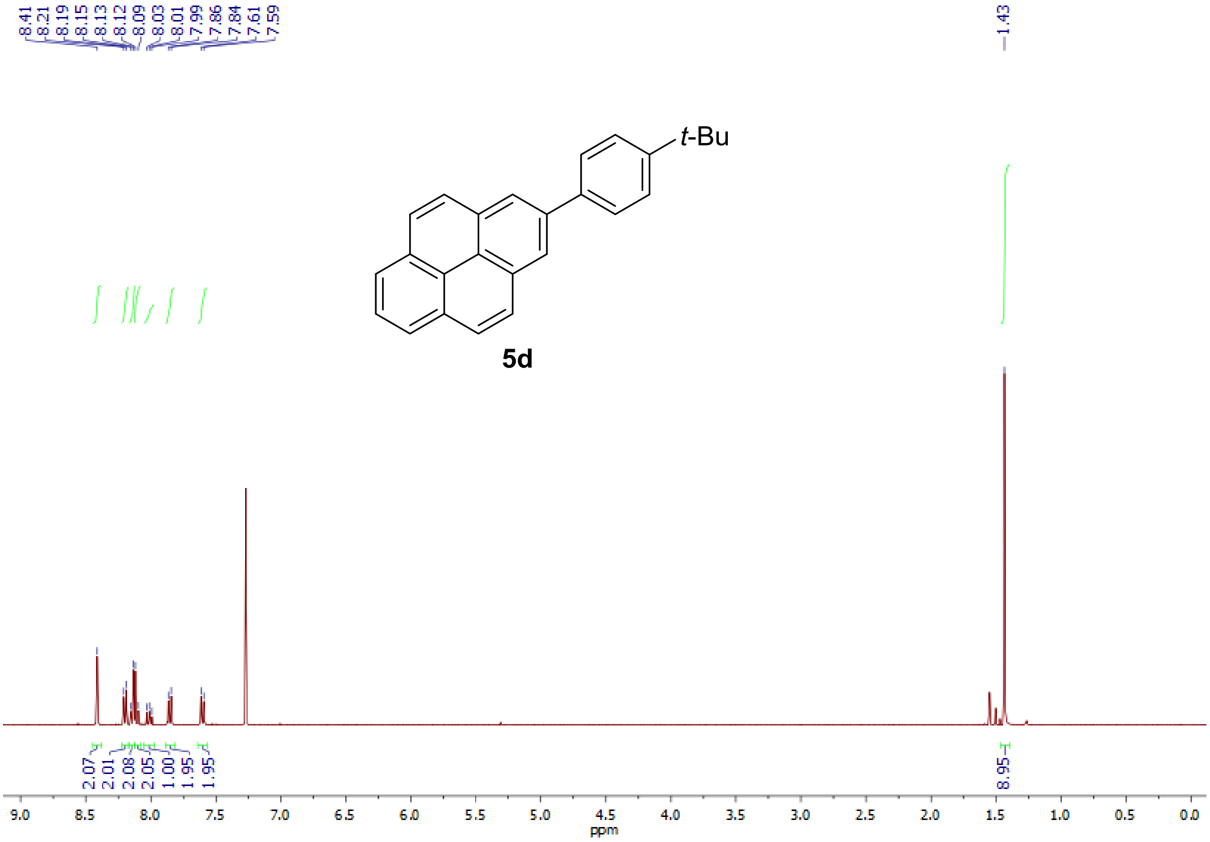

^{13}C-NMR (125 MHz, $CDCl_3$)

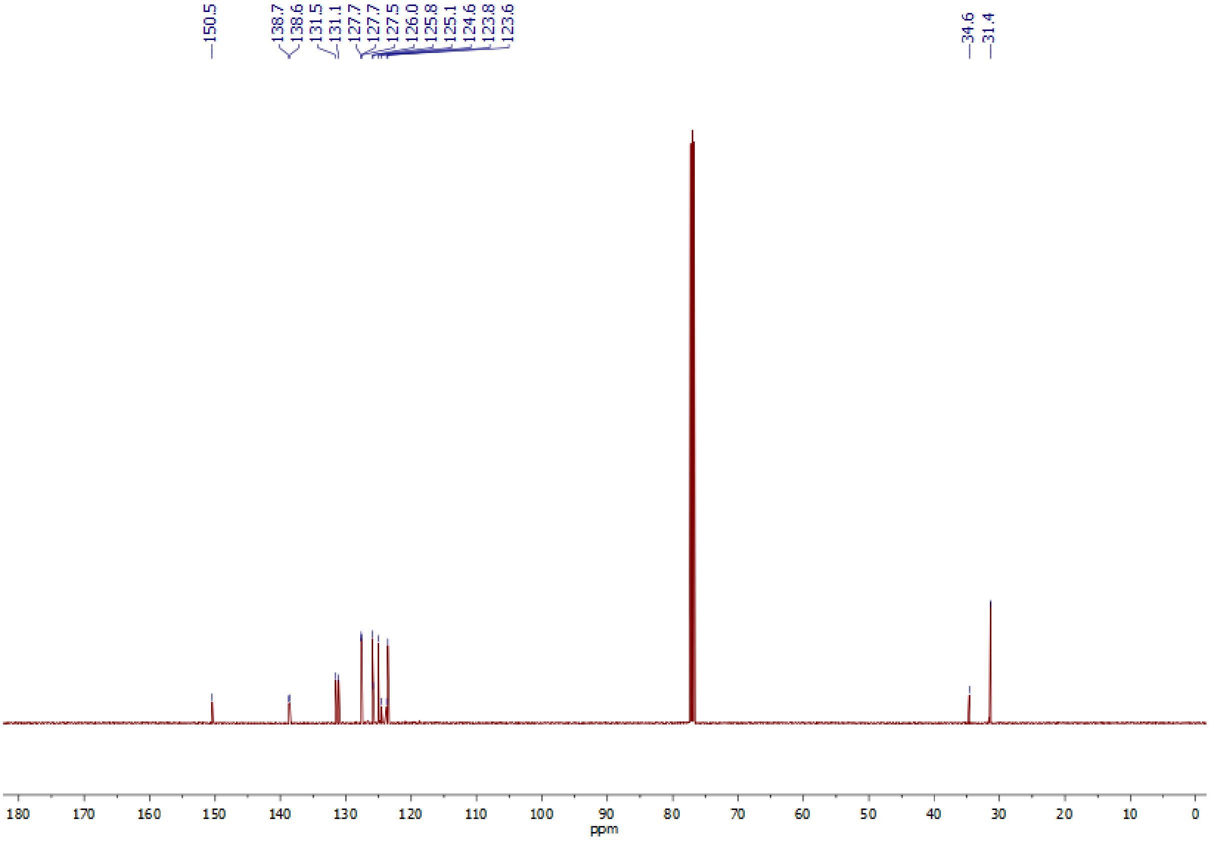

2-(4-methoxyphenyl)pyrene (5e)

^{1}H-NMR (400 MHz, $CDCl_3$)

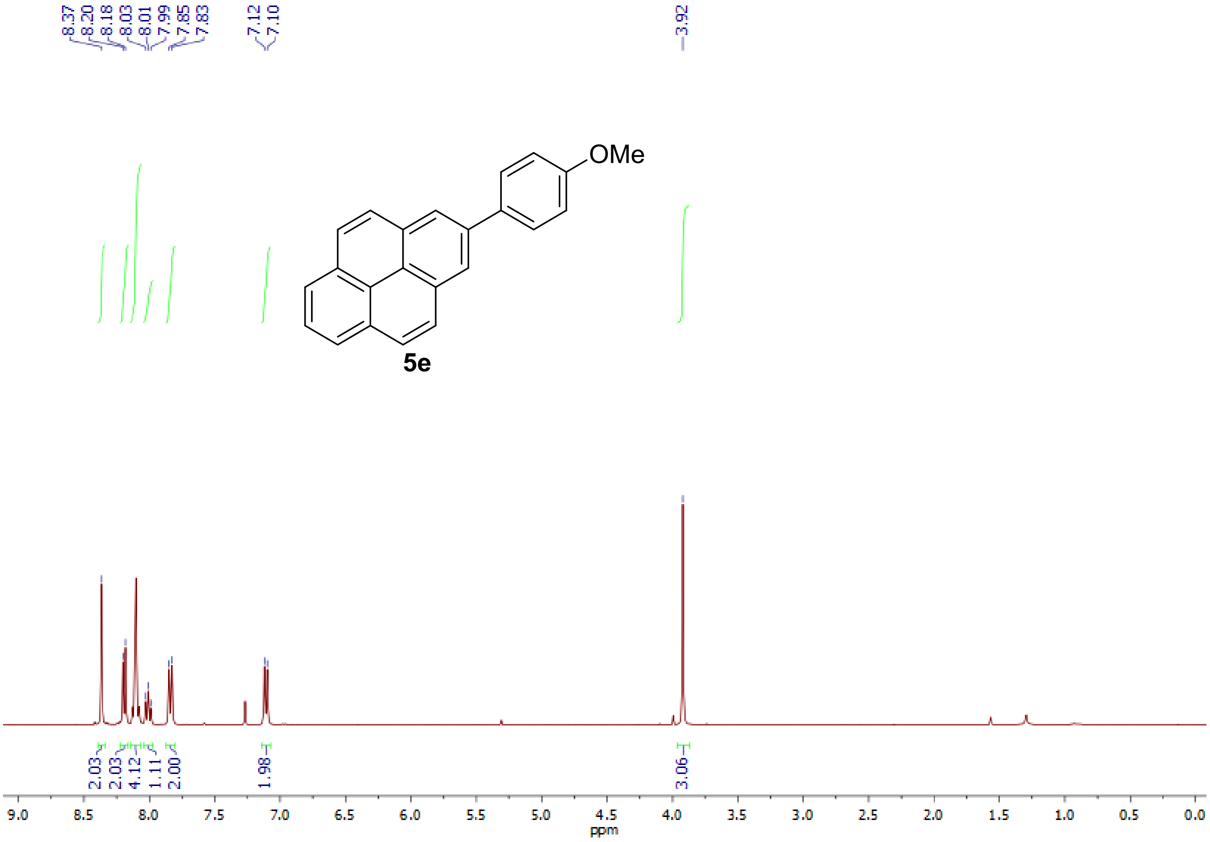

^{13}C-NMR (125 MHz, $CDCl_3$)

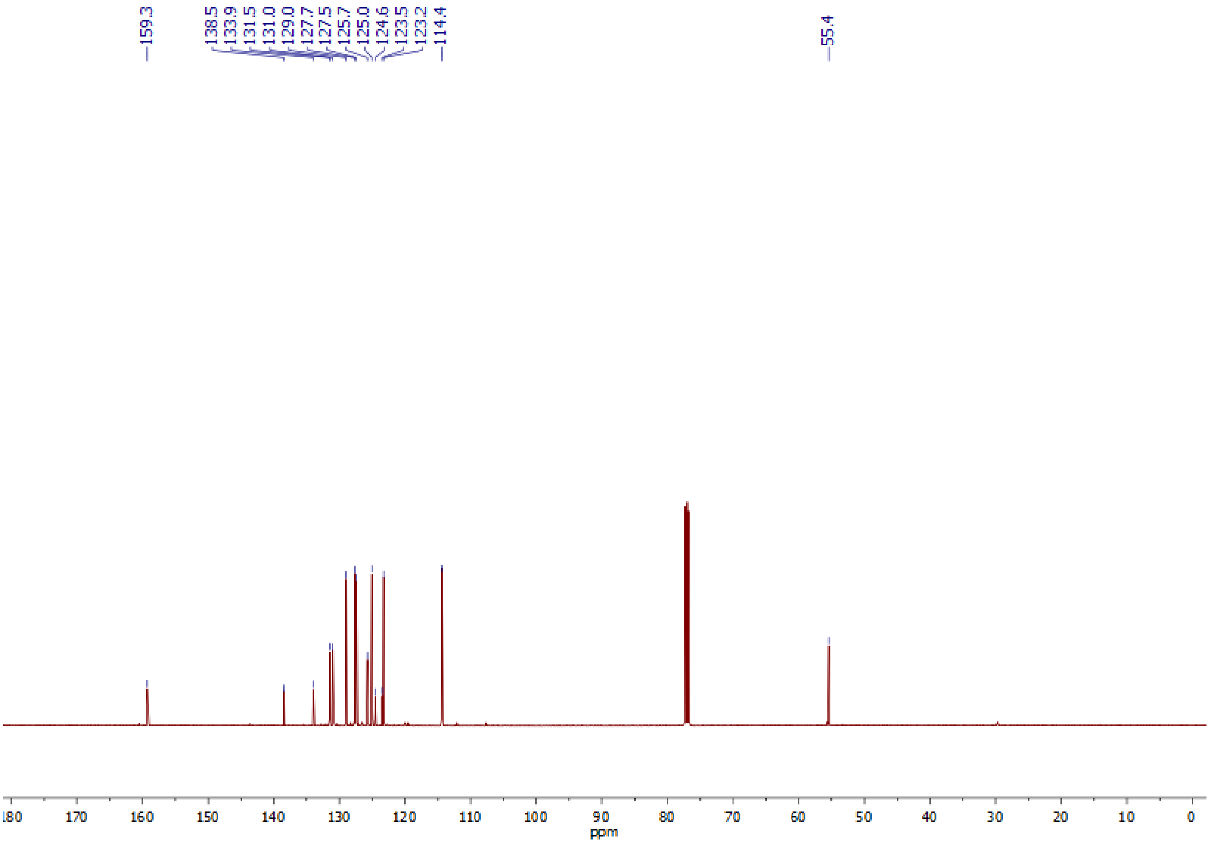

2-Phenylpyrene (5f)

^{1}H-NMR (400 MHz, $CDCl_3$)

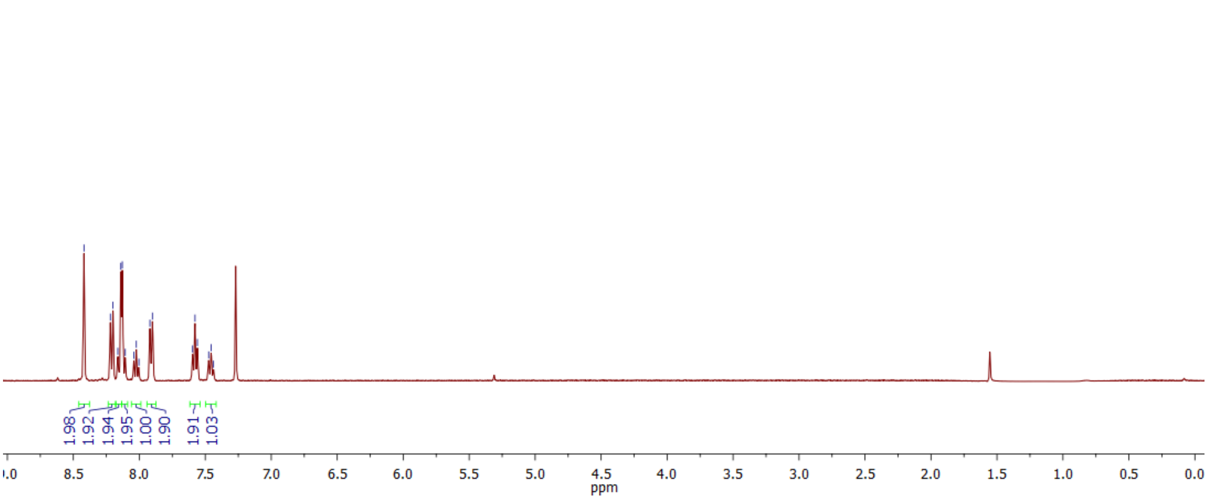

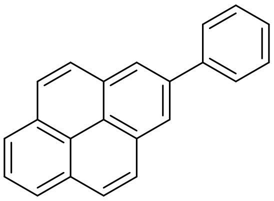

5f

^{13}C-NMR (100 MHz, $CDCl_3$)

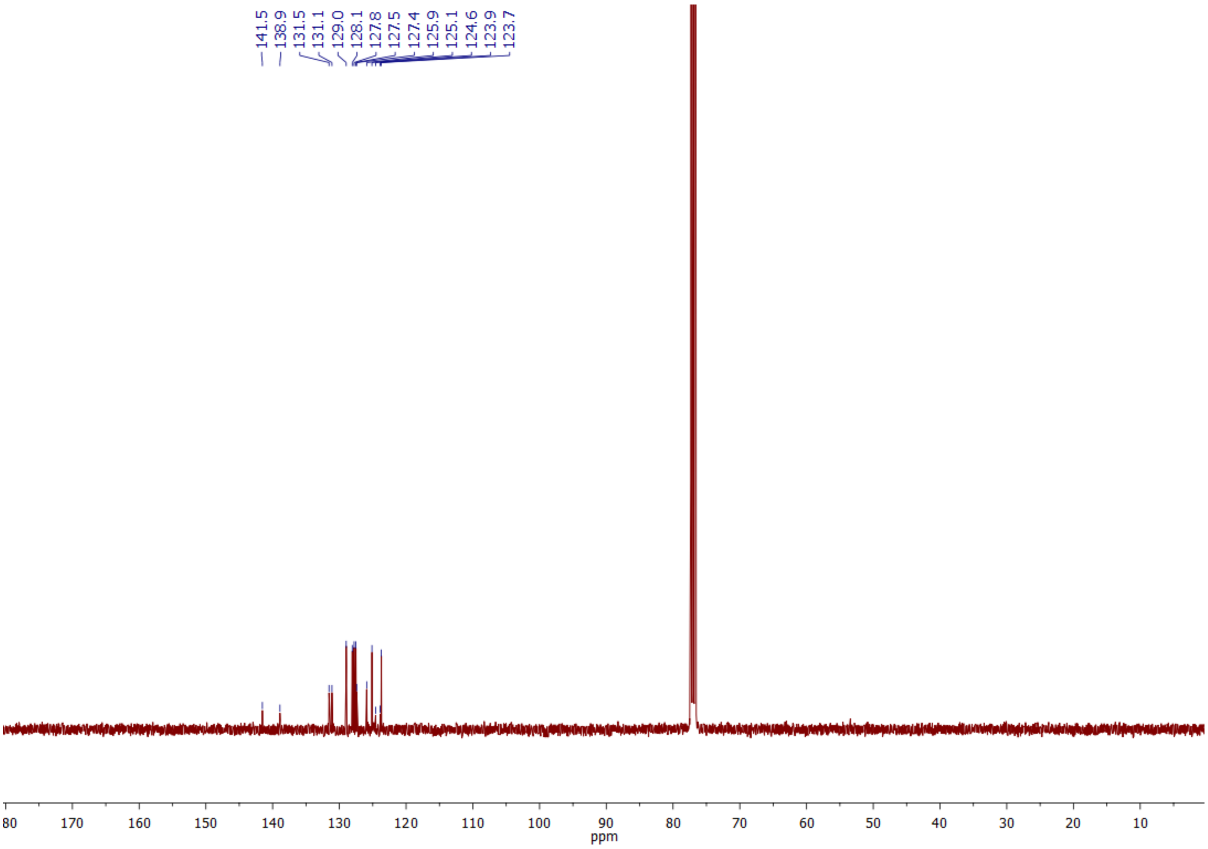

2-(4-Bromophenyl)pyrene (5g)

^{1}H-NMR (400 MHz, $CDCl_3$)

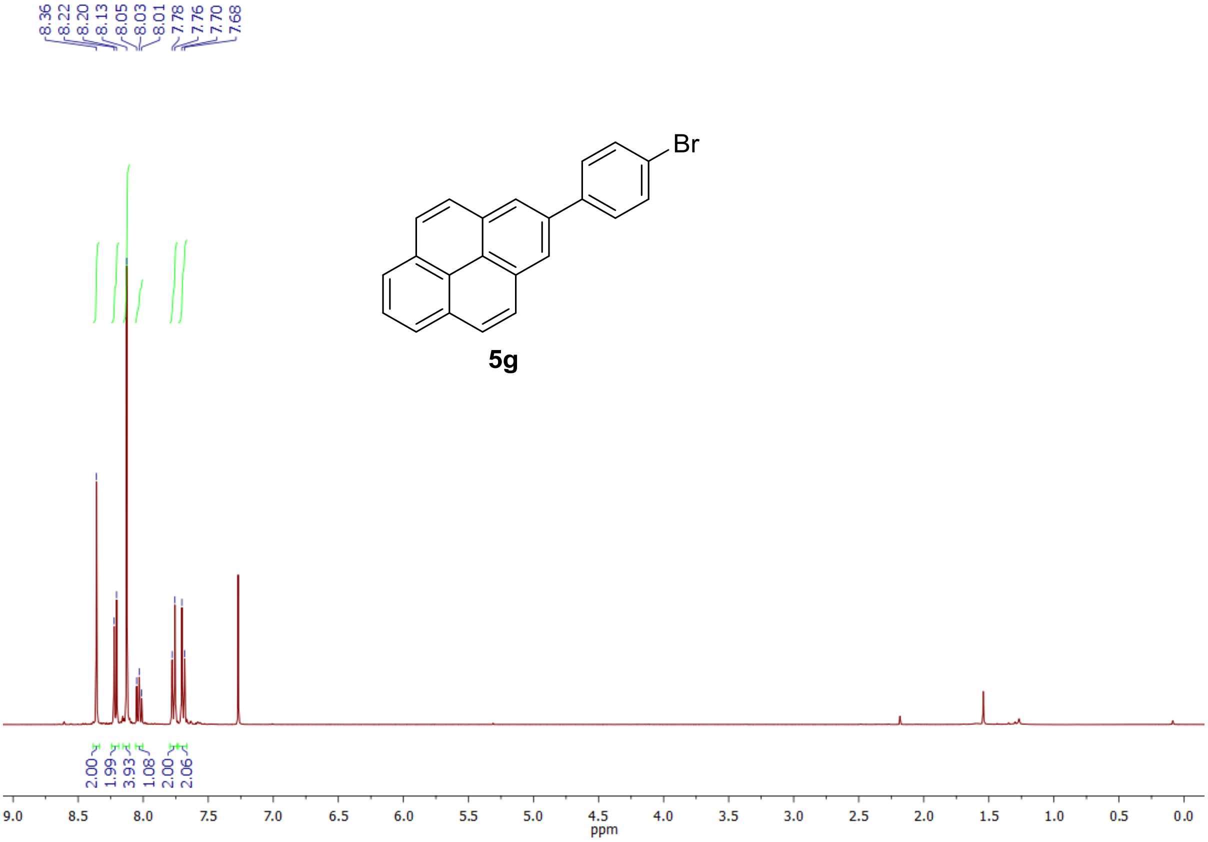

^{13}C-NMR (100 MHz, $CDCl_3$)

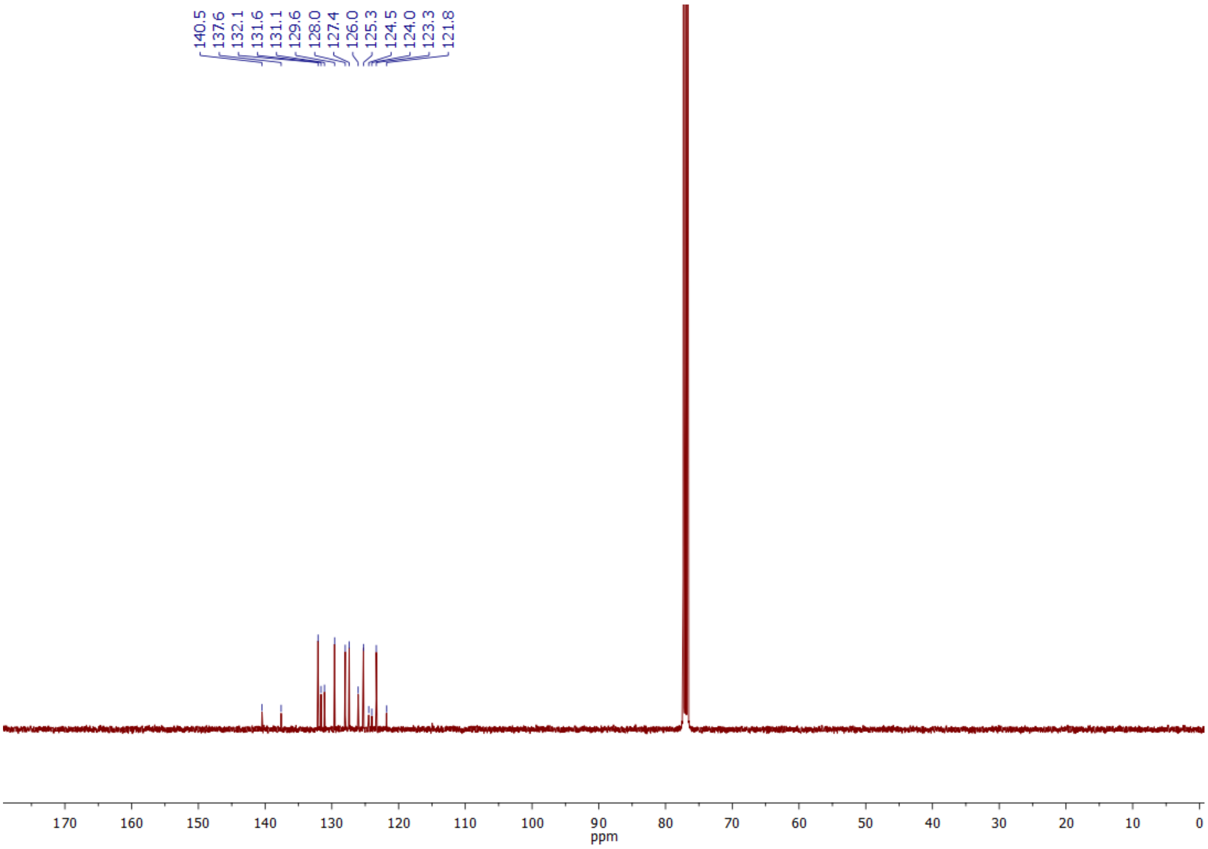

2-(3-Bromophenyl)pyrene (5h)

^{1}H-NMR (500 MHz, $CDCl_3$)

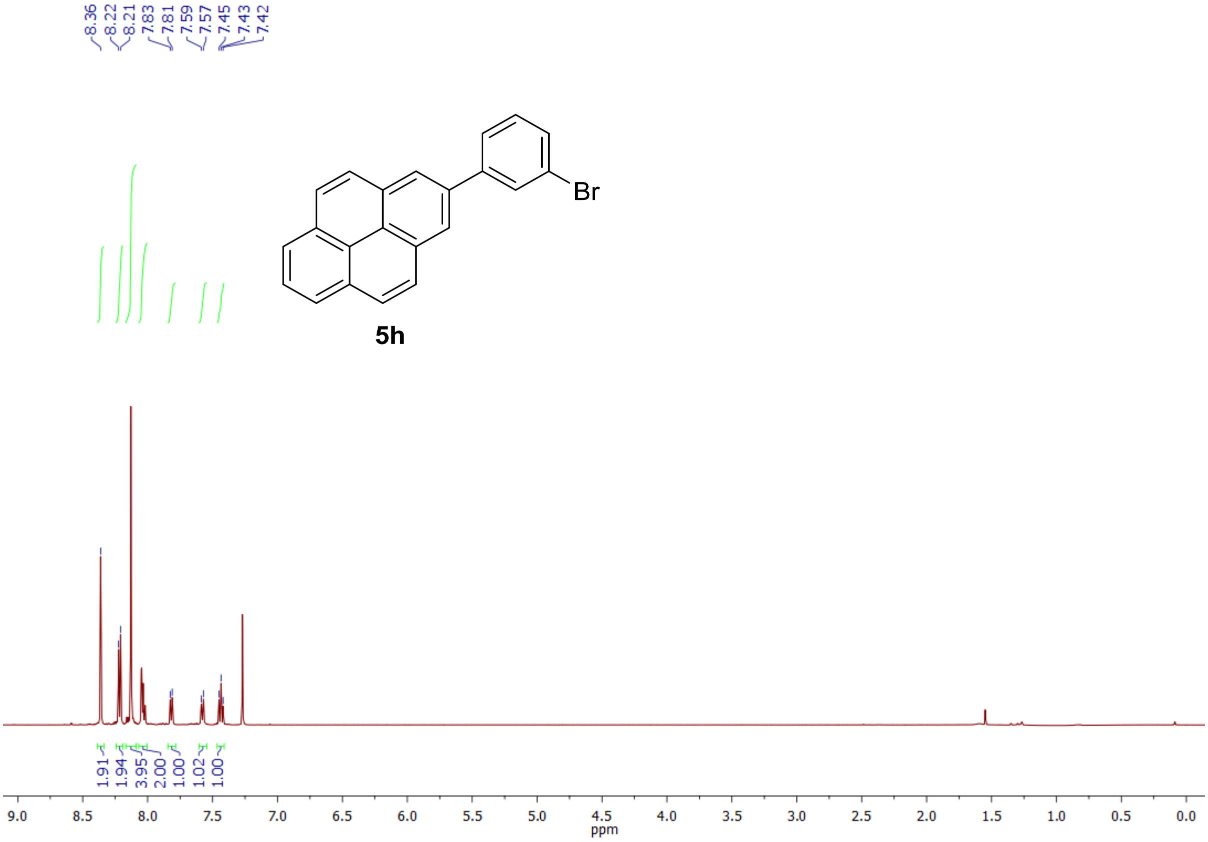

^{13}C-NMR (125 MHz, $CDCl_3$)

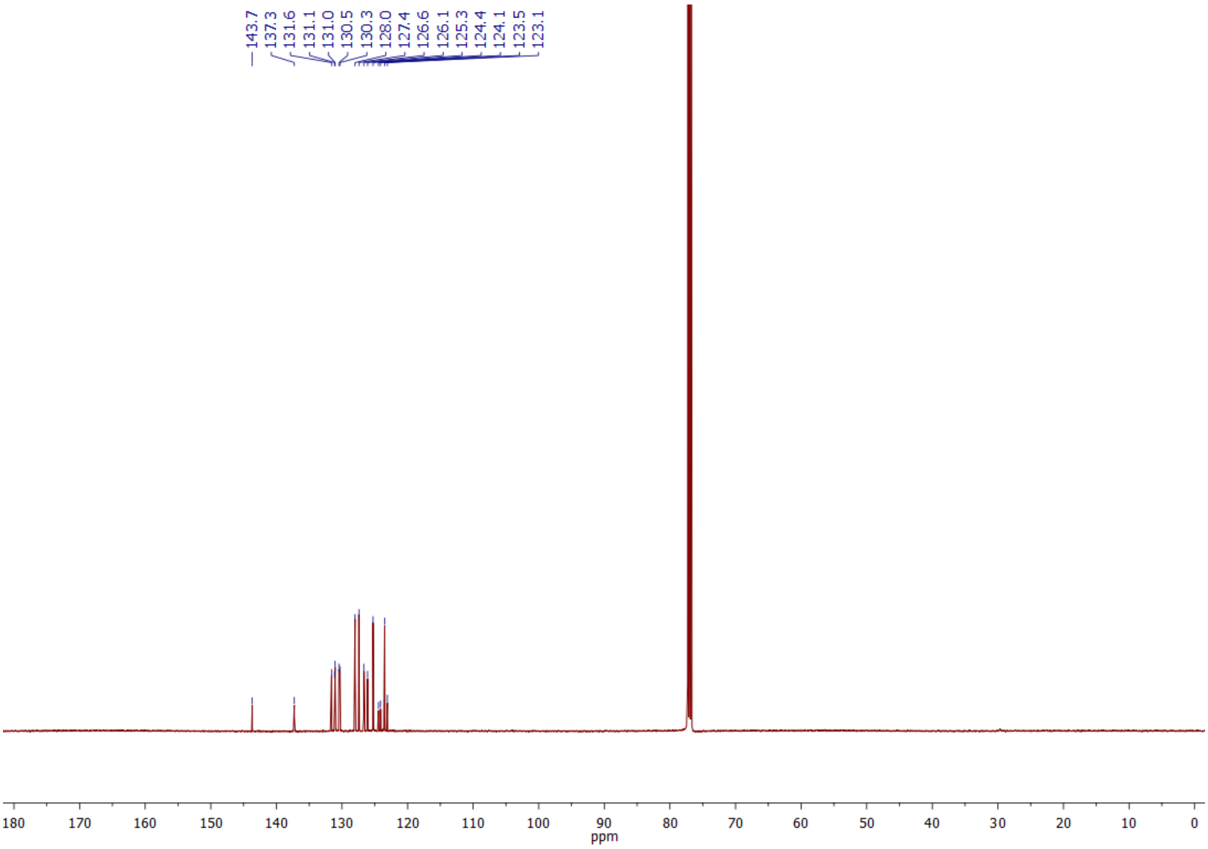

2-(3-Chlorophenyl)pyrene (5i)

^{1}H-NMR (500 MHz, $CDCl_3$)

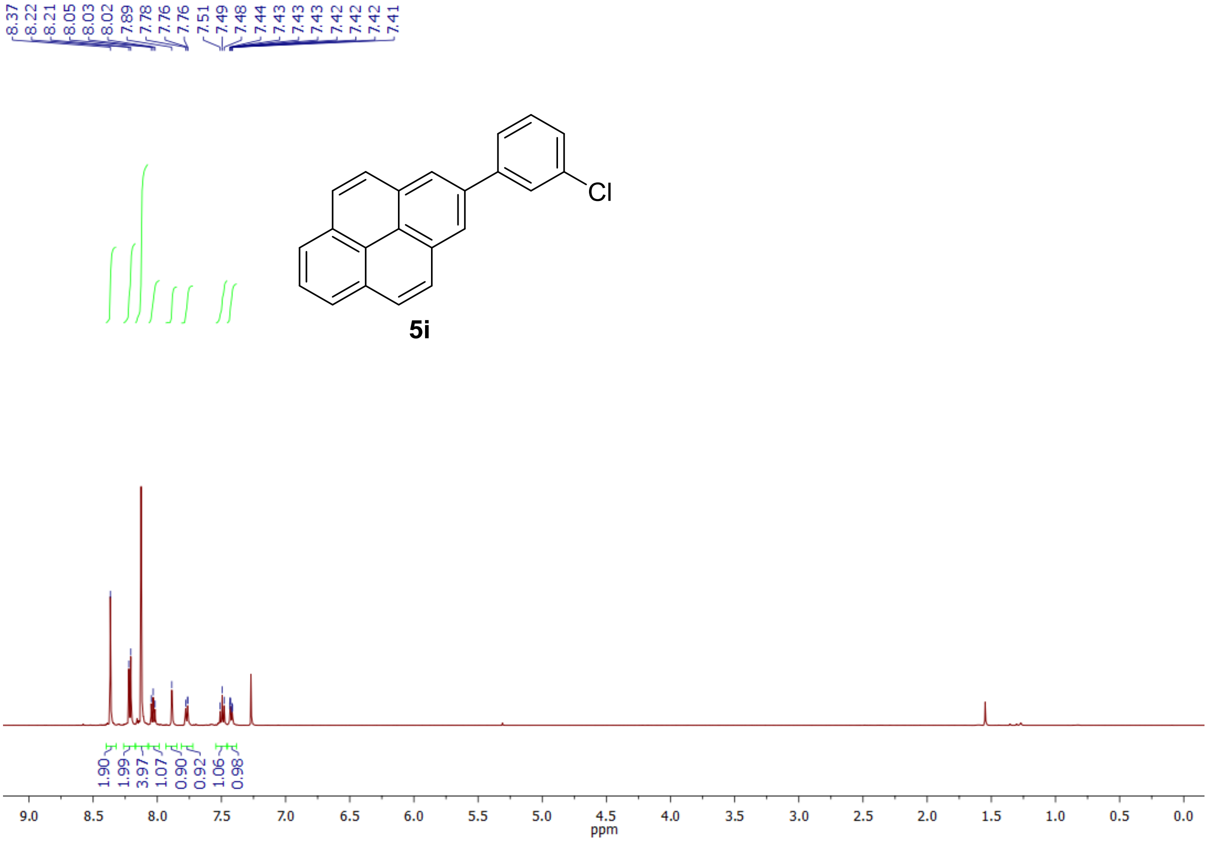

^{13}C-NMR (125 MHz, $CDCl_3$)

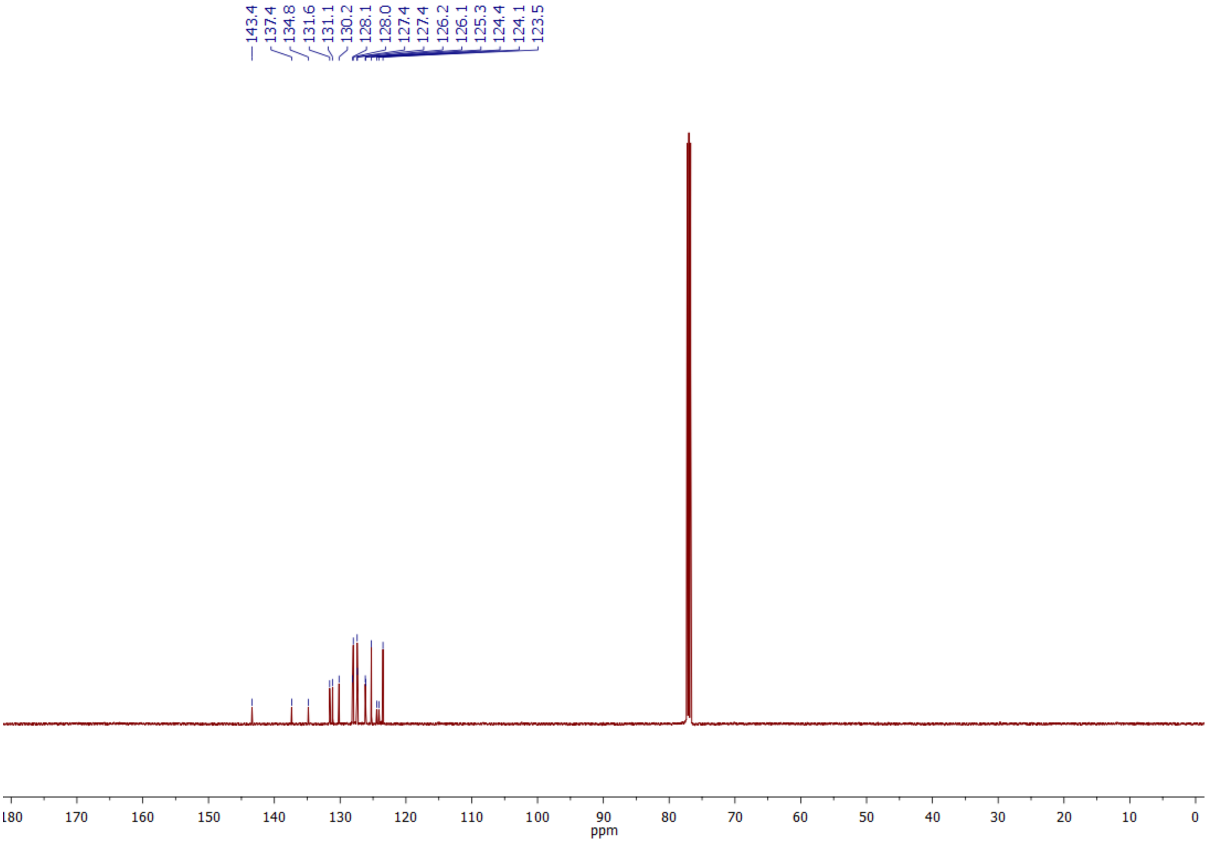

2-(3,5-Dichlorophenyl)pyrene (5j)

^{1}H-NMR (400 MHz, $CDCl_3$)

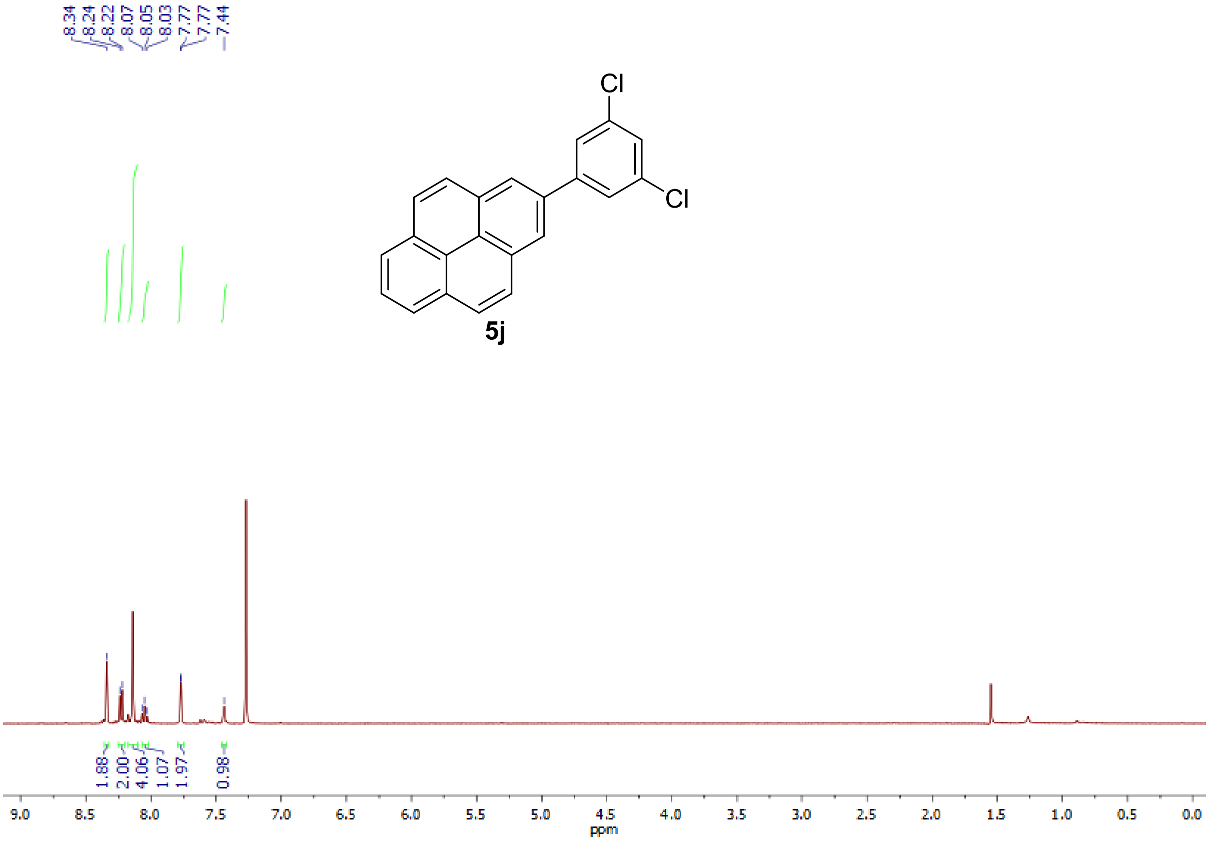

^{13}C-NMR (125 MHz, $CDCl_3$)

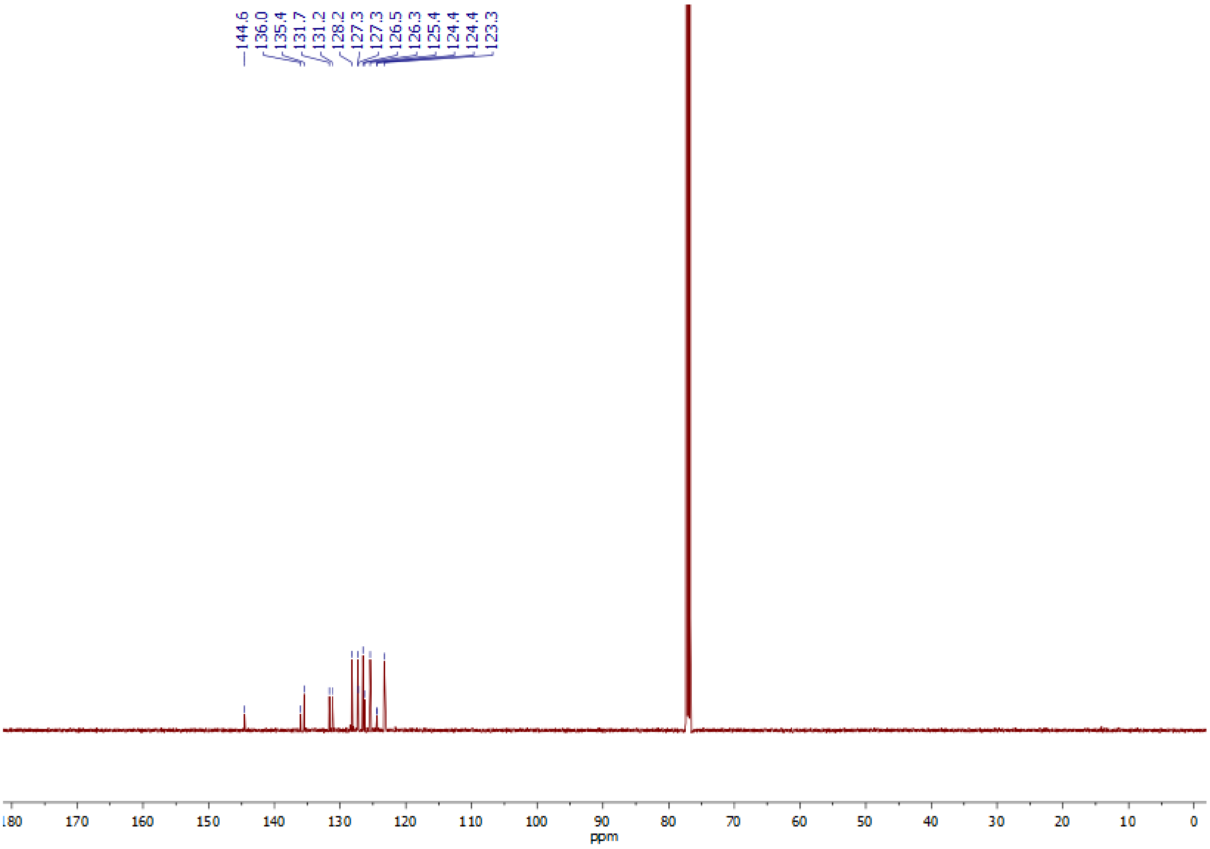

2-(3,5-Bis(trifluoromethyl)phenyl)pyrene (5k)

^{1}H-NMR (400 MHz, $CDCl_3$)

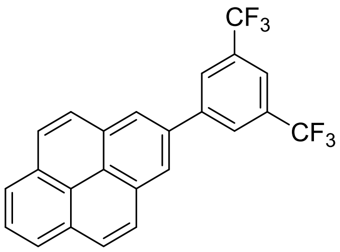

5k

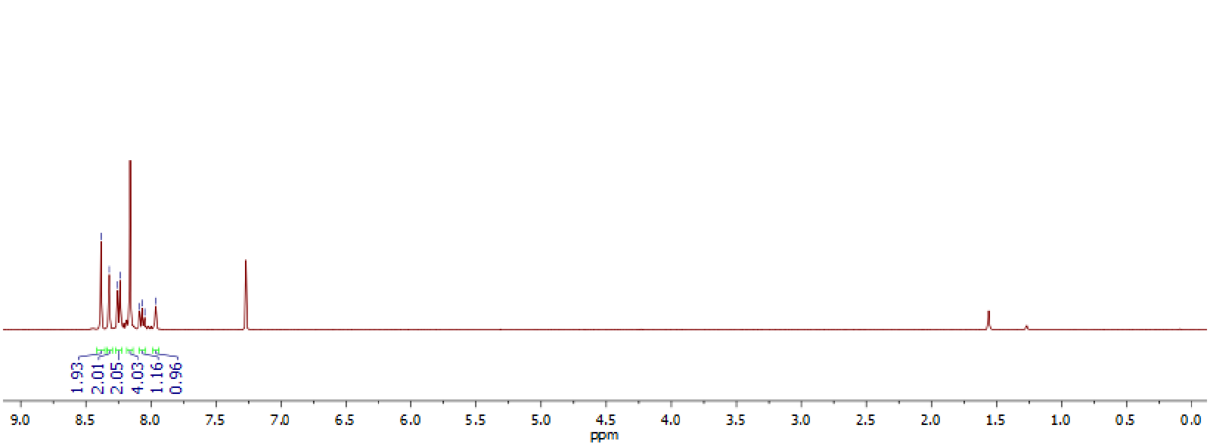

^{13}C-NMR (125 MHz, $CDCl_3$)

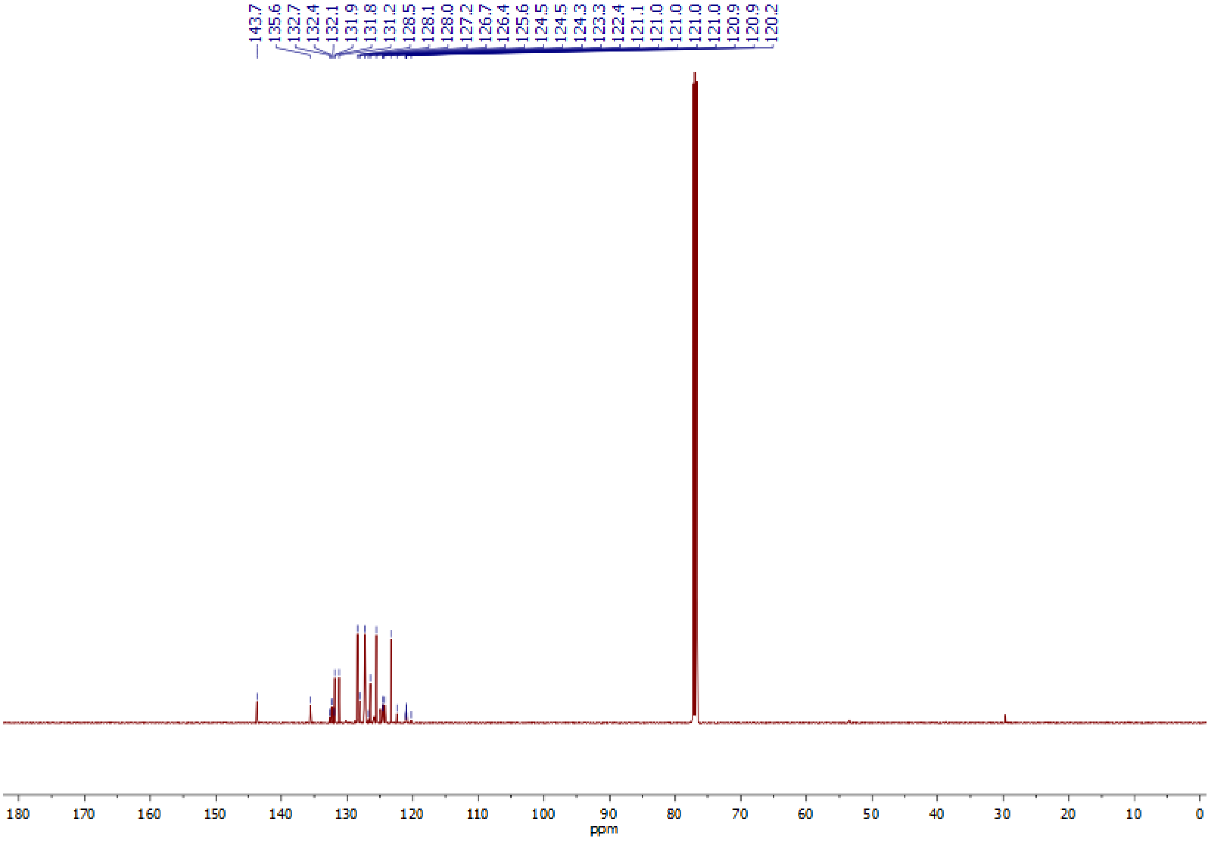

^{19}F-NMR (471 MHz, $CDCl_3$)

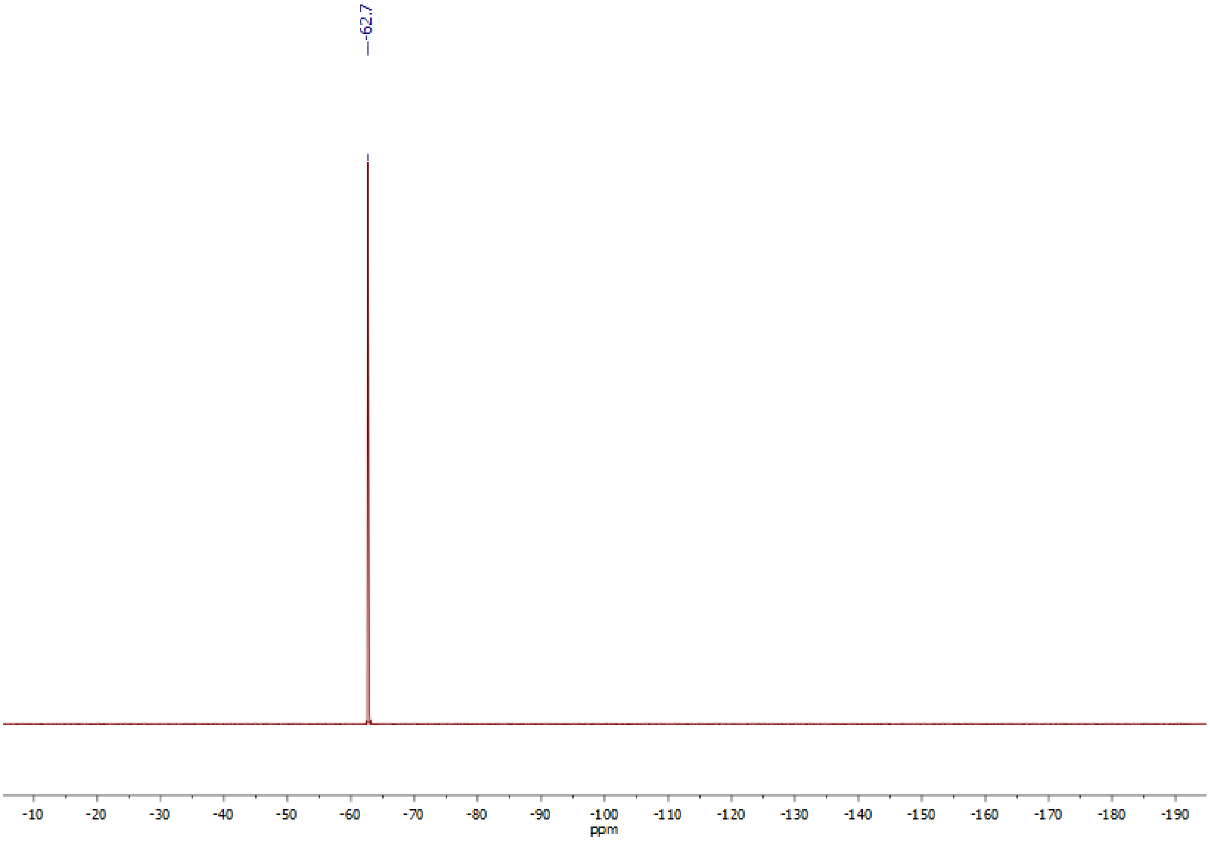

2-(3-nitrophenyl)pyrene (5l)

^{1}H-NMR (500 MHz, $CDCl_3$)

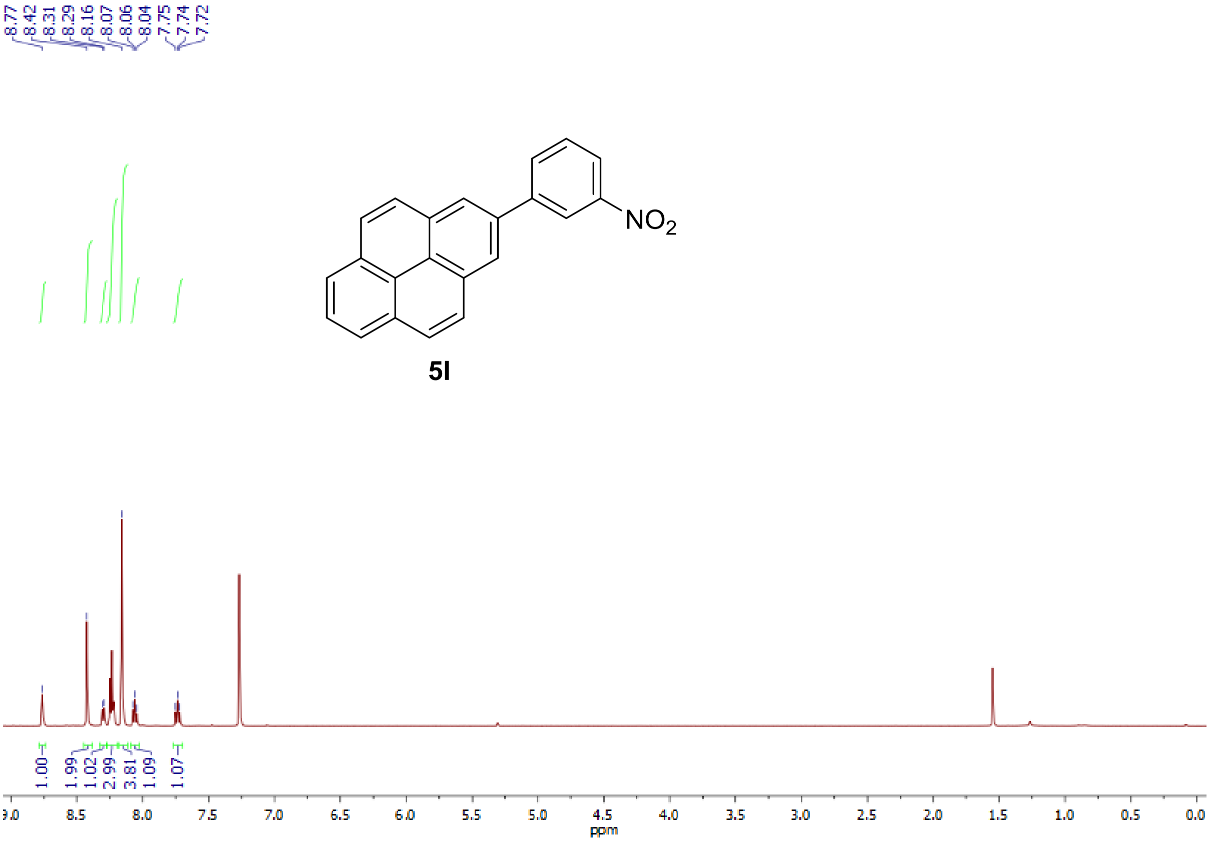

^{13}C-NMR (125 MHz, $CDCl_3$)

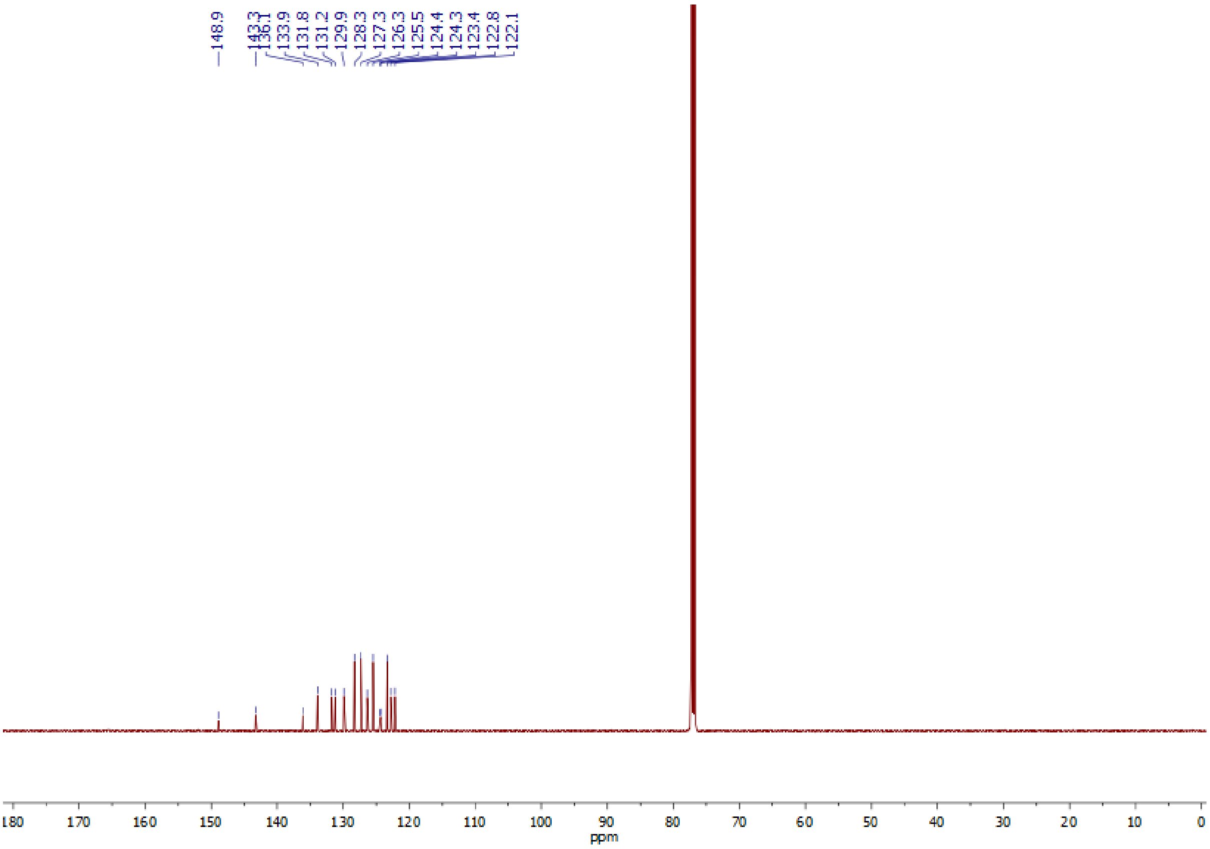

2-(3-trifluoromethylphenyl)pyrene (5m)

^{1}H-NMR (400 MHz, $CDCl_3$)

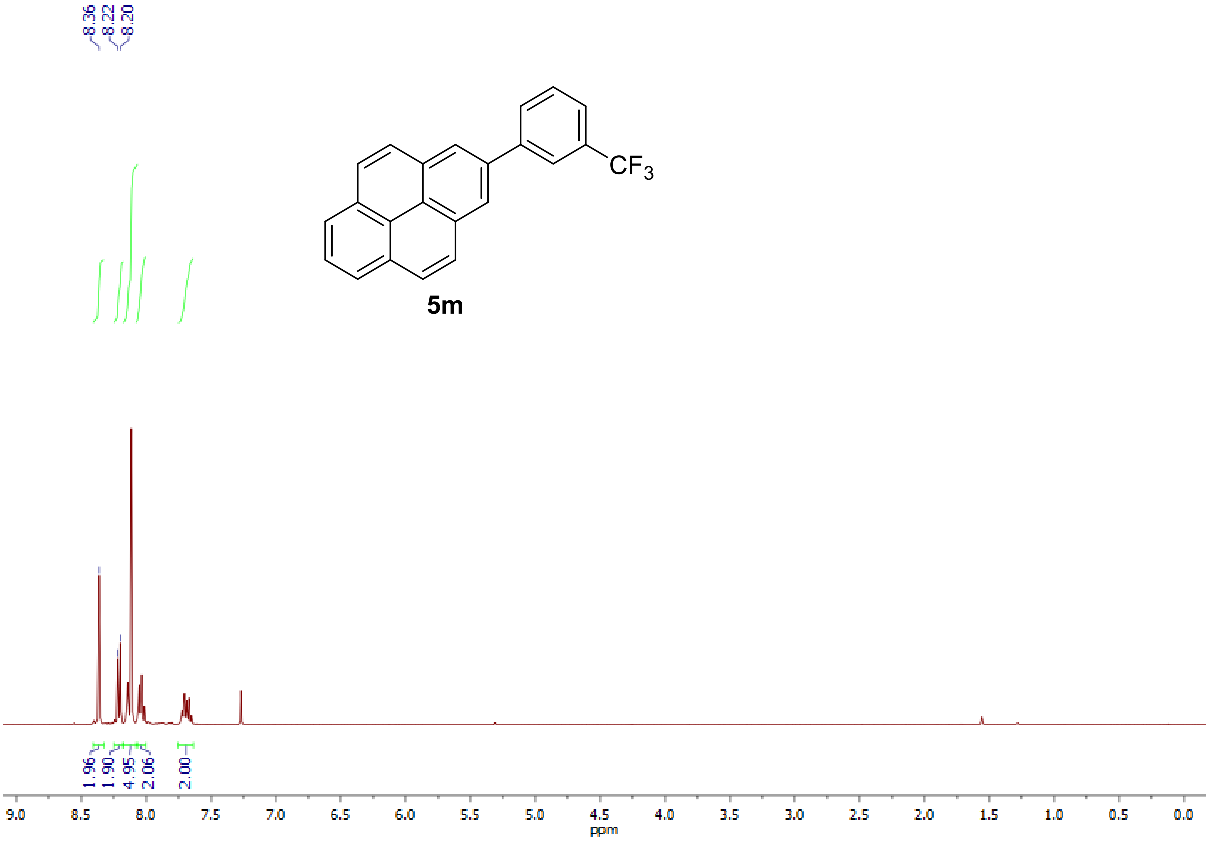

^{13}C-NMR (125 MHz, $CDCl_3$)

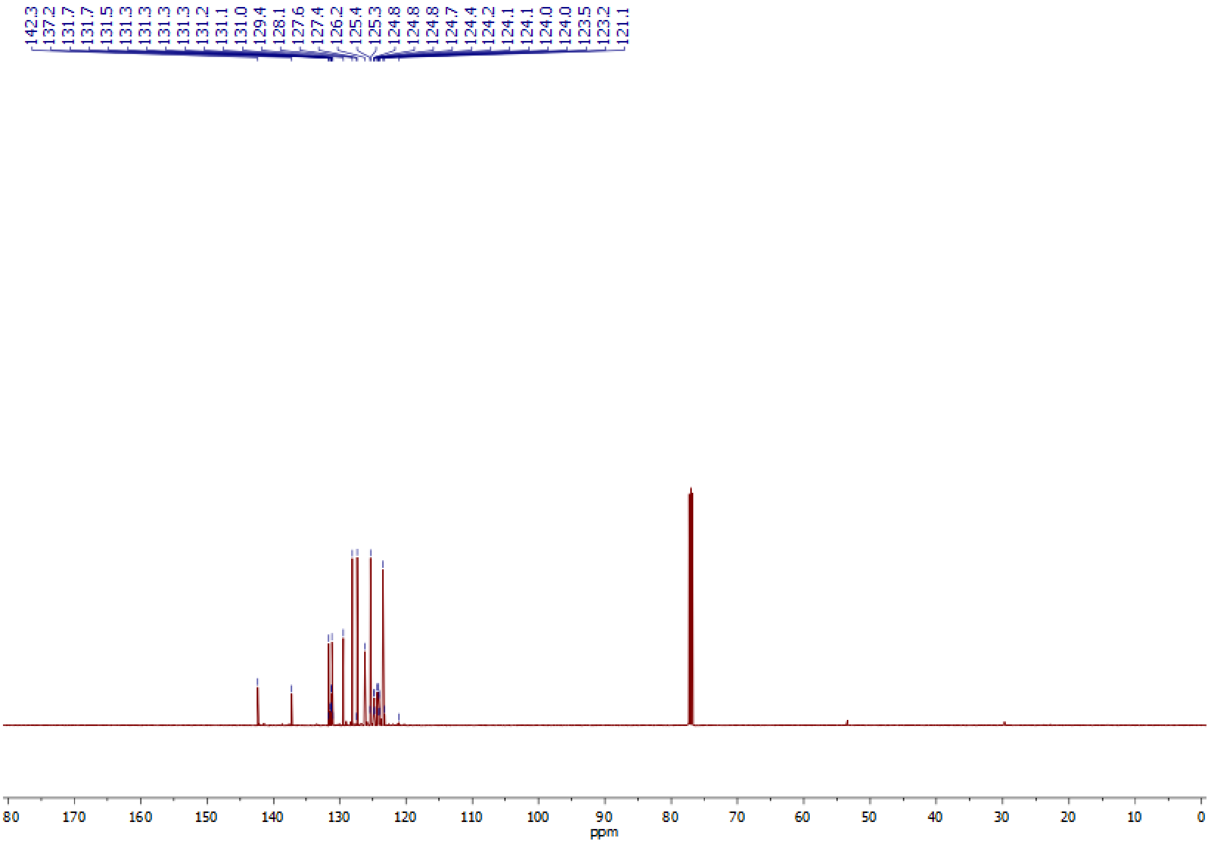

^{19}F-NMR (471 MHz, $CDCl_3$)

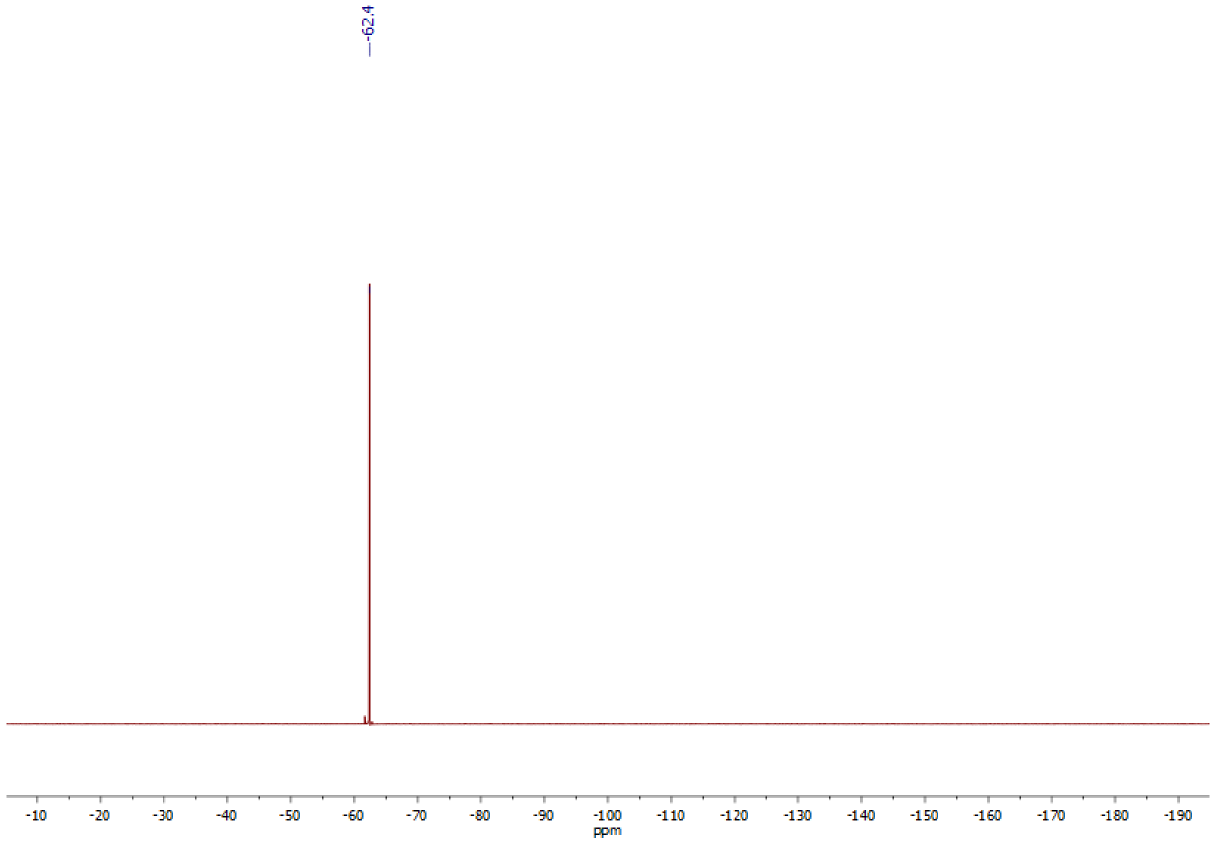

2-(3-Cyanophenyl)pyrene (5n)

^{1}H-NMR (500 MHz, $CDCl_3$)

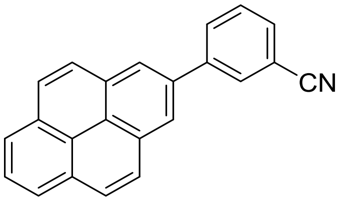

5n

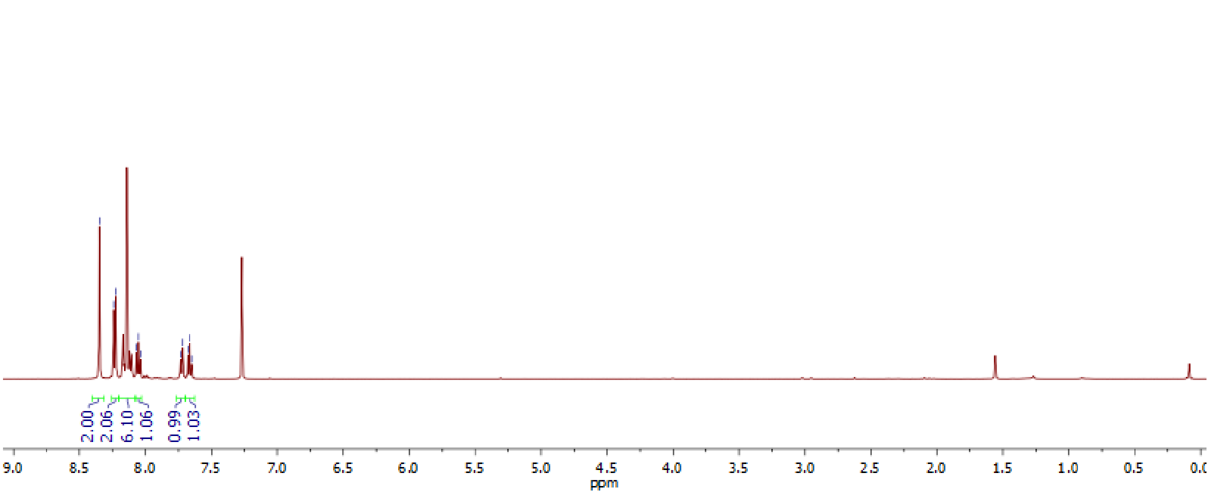

^{13}C-NMR (125 MHz, $CDCl_3$)

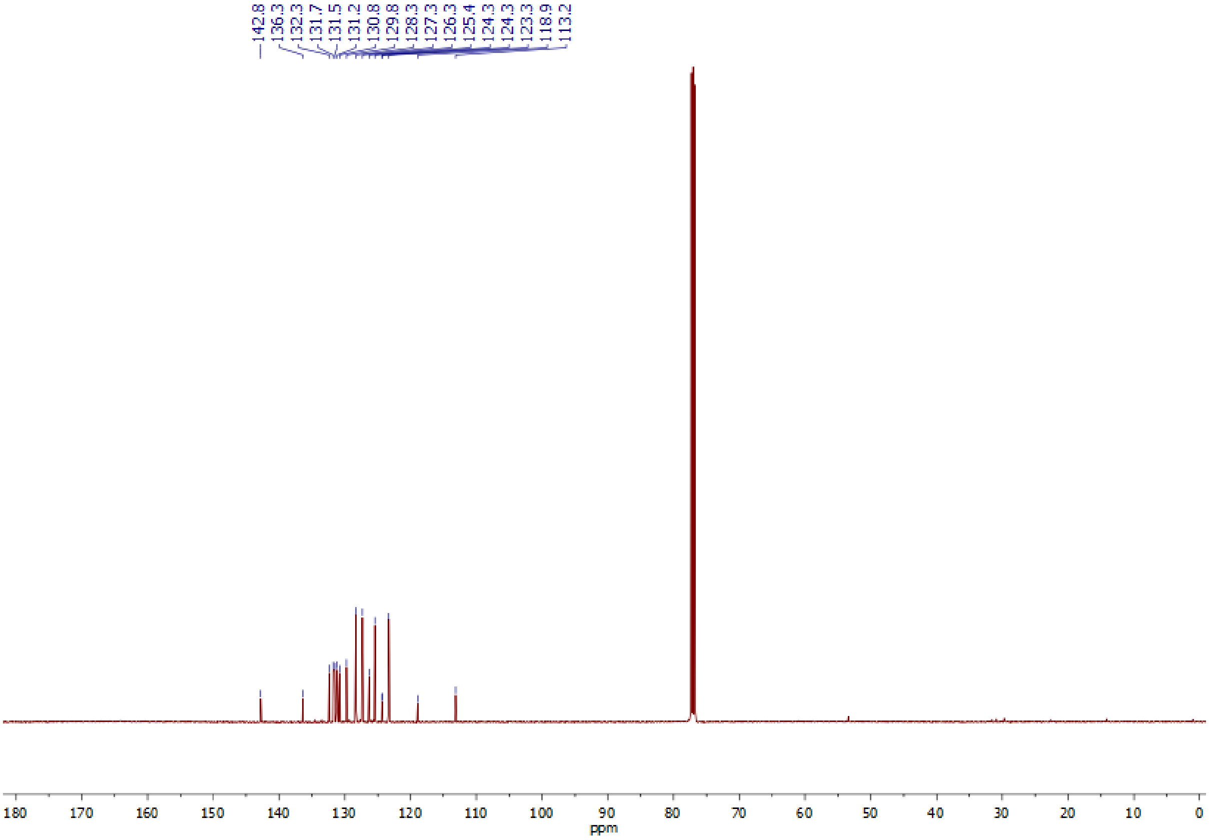

Methyl 3-(pyren-2-yl)benzoate (5o)

^{1}H-NMR (400 MHz, $CDCl_3$)

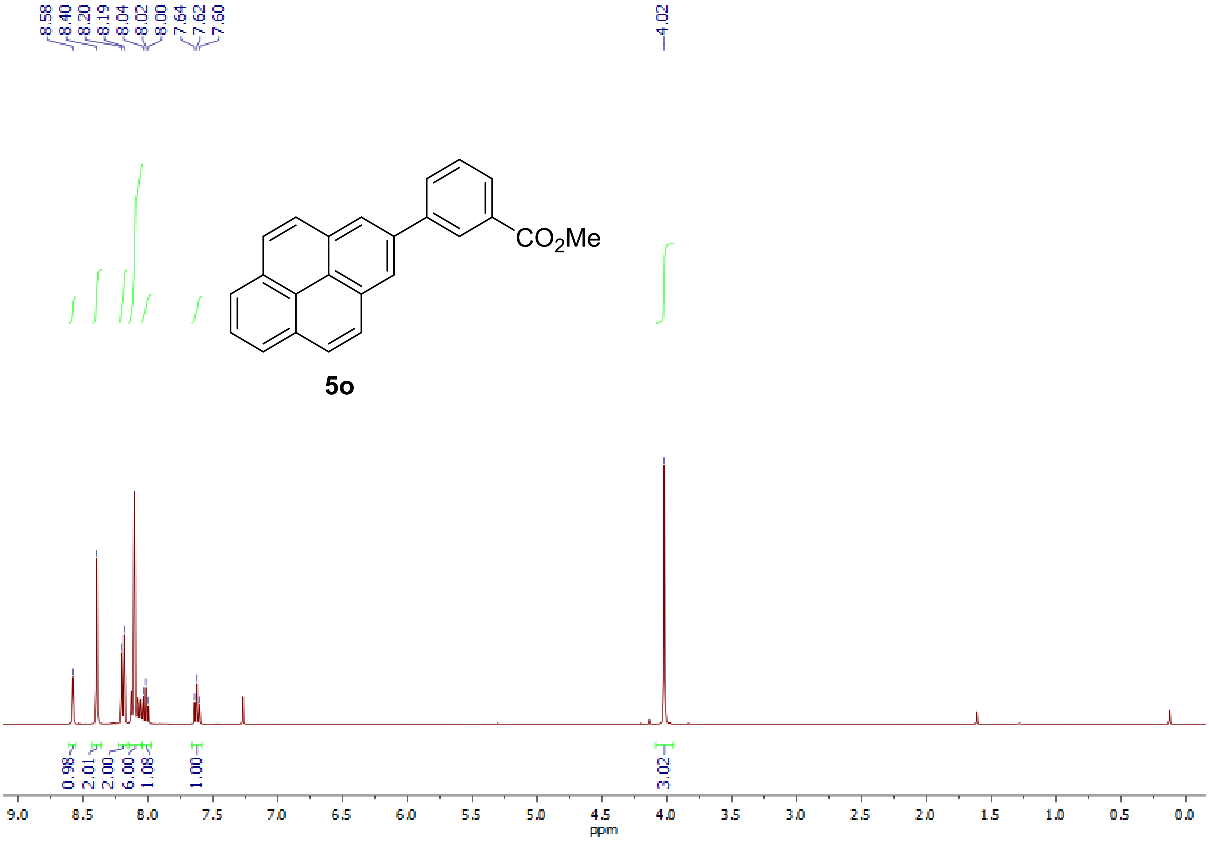

^{13}C-NMR (125 MHz, $CDCl_3$)

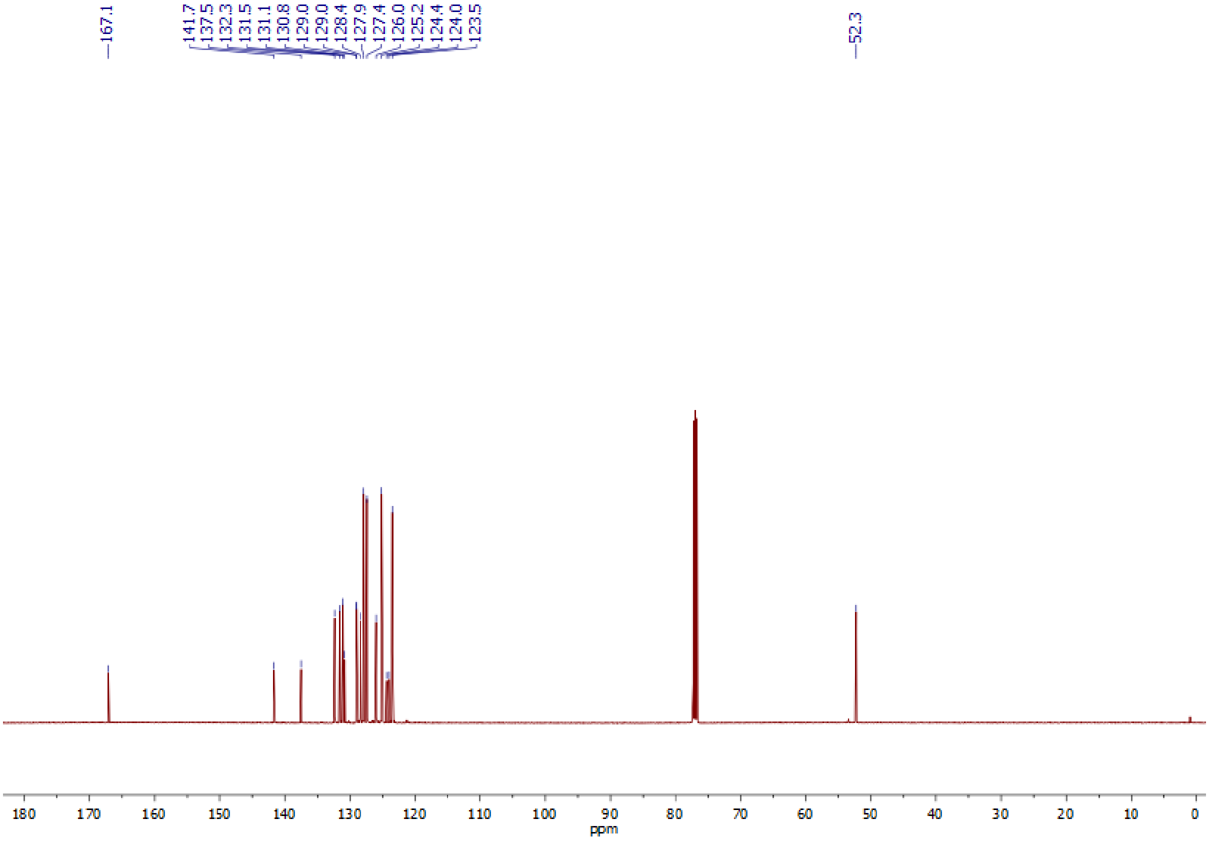

Methyl 4-(pyren-2-yl)benzoate (5p)

^{1}H-NMR (500 MHz, $CDCl_3$)

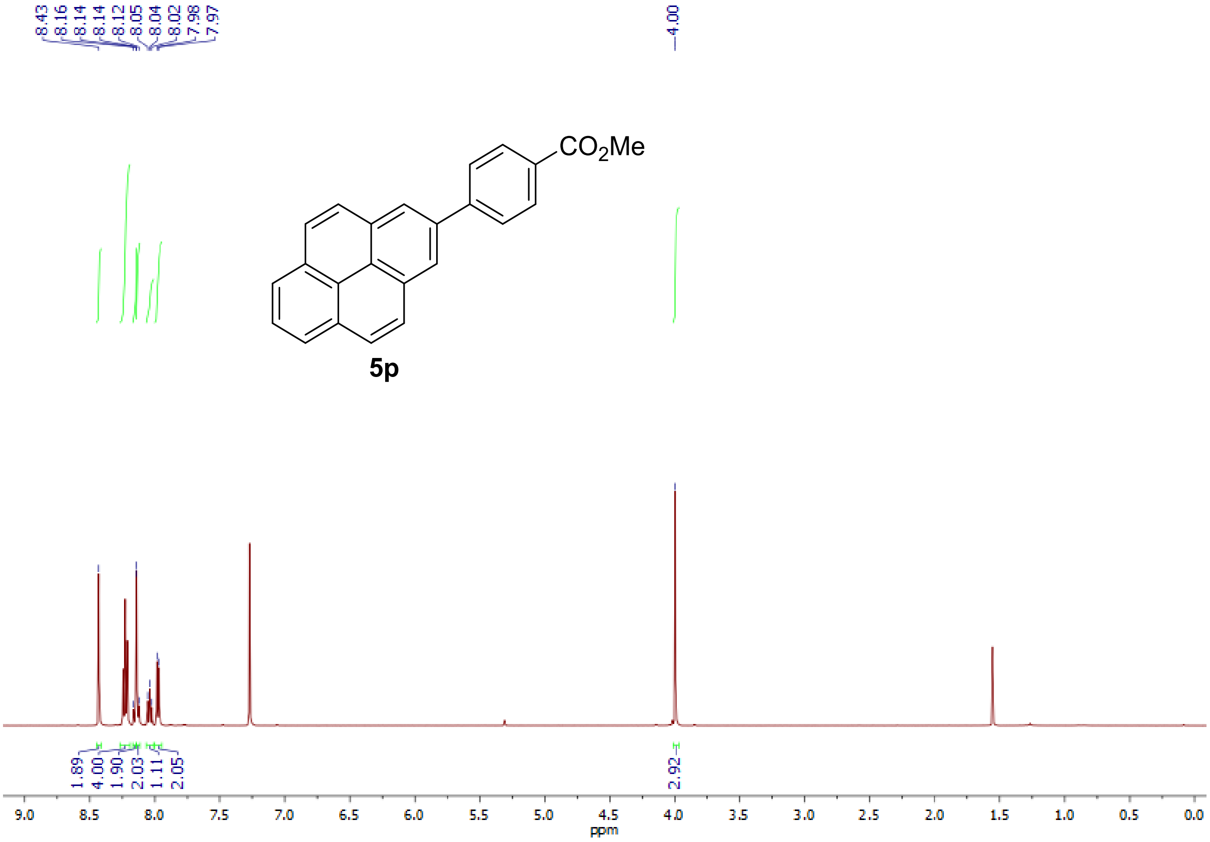

^{13}C-NMR (125 MHz, $CDCl_3$)

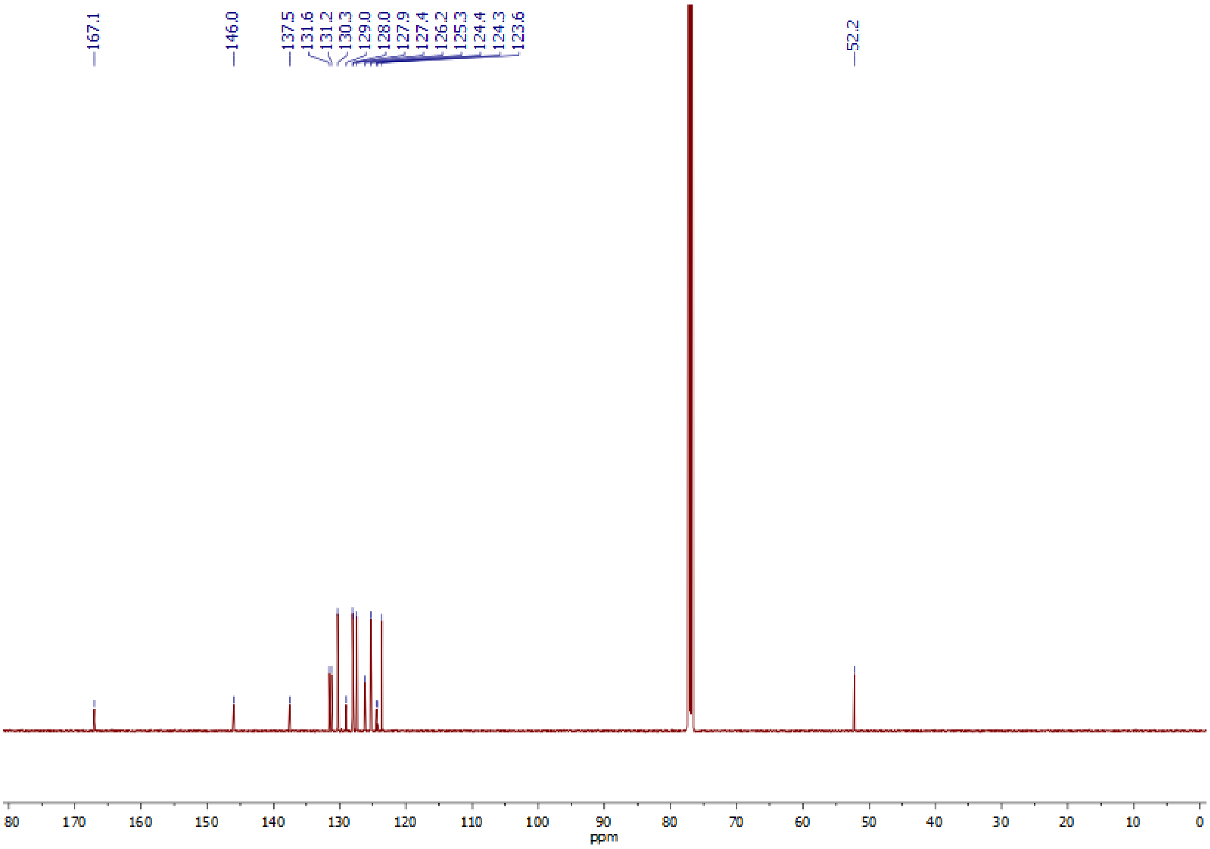

2-(3,5-Dimethylphenyl)-1-(phenylethynyl)pyrene (6a)

^{1}H-NMR (500 MHz, $CDCl_3$)

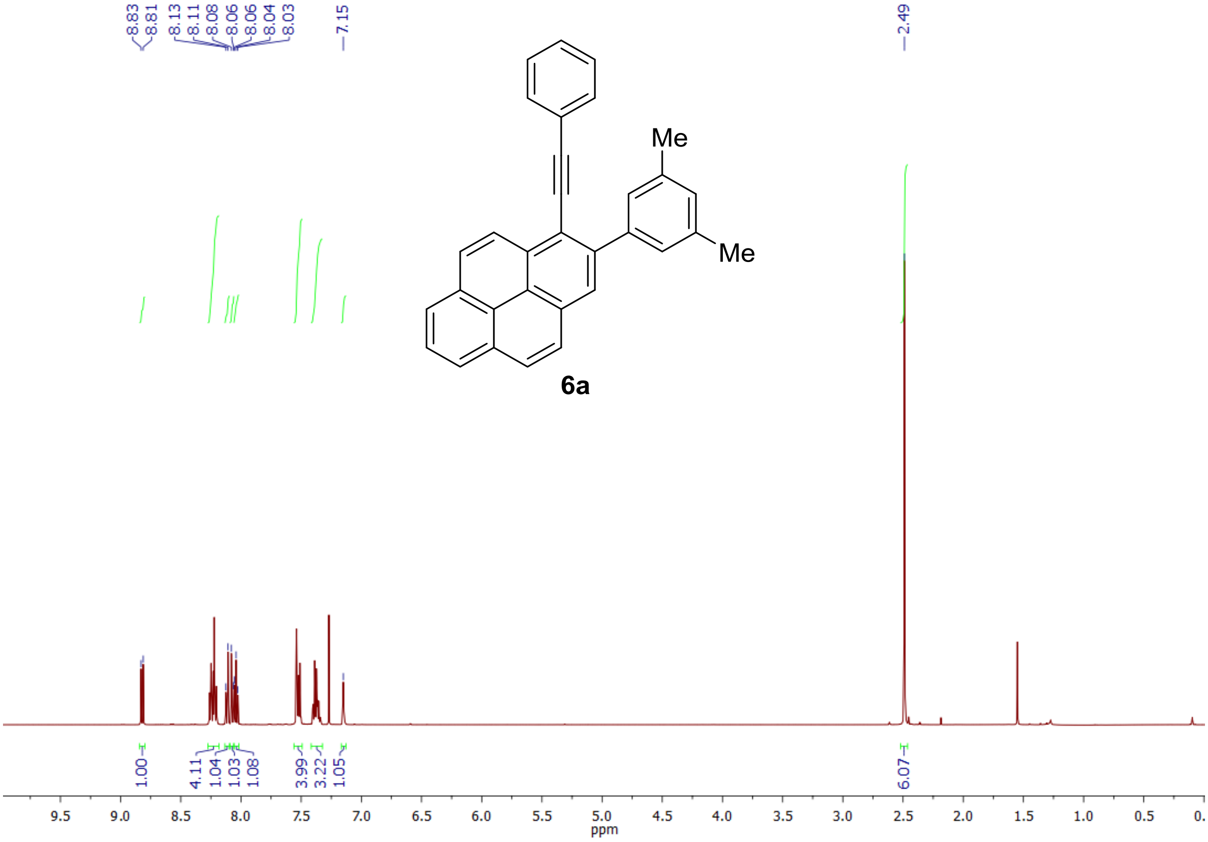

^{13}C-NMR (125 MHz, $CDCl_3$)

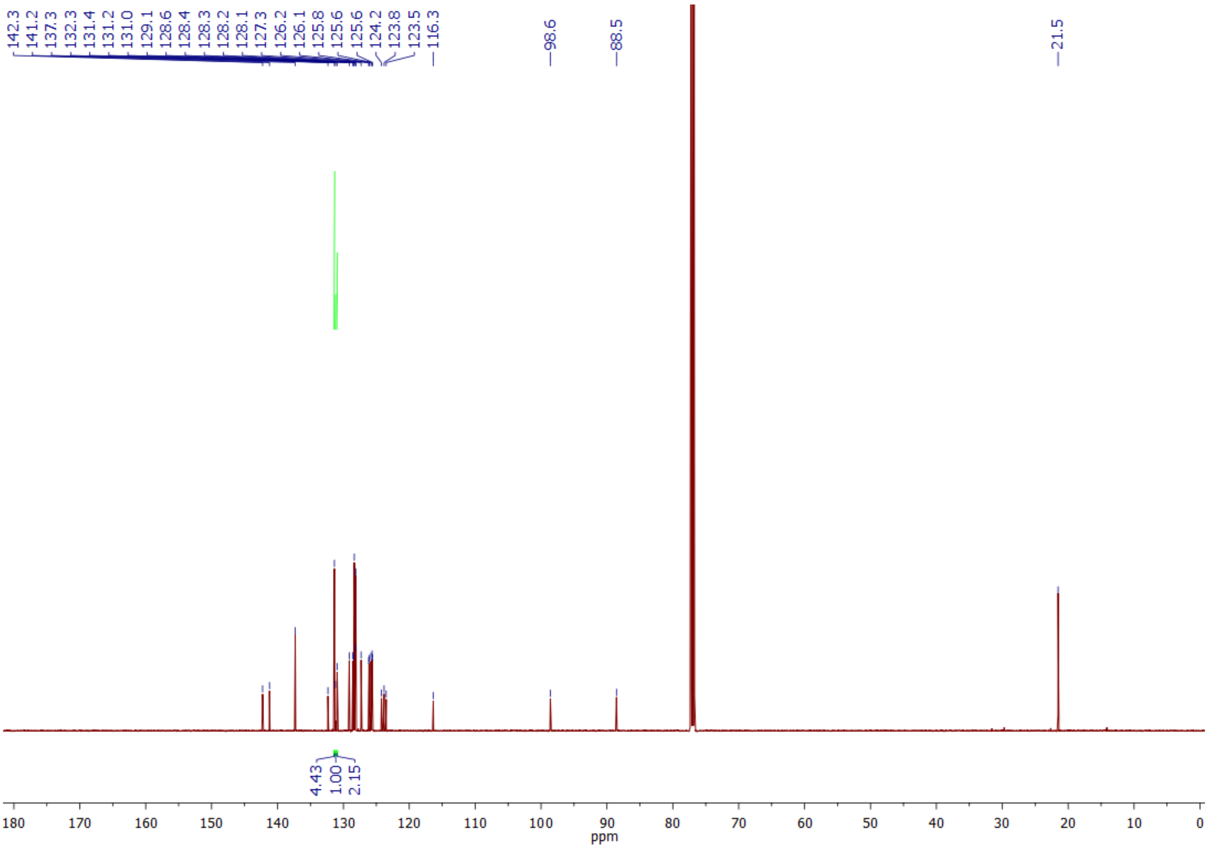

2-(3,5-Dimethylphenyl)-1-(4-trifluoromethylphenylethynyl)pyrene (6b)

^{1}H-NMR (400 MHz, $CDCl_3$)

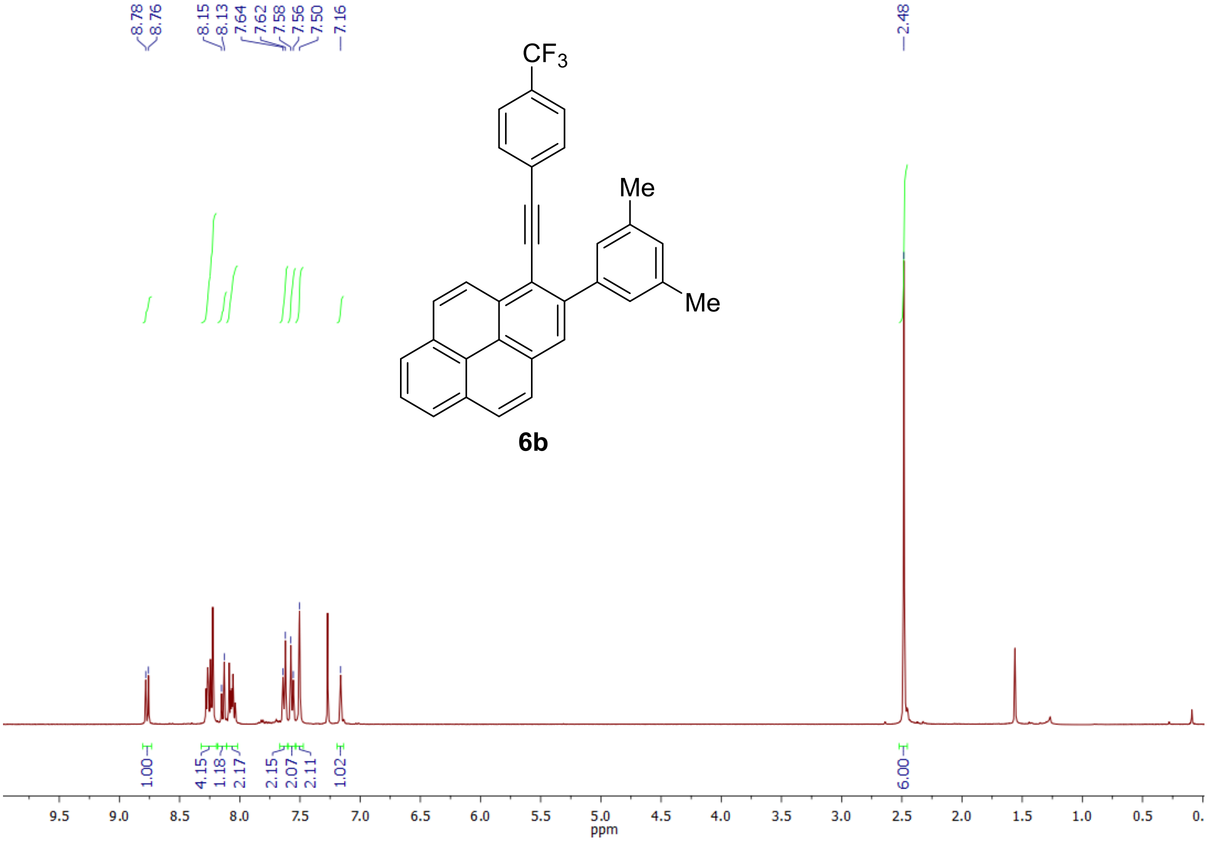

^{13}C-NMR (125 MHz, $CDCl_3$)

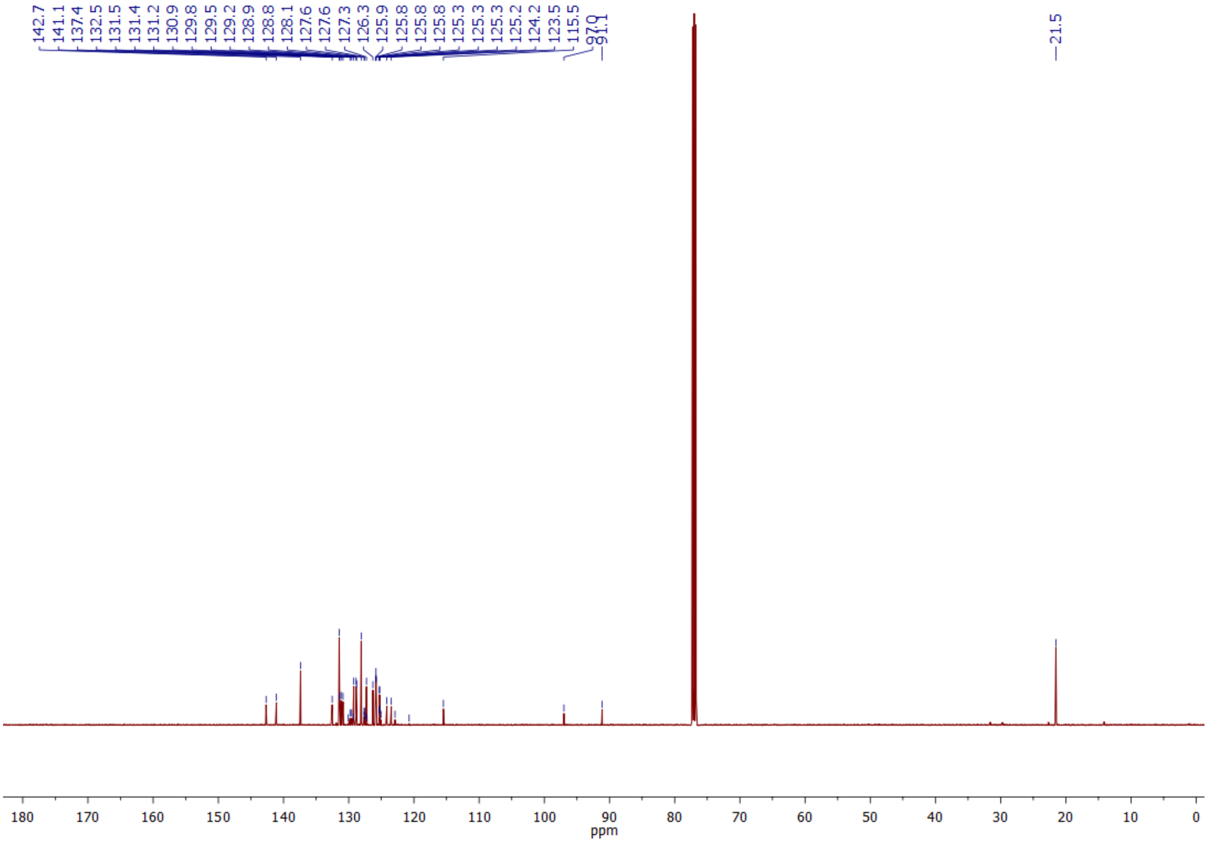

^{19}F-NMR (376 MHz, $CDCl_3$)

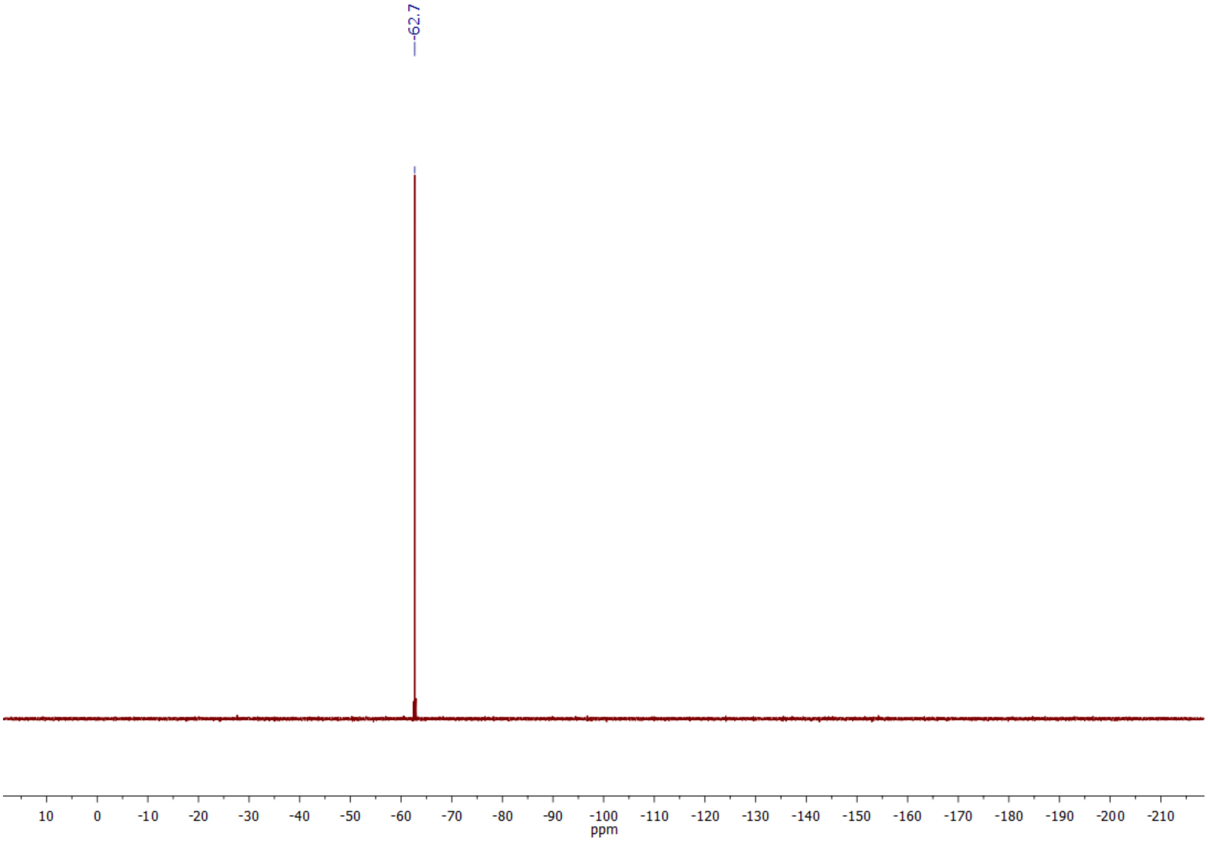

2-(3,5-Dimethylphenyl)-1-(trimethylsilylethynyl)pyrene (6c)

^{1}H-NMR (500 MHz, $CDCl_3$)

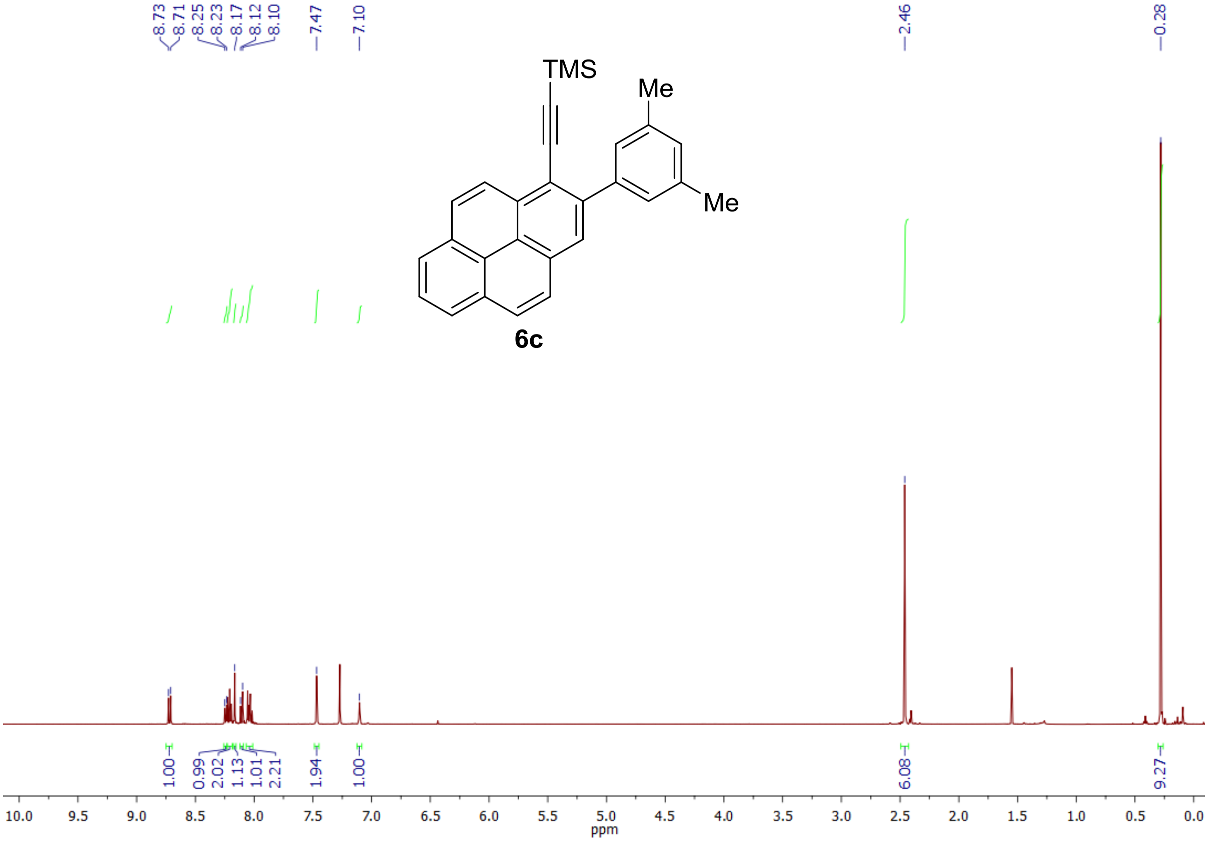

^{13}C-NMR (125 MHz, $CDCl_3$)

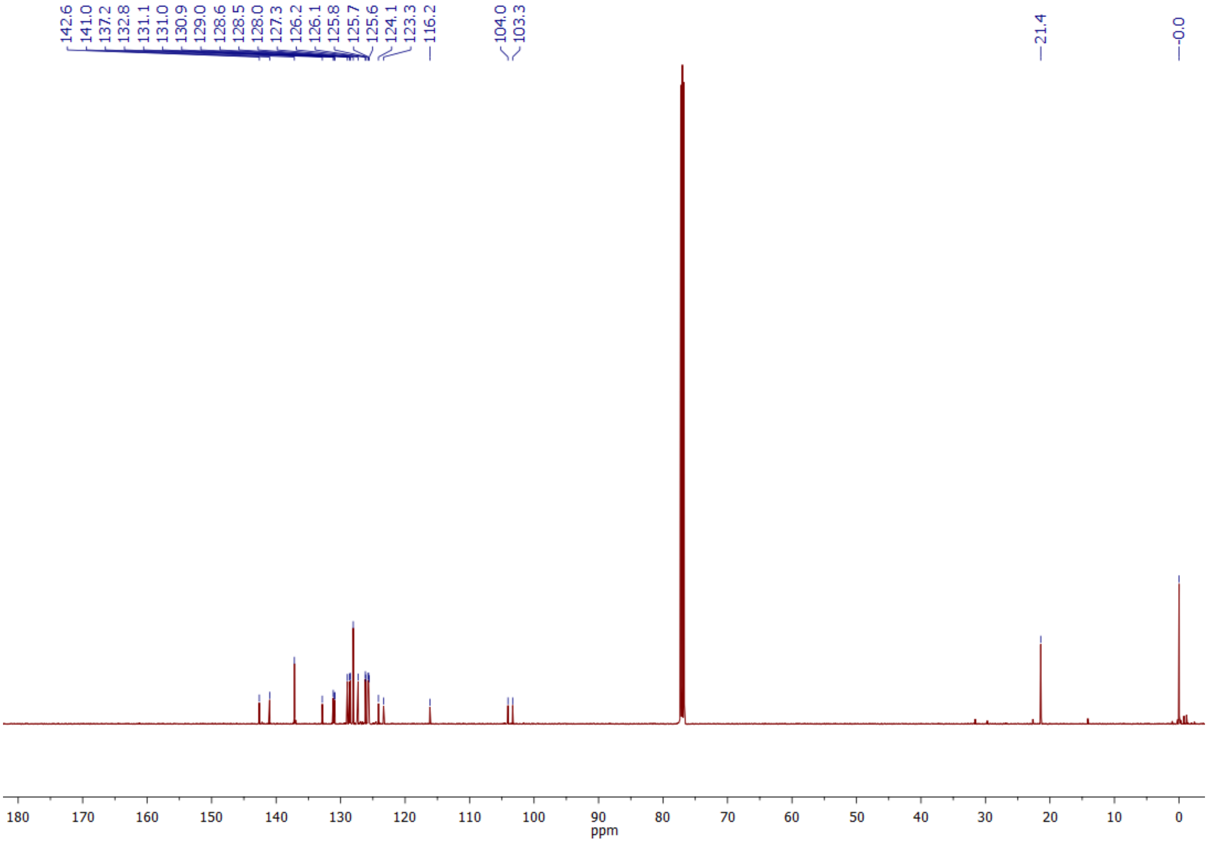

2-(4-Methylphenyl)-1-(phenylethynyl)pyrene (6d)

^{1}H-NMR (400 MHz, $CDCl_3$)

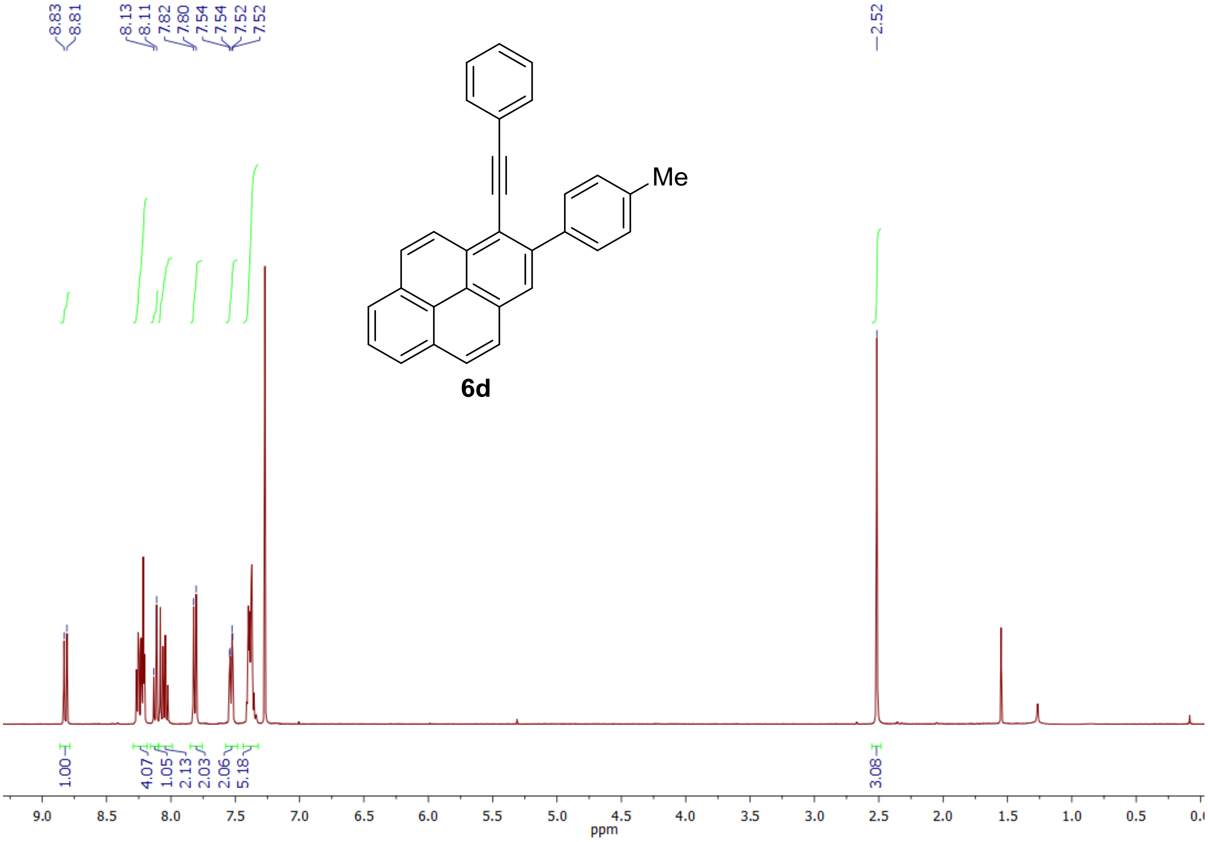

^{13}C-NMR (125 MHz, $CDCl_3$)

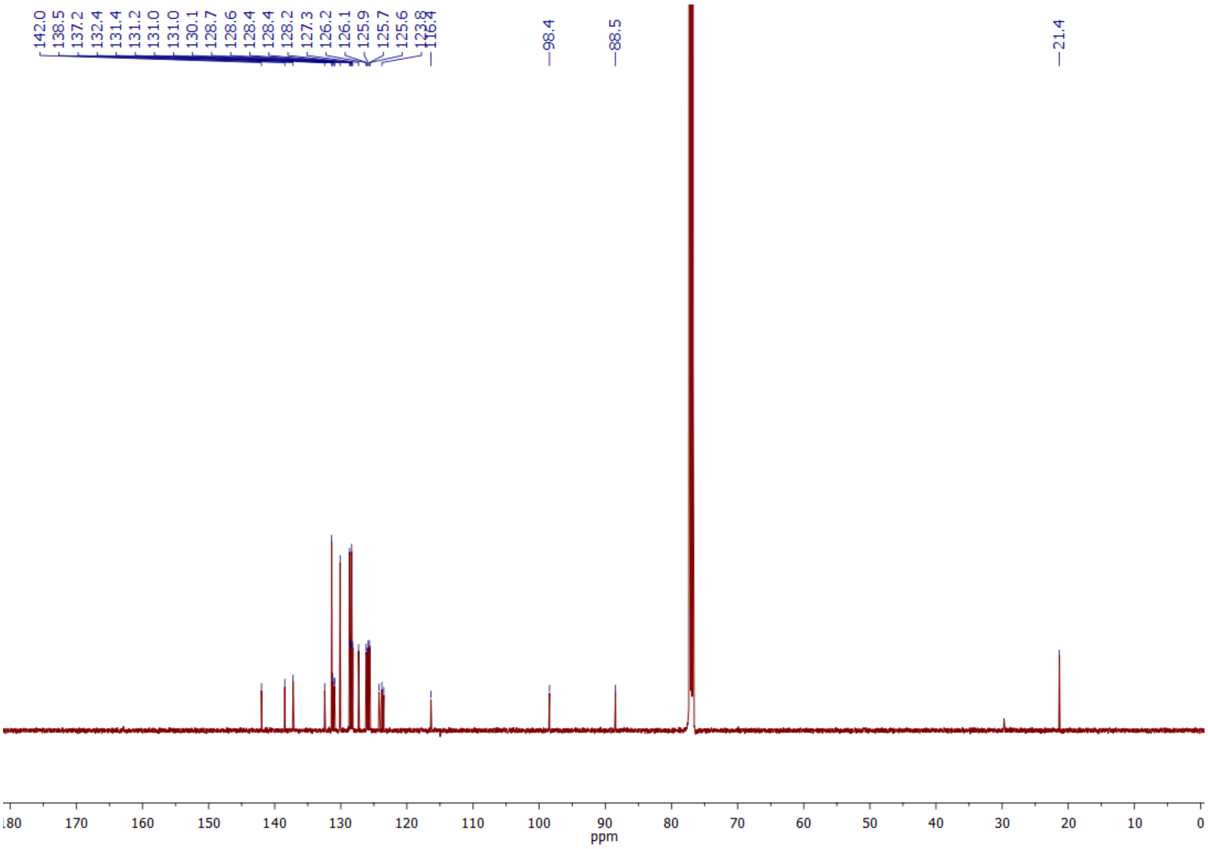

2-(4-Methylphenyl)-1-(trimethylsilylethynyl)pyrene (6e)

^{1}H-NMR (500 MHz, $CDCl_3$)

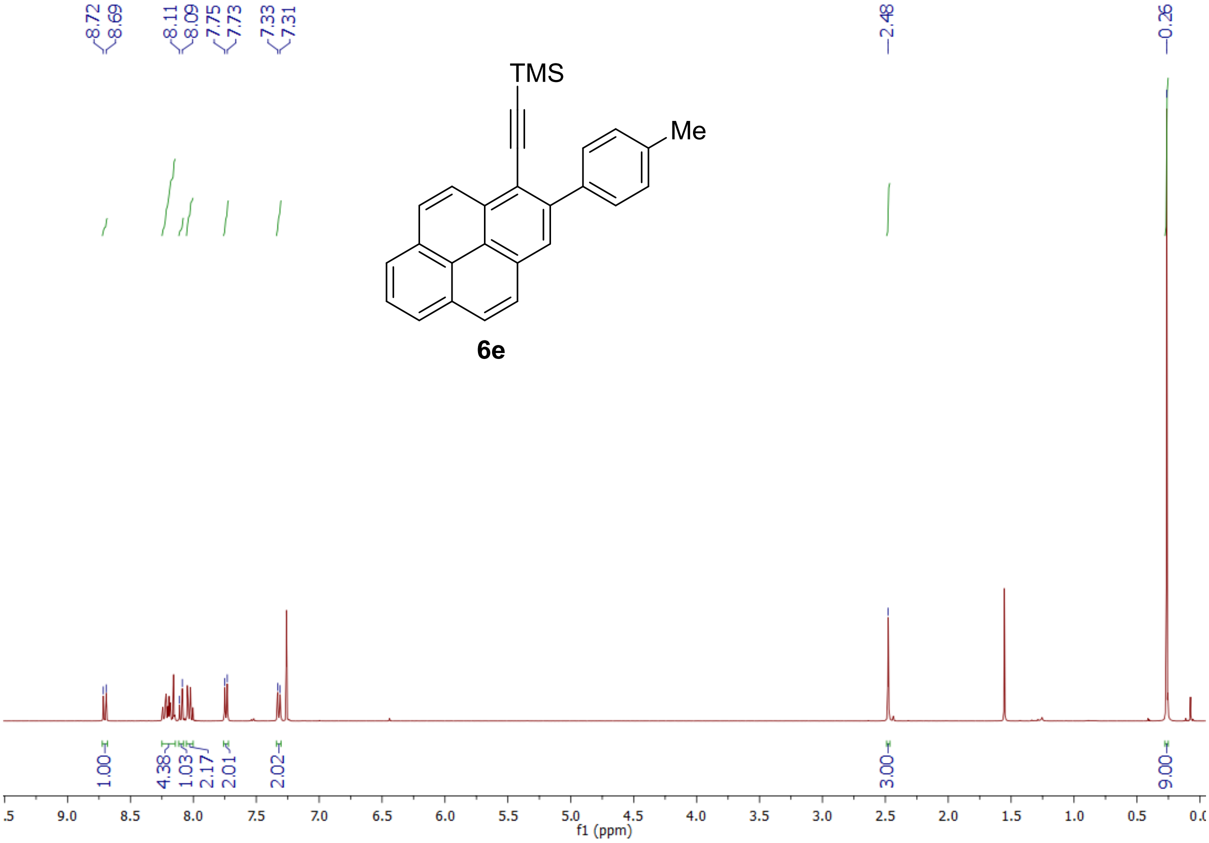

^{13}C-NMR (125 MHz, $CDCl_3$)

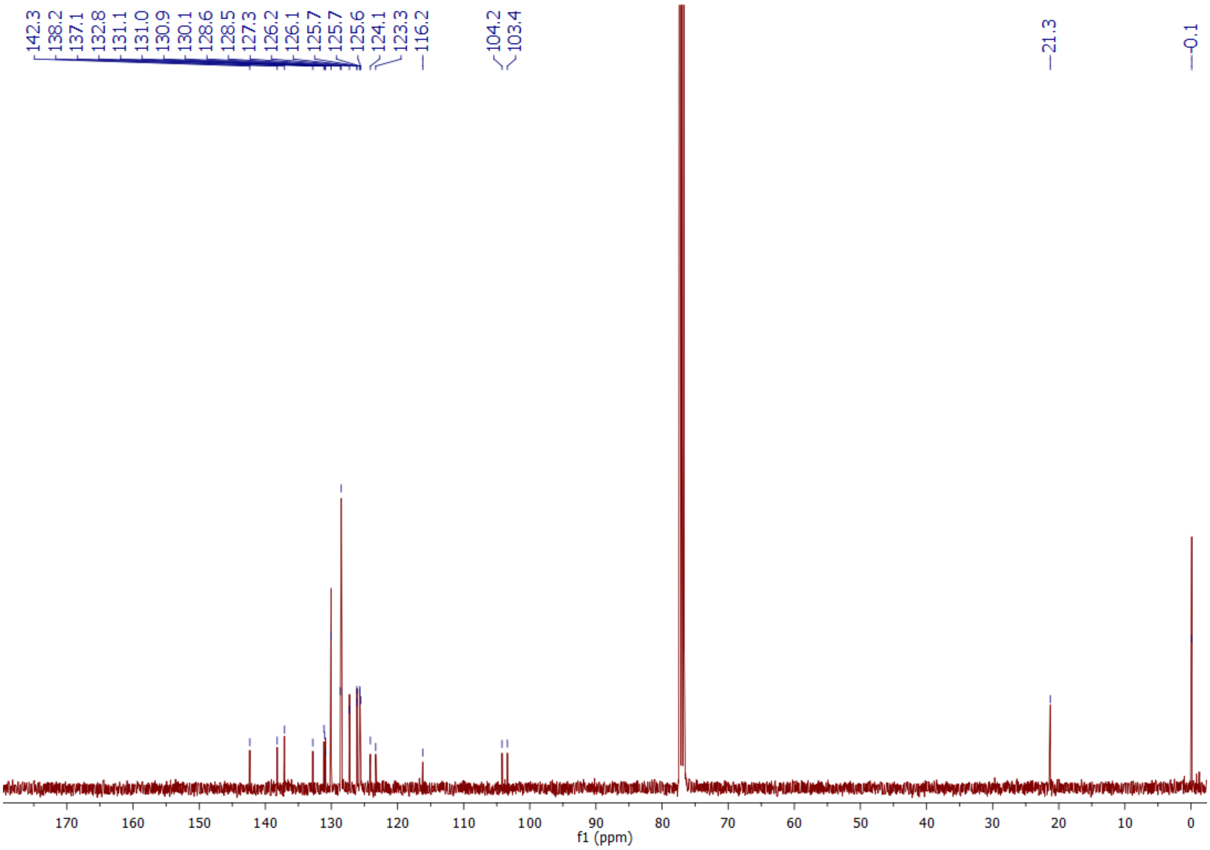

1-(4-Fluorophenylethynyl)-2-(4-methylphenyl)pyrene (6f)

^{1}H-NMR (500 MHz, $CDCl_3$)

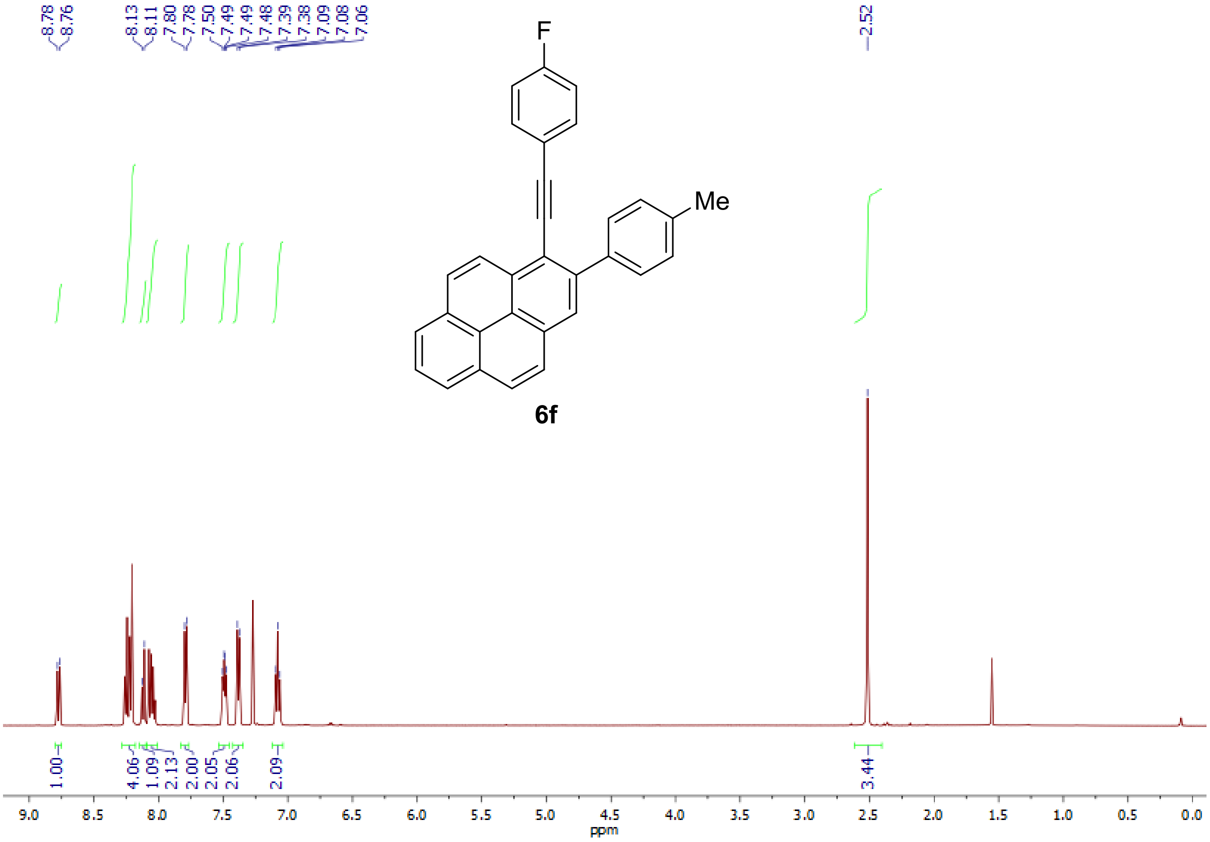

^{13}C-NMR (125 MHz, $CDCl_3$)

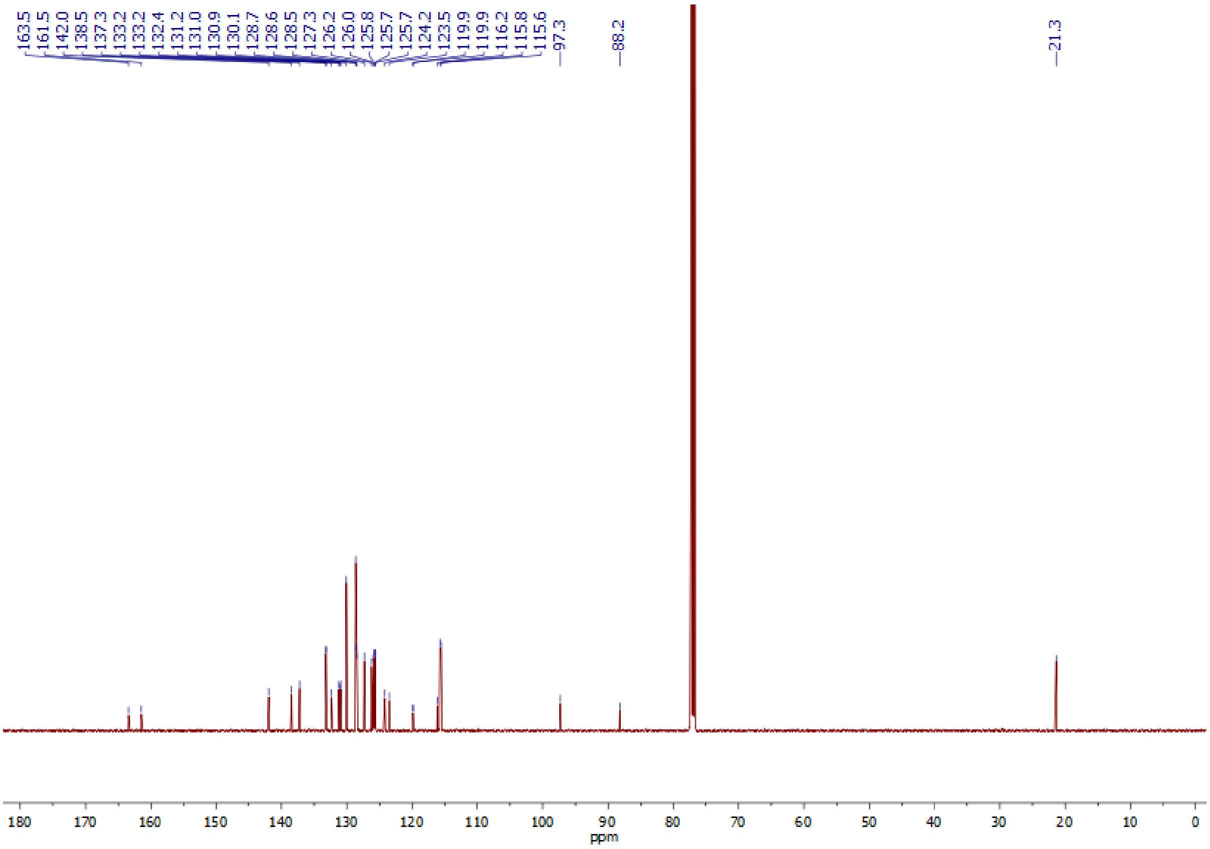

^{19}F-NMR (471 MHz, $CDCl_3$)

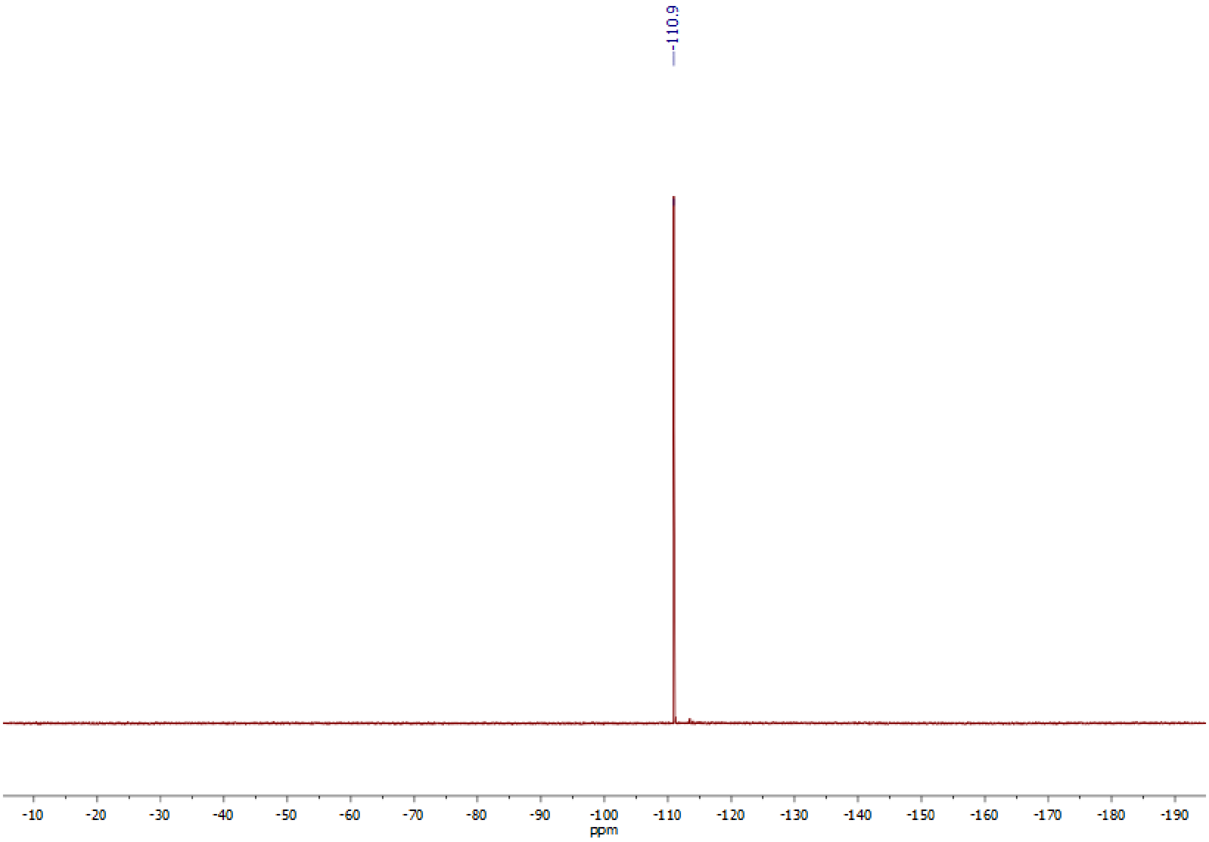

2-(4-Methoxyphenyl)-1-(phenylethynyl)pyrene (6g)

^{1}H-NMR (500 MHz, $CDCl_3$)

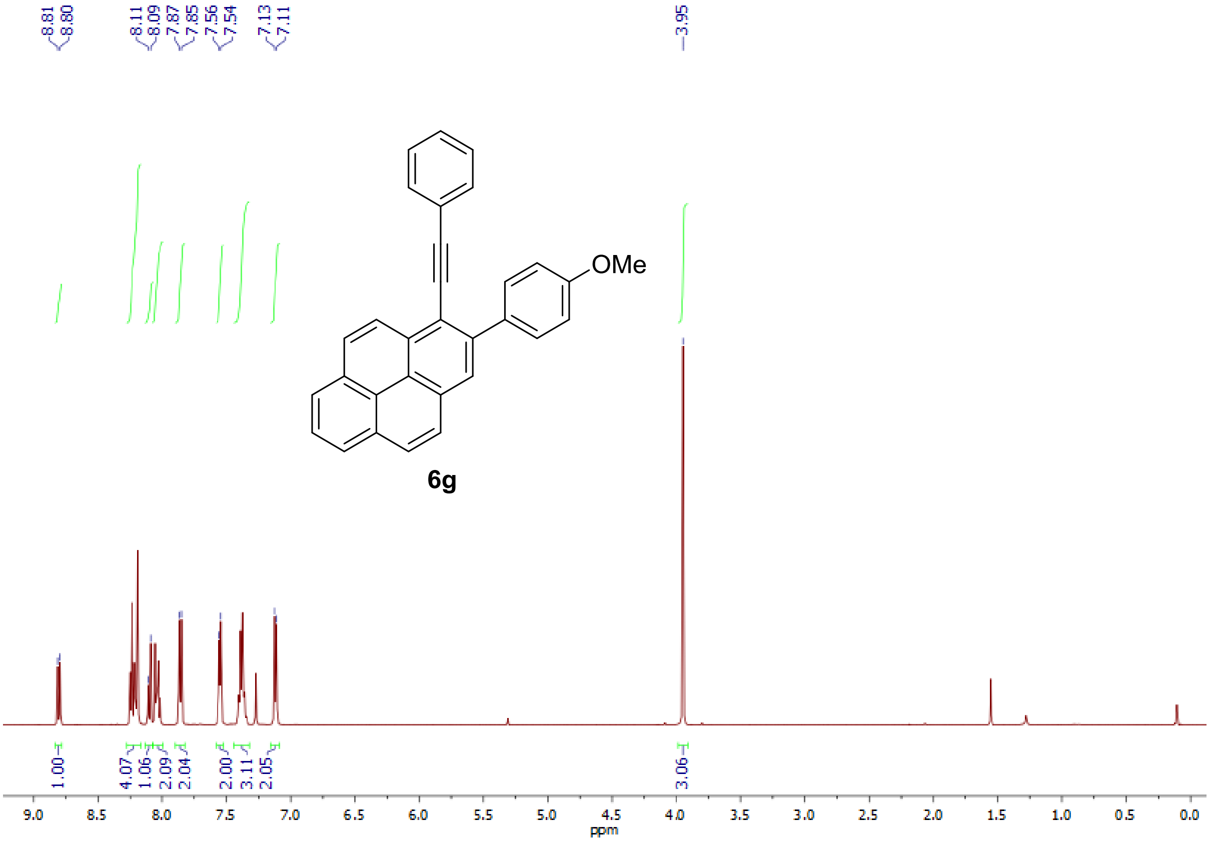

^{13}C-NMR (125 MHz, $CDCl_3$)

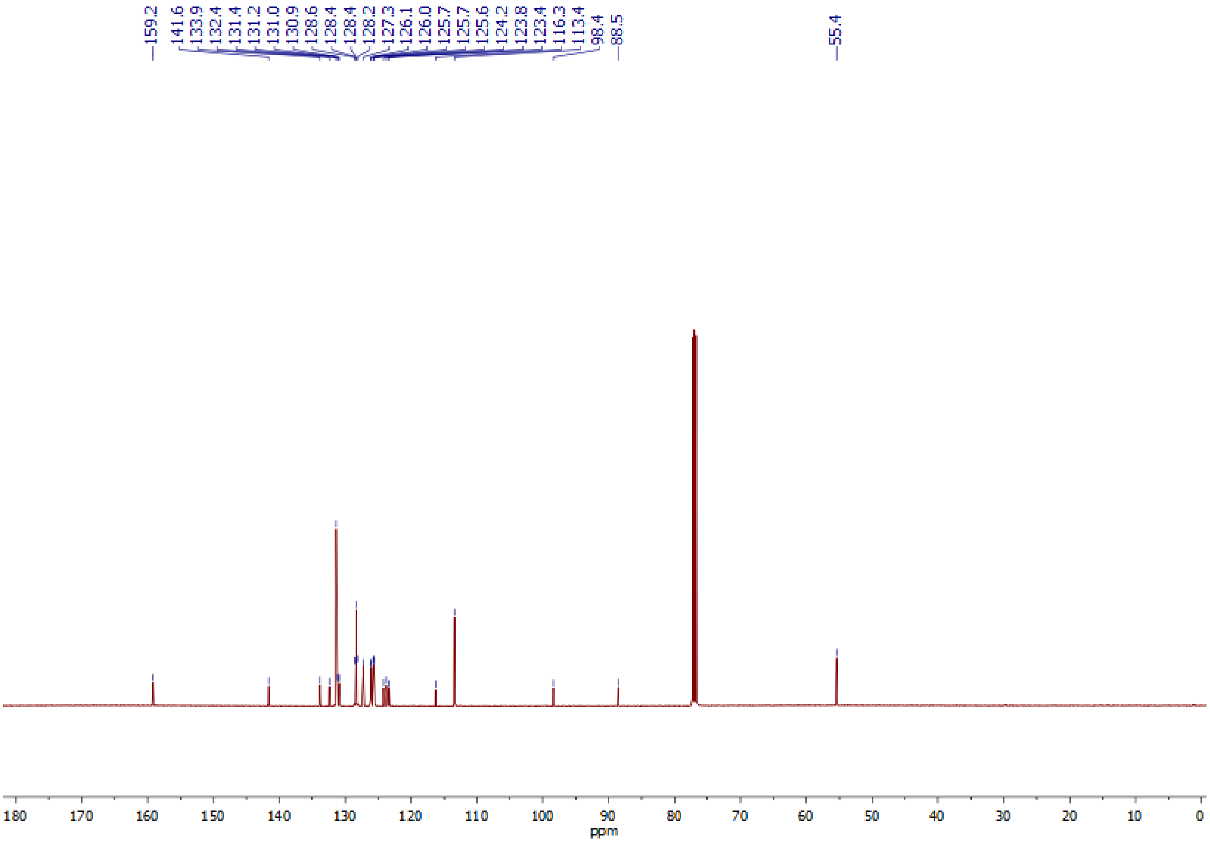

2-(3,5-Dimethylphenyl)-1-(4-ethoxycarbonylphenyl)pyrene (7a)

^{1}H-NMR (400 MHz, $CDCl_3$)

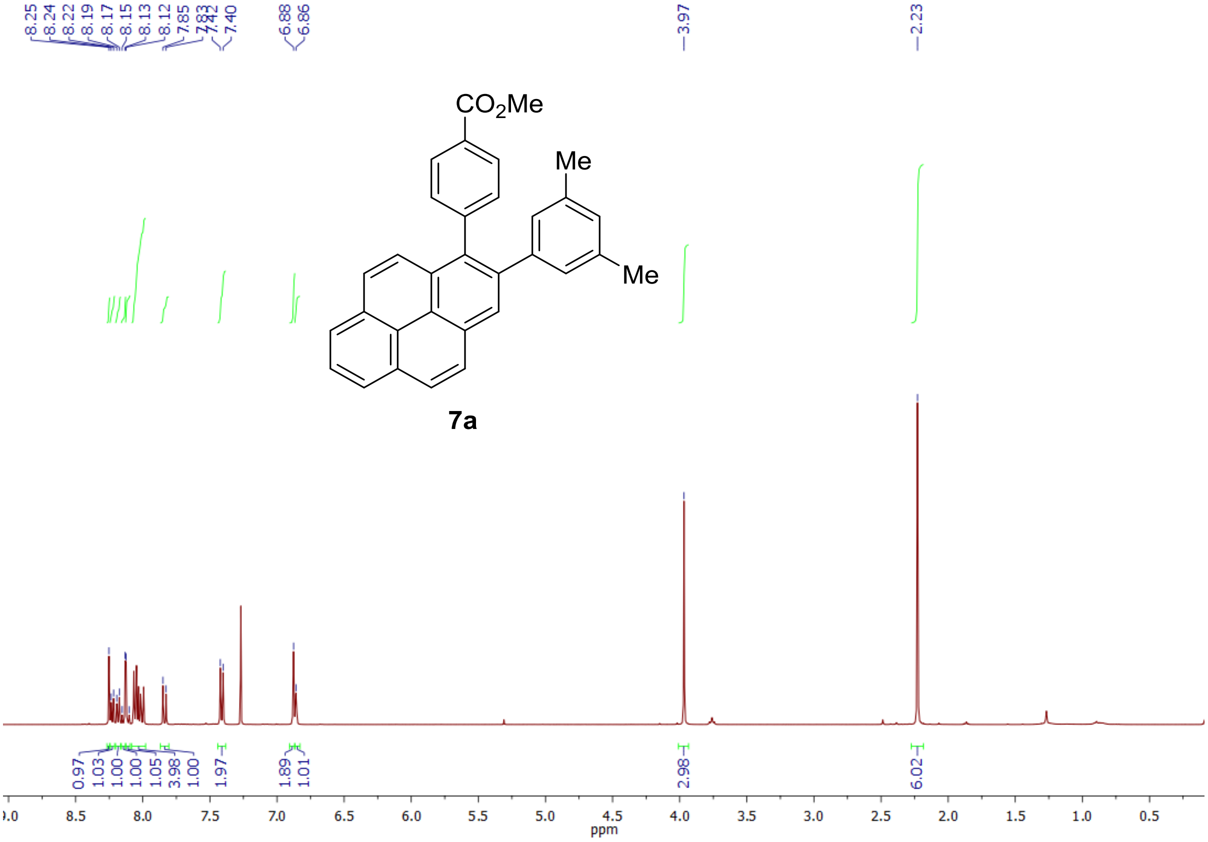

^{13}C-NMR (100 MHz, $CDCl_3$)

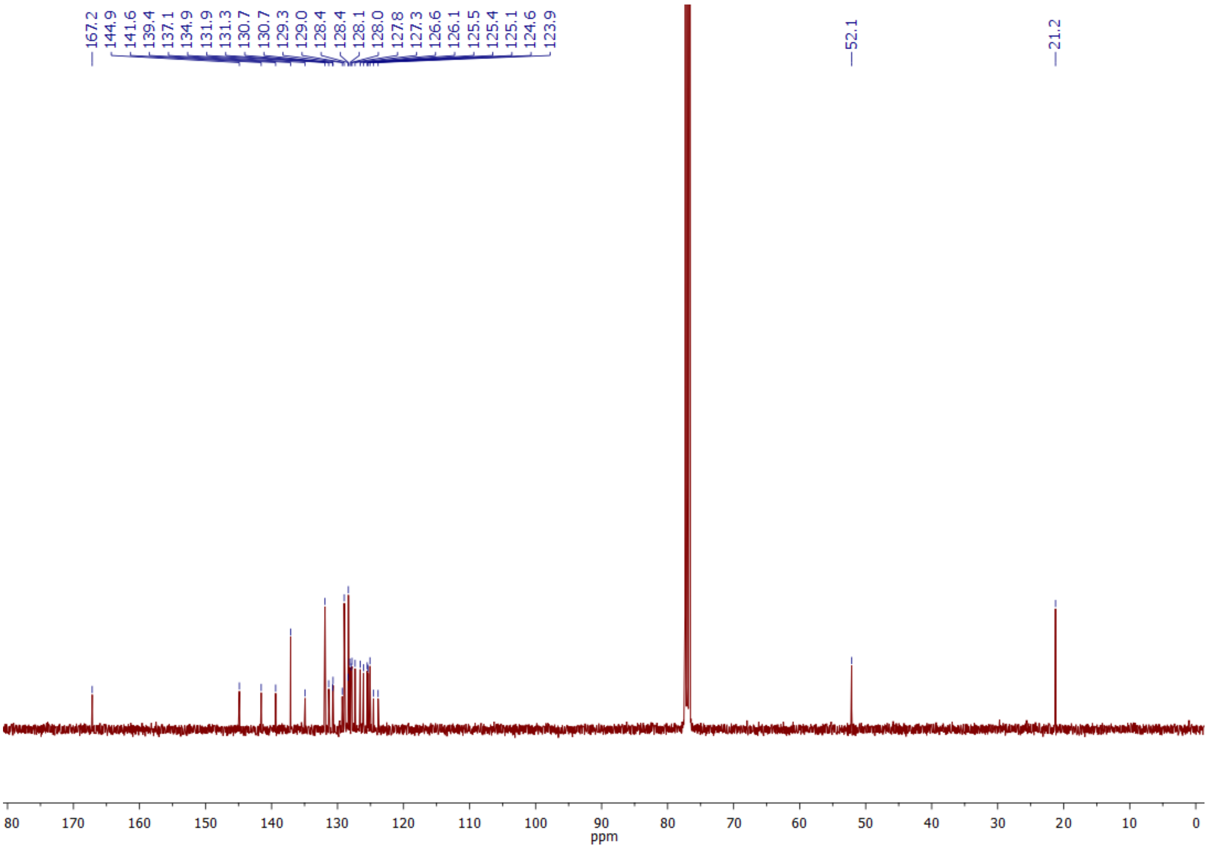

2-(3,5-Dimethylphenyl)-1-(4-methoxyphenyl)pyrene (7b)

^{1}H-NMR (400 MHz, $CDCl_3$)

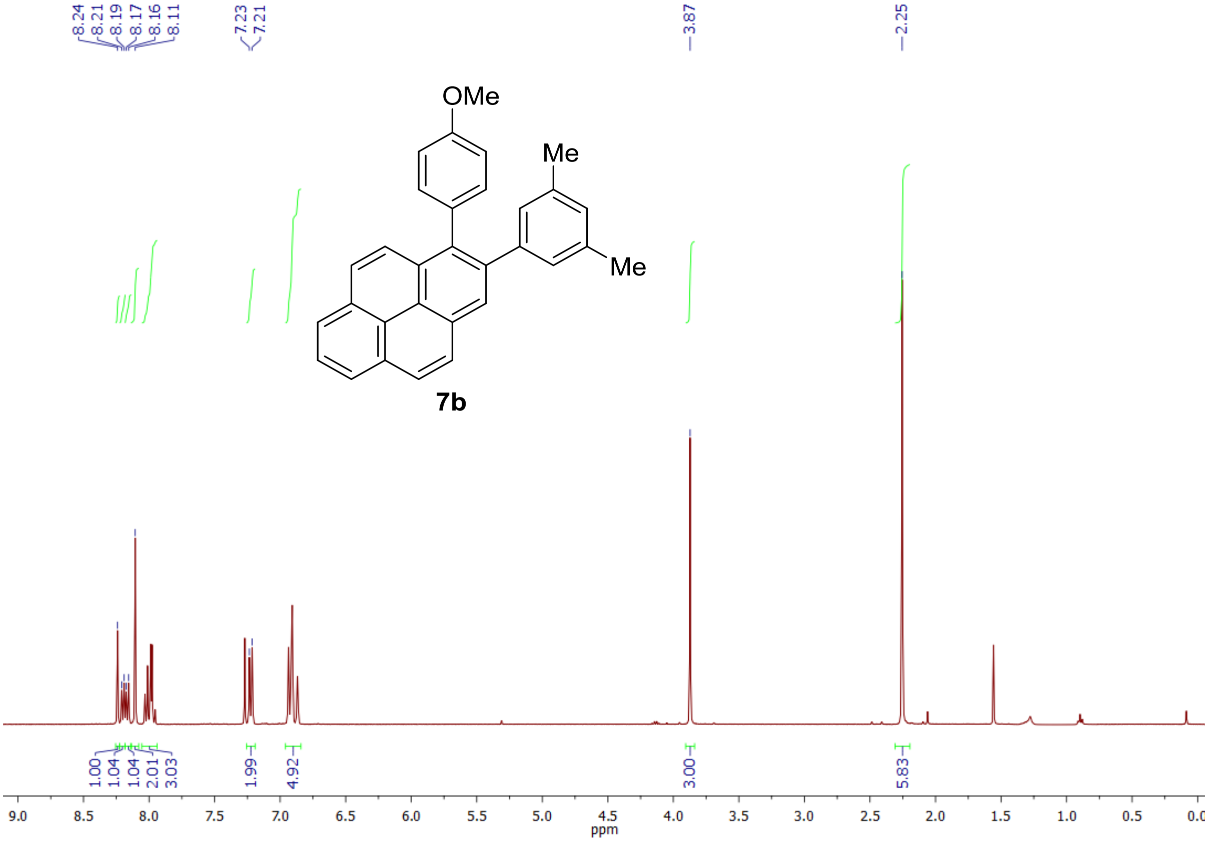

^{13}C-NMR (100 MHz, $CDCl_3$)

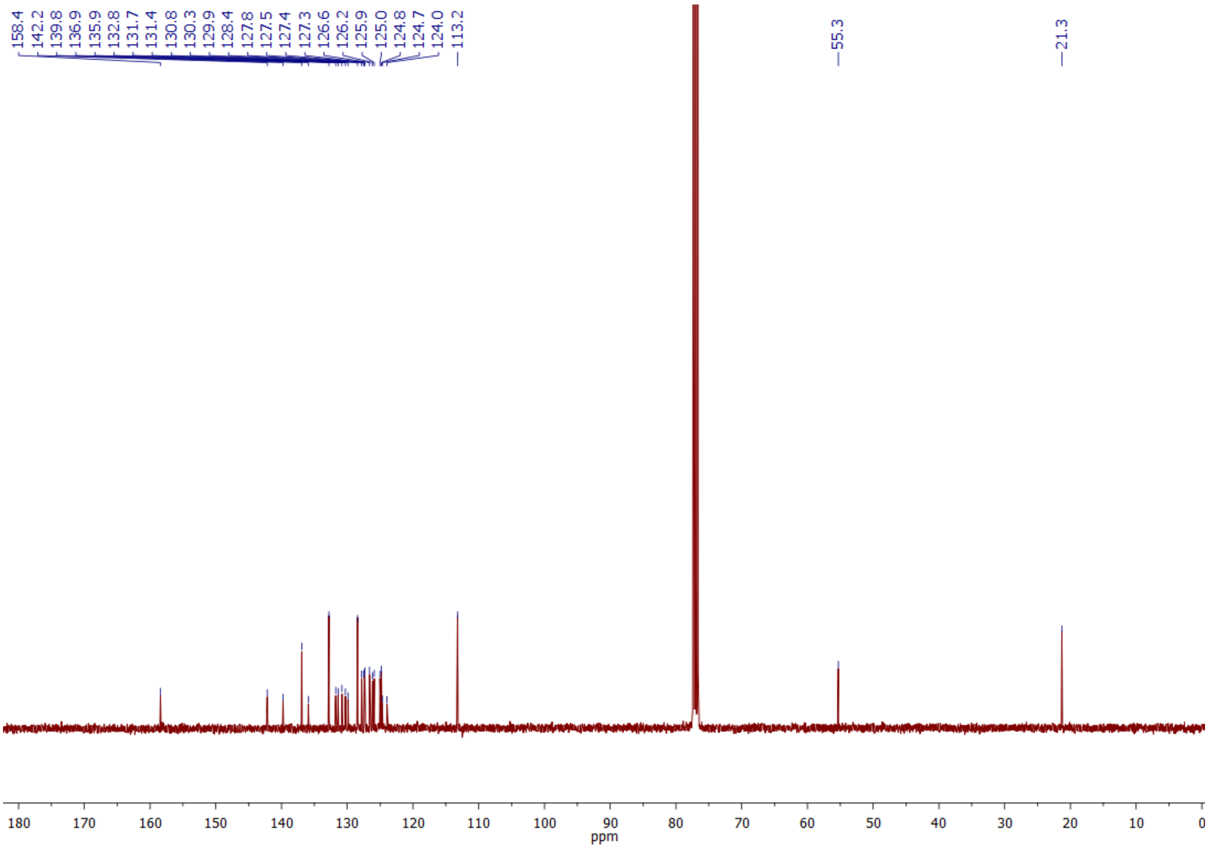

1-(4-Methoxyphenyl)-2-(4-methylphenyl)pyrene (7c)

^{1}H-NMR (500 MHz, $CDCl_3$)

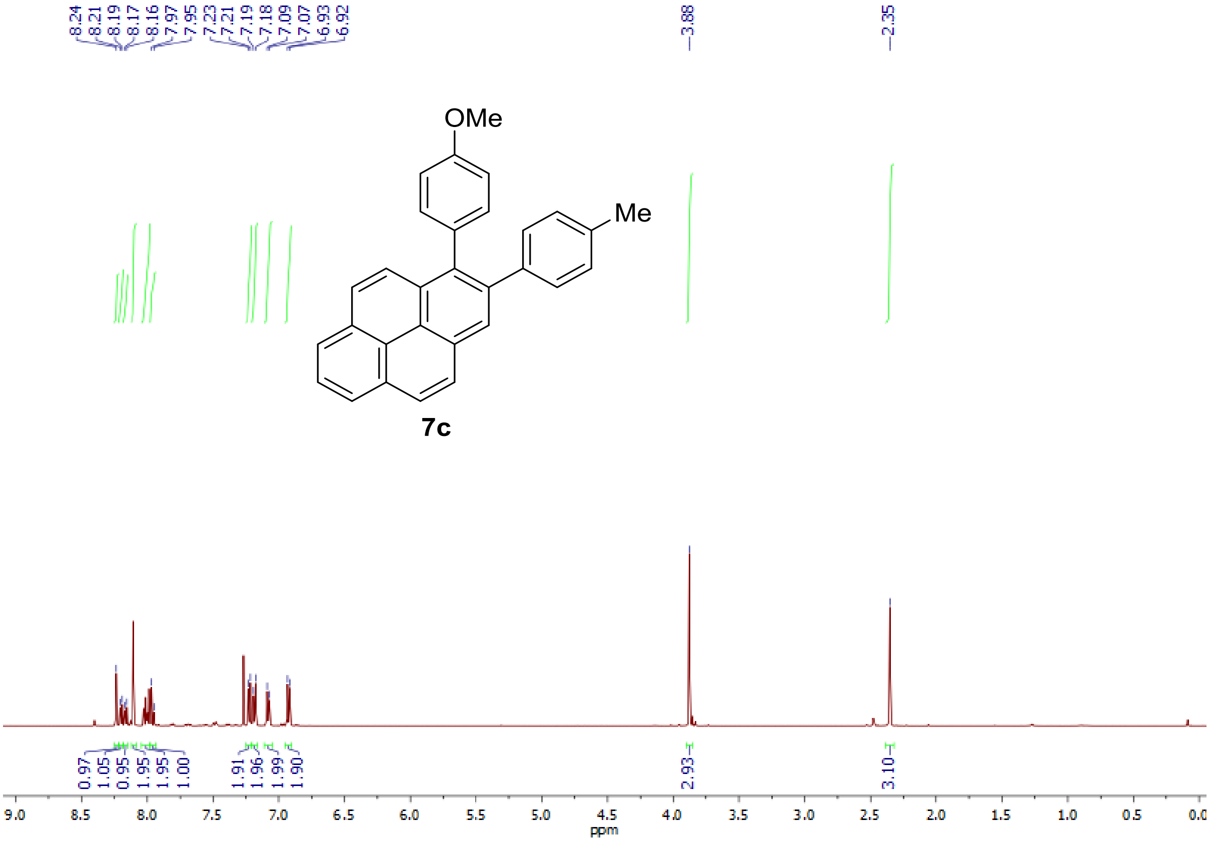

^{13}C-NMR (125 MHz, $CDCl_3$)

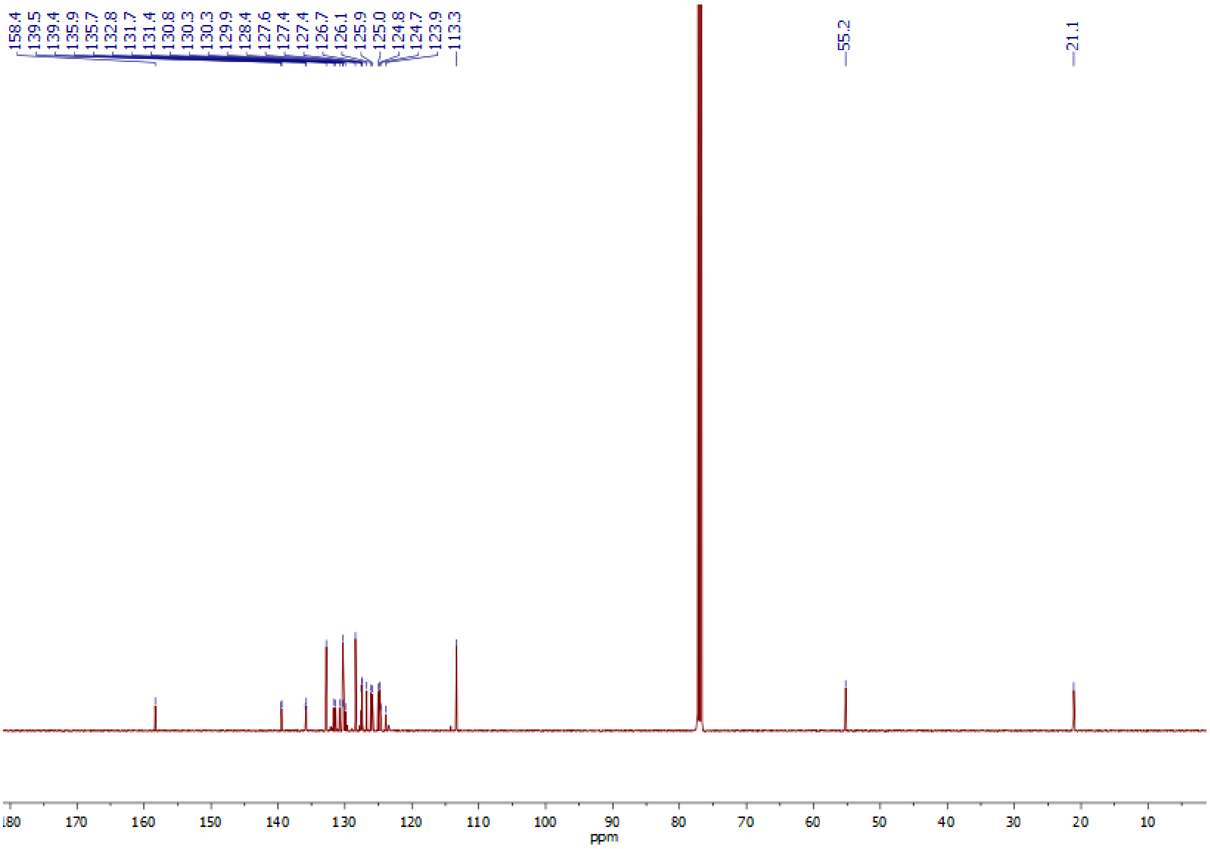

2-(6-(4-Methoxyphenyl)-7-(p-tolyl)pyren-2-yl)-4,4,5,5-tetramethyl-1,3,2-dioxaborolane (8)

^{1}H-NMR (400 MHz, $CDCl_3$)

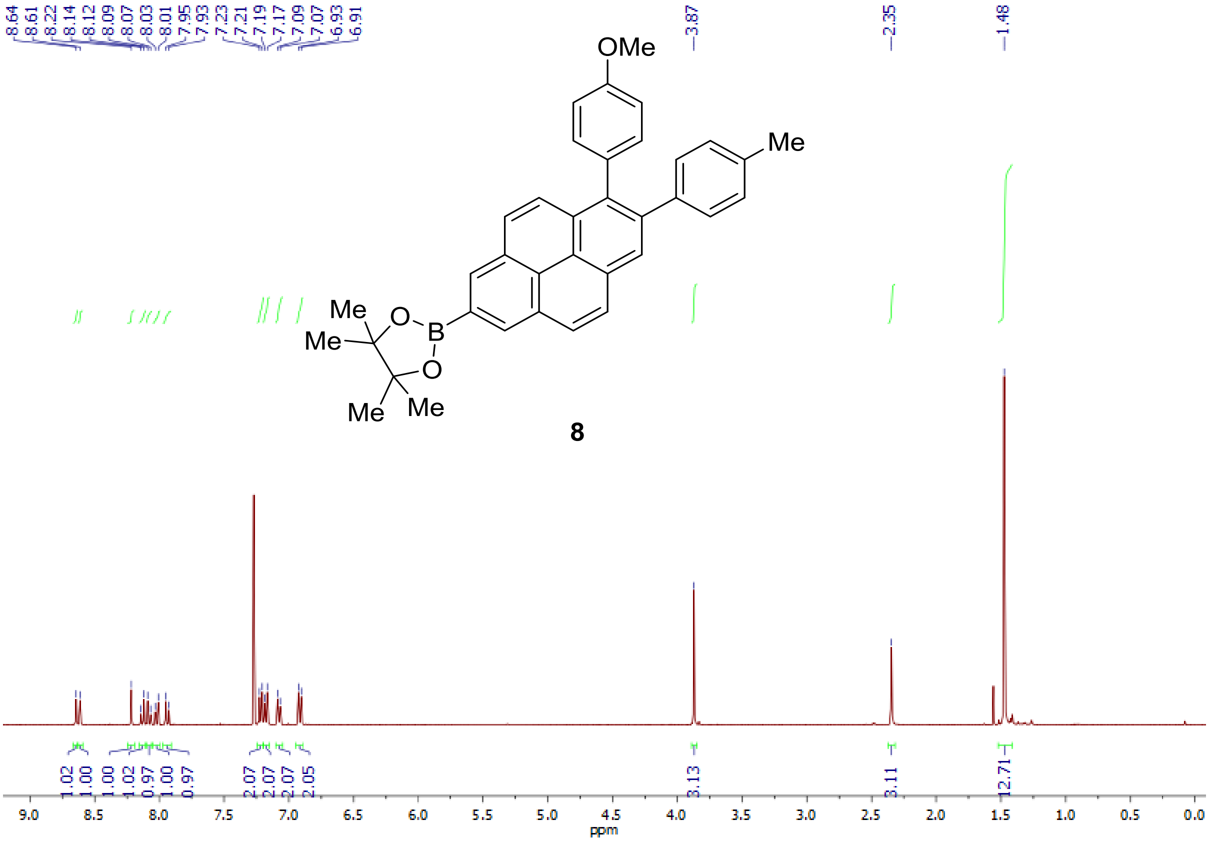

^{13}C-NMR (125 MHz, $CDCl_3$)

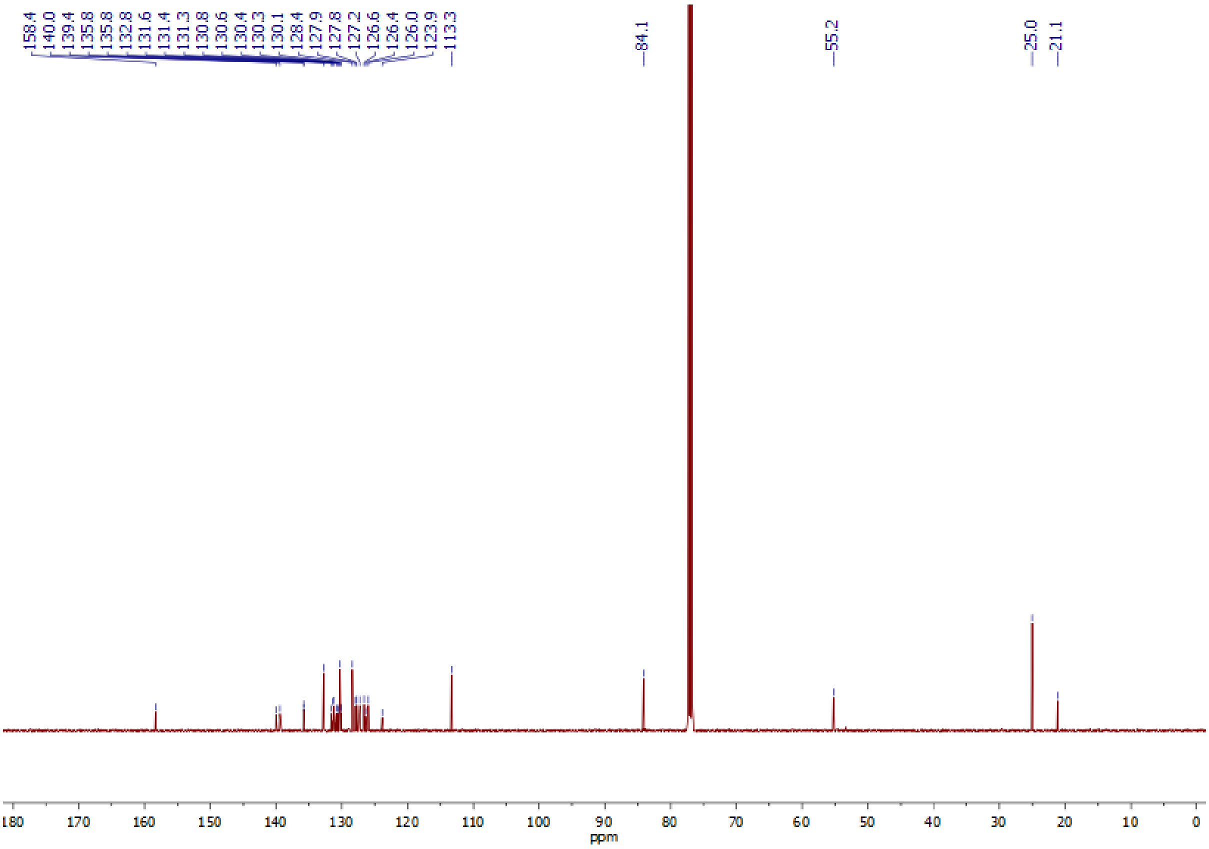

1-(Hydroxymethyl)-2-(*p*-tolyl)pyrene (9a)

^{1}H-NMR (500 MHz, $CDCl_3$)

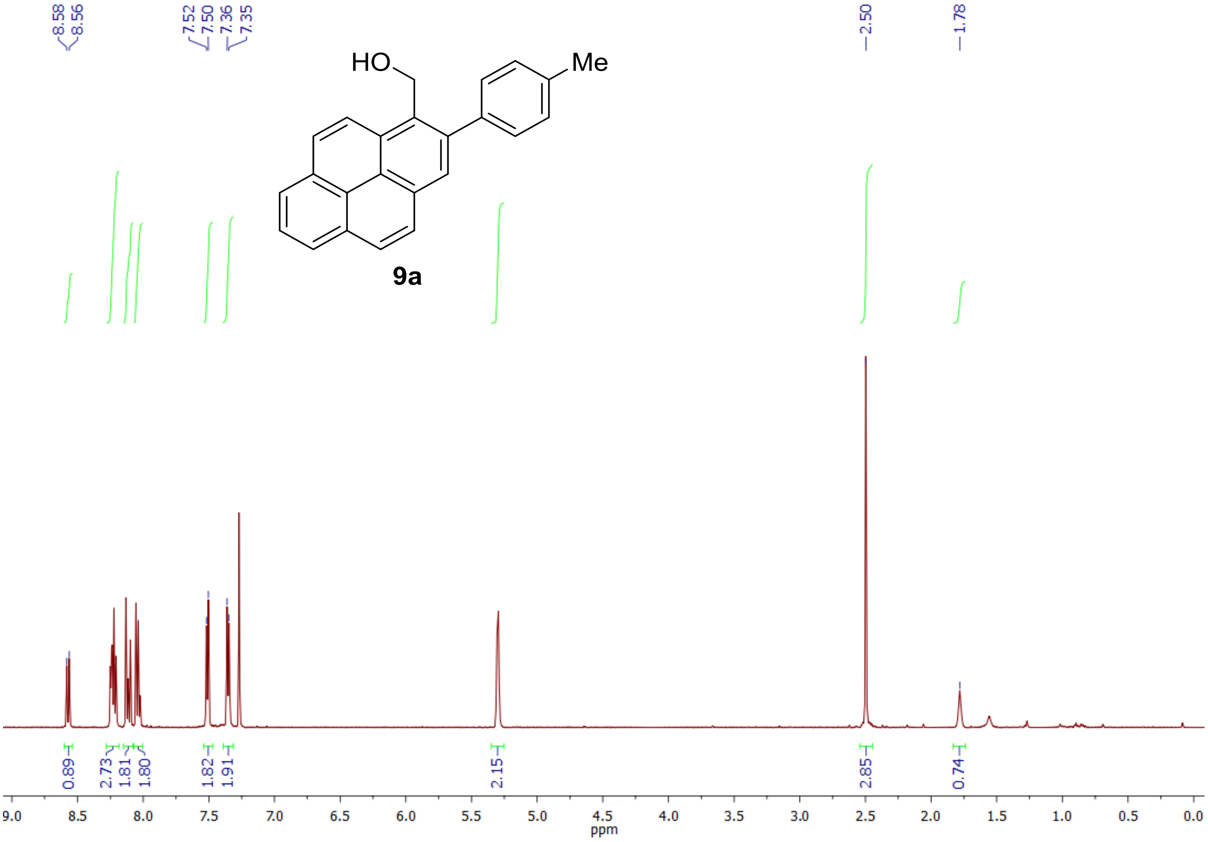

^{13}C-NMR (125 MHz, $CDCl_3$)

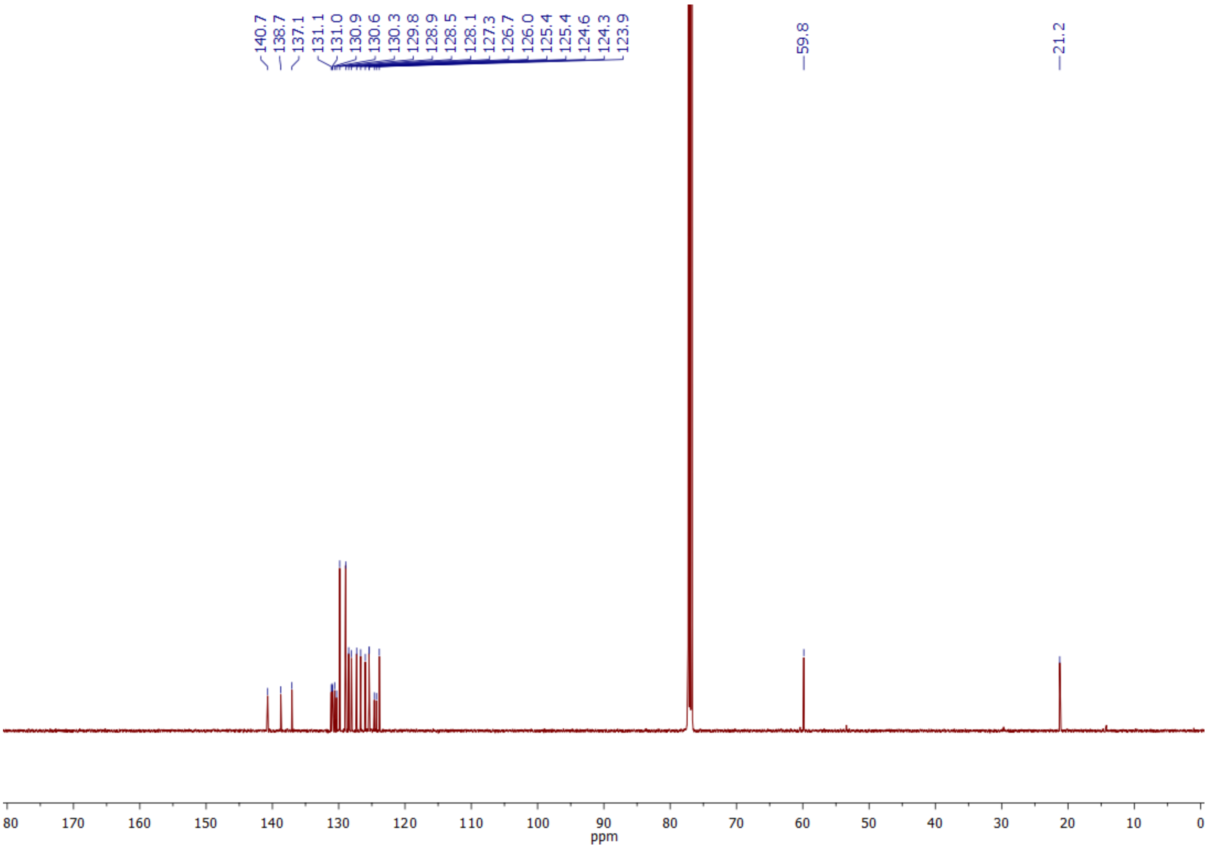

Methyl 4-(1-(hydroxymethyl)pyren-2-yl)benzoate (9b)

^{1}H-NMR (500 MHz, $CDCl_3$)

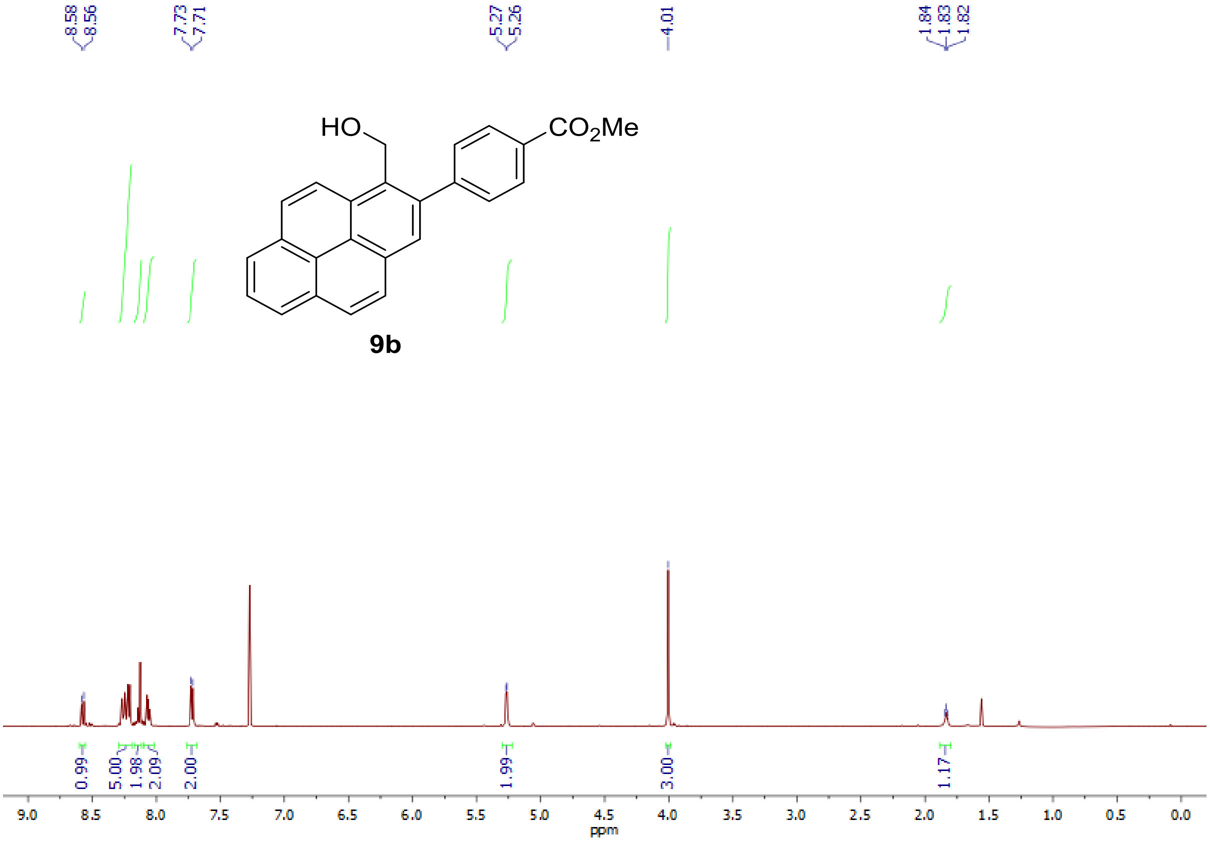

^{13}C-NMR (125 MHz, $CDCl_3$)

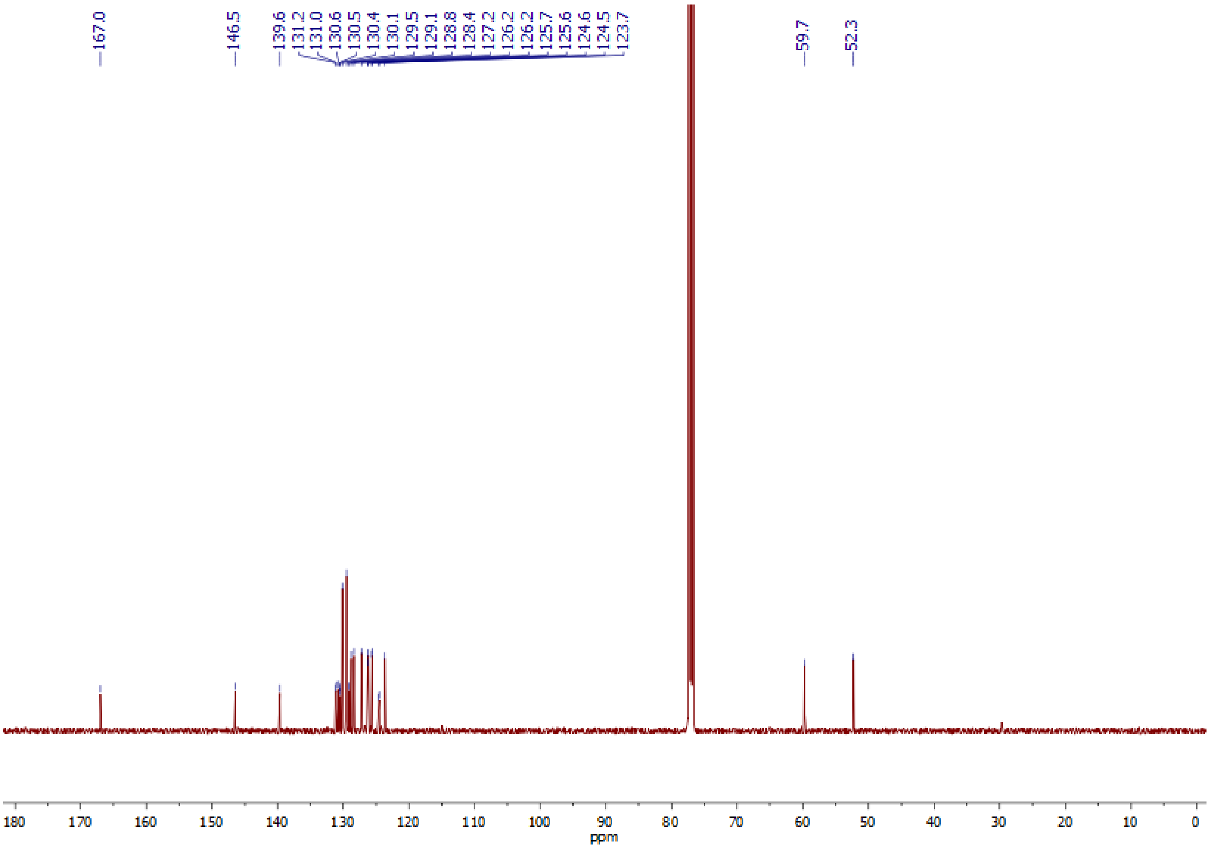

(2-(4-(Hydroxymethyl)phenyl)pyren-1-yl)methanol (9c)

^{1}H-NMR (500 MHz, MeOD-d$_4$)

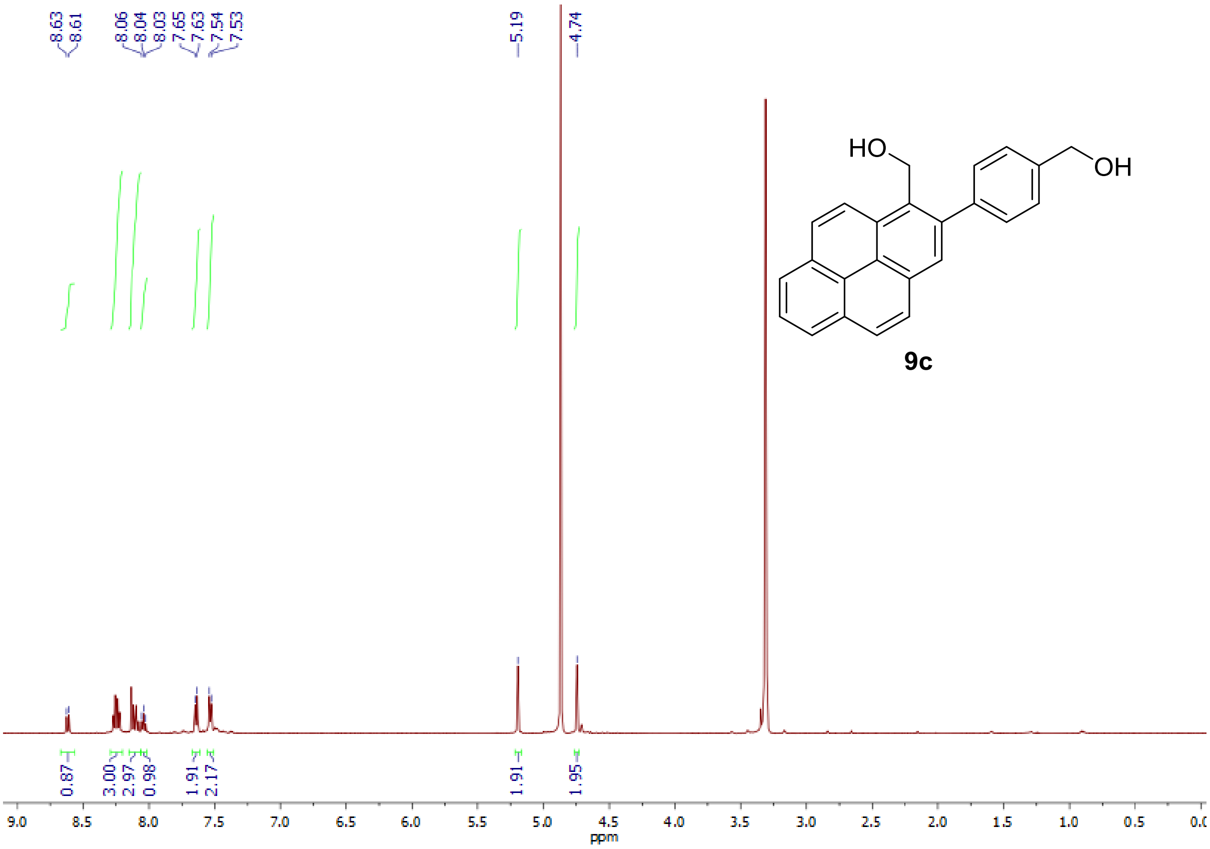

^{13}C-NMR (125 MHz, MeOD-d$_4$)

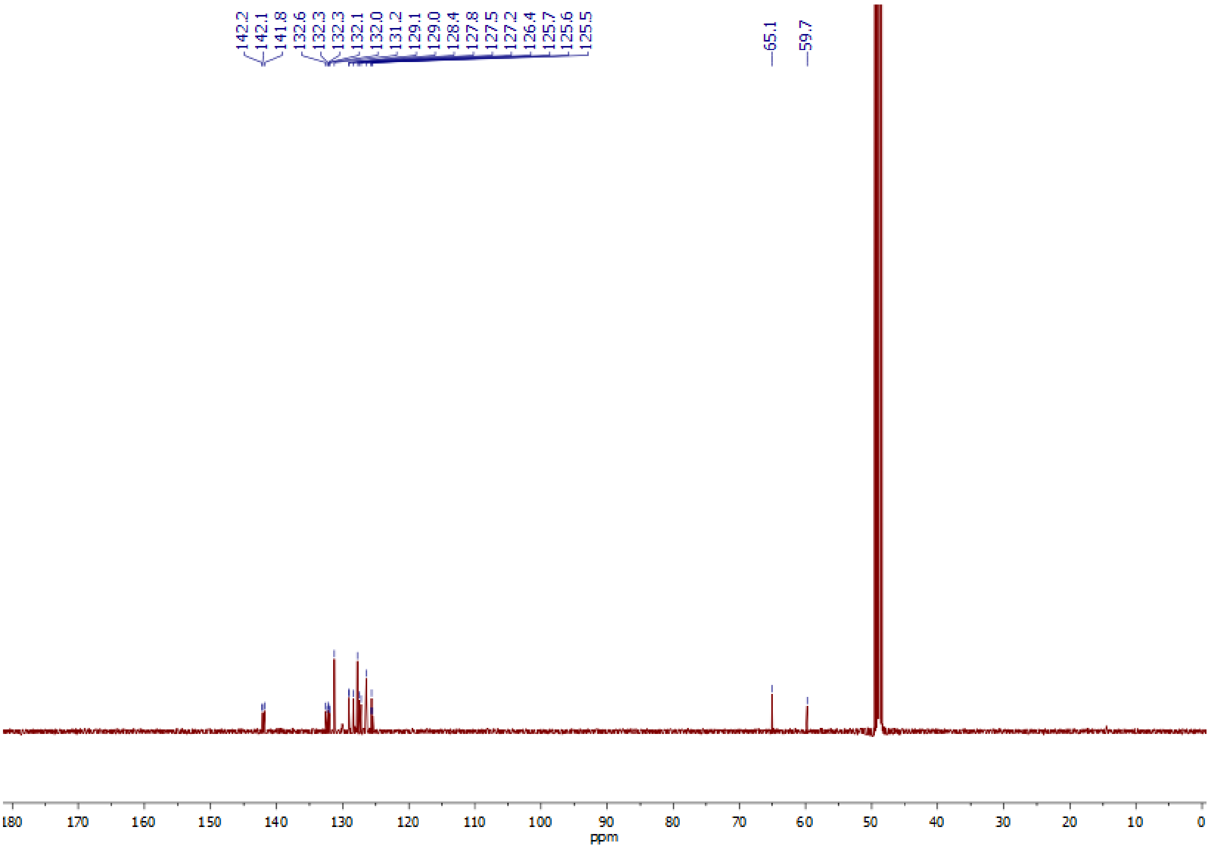

1-(Hydroxymethyl)-2-(3,5-bis(trifluoromethyl)phenyl)pyrene (9d)

^{1}H-NMR (500 MHz, acetone-d$_6$)

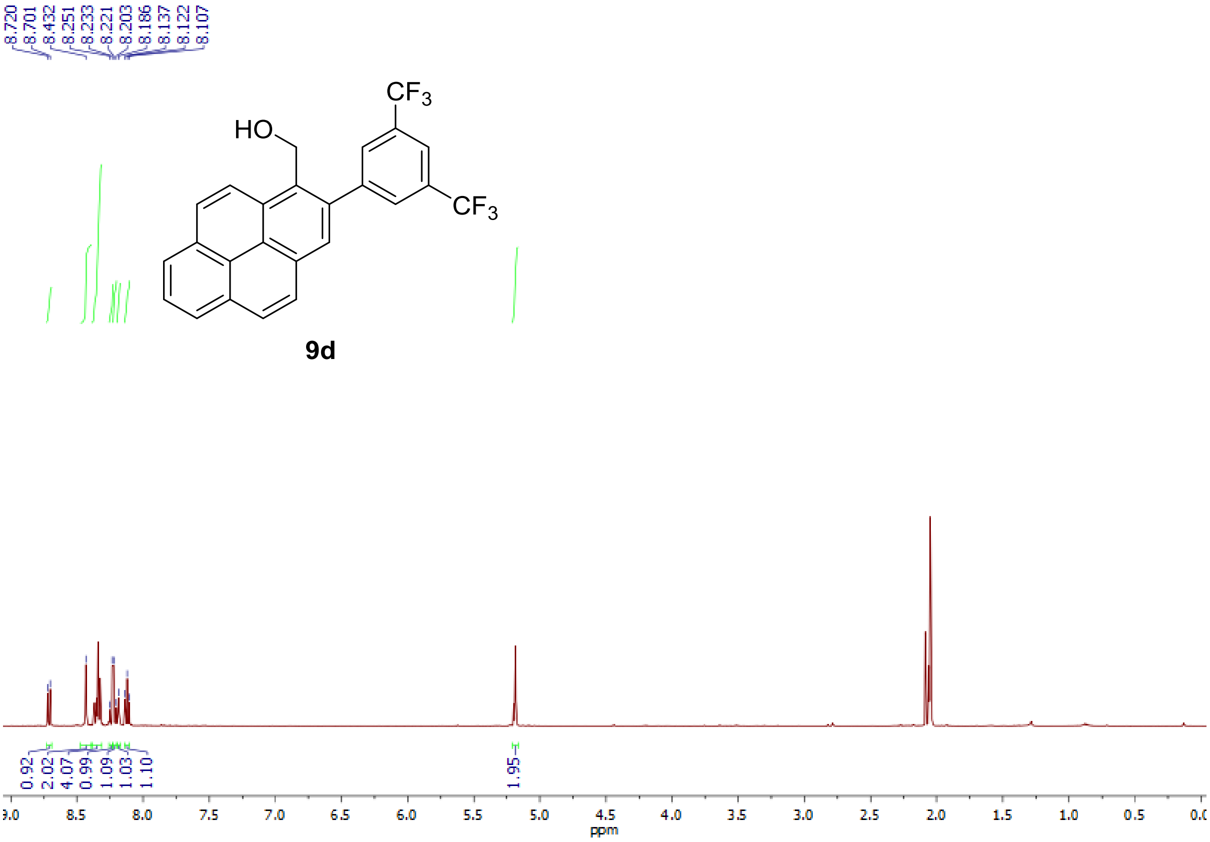

^{13}C-NMR (125 MHz, acetone-d$_6$)

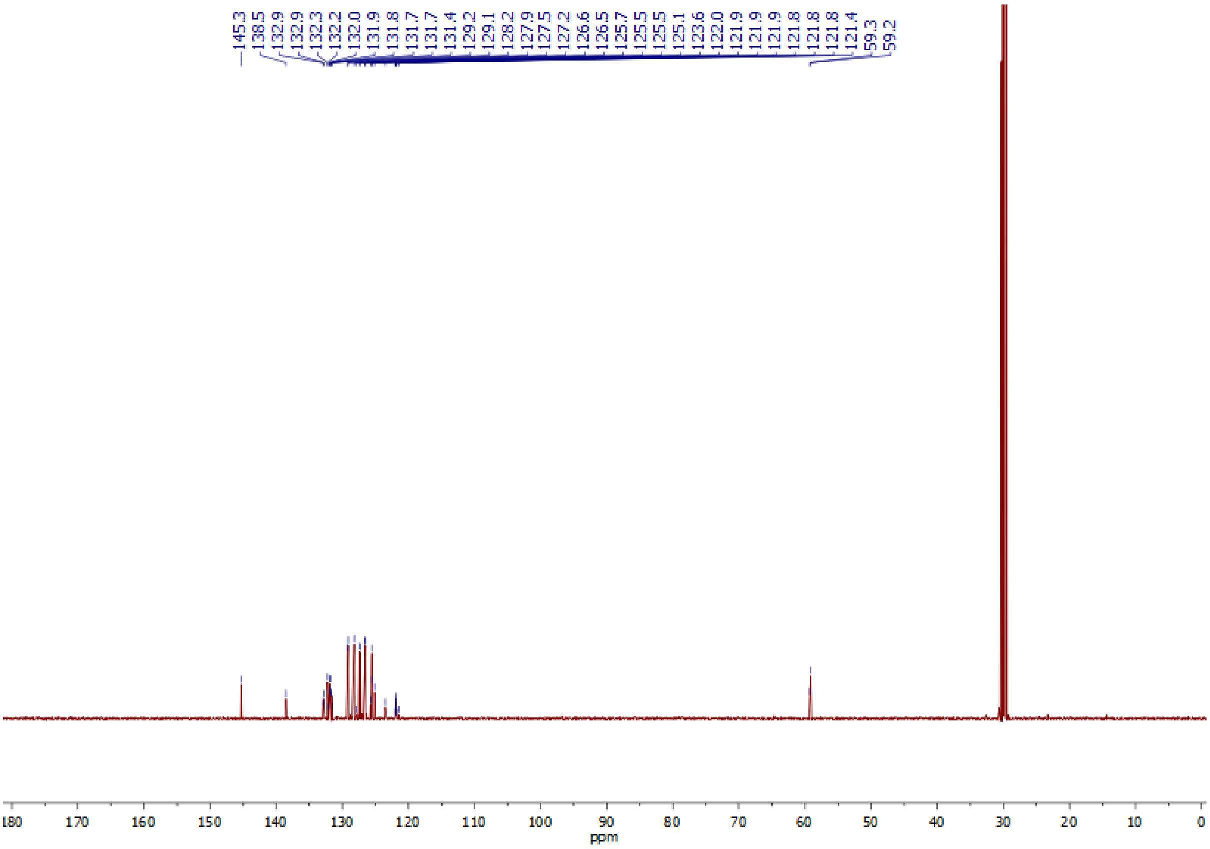

^{19}F-NMR (471 MHz, acetone-d$_6$)

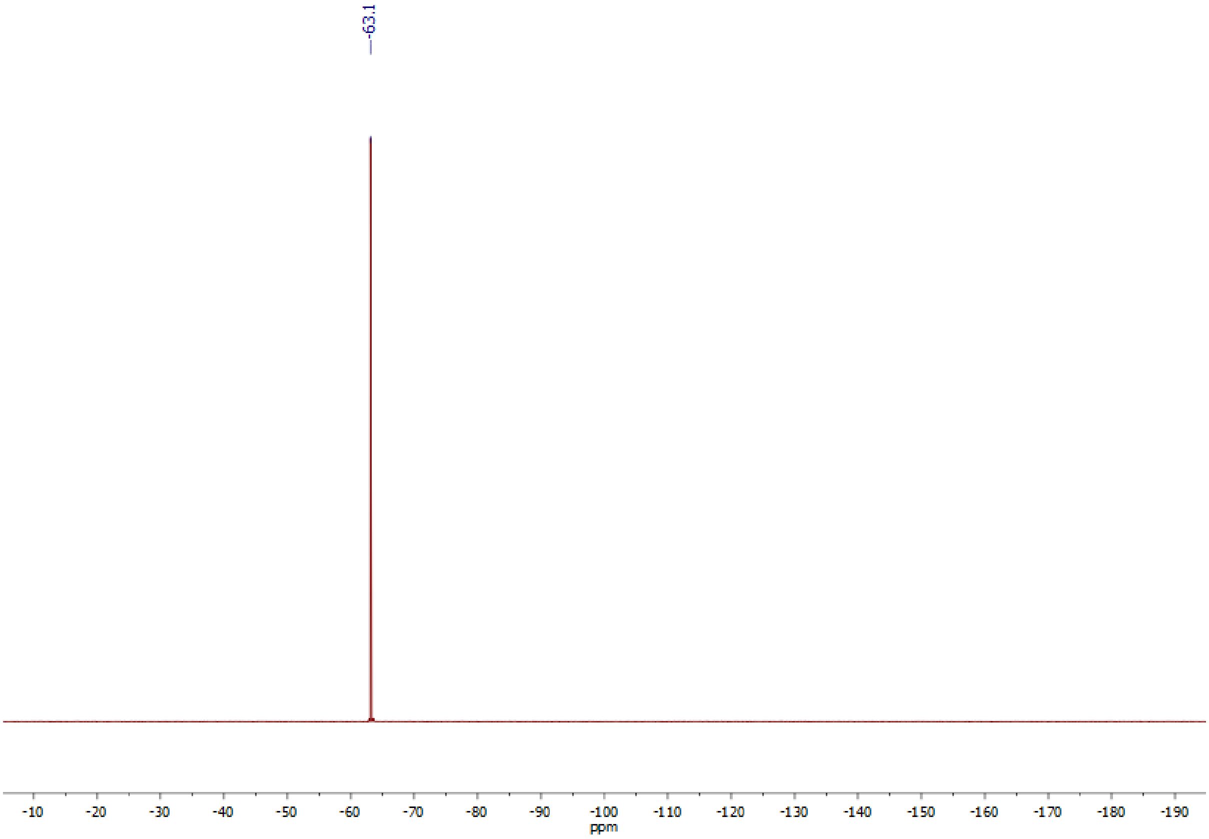

N,N,N-Trimethyl-1-(2-(*p*-tolyl)pyren-1-yl)methanammonium bromide (10a)

^{1}H-NMR (500 MHz, MeOD-d$_4$)

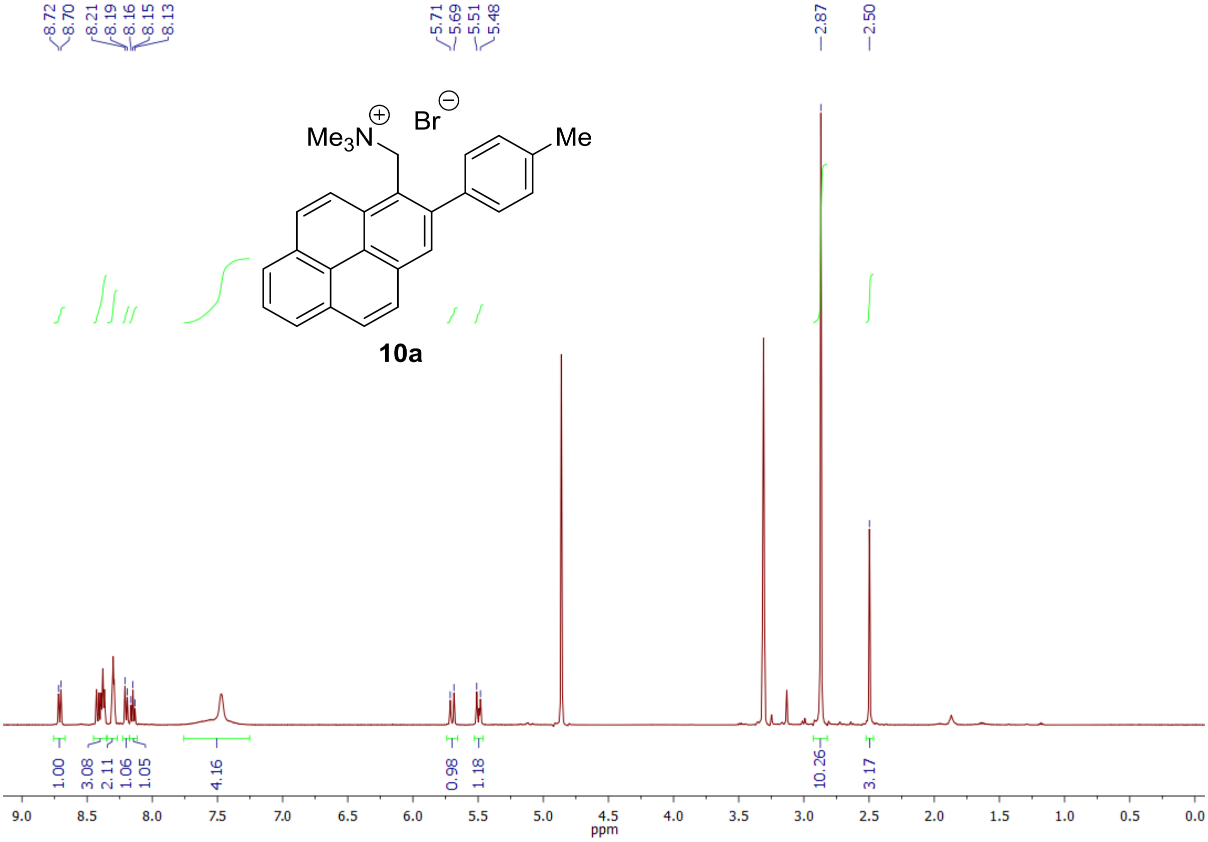

^{13}C-NMR (125 MHz, MeOD-d$_4$)

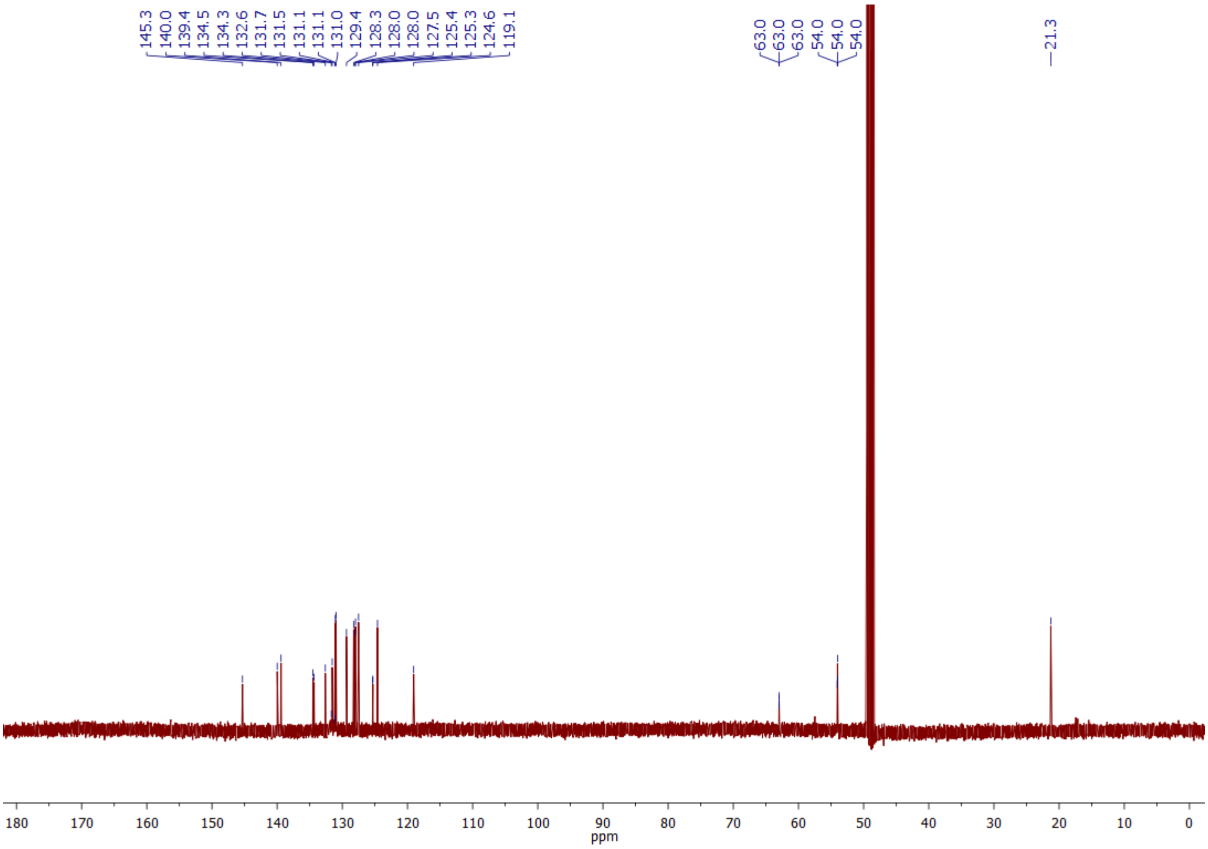

1-(2-(4-(Methoxycarbonyl)phenyl)pyren-1-yl)-*N*,*N*,*N*-trimethylmethanammonium bromide (10b)

^{1}H-NMR (400 MHz, MeOD-d_4)

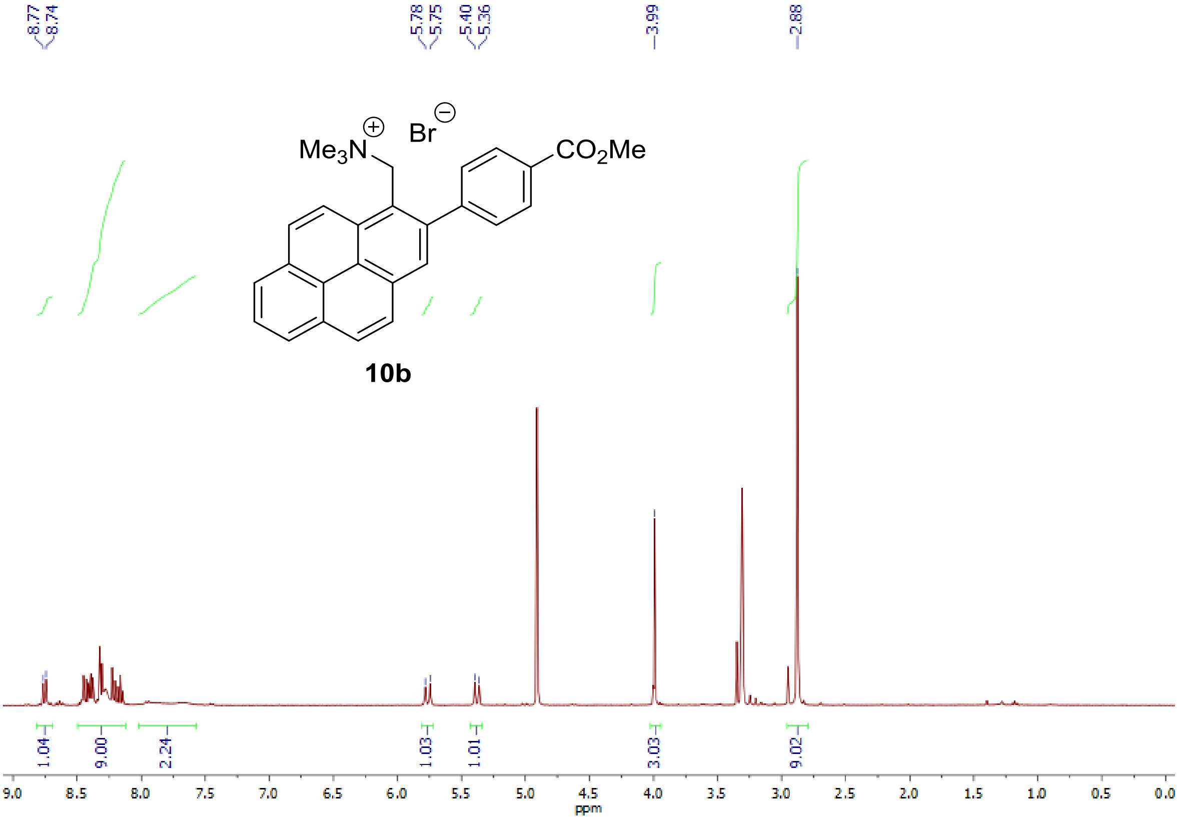

^{13}C-NMR (125 MHz, MeOD-d_4)

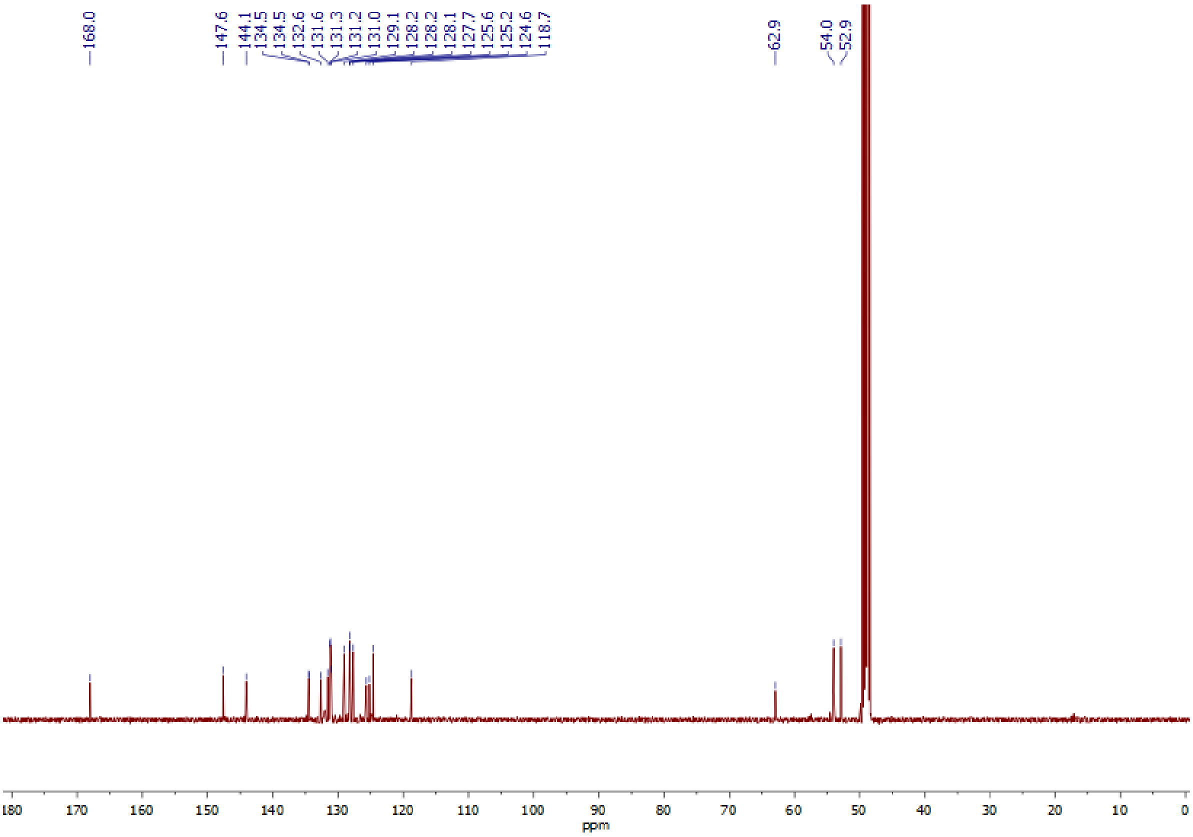

_N,N,N_-trimethyl-1-(2-(4-((trimethylammonio)methyl)phenyl)pyren-1-yl)methanammonium bromide (10c)

^{1}H-NMR (400 MHz, MeOD-d$_4$)

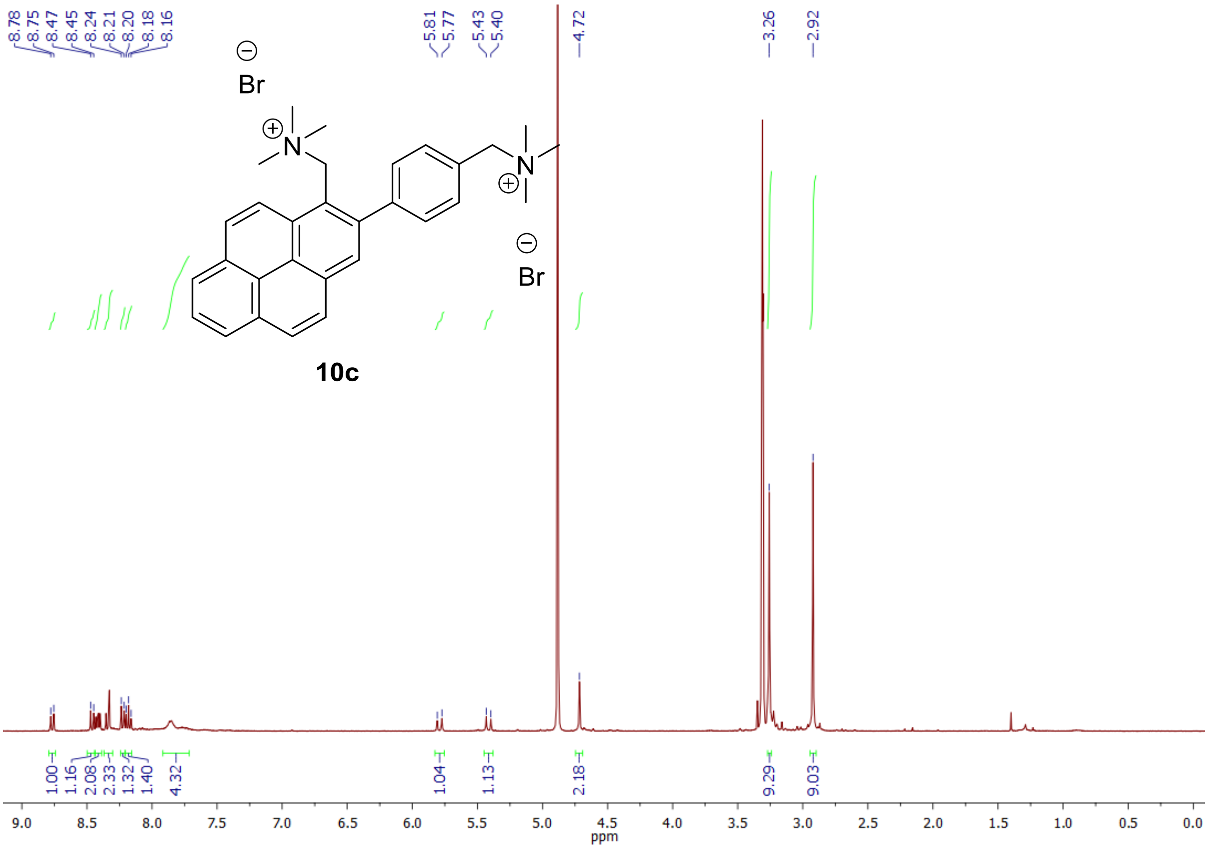

^{13}C-NMR (125 MHz, MeOD-d$_4$)

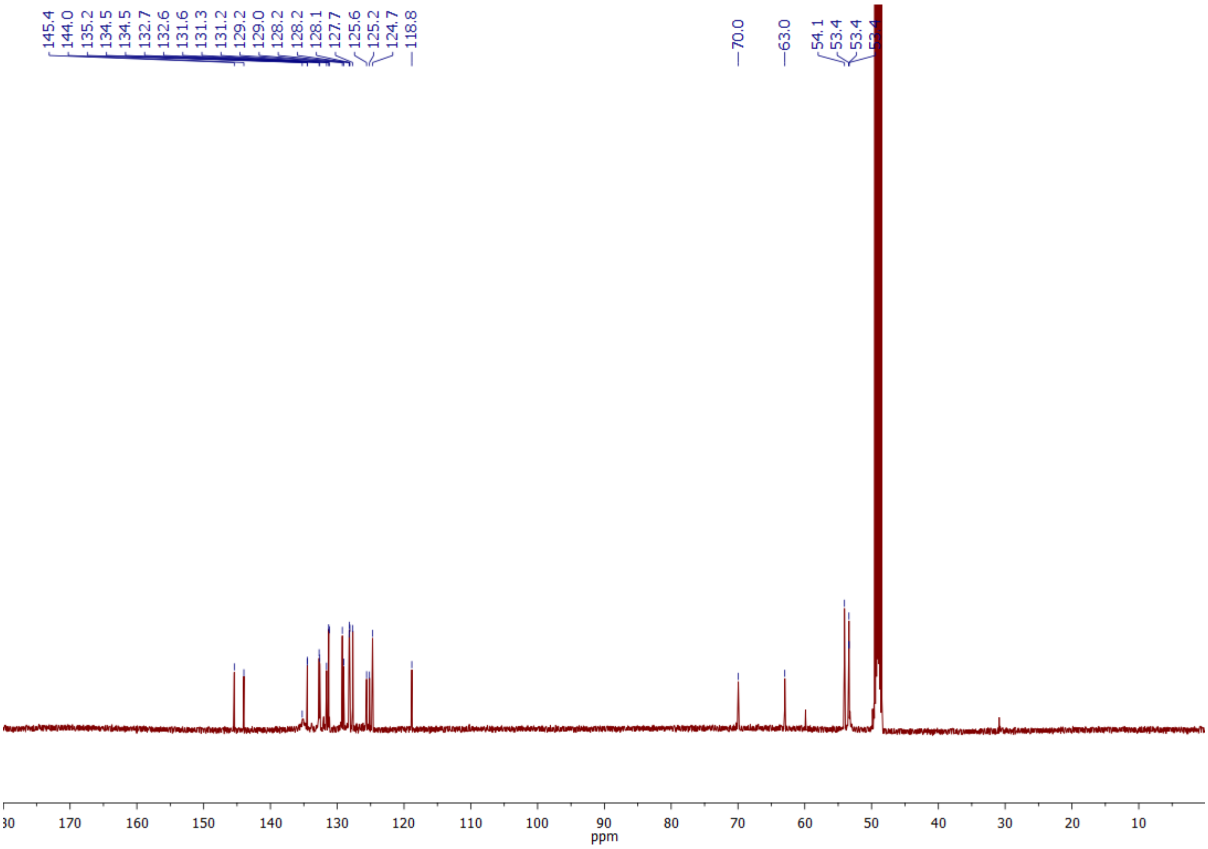

1-(2-(3,5-Bis(trifluoromethyl)phenyl)pyren-1-yl)-*N*,*N*,*N*-trimethylmethanammonium bromide (10d)

^{1}H-NMR (400 MHz, MeOD-d$_4$)

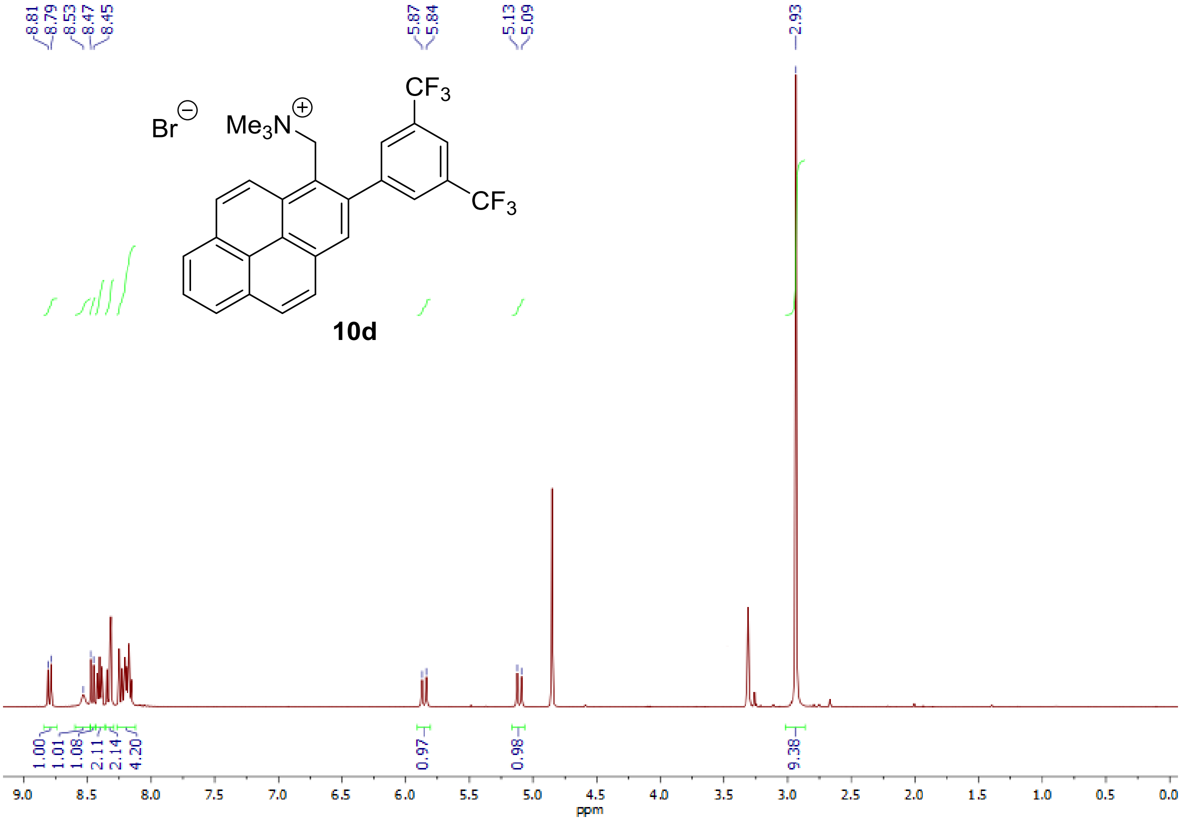

^{13}C-NMR (100 MHz, MeOD-d$_4$)

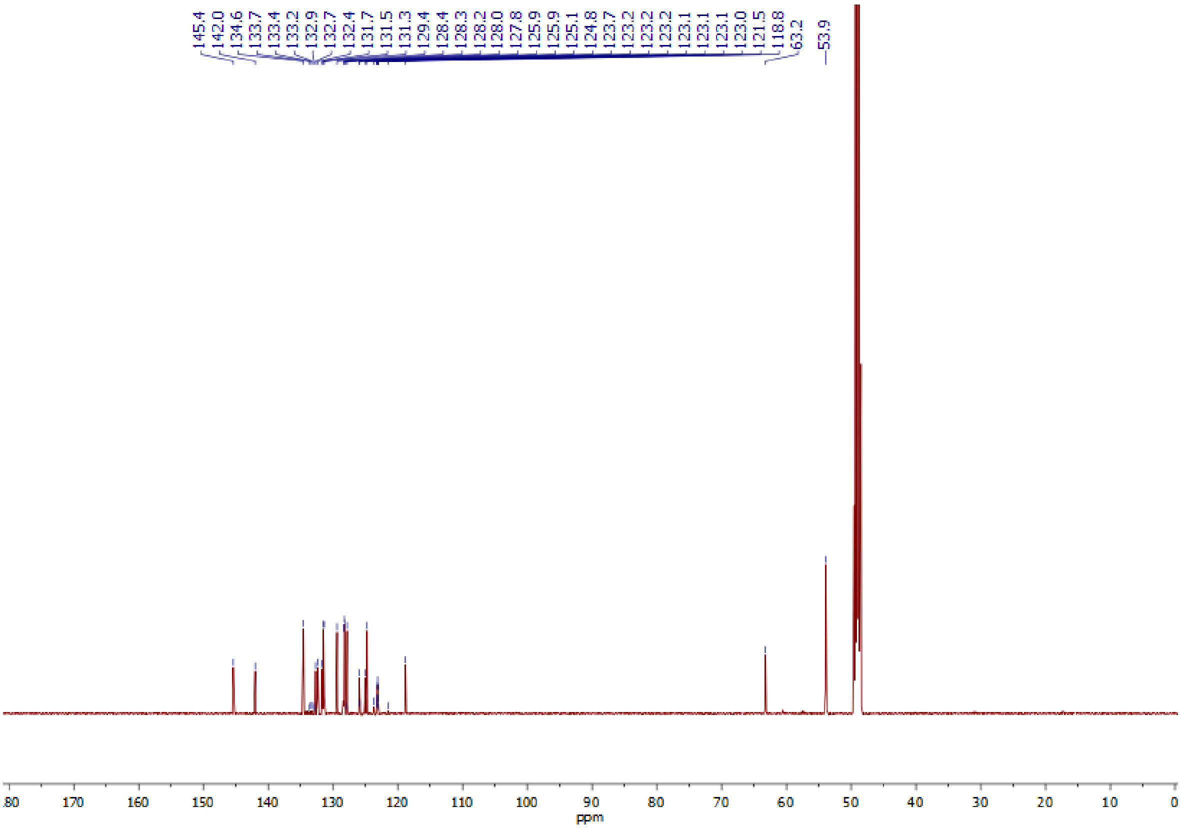

^{19}F-NMR (376 MHz, MeOD-d$_4$)

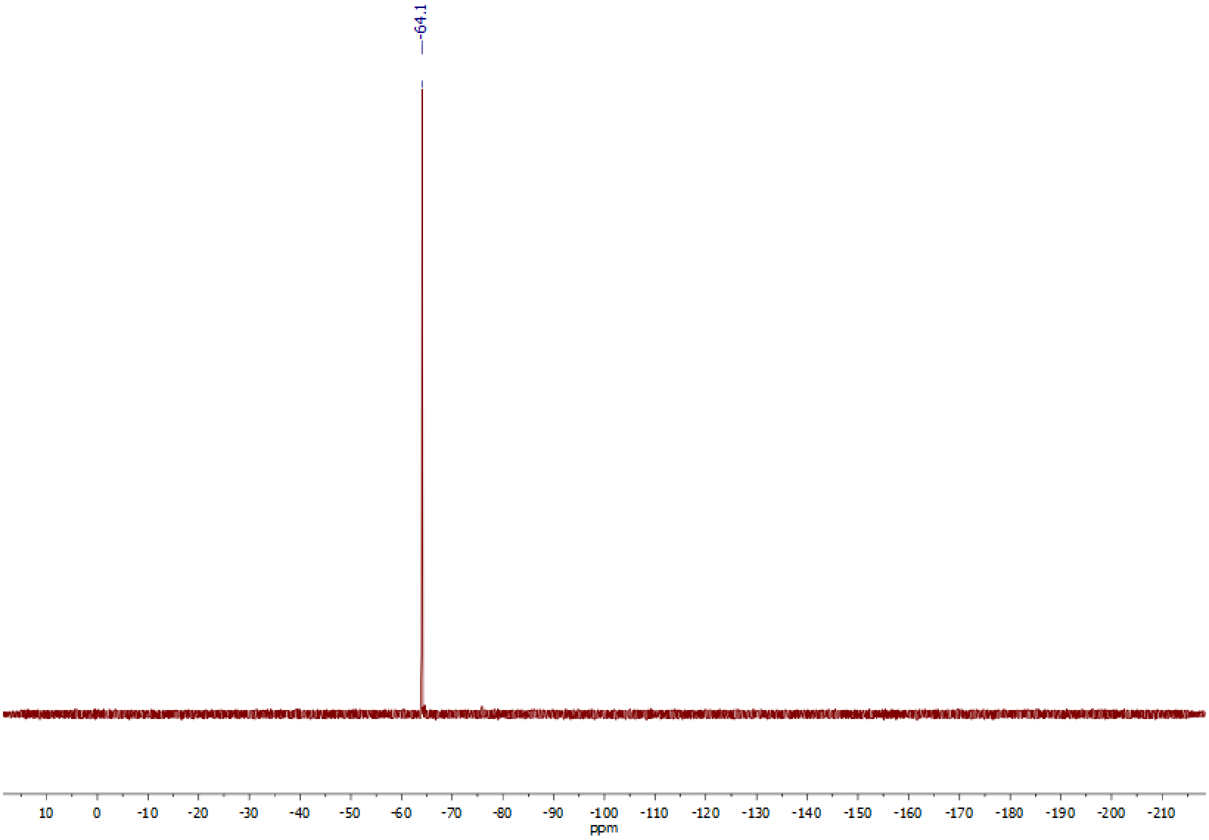

1-(Bromomethyl)pyrene (S1)

^{1}H-NMR (400 MHz, $CDCl_3$)

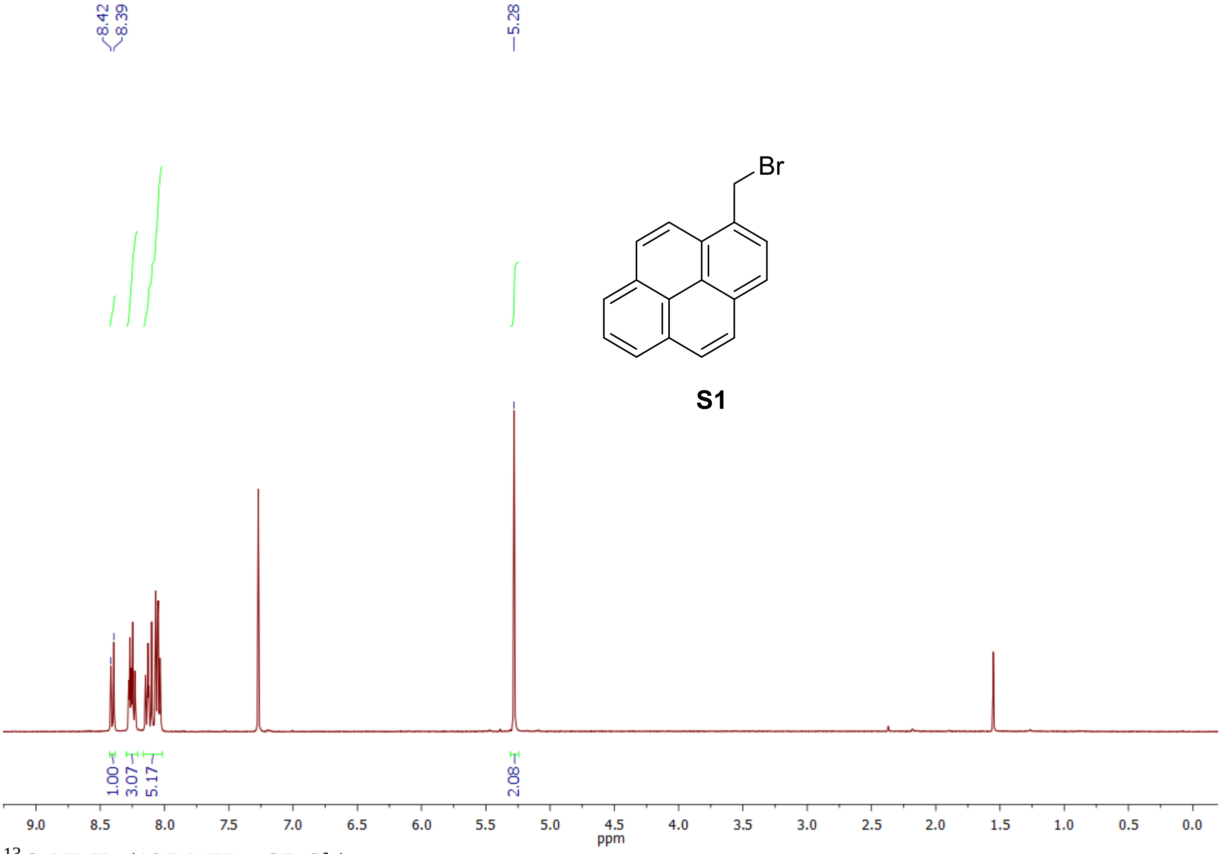

^{13}C-NMR (125 MHz, $CDCl_3$)

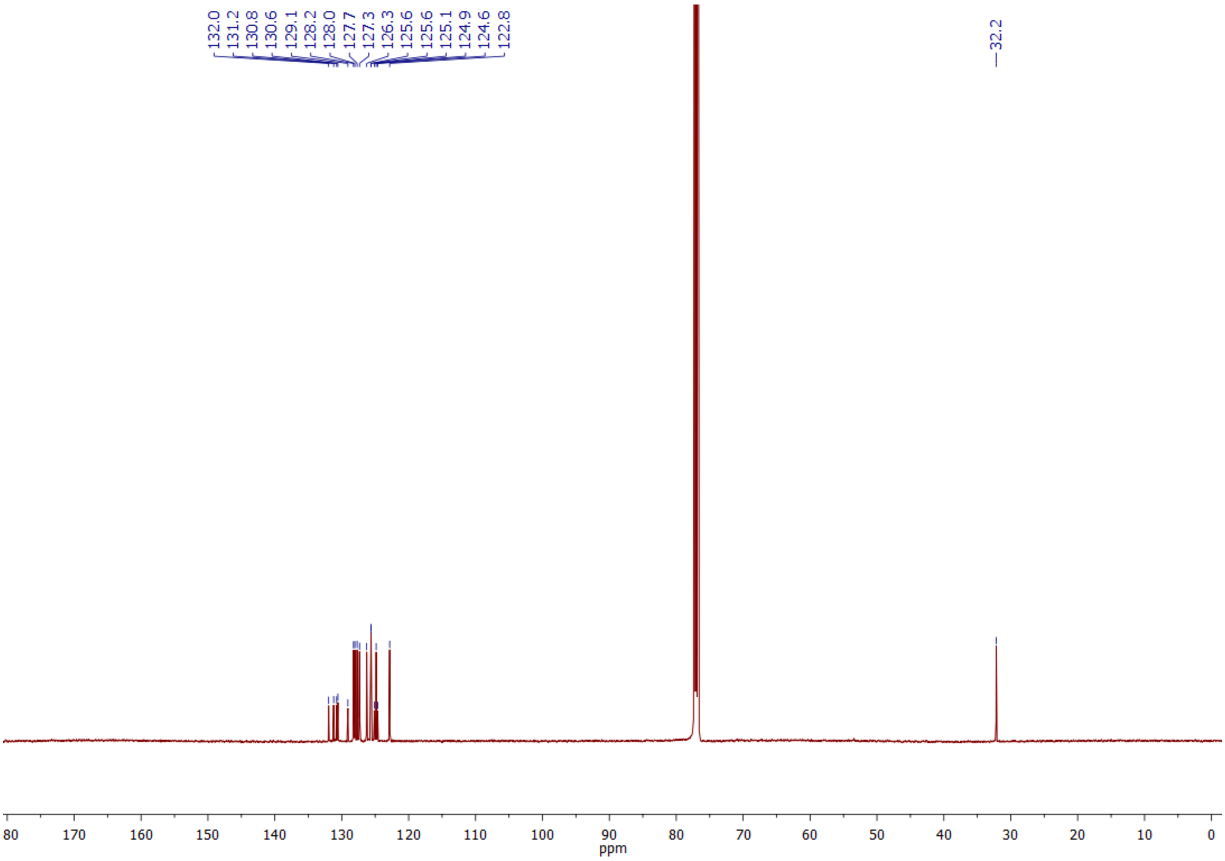

_N,N,N_-Trimethyl-_N_-(1-pyrenylmethyl)ammonium bromide (11)

^{1}H-NMR (500 MHz, MeOD-d_4)

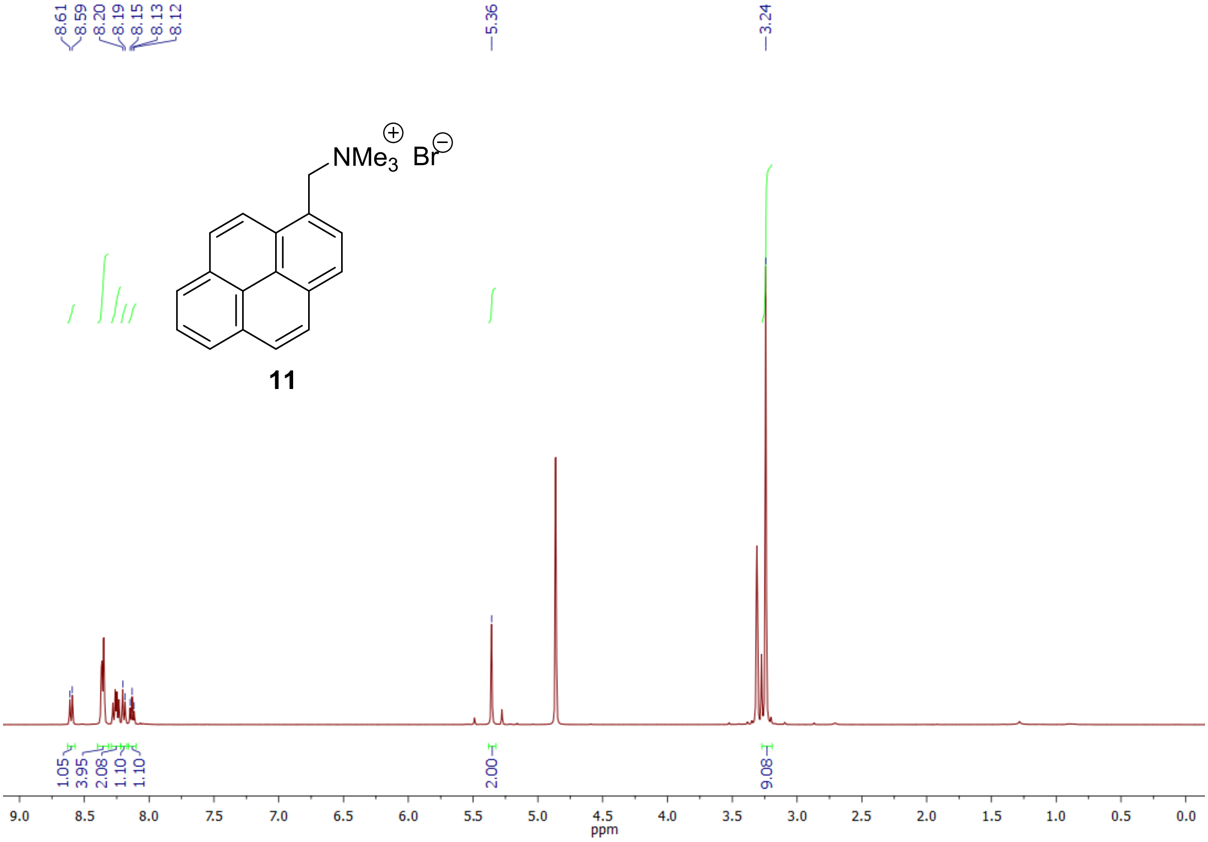

^{13}C-NMR (125 MHz, MeOD-d_4)

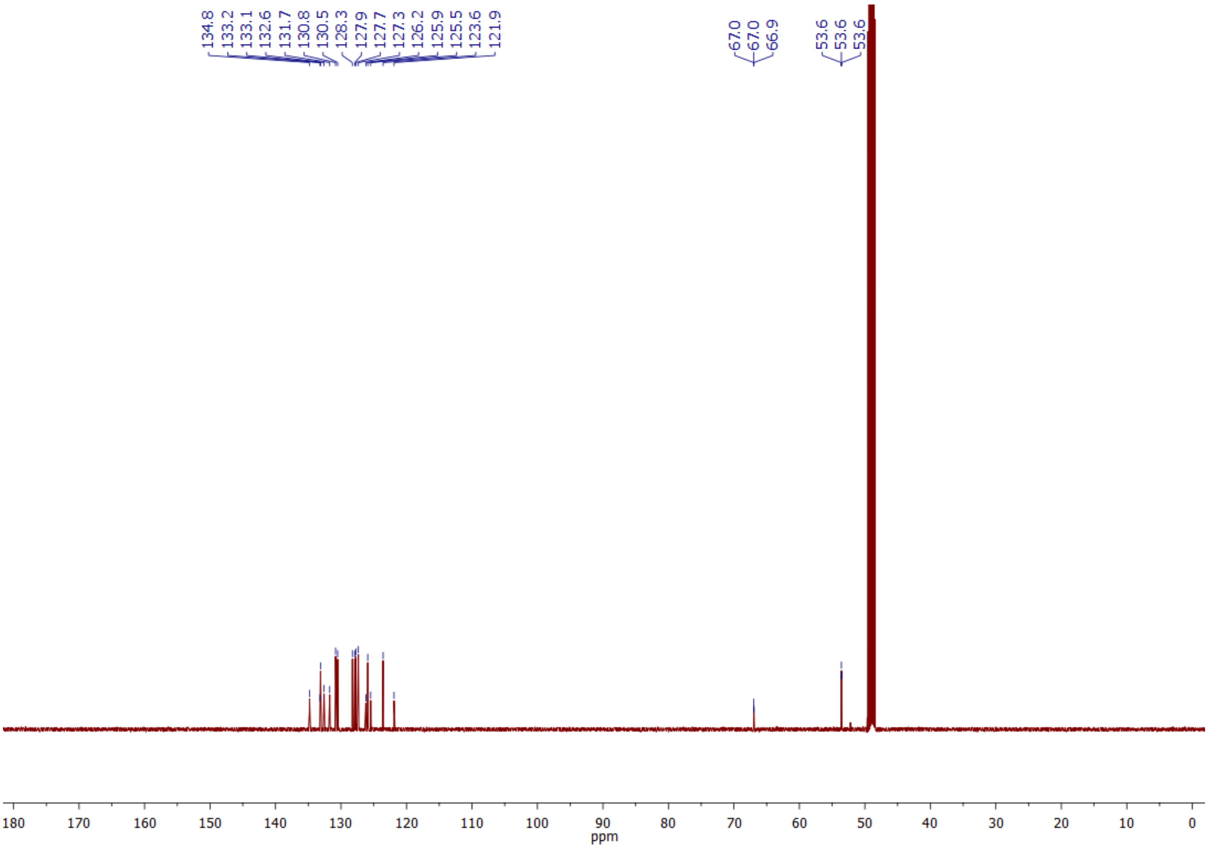

5. Liquid Phase Exfoliation

The LPE process was performed in the following way: 30 mg of graphite (Graphexel ltd.) was added to 10 mL of de-ionized water, previously mixed with 4 mg of the prepared pyrene derivatives. The mixture was sonicated at 600 W for 7 days using a Hilsonic bath sonicator fit with a chiller to keep temperature constant at 20 ºC. Un-exfoliated graphite was removed by 2-steps centrifugation at 3500 rpm (903 g) for 20 minutes. After each centrifugation step, the supernatant containing dispersed exfoliated graphite in water was collected and analysed. Dispersions were diluted (×10) for visual inspection.

6. AFM Analysis

A Bruker Atomic Force Microscope (MultiMode 8) in Peak Force Tapping mode, equipped with ScanAsyst-Air tips is used to determine the lateral size distribution of the flakes. The sample was prepared by drop casting the solution on a clean silicon substrate and subsequent annealing at 250 °C for 2 hours. Lateral dimension and thickness distributions of graphene nanosheets were carried out using Gwyddion scanning probe microscopy data processing software (Figure S1).

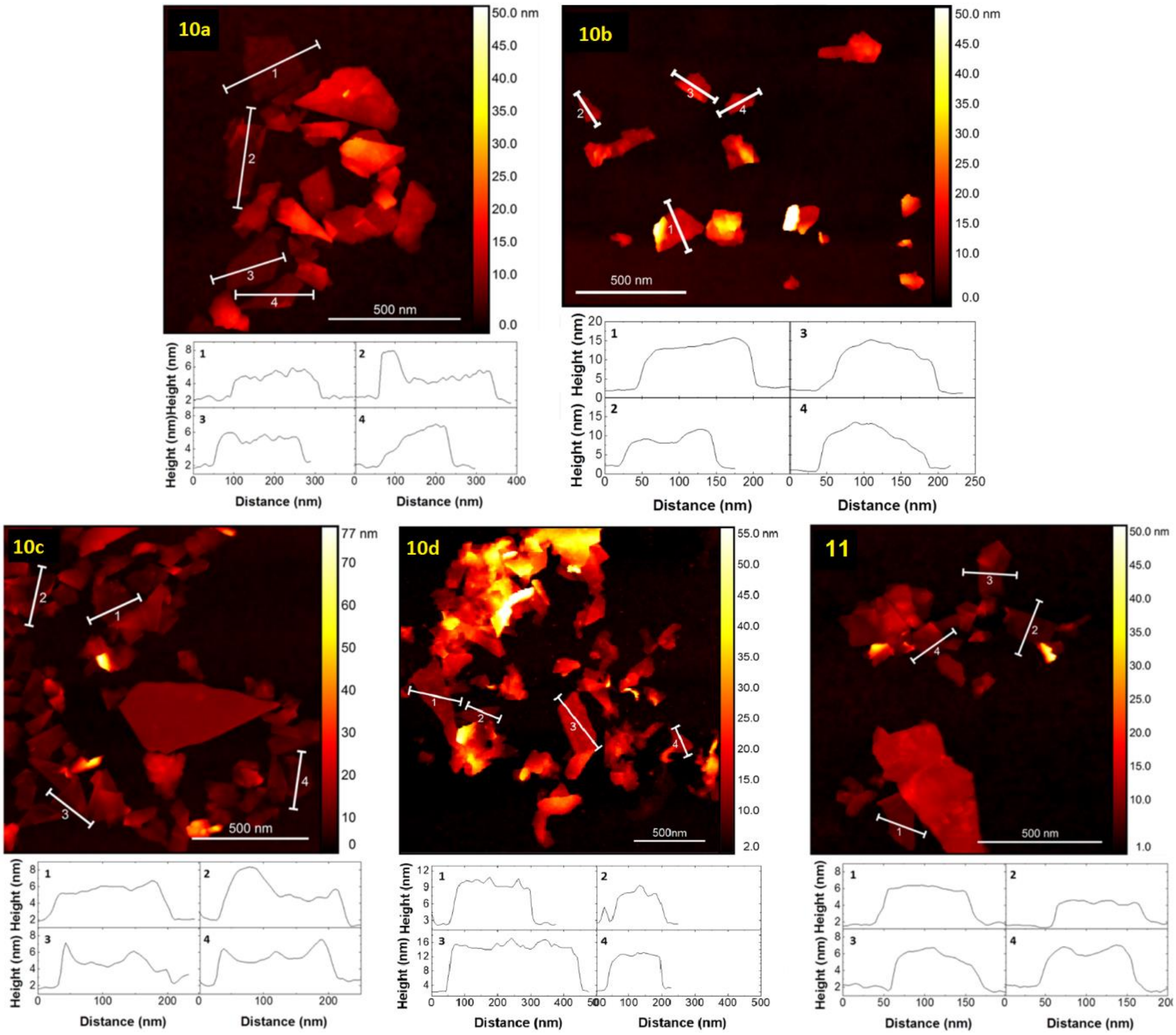

Figure S1. AFM images of graphene nanosheets drop-casted on silicon substrate.

7. Raman Analysis

Micro Raman measurements were carried out with a Renishaw Invia Raman spectrometer with excitation energy of 514.5 nm at 1.0 mW power. A 100X objective with a NA of 0.85 and 2400 grooves/mm grating was used for the characterization. Graphene dispersions were drop cast onto silicon substrates and measurements were performed on isolated and individual flakes. Typically about 30 flakes were measured for each sample (Table S5). The Raman peaks are fitted with Lorentzian lineshape.

Table S5. Raman analysis summary.

Compound	SLG[a]	FLG[b]	Thick layers	Total (%)
**10a**	20.0%	80.0%	0%	100.0
**10b**	6.7%	93.3%	0%	100.0
**10c**	6.7%	93.3%	0%	100.0
**10d**	5.9%	94.1%	0%	100.0
**11**	26.7%	73.3%	0%	100.0

[a]Single-layer graphene. [b]Few-layer graphene.

The Raman analysis is performed using a qualitative protocol for analyzing LPE graphene, which was introduced in our previous works.[1] In details, the 2D peak was fitted with a Lorentzian lineshape and the coefficient of determination, R^2, was used to discriminate between a single layer ($n = 1$, symmetric peak), a few layers ($2 \leq n \leq 10$, asymmetric 2D peak) and graphitic ($n > 10$ with AB stacking, characteristic peak shape with shoulder) material. Figure S2 shows typical Raman spectra corresponding to LPE single-layer graphene (SLG), few-layer graphene (FLG) and thick flakes measured on the graphene dispersions obtained with pyrene derivatives. This analysis provides qualitative information on the thickness distribution.

1 a) A. Ciesielski, S. Haar, M. El Gemayel, H. Yang, J. Clough, G. Melinte, M. Gobbi, E. Orgiu, M. V. Nardi, G. Ligorio, V. Palermo, N. Koch, O. Ersen, C. Casiraghi, P. Samorì, *Angew. Chem. Int. Ed.*, 2014, **53**, 10355; b) A. Haar, A. Ciesielski, , J. Clough, H. Yang, R. Mazzaro, F. Richard, S. Conti, N. Merstorf, M. Cecchini, V. Morandi, C. Casiraghi, P. Samorì, *Small*, 2015, **11**, 1691; c) S. Haar, M. El Gemayel, Y. Shin, G. Melinte, M. A. Squillaci, O. Ersen, C. Casiraghi, A. Ciesielski, P. Samorì, *Sci. Rep.*, 2015, **5**, 16684; d) S. Conti, M. G. del Rosso, A. Ciesielski, J. Weippert, A. Böttcher, Y. Shin, G. Melinte, O. Ersen, C. Casiraghi, X. Feng, K. Müllen, M. M. Kappes, P. Samorì, M. Cecchini, *ChemPhysChem*, 2016, **17**, 352; e) Y. Shin, E. Prestat, K.-G. Zhou, P. Gorgojo, K. Althumayri, W. Harrison, P. M. Budd, S. J. Haigh, C. Casiraghi, *Carbon*, 2016, **102**, 357.

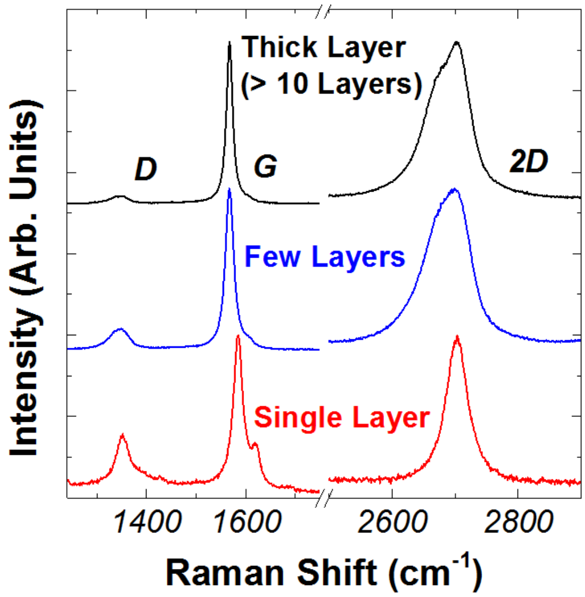

Figure S2. Representative Raman spectra of sample exfoliated with **10a** for graphene flakes with different thickness.

8. Zeta-Potential Measurements

Electrophoretic mobility (μ) was measured using a ZetaSizer Nano ZS (Malvern Instruments, UK) in the folded capillary cells at 25°C and the natural pH. The equipment software automatically converted the μ to zeta-potential (ζ) values by Henry's equation[2]: $\mu = 2\varepsilon\zeta\ F(\kappa a)/3\eta$ where ε is dielectric constant, η is the solution viscosity and F(κa) is the Henry's function which is approximated to the value of 1.5 using Smoluchowski approximation for polar media, valid for dispersed particles of any shape including plate-like particles. All values for samples are mean values, calculated from triplicate measurements.

9. UV-Vis Spectroscopy

The final concentration of graphene dispersed in the solution was determined using UV-Vis spectroscopy. The UV-Vis spectrum of graphene appears flat and featureless in the visible-IR region,[3] so the absorption is measured at 660 nm. The Beer-Lambert law is used to derive the concentration by assuming an absorption coefficient of 2460 $L\cdot g^{-1}\cdot m^{-1}$ at 660 nm.[4] A Perkin-Elmer l-900 UV-Vis-NIR spectrophotometer was used to acquire the spectra.

2 R. J. Hunter, Zeta potential in colloid science: Principles and applications. *Colloid Science*, 1981, *2*.

3 Y. Hernandez, V. Nicolosi, M. Lotya, F. M. Blighe, Z. Sun, S. De, I. T. McGovern, B. Holland, M. Byrne, Y. K. Gun'ko, J. J. Boland, P. Niraj, G. Duesberg, S. Krishnamurthy, R. Goodhue, J. Hutchison, V. Scardaci, A. C. Ferrari, J. N. Coleman, *Nat. Nanotechnol.*, 2008, **3**, 563.

4 a) U. Khan, A. O'Neil, M. Loyta, S. De, J. N. Coleman, *Small,* **2010**, 6, 864; b) M. Loyta, Y. Hernandez, P. J. King, R. J. Smith, V. Nicolosi, L. S. Karlsson, F. M. Blighe, S. De, Z. Wang, I. T. McGovern, G. S. Duesberg, J. N. Coleman, *J. Am. Chem. Soc.*, 2009, **131**, 3611; c) M. Loyta, P. J. King, U. Khan, S. De, J. N. Coleman, *ACS Nano,* 2010, **4**, 3155.
